# Author Correction: The pan-cancer lncRNA PLANE regulates an alternative splicing program to promote cancer pathogenesis

**DOI:** 10.1038/s41467-025-59086-6

**Published:** 2025-05-16

**Authors:** Liu Teng, Yu Chen Feng, Su Tang Guo, Pei Lin Wang, Teng Fei Qi, Yi Meng Yue, Shi Xing Wang, Sheng Nan Zhang, Cai Xia Tang, Ting La, Yuan Yuan Zhang, Xiao Hong Zhao, Jin Nan Gao, Li Yuan Wei, Didi Zhang, Jenny Y. Wang, Yujie Shi, Xiao Ying Liu, Jin Ming Li, Huixia Cao, Tao Liu, Rick F. Thorne, Lei Jin, Feng-Min Shao, Xu Dong Zhang

**Affiliations:** 1 Translational Research Institute, Henan Provincial People’s Hospital and People’s Hospital of Zhengzhou University, Academy of Medical Science, Zhengzhou University, Henan, China; 2 School of Medicine and Public Health, The University of Newcastle, Callaghan, NSW Australia; 3 Department of Molecular Biology, Shanxi Cancer Hospital and Institute, Shanxi, China; 4 School of Biomedical Sciences and Pharmacy, The University of Newcastle, Callaghan, NSW Australia; 5 Department of Breast Surgery, Shanxi Bethune Hospital, Shanxi, China; 6 Orthopaedics Department, John Hunter Hospital, Hunter New England Health, New Lambton, NSW Australia; 7 Children’s Cancer Institute Australia for Medical Research, University of New South Wales, Sydney, NSW Australia; 8 Department of Pathology, Henan Provincial People’s Hospital, Zhengzhou University People’s Hospital, Henan, China; 9 Department of Nephrology, Henan Provincial People’s Hospital, Zhengzhou University People’s Hospital, Henan, China

Correction to: *Nature Communications* 10.1038/s41467-021-24099-4, published online 18 June 2021

In the version of the article initially published there were errors in Fig. 2c and f. In Fig. 2c, the NCI-H226 si-PLANE.2 image was incorrect and from another experimental replicate of HCT116 si-PLANE.2. In Fig. 2f, the three images in A549. shPLANE.2 (-Dox, +Dox and Dox withdrawal) are incorrect, and from images of A549 si-ctrl, si-PLANE.1 from Fig. 2c and another experimental replicate of A549 si-PLANE.2, respectively. In Fig. 2f, the three images of stained cell colonies shown in H1299.shPLANE.1 (-Dox, +Dox and Dox withdrawal) are incorrect and from images of H1299 si-ctrl, si-PLANE.1 and parental from Fig. 2c respectively. The original and corrected Fig. 2c and f are shown below. The figure has been corrected in the PDF and HTML versions of the article. The raw data for Fig. 2 are included as Supplementary information alongside the online version of the article.

Fig. 1 Original and corrected Fig. 2c and f

Original Figure:

**Figure:**
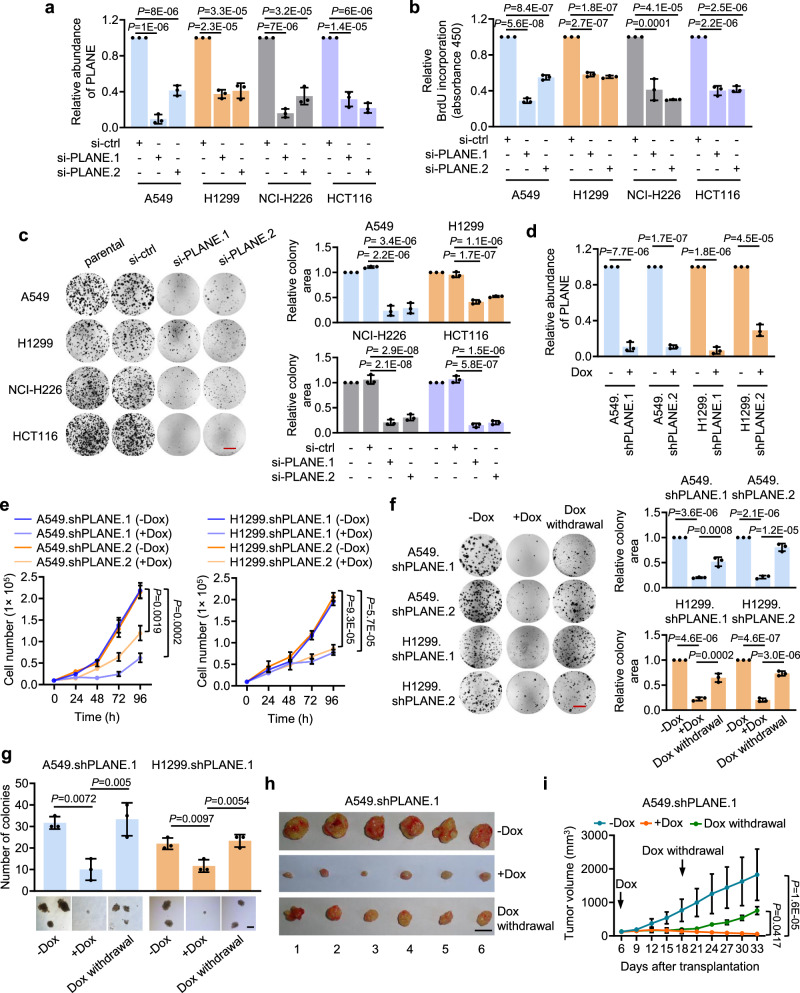

Corrected Figure:

**Figure:**
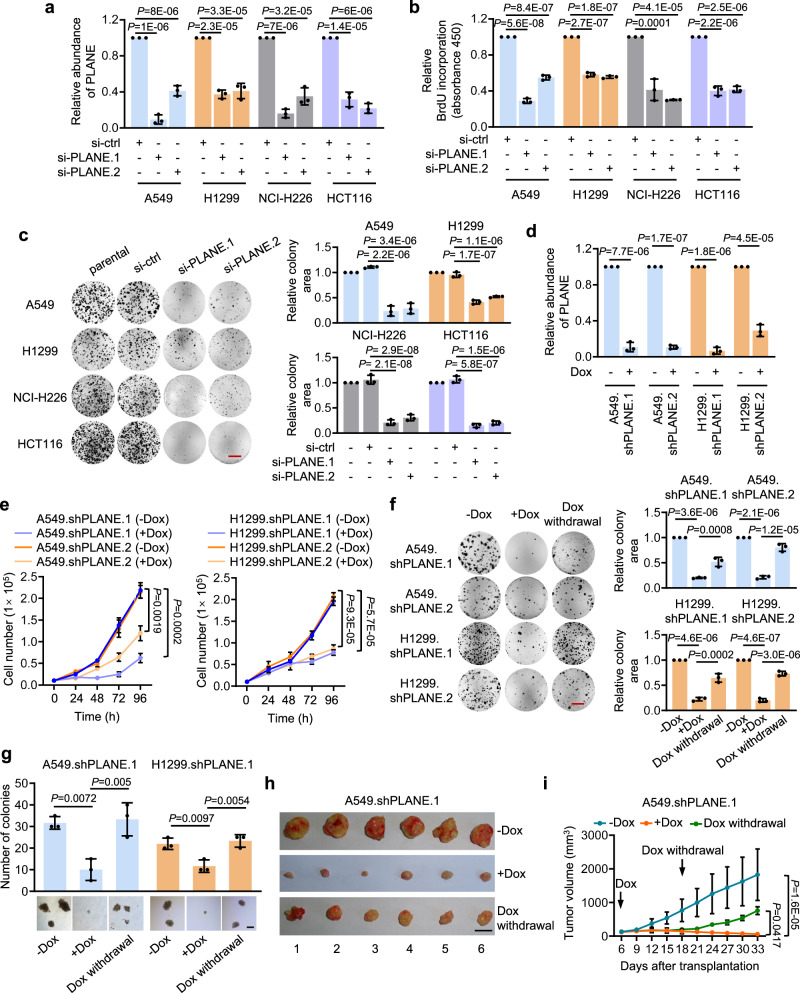

## Supplementary information

Raw data

