# Supplementary material for: The pan-cancer lncRNA PLANE regulates an alternative splicing program to promote cancer pathogenesis
Source: Nat Commun. 2021 Jun 18;12:3734. doi: 10.1038/s41467-021-24099-4 (PMC8213729; doi:10.1038/s41467-021-24099-4)
Supplement: Supplementary file 1 — Supplementary Information [file 41467_2021_24099_MOESM1_ESM.pdf]

Supplementary Figure 1

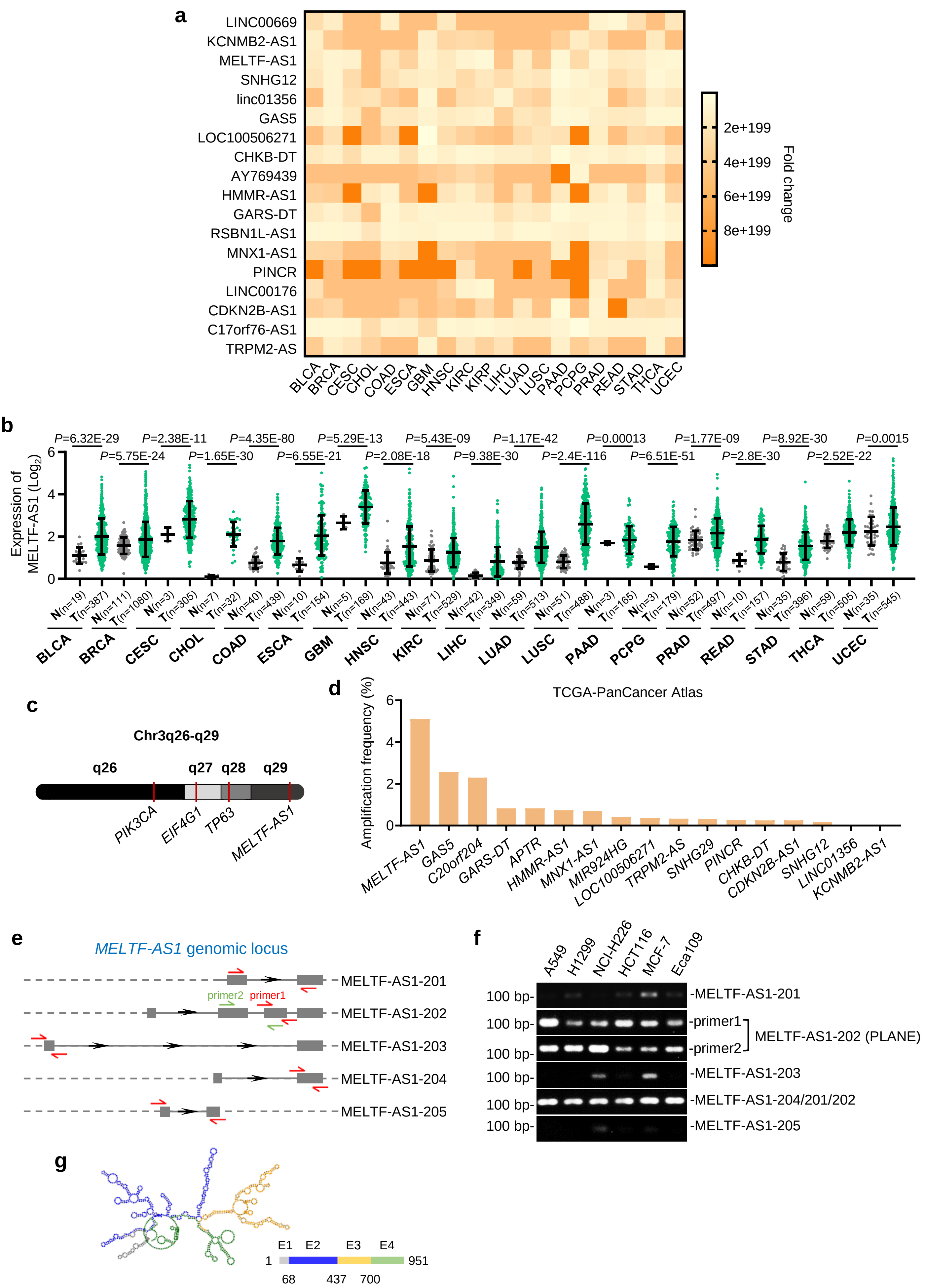

Supplementary Figure 1. MELTF-AS1 (PLANE) is a pan-cancer-associated lncRNA

a Identification of pan-cancer-associated lncRNAs through analysis of the lncRNA expression data from the TCGA dataset. Eighteen lncRNAs that are increased in at least 19 of 20 types of cancer relative to corresponding normal tissues are depicted using a heatmap. The data are fold changes in cancer compared with normal tissues. BLCA: bladder urothelial carcinoma; BRCA: breast invasive carcinoma; CESC: cervical squamous cell carcinoma and endocervical adenocarcinoma; CHOL: cholangiocarcinoma; COAD: colon adenocarcinoma; ESCA: esophageal carcinoma; GBM: glioblastoma multiforme; HNSC: head and neck squamous cell carcinoma; KIRC: kidney renal clear cell carcinoma; KIRP: kidney renal papillary cell carcinoma; LIHC: liver hepatocellular carcinoma; LUAD: lung adenocarcinoma; LUSC: lung squamous cell carcinoma; PAAD: pancreatic adenocarcinoma; PCPG: pheochromocytoma and paraganglioma; PRAD: prostate adenocarcinoma; READ: rectum adenocarcinoma; STAD: stomach adenocarcinoma; THCA: thyroid carcinoma; UCEC: uterine corpus endometrial carcinoma.

b MELTF-AS1 is upregulated in diverse types of cancer compared with corresponding normal tissues as revealed by analysis of the lncRNA expression data in the TCGA dataset. Data are mean ± s.d.; two-tailed Student's *t*-test. N: normal tissues; T: tumour tissues.

c Schematic illustration of the genomic location of the *MELTF-AS1* gene and representative protein-coding genes that are involved in cancer pathogenesis at chromosome 3q26-29.

d Analysis of copy-number alteration data of the PanCancer Atlas of the TCGA showing that *MELTF-AS1* was the most frequently amplified gene among those that encode the pan-cancer upregulated lncRNAs as shown in **a**.

e Schematic illustration of the five annotated MELTF-AS1 transcript isoforms. Filled boxes represent exons. The primers used for specific detection of each isoform were depicted in red and green. Primers directed against MELTF-AS1-204 span a common region shared by MELTF-AS1-201 and MELTF-AS1-202.

f PCR analysis showing that the longest isoform of MELTF-AS1, MELTF-AS1-202 (PLANE) was markedly more abundant than other isoforms in the indicated cancer cell lines. Data are representatives of 3 independent experiments.

g The secondary structure model of PLANE is predicted based on minimum free energy algorithm.

Source data are provided as a Source Data file.

Supplementary Figure 2

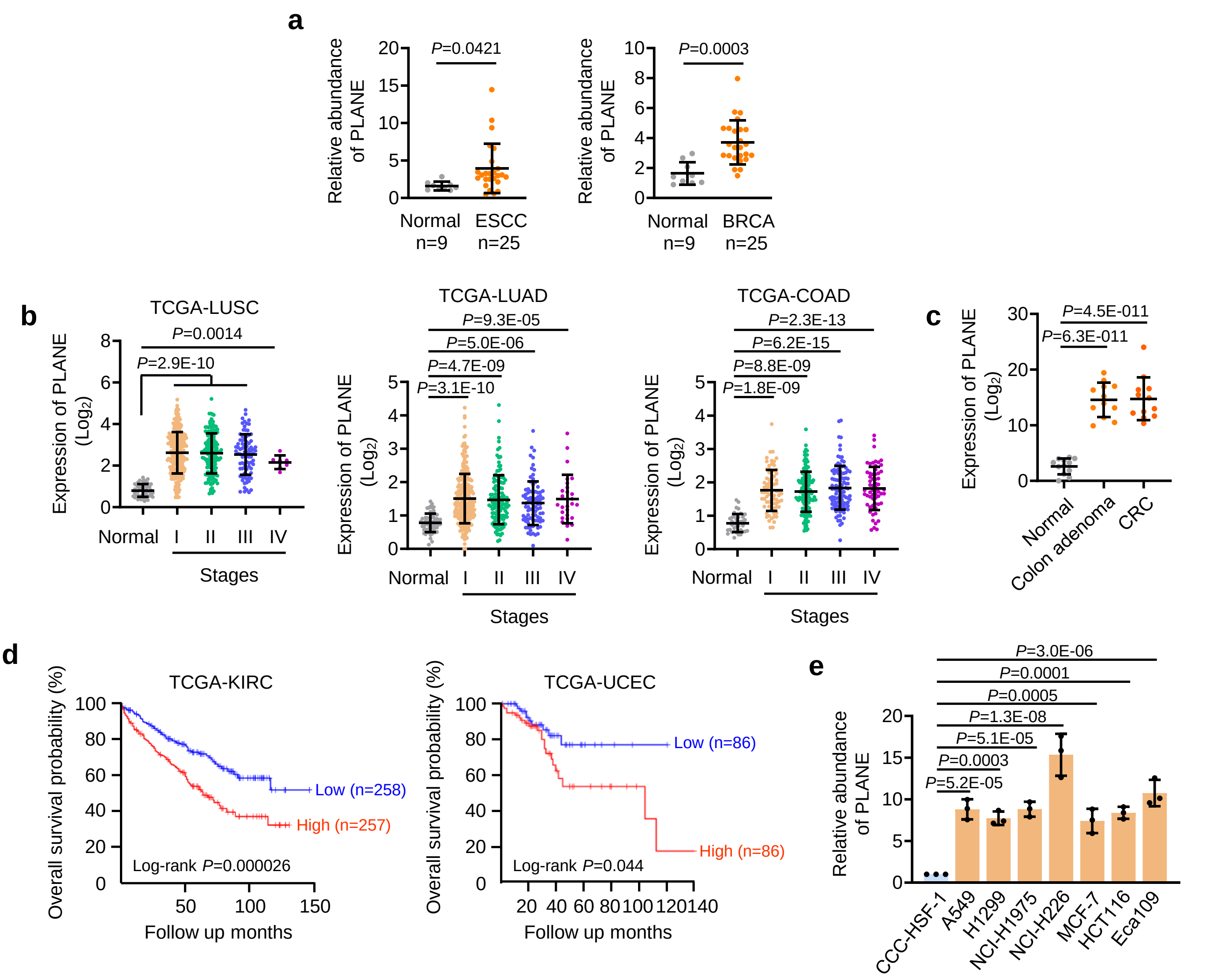

Supplementary Figure 2. PLANE is upregulated at early stages during cancer pathogenesis

a qPCR analysis of PLANE expression from esophageal squamous cell carcinoma (ESCC; n=25) and breast carcinoma (BRCA; n=25) tissues and corresponding adjacent normal tissues. The expression of PLANE in one of corresponding adjacent normal tissues was arbitrarily designated as 1. Data are mean ± s.d.; two-tailed Student's *t*-test.

b Analysis of the TCGA data showing that PLANE expression levels did not differ among tumours of different stages in LUSC, LUAD and COAD. TCGA-LUSC: Normal (n=51); Stage I (n=239); Stage II (n=154); Stage III (n=82); Stage IV (n=7). TCGA-LUAD: Normal (n=59); Stage I (n=273); Stage II (n=124); Stage III (n=84); Stage IV (n=27). TCGA-COAD: Normal (n=39); Stage I (n=74); Stage II (n=146); Stage III (n=104); Stage IV (n=61). Data are mean ± s.d.; one-way ANOVA followed by Tukey's multiple comparisons test.

c PLNAE expression was upregulated in colon adenoma tissues (n = 12) compared with normal colon epithelial tissues (n = 12), whereas there was no significant difference in PLANE expression levels between colon adenoma and colon cancer tissues (n = 12) as determined by qPCR. Data are mean ± s.d.; one-way ANOVA followed by Tukey's multiple comparisons test.

d Kaplan-Meier analysis of the probability of overall survival of kidney clear cell carcinoma (KIRC; n=515) and uterine corpus endometrial carcinoma (UCEC; n=172) patients derived from the TCGA dataset using the median of PLANE levels as the cutoff. The log rank test.

e PLANE was expressed at higher levels in the indicated cancer cell lines than the normal human fibroblast cell line CCC-HSF-1. Data are mean ± s.d.; n = 3 independent experiments, one-way ANOVA followed by Tukey's multiple comparisons test.

Source data are provided as a Source Data file.

Supplementary Figure 3

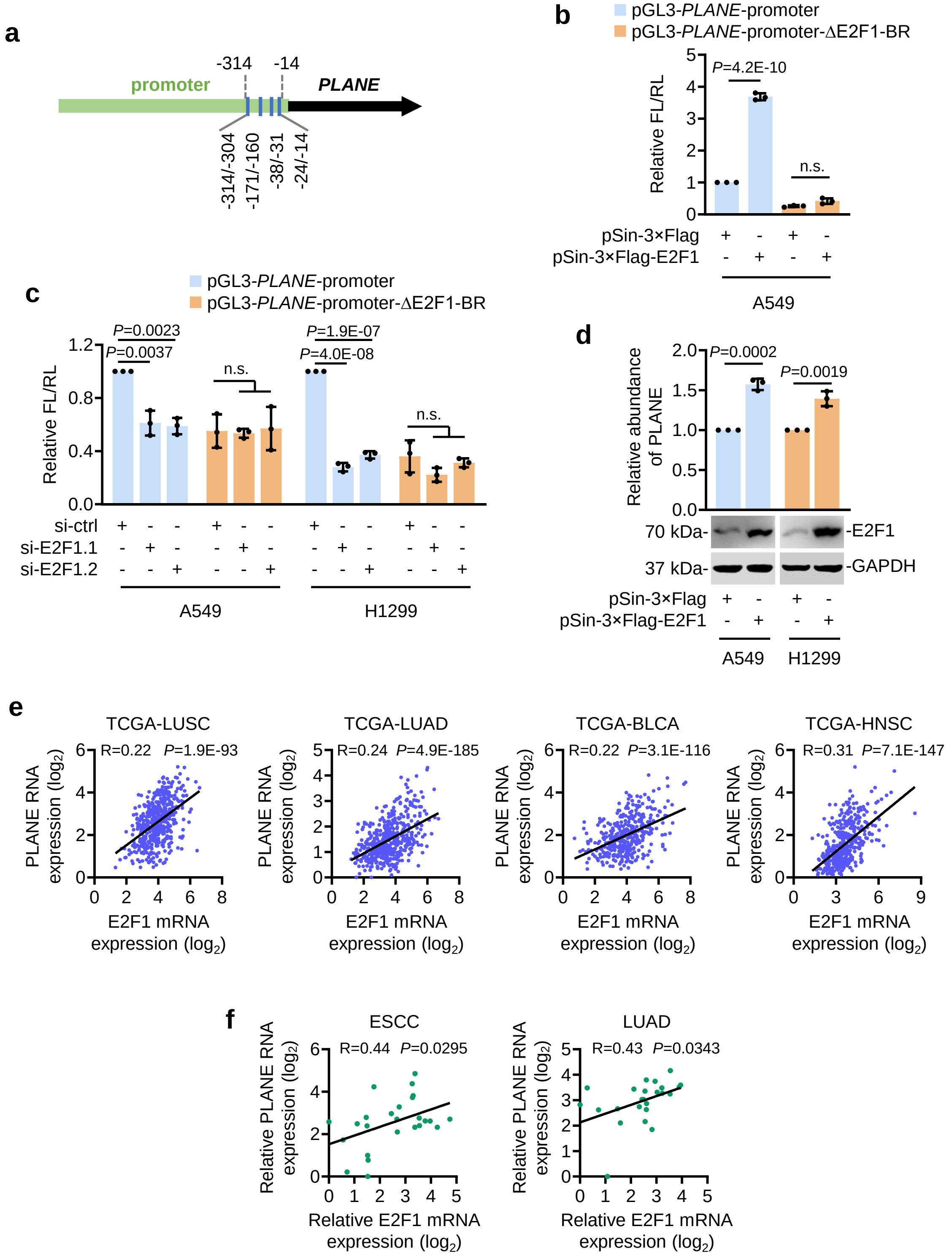

Supplementary Figure 3. E2F1 transcriptionally activates PLANE expression

a Schematic illustration of multiple consensus E2F1 binding motifs (blue bars) located to the -314/-14 region of the proximal promoter of *PLANE* gene.

b Overexpression of E2F1 enhanced the transcriptional activity of a *PLANE* reporter construct with the intact E2F1 binding region (BR) (pGL3-*PLANE*-promoter) but did not affect the activity of a construct with the E2F1-BR deleted (pGL3-*PLANE*-promoter-ΔE2F1-BR) in A549 cells. Data are mean ± s.d.; n = 3 independent experiments, one-way ANOVA followed by Tukey’s multiple comparisons test. FL: Firefly luciferase activity; RL: Renilla luciferase activity.

c SiRNA knockdown of E2F1 reduced the transcriptional activity of a *PLANE* reporter construct with the intact E2F1-BR (pGL3-*PLANE*-promoter) but did not affect the activity of a construct with the E2F1-BR deleted (pGL3-*PLANE*-promoter-ΔE2F1-BR). Data are mean ± s.d.; n = 3 independent experiments, one-way ANOVA followed by Tukey’s multiple comparisons test.

d Overexpression of E2F1 caused upregulation of PLANE in A549 and H1299 cells. Data are mean ± s.d. or representatives; n = 3 independent experiments, two-tailed Student’s *t*-test.

e Linear regression analysis of the relationship between PLANE and E2F1 mRNA expression in the LUSC, LUAD, bladder urothelial carcinoma (BLCA) and head and neck squamous cell carcinoma (HNSC) datasets derived from the TCGA. Two-tailed Student’s *t*-test.

f Linear regression analysis of the relationship between PLANE and E2F1 mRNA expression in the ESCC (n=25) and LUAD (n=24) tissues. The expression of PLANE and E2F1 was measured using qPCR. The lowest expression of PLANE or E2F1 was arbitrarily designated as 1. Two-tailed Student’s *t*-test.

Source data are provided as a Source Data file.

Supplementary Figure 4

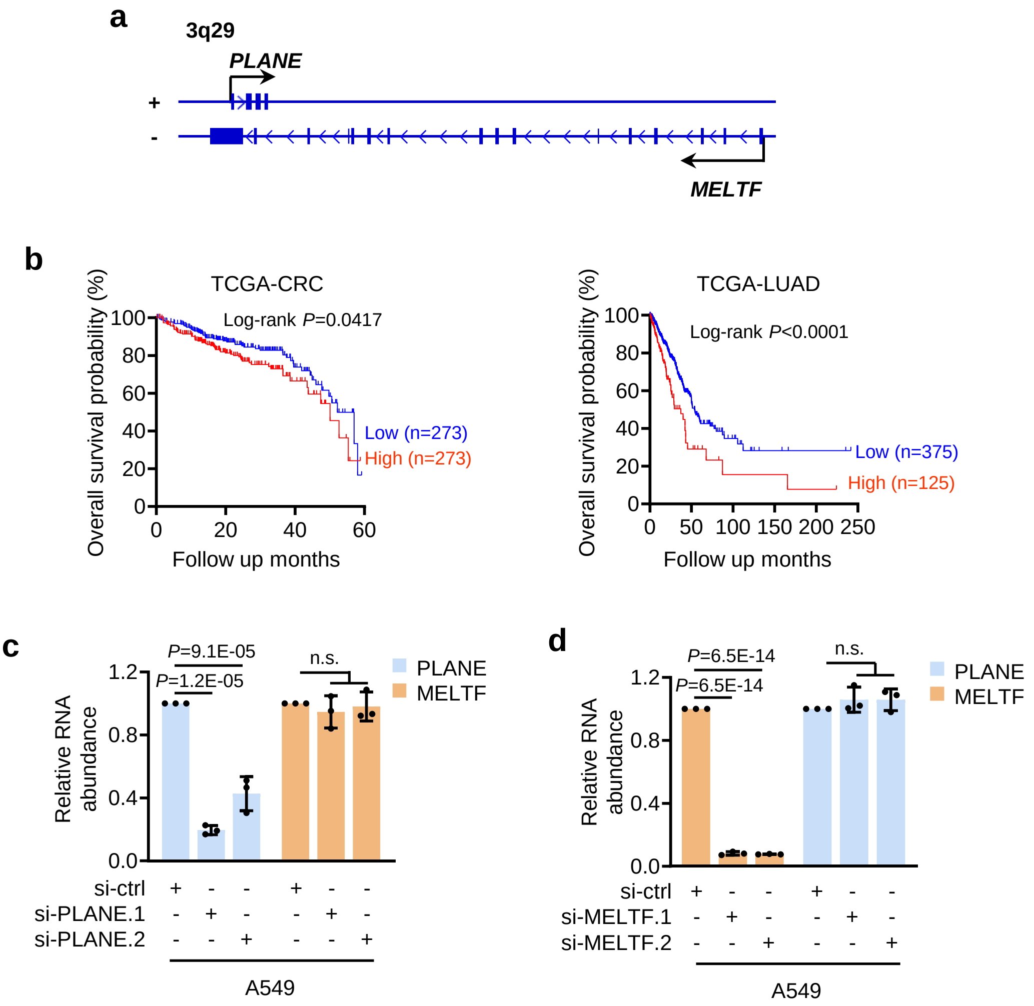

Supplementary Figure 4. PLANE does not affect the expression of its neighbour gene *MELTF*
a Schematic illustration of the genomic location of the *PLANE* and *MELTF* genes.
b Kaplan-Meier analysis of the probability of overall survival of colorectal carcinoma (CRC; n=546) and lung adenocarcinoma (LUAD; n=500) patients derived from the TCGA dataset using the median or quartile of MELTF levels as the cutoff, respectively. The log rank test.
c Knockdown of PLANE did not impinge on MELTF expression in A549 cells. Data are mean ± s.d.; n = 3 independent experiments, one-way ANOVA followed by Tukey's multiple comparisons test.
d Knockdown of MELTF did not impact PLANE expression in A549 cells. Data are mean ± s.d.; n = 3 independent experiments, one-way ANOVA followed by Tukey's multiple comparisons test.
Source data are provided as a Source Data file.

Supplementary Figure 5

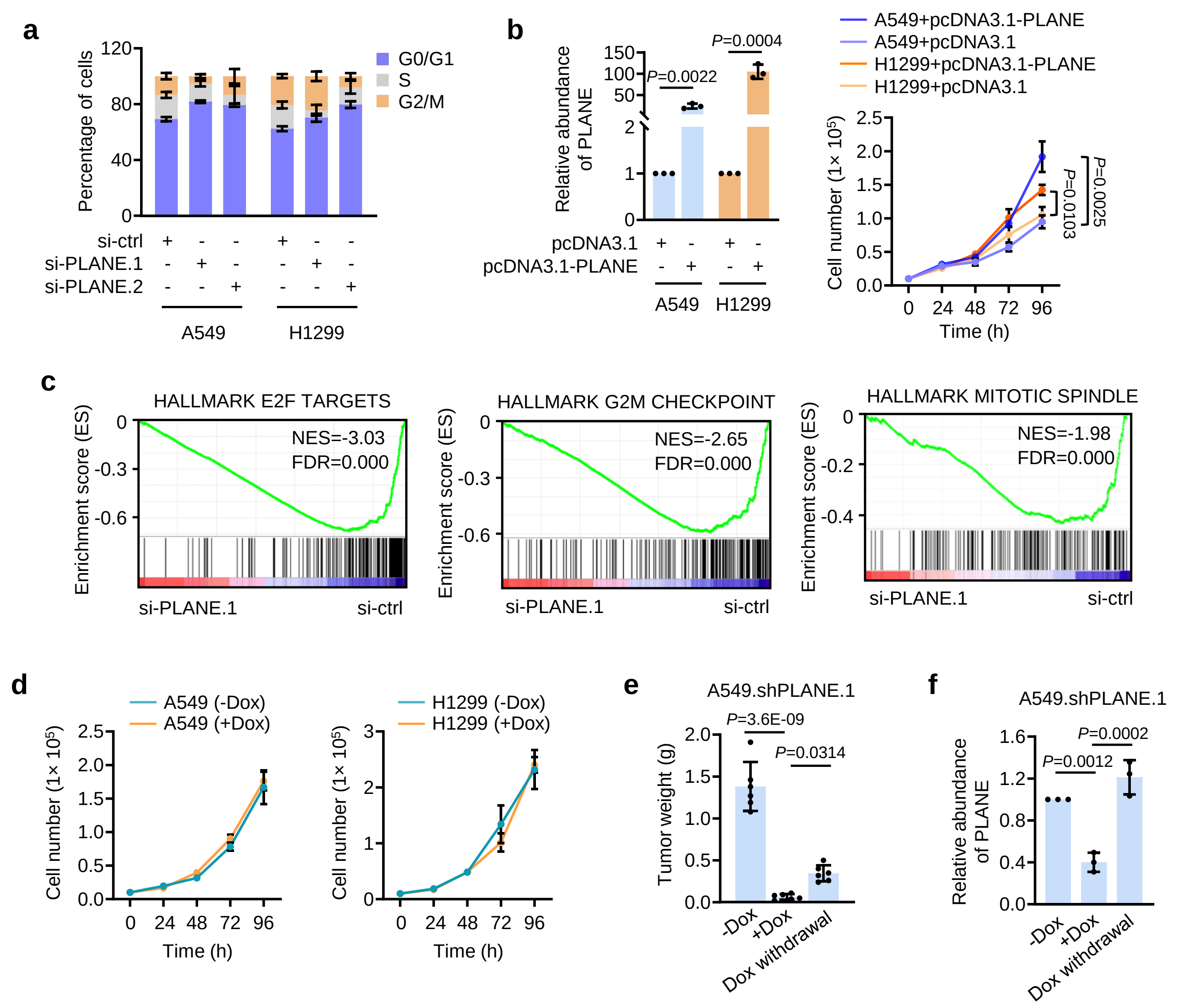

Supplementary Figure 5. PLANE promotes cancer cell proliferation

a SiRNA knockdown of PLANE caused cell cycle arrest at G0/G1 phase in A549 and H1299 cells as shown by propidium iodide staining followed by flow cytometry analysis. Data are mean ± s.d.; n = 3 independent experiments.

b Overexpression of PLANE (left) promoted cell proliferation as shown by accelerated cell number increases (right) in A549 and H1299 cells. Data are mean ± s.d.; n = 3 independent experiments, two-tailed Student's *t*-test.

c GSEA of RNA-seq data from A549 cells. n=2 experimental repeats. NES, normalised enrichment score; FDR, false discovery rate.

d Doxycycline (Dox, 500nM) treatment did not impinge on cell number increases in A549 and H1299 cells. Data are mean ± s.d.; n = 3 independent experiments.

e & f Tumour weights (**e**) (n=6 biologically independent animals) and PLANE expression in representative samples (n=3 biologically independent animals) (**f**) of A549.shPLANE xenografts in nu/nu mice with or without treatment with doxycycline (Dox, 2 mg/ml supplemented with 10 mg/ml sucrose in drinking water) and cessation of Dox treatment. Data are mean ± s.d.; one-way ANOVA followed by Tukey's multiple comparisons test.

Source data are provided as a Source Data file.

Supplementary Figure 6

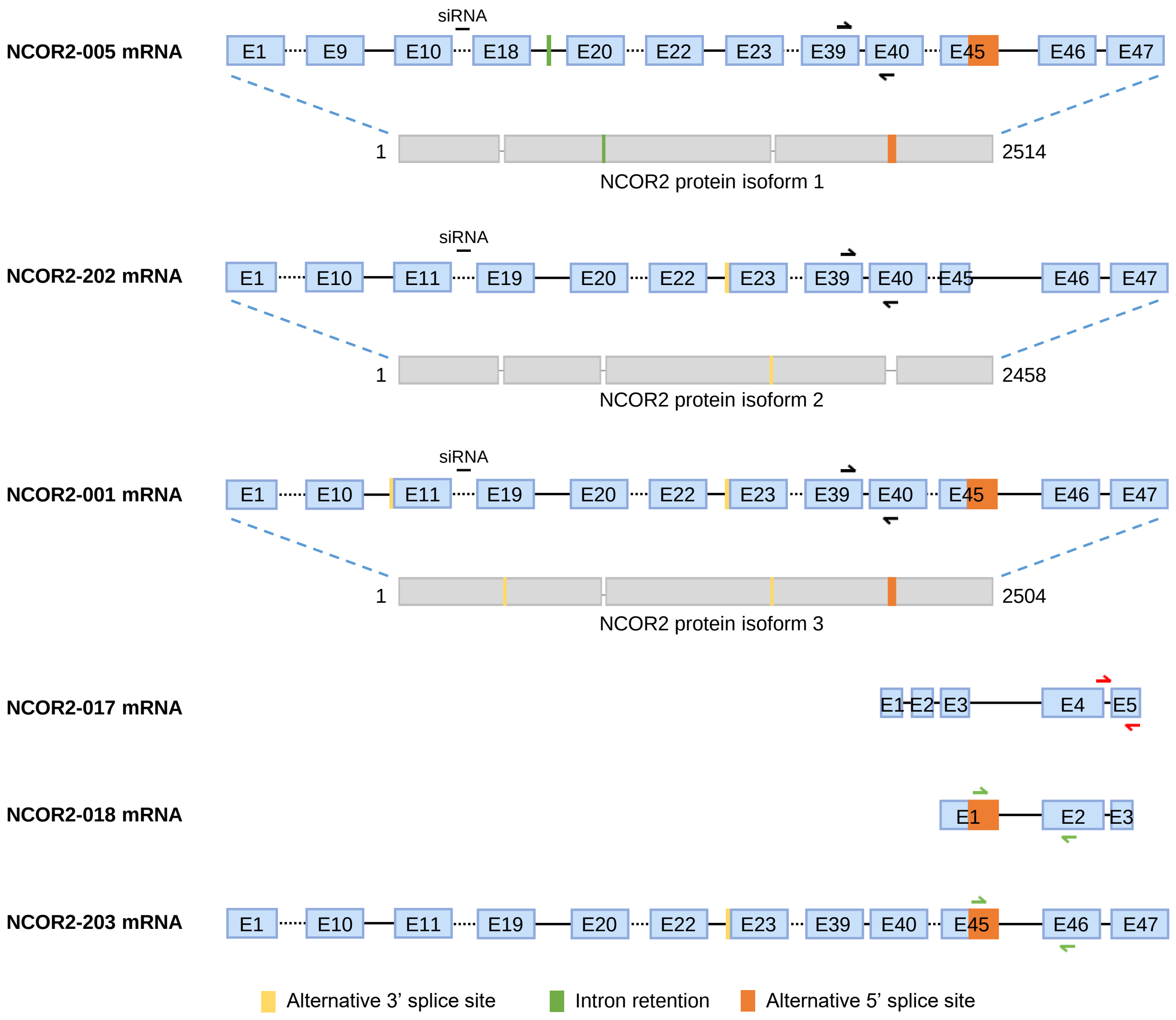

Supplementary Figure 6. Schematic illustration of the NCOR2 mRNA variants and protein isoforms
Schematic illustration of the NCOR2 mRNA variants NCOR2-005, -202, -001, -017, -018 and -203, and NCOR2 protein isoforms NCOR2-1, -2 -3 encoded individually by NCOR2-005, -202 and -001. E: exon. Black arrows indicate primers recognising a common region present in NCOR2-001, -202, -005 as well as -002, -201 and -203. Red arrows indicate primers recognising NCOR2-017. Green arrows indicate primers recognising a common region present in NCOR2-018 and -203.

Supplementary Figure 7

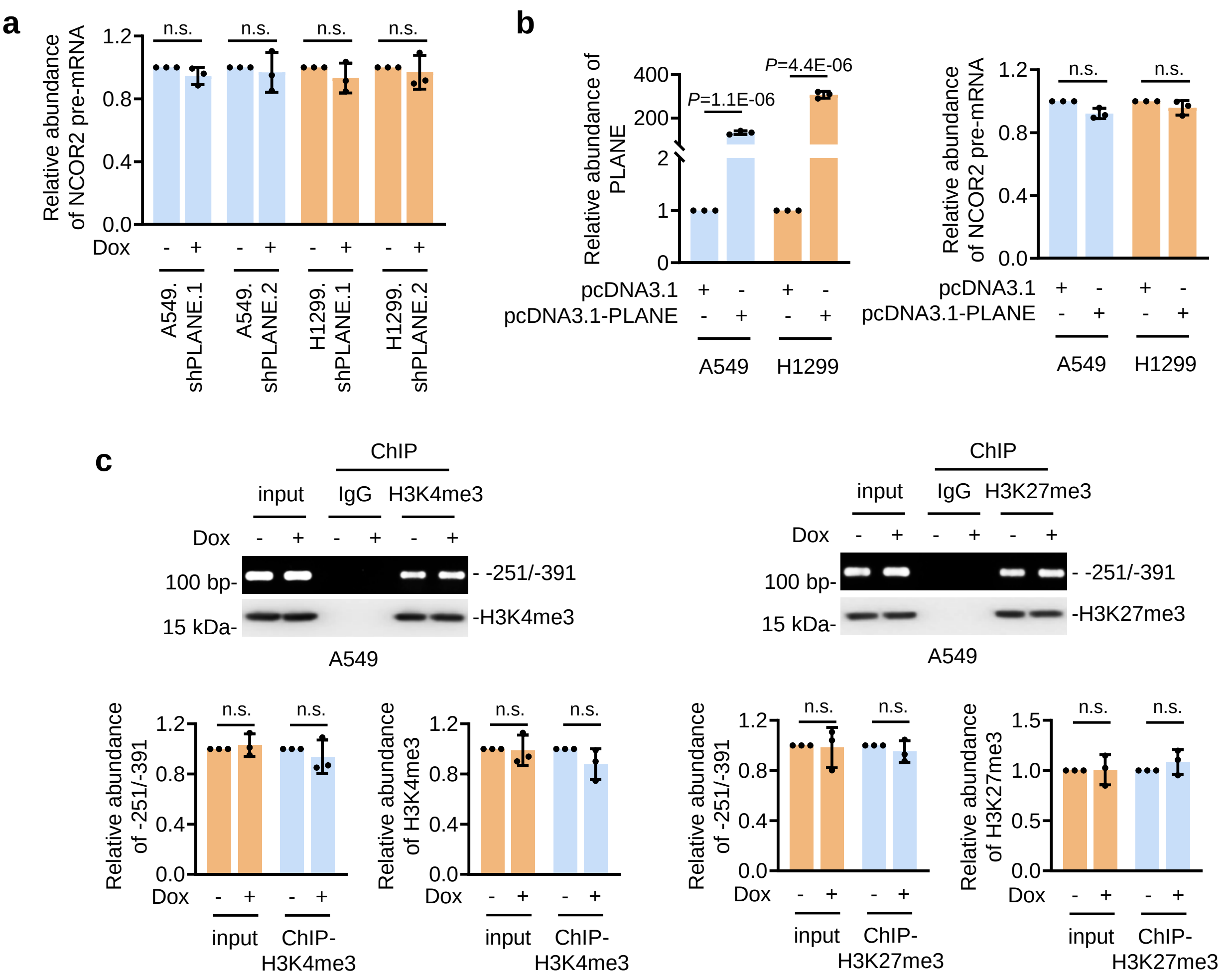

Supplementary Figure 7. PLANE does not regulate NCOR2 transcription

a & b Neither induced knockdown (**a**) nor overexpression (**b**) of PLNAE impinged on NCOR2 pre-mRNA expression. Data are mean ± s.d.; n = 3 independent experiments, two-tailed Student's *t*-test.

c Induced knockdown of PLANE did not affect the enrichment of the transcriptional activation mark H3K4me3 and the transcriptional repression mark H3K27me3 to the *NCOR2* promoter in A549 and H1299 cells as shown using chromatin immunoprecipitation (ChIP) assays. Relative abundance of -251/-391 region within *NCOR2* promoter and relative abundance of H3K4me3 and H3K27me3 protein levels were quantified. Data are representatives or mean ± s.d.; n = 3 independent experiments, two-tailed Student's *t*-test.

Source data are provided as a Source Data file.

Supplementary Figure 8

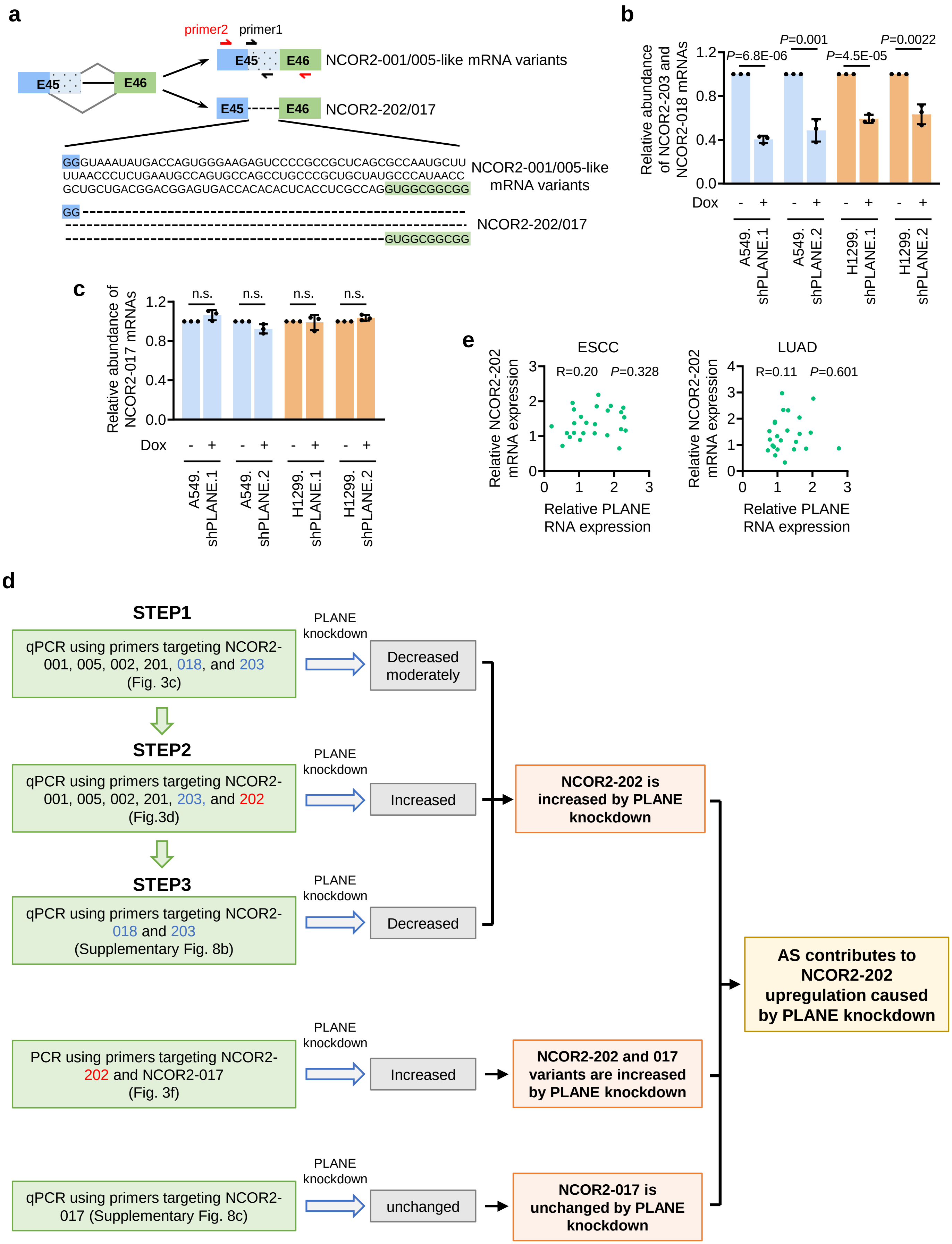

Supplementary Figure 8. PLANE regulates NCOR2 pre-mRNA AS

a Schematic illustration of the sequence overlaps between NCOR2-202/017 and NCOR2-001/005-like mRNA variants and the generation of NCOR2-202 through an alternative 5' splice site. Primer pair 1 recognising the 1-138 fragment of intron 45 that is not contained by NCOR2-202 was used to detect NCOR2-001 and NCOR2-005 as well as NCOR2-002, NCOR2-018, NCOR2-201 and NCOR2-203. Primer pair 2 flanking the splice site at the junction of exon/intron 45 was used to detect the NCOR2-202-generating AS event.

b qPCR analysis showed the levels of NCOR2 AS variants NCOR2-203 and NCOR-018 were reduced in cells with PLANE knocked down. Data are mean ± s.d.; n = 3 independent experiments, two-tailed Student's *t*-test.

c qPCR analysis showed that induced knockdown of PLANE did not affect the expression level of NCOR2 AS variant NCOR2-017. Data are mean ± s.d.; n = 3 independent experiments, two-tailed Student's *t*-test.

d The workflow of identification of the effect of AS on NCOR2-202 upregulation caused by PLANE knockdown.

e Linear regression analysis showed that there was no significant relationship between PLANE and NCOR2-202 expression levels in ESCC (n=25) and LUAD (n=24) tissues. Expression levels of PLANE and NCOR2-202 were quantitated using semi-quantitative RT-PCR. Two-tailed Student's *t*-test.

Source data are provided as a Source Data file.

Supplementary Figure 9

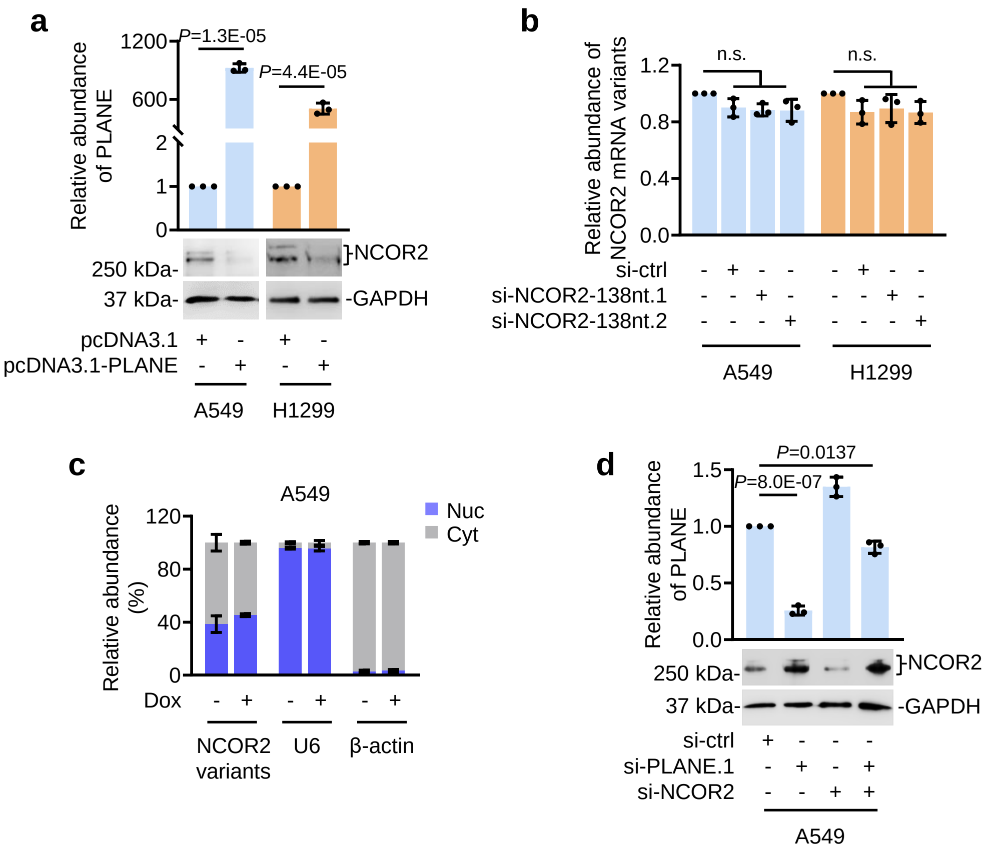

Supplementary Figure 9. PLANE suppresses NCOR2 protein expression

a Overexpression of PLANE caused downregulation of NCOR2 at the protein level in A549 and H1299 cells. Data are representatives or mean ± s.d.; n = 3 independent experiments, two-tailed Student's *t*-test.

b Knockdown of NCOR2 using siRNAs targeting the 1-138 nucleotide (nt) fragment present in NCOR2-001/005-like variants but not NCOR2-202/017 within intron 45 did not reduce the expression levels of NCOR2 mRNA variants including NCOR2-001 and NCOR2-005 as well as NCOR2-002, NCOR2-018, NCOR2-201 and NCOR2-203. Data are mean ± s.d.; n=3 independent experiments, one-way ANOVA followed by Tukey's multiple comparisons test.

c qPCR analysis using primers spanning across a common region present in nine NCOR2 AS variants (NCOR2-202/001/005/017/015/022/018/201/203) showed that PLANE knockdown did not cause any significant changes in the proportions of NCOR2 AS variants distributing to nuclear and cytoplasmic fractions. U6 and β-actin RNA expression was included as controls for nuclear and cytoplasmic fractions, individually. Data are mean ± s.d.; n = 3 independent experiments, two-tailed Student's *t*-test. Cyt: cytoplasm; Nuc: nucleus.

d SiRNA knockdown of PLANE and NCOR2 in A549 cells. Data are mean ± s.d. or representatives; n = 3 independent experiments, one-way ANOVA followed by Tukey's multiple comparisons test.

Source data are provided as a Source Data file.

Supplementary Figure 10

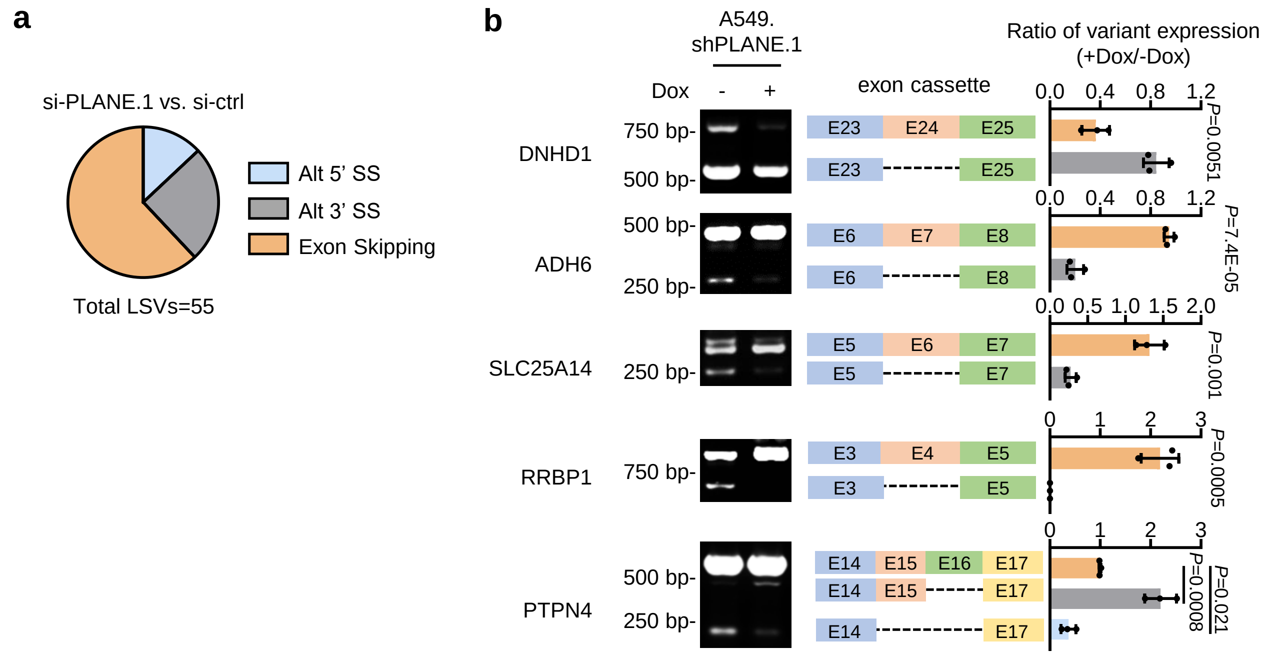

Supplementary Figure 10. PLANE regulates AS of other pre-mRNAs

a MAJIQ analysis of RNA-seq data (two experimental repeats) showing the categorization of alternative splicing events caused by PLANE knockdown in A549 cells. LSVs, local splicing variations; Alt 5' SS, Alternative 5' splicing site; Alt 3' SS, Alternative 3' splicing site. MAJIQ, modelling alternative junction inclusion quantification.

b RT-PCR analysis of the indicated AS events using primers flanking PLANE-regulated alternative exons. Relative levels of relevant AS variants quantitated using densitometry are also shown. Data are representatives or mean ± s.d.; n = 3 independent experiments, two-tailed Student's *t*-test. DNHD1, Dynein Heavy Chain Domain 1; ADH6, Alcohol Dehydrogenase 6; SLC25A14, Solute Carrier Family 25 Member 14; RRBP1, Ribosome Binding Protein 1; PTPN4, Protein Tyrosine Phosphatase Non-Receptor Type 4.

Source data are provided as a Source Data file.

Supplementary Figure 11

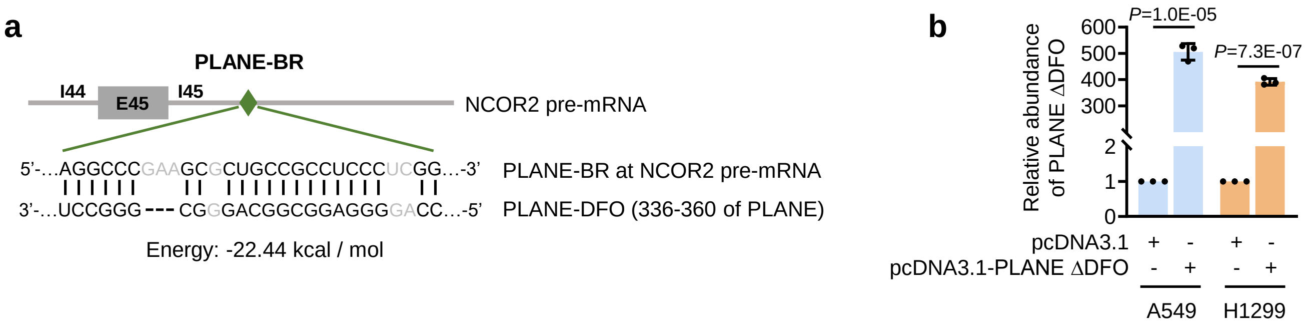

Supplementary Figure 11. PLANE interacts with NCOR2 pre-mRNA

a Schematic illustration of base-pairing between the PLANE binding region (PLANE-BR) at intron 45 (I45) of the NCOR2 pre-mRNA and the duplex-forming oligonucleotides (DFO) of PLANE.

b Overexpression of a PLANE mutant with the DFO deleted (PLANE-ΔDFO) in A549 and H1299 cells. Data are mean ± s.d.; n=3 independent experiments, two-tailed Student's *t*-test.

Source data are provided as a Source Data file.

Supplementary Figure 12

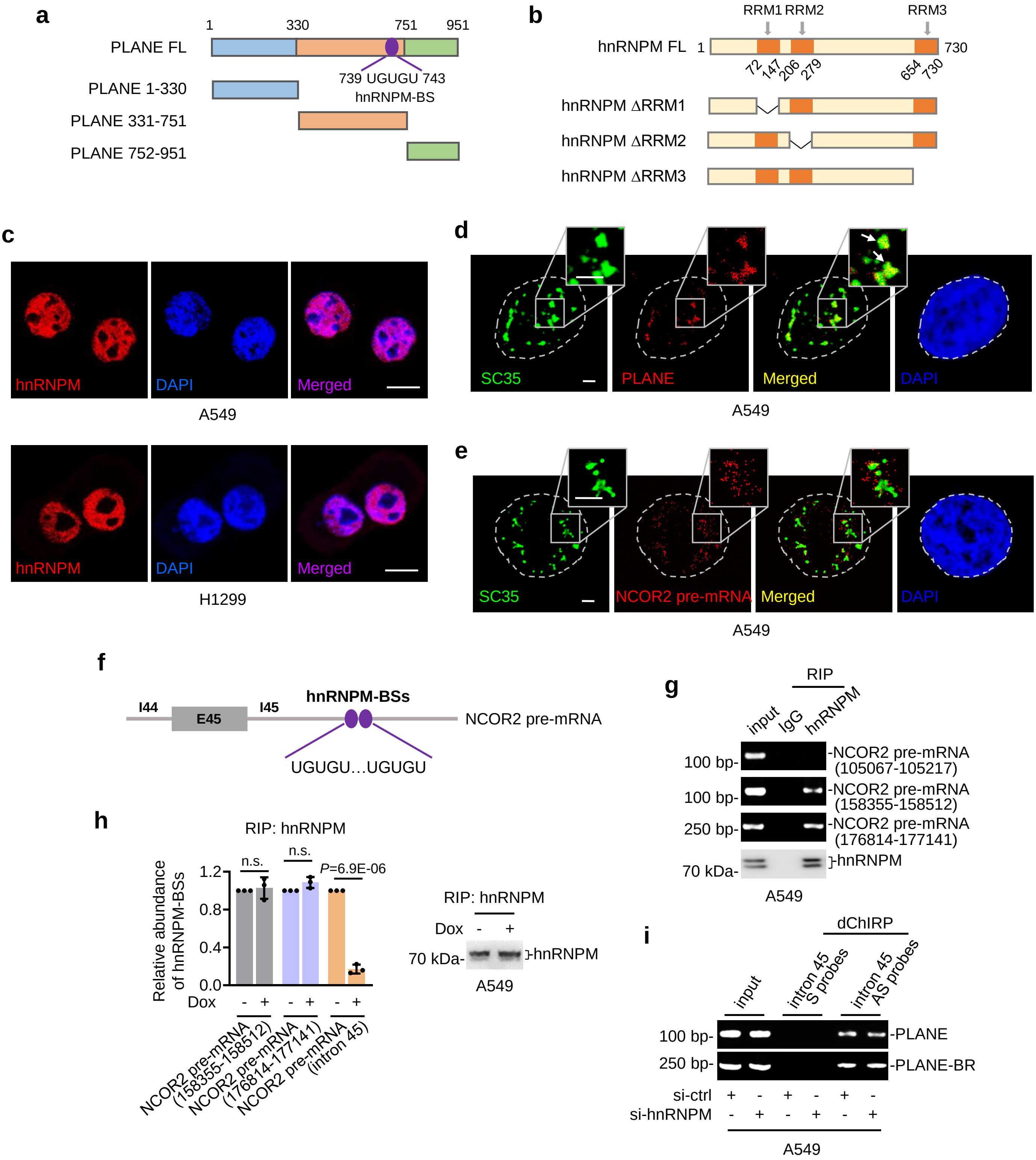

Supplementary Figure 12. PLANE binds to hnRNPM and facilitates repression of the NCOR2-202-generating AS event

a Schematic illustration of full-length PLANE (PLANE-FL) and the PLANE mutants (PLANE 1-330, PLANE 331-751 and PLANE 752-951) used in mapping experiments. The consensus hnRNPM binding site (hnRNPM-BS) within PLANE 331-751 region is also shown.

b Schematic illustration of full-length hnRNPM (hnRNPM FL) and the hnRNPM mutants with individual RNA recognition motifs (RRM1, RRM2 and RRM3) deleted.

c Representative confocal images of immunofluorescence staining of hnRNPM in A549 cells. DAPI was used to decorate nuclear DNA. Data shown are representatives of 3 independent experiments. Scale bar: 10 μm.

d Dual colour confocal images showing that a proportion of SC35 immunostaining co-localises with PLANE within the nucleus of A549 cells. Data shown are representatives of 3 independent experiments. Scale bar: 2 μm

e Dual colour confocal images showing limited co-localisation between SC35 immunostaining and NCOR2 pre-mRNA within the nucleus of A549 cells. Data shown are representatives of 3 independent experiments. Scale bar: 2 μm

f Schematic illustration of the consensus hnRNPM-binding sites (hnRNPM-BSs) at intron 45 of the NCOR2 pre-mRNA.

g RIP assays in A549 cells showing co-precipitation between hnRNPM and NCOR2 pre-mRNA fragments 158355-158512 and 176814-177141 but not 105067-105217. Data shown are representative of 3 independent experiments.

h RIP assay showed that induced knockdown of PLANE decreased the amount of hnRNPM associated with the intron 45 of NCOR2 pre-mRNA but did not affect the association between hnRNPM with fragments 158355-158512 and 176814-177141 in A549 cells. Data are mean ± s.d. or representatives; n = 3 independent experiments, two-tailed Student's *t*-test.

i PLANE was coprecipitated with a fragment at intron 45 of NCOR2 pre-mRNA containing the PLANE-BR in A549 cells with or without siRNA knockdown of hnRNPM as shown using dChIRP assays. Data are representatives of 3 independent experiments.

Source data are provided as a Source Data file.

Supplementary Figure 13

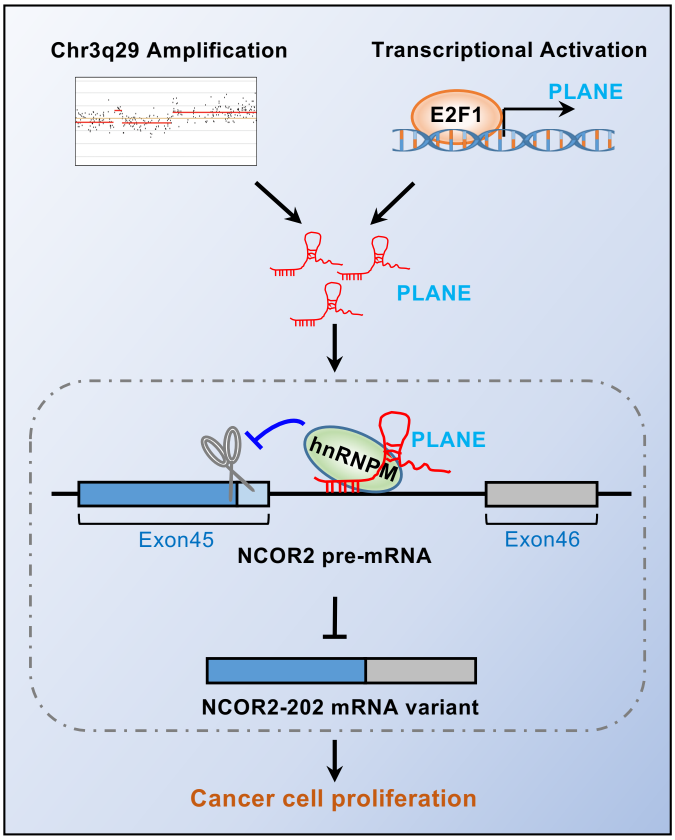

Supplementary Figure 13. Schematic illustration PLANE facilitation of hnRNPM-mediated repression of the NCOR2-202-generating alternative splicing (AS) event to promote tumorigenesis

PLANE is upregulated in diverse cancer types driven by genomic amplification and transcriptional activation by E2F1 and forms RNA-RNA duplex with the NCOR2 pre-mRNA and binds to hnRNPM. This facilitates the association of hnRNPM with the NCOR2 pre-mRNA at intron 45, leading to repression of the NCOR2-202-generating alternative splicing event, leading to promotion of tumorigenesis.

Supplementary Figure 14

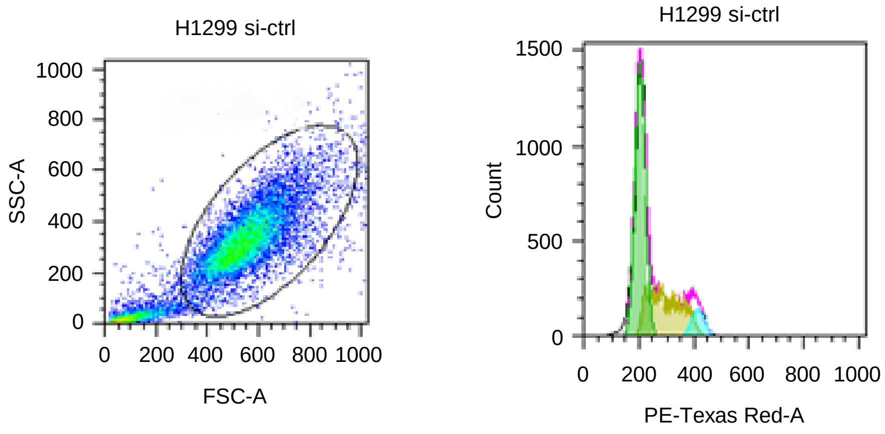

Supplementary Figure 14. Representative FACS sequential gating strategy for cell cycle analysis. (Related to Supplementary Figure 5a). Left panel demonstrates the gating strategy on a SSC vs FSC plot following capture of 10,000 events on a FACSCanto flow cytometer (BD Biosciences). This gate setting used in all subsequent sample runs. Right panel demonstrates typical cell cycle plot obtained using these gate settings.

Supplementary Tables

Supplementary Table 1. Summary of clinicopathological characteristics of the cohort of 75 lung squamous cell carcinoma patients

Characteristics	Cases	PLANE abundance in Lung squamous cell carcinoma(RS[1])	*P* value[2]
**Gender**	75		
Male	69	0.75 ± 0.11[(3)]	0.102
Female	6	0.10 ± 0.15	
**Age**	75		
≥62[(4)]	39	0.87 ± 0.16	0.101
<62	36	0.51 ± 0.12	
**TNM Stage**	75		
I	28	0.73 ± 0.17	0.808
II/III	35	0.67 ± 0.15	
**Histological Grade**	75		
I/II	55	0.65 ± 0.12	0.447
II-III/III	20	0.84 ± 0.23	

[1]RS: Reactive score
[2]Two-tailed Student's *t*-test; a *P* value less than 0.05 was considered statistically significant
[3]Data shown are mean ± s.e.m.
[4]The median age of the patients in this cohort was 62

Supplementary Table 2. Summary of clinicopathological characteristics of the cohort of 92 lung adenocarcinoma patients

Characteristics	Cases	PLANE abundance in Lung squamous cell carcinoma(RS[1])	*P* value[2]
**Gender**	92		
Male	51	0.34 ± 0.08[(3)]	0.325
Female	41	0.48 ± 0.11	
**Age**	92		
≥63[(4)]	48	0.47 ± 0.11	0.339
<63	44	0.34 ± 0.04	
**TNM Stage**	67		
I /II	41	0.55 ± 0.12	0.276
II/III	26	0.35 ± 0.10	
**Histological Grade**	92		
I/II	57	0.33 ± 0.07	0.143
III	35	0.54 ± 0.13	

[1]RS: Reactive score
[2]Two-tailed Student's; a *P* value less than 0.05 was considered statistically significant
[3]Data shown are mean ± s.e.m.
[4]The median age of the patients in this cohort was 63

Supplementary Table 3. Summary of clinicopathological characteristics of the cohort of 89 colon cancer patients

Characteristics	Cases	PLANE abundance in Lung squamous cell carcinoma(RS[1])	*P* value[2]
**Gender**	89		
Male	51	0.43 ± 0.08[(3)]	0.239
Female	38	0.61 ± 0.12	
**Age**	89		
≥68[(4)]	42	0.41 ± 0.09	0.203
<68	47	0.66 ± 0.11	
**TNM Stage**	80		
I/II	43	0.66 ± 0.11	0.054
III/IV	37	0.35 ± 0.10	
**Histological Grade**	89		
I/II	72	0.52 ± 0.07	
II-III/III	17	0.46 ± 0.21	0.750

[1]RS: Reactive score
[2]Two-tailed Student's; a *P* value less than 0.05 was considered statistically significant
[3]Data shown are mean ± s.e.m.
[4]The median age of the patients in this cohort was 68

Supplementary Table 4. List of siRNAs/shRNAs

PLANE	siRNA.1/shRNA.1: GACCCAAAGAGCAAGUCAU<br>siRNA.2/shRNA.2: GCUCAGAAUCACUAGAAUG
NCOR2-138nt	siRNA.1: UGCUGACGGACGGAGUGACCACACA<br>siRNA.2: AAUGCUUUUAACCCUCUGAAUGCCA
NCOR2	siRNA: UGGUGGAGGAUGAGGAGAU
hnRNPM	siRNA.1: GCAUCGGAAUGGGAAACAU<br>siRNA.2: CCAUUUGACUGUUUGCAUU
MELTF	siRNA.1: GGAUGGAGGAGCCAUCUAU<br>siRNA.2: GGGCGAAGUGUACGAUCAA

Supplementary Table 5. Summary of proteins that interact with PLANE detected using mass spectrometry

No.	Entry name	Coverage [%]	MW [kDa]	Score
1	HNRPM	32	77.5	139.38
2	K2C1	24	66	40.71
3	K1C10	22	58.8	30.7
4	K22E	21	65.4	28.63
5	NUCL	11	76.6	19.43
6	F5H5D3	17	57.7	18.9
7	K1C9	15	62	18.85
8	TBA1A	20	50.1	18.12
9	Q5ST81	16	41.7	12.98
10	RFA1	10	68.1	12.31
11	BIP	7	72.3	9.96
12	WDR76	6	69.7	8.81
13	TBB2A	14	49.9	8.8
14	E7EWK3	4	91.4	8.17
15	HNRPK	9	50.9	7.68
16	H0YIN9	16	22	7.62
17	CSTF3	3	82.9	7.53
18	E7EUT5	9	27.9	6.97
19	DDX3X	4	73.2	5.85
20	PLOD1	2	83.5	5.85
21	A6NLN1	7	56.5	5.39
22	PCBP1	10	37.5	5.35
23	IF4A1	8	46.1	4.87
24	TOP2A	2	174.3	4.71
25	E9PKE3	5	68.8	4.65
26	J3KTA4	4	69	4.61
27	YBOX3	5	40.1	4.44
28	D6RBZ0	7	35.7	4.37
29	J3KT29	15	13.2	3.97
30	HNRPF	4	45.6	3.48
31	M0R076	11	13.1	3.4
32	API5	3	59	3.31
33	DDX21	3	87.3	3.25
34	WDR43	2	74.8	3.09
35	H3BSS4	11	19.4	3.01
36	K1C14	3	51.5	2.92
37	M0R1V7	25	7.1	2.91
38	I3L239	7	24.9	2.89
39	F5GYT8	2	64	2.77
40	RBM14	2	69.4	2.59
41	E9PQD7	6	25.2	2.51
42	A2A3R5	8	25	2.44
43	H0Y449	5	42	2.4
44	G3XAC6	3	48	2.39
45	NOG1	2	73.9	2.34
46	TGFB3	3	47.3	2.33

Supplementary Table 5, continued

47	RS8	7	24.2	2.3
48	PURB	4	33.2	2.29
49	D6RG13	8	25.6	2.25
50	A0A087WVQ9	3	47.9	2.21
51	RS23	8	15.8	2.19
52	B5MCT8	6	16.6	2.17
53	ALBU	2	69.3	2.17
54	FILA2	1	247.9	2.14
55	B7ZM68	1	129.4	2.14
56	F8WA26	19	18.5	0
57	BROMI	3	144.7	0
58	AMD	3	108.3	0

Supplementary Table 6. Sequence similarity of transcripts in other species compared with human PLANE

Description	Total score	Query cover	E value
Homo sapiens	1716	100%	0.0
Gorillas	1467	92%	0.0
Mus musculus	122	7%	2e-12

Supplementary Table 7. List of cell lines

Cell lines	Source	Catalogue No.
A549	ATCC	CCL-185
MCF-7	ATCC	HTB-22
HCT116	ATCC	CCL-247
H1299	National Science and Technology Infrastructure (NSTI, Shanghai, China)	SCSP-589
CCC-HSF-1	National Infrastructure of Cell Line Resource (NICR, Beijing, China)	3111C0001CCC000069
CCC-HIE-2	National Infrastructure of Cell Line Resource (NICR, Beijing, China)	3111C0001CCC000178
Eca109	Dr Xiao Ying Liu (Translational Research Institute, Henan Provincial People's Hospital and People's Hospital of Zhengzhou University, Zhengzhou, China)	N/A
NCI-H1975	Prof Xiaoju Zhang (Respiration Department, Henan Provincial People's Hospital, Zhengzhou, China)	N/A
NCI-H226	Prof Xiaoju Zhang (Respiration Department, Henan Provincial People's Hospital, Zhengzhou, China)	N/A

Supplementary Table 8. List of antibodies

Antibody (Ab)	Catalogue No.	Company	Dilution
E2F1 Rabbit mAb	ab179445	Abcam (Cambridge, UK)	1:1000
H3K4me3 Rabbit mAb	ab1012	Abcam (Cambridge, UK)	1:500
H3K27me3 Rabbit mAb	ab192985	Abcam (Cambridge, UK)	1:1000
Normal mouse IgG	sc-2025	Santa Cruz Biotechnology (Dallas, TX)	1: 500
Normal rabbit IgG mAb	ab172730	Abcam (Cambridge, UK)	1:1000
HRP Conjugated AffiniPure Goat Anti-mouse IgG (H+L)	BA1050	BOSTER Biological Technology (Wuhan, Hubei, P.R.C)	1:2000
HRP Conjugated AffiniPure Goat Anti-rabbit IgG (H+L)	BA1054	BOSTER Biological Technology (Wuhan, Hubei, P.R.C)	1:2000
hnRNPM Rabbit pAb	26897-1-AP	Proteintech Group (Wuhan, Hubei, P.R.C)	1:500
SC35 Mouse mAb	ab11826	Abcam (Cambridge, UK)	1:500
CY3 Conjugated AffiniPure Goat Anti-rabbit IgG (H+L)	BA1032	BOSTER Biological Technology (Wuhan, Hubei, P.R.C)	1:200
Chicken anti-Mouse IgG (H+L) Cross-Adsorbed Secondary Antibody, Alexa Fluor 488	A21200	Thermo Fisher Scientific (Waltham, MA)	1:1000
U1-70K Rabbit pAb	ab51266	Abcam (Cambridge, UK)	1:2000
hnRNPK Rabbit mAb	ab52600	Abcam (Cambridge, UK)	1:10000
NCOR2 Rabbit mAb	#62370	Cell Signaling Technology (Danvers, MA))	1:1000

mAb: monoclonal antibody
pAb: polyclonal antibody

Supplementary Table 9. List of reagents

Reagent	Catalogue No.	Company
Doxycycline	D9891	Sigma-Aldrich (Saint Louis, MO)
Piece^{TM} RIPA Buffer	89900	Thermo Fisher Scientific (Waltham, MA)
Piece® IP Lysis Buffer	87787	Thermo Fisher Scientific (Waltham, MA)
BrdU Cell Proliferation Assay Kit	6813	Cell Signaling Technology (Danvers, MA)
Protease Inhibitor Cocktail	HY-K0010	Med Chem Express (Monmouth Junction, NJ)
RiboLock RNase Inhibitor	EO0384	Thermo Fisher Scientific (Waltham, MA)
4% paraformaldehyde	AR1068	BOSTER Biological Technology (Wuhan, Hubei, P.R.C)
Opti-MEM^{TM} Reduced Serum Medium	31985070	Thermo Fisher Scientific (Waltham, MA)
DNA extraction buffer	P1012	Solarbio Life Sciences (Beijing, P.R.C)
Protease K	P1120	Solarbio Life Sciences (Beijing, P.R.C)
Anti-GFP mAb-Magnetic Agarose	D153-10	Medical& Biological Laboratories (Sakae, Nagoya, Aichi,Japan)
TRIzol^{TM} Reagent	15596018	Thermo Fisher Scientific (Waltham, MA)

Supplementary Table 10. List of primers used for recombinant plasmid

pcDNA3.1(+)-PLANE	Forward: CGGGATCCGCTGTCCCACGCGCCGGGT<br>Reverse: AACCTCGAGTGCATGTTCTCCAGATGTCCT
pcDNA3.1(+)-PLANE-R	Forward1: CGGGATCCGCTGTCCCACGCGCCGGGT<br>Reverse1: CTGAAATGACGGTCTCGTTCTATC<br>Forward2: GATAGAACGAGACCGTCATTTCAG<br>Reverse2: AACCTCGAGTGCATGTTCTCCAGATGTCCT
pcDNA3.1(+)-PLANE-△DFO	Forward1: GGATCCGCTGTCCCACGCGCCGGGT<br>Reverse1: GCGCAGGTAGGACTGCTATTCAGAC<br>Forward2: CTGAATAGCAGTCCTACCTGCGCCC<br>Reverse2: CTCGAGTGCATGTTCTCCAGATGTCCT
pcDNA3.1(+)-PLANE-R-△331-751	Forward1: GGGATCCGCTGTCCCACGCGCCGGGT<br>Reverse1: TGATATTCTGTGACTGGTTCAGACCCCTTCA<br>Forward2: GAAGGGGTCTGAACCAGTCACAGAATATCAG<br>Reverse2: CCTCGAGTGCATGTTCTCCAGATGTCCT
pEGFP-C1-hnRNPM	Forward: TAGGTACCATGGCGGCAGGGGTCGAAG<br>Reverse: CGGGATCCTTAAGCGTTTCTATCAATTCGAAC
pEGFP-C1-hnRNPM-△RRM1	Forward1: TAGGTACCATGGCGGCAGGGGTCGAAG<br>Reverse1: TGTTCACCATCAGGCTGTATCTTTTAGT<br>Forward2: ACTAAAAGATACAGCCTGATGGTGAACA<br>Reverse2: CGGGATCCTTAAGCGTTTCTATCAATTCGAAC
pEGFP-C1-hnRNPM-△RRM2	Forward1: TAGGTACCATGGCGGCAGGGGTCGAAG<br>Reverse1: TGGTAAGGCCCTCTCACTGTGCTTCCAAGTC<br>Forward2: GACTTGGAAGCACAGTGAGAGGGCCTTACCA<br>Reverse2: CGGGATCCTTAAGCGTTTCTATCAATTCGAAC
pEGFP-C1-hnRNPM-△RRM3	Forward: TAGGTACCATGGCGGCAGGGGTCGAAG<br>Reverse: CGGGATCCTGGCAGGCCTTCCTGGCCA

Supplementary Table 11. List of qRT-PCR primers

PLANE	Forward: TACATACAGTGACCCAAAGAGCA<br>Reverse: CAGTGCTTCTGAACGCCTCTT
E2F1	Forward: CACTTTCGGCCCTTTTGCTC<br>Reverse: GATTCCCCAGGCTCACCAAA
NCOR2 pre-mRNA	Forward: GGCTTCTTGGCCCATCT<br>Reverse: AAGGCTCCCTGACTCCC
NCOR2-001/005-like variants	Forward: ATGACCAGTGGGAAGAGTCCCCG<br>Reverse: TGGTCACTCCGTCCGTCAGCA
NCOR2-001/005/202/002/201/203	Forward: AAGAGCTCGACAAGAGCCAC<br>Reverse: CTGGTGACCTTTGACCCCTG
NCOR2-018/203	Forward: TGCTGACGGACGGAGTGACC<br>Reverse: TCCGGCGGTTGCAGTCTC
NCOR2-017	Forward: GCACCAGGTTCCACGCCATT<br>Reverse: CCCGCCCTGTTCTGAGTCACT
NCOR2 mRNA nuclear export	Forward: GGGCCTGGCATCTGGGGA<br>Reverse: GACGAGGGCCTGTCCTCCCA
GAPDH	Forward: GCTCTCTGCTCCTCCTGTTC<br>Reverse: ACGACCAAATCCGTTGACTC
β-actin	Forward: GGACTTCGAGCAAGAGATGG<br>Reverse: AGCACTGTGTTGGCGTACAG
U6	Forward: TCGCTTCGGCAGCACATAT<br>Reverse: ATTTGCGTGTCATCCTTGC
18S rRNA	Forward: GCTTAATTTGACTCAACACGGGA<br>Reverse: AGCTATCAATCTGTCAATCCTGTC
MELTF	Forward: GAGCCCCCTGGAGAGATACT<br>Reverse: CATCGTCCTACGTGCTTCCT

Supplementary Table 12. List of RT-PCR primers and RNA pulldown probes

ChIP-E2F1-BR	Forward: AGGGGGGCAGGGCTAGTAG<br>Reverse: ACCTAGATCCTGCCTCCC
NCOR2-202/017-generating AS event	Forward: GAACATGCCAGCACCAACA<br>Reverse: GGAATGGCGTGGAACCTG
ChIP-H3K4me3-BS/-H3K27me3-BS	Forward: AACGCGATTCCAGTGAGGT<br>Reverse: GCGACGCCGAGTTTCTTT
MELTF-AS1-201	Forward: CTGCTGAGACGACATCCCTT<br>Reverse: ACGCCCAGCTCACCTGAT
MELTF-AS1-202 (PLANE): primer1	Forward: TACATACAGTGACCCAAAGAGCA<br>Reverse: CAGTGCTTCTGAACGCCTCTT
MELTF-AS1-202 (PLANE): primer2	Forward: TGAGACGACATCCCTTCC<br>Reverse: TGCTCTTTGGGTCACTGTAT
MELTF-AS1-203	Forward: GCAACCCACGCTTCGAG<br>Reverse: TCCTGCCTCCCAAGGTG
MELTF-AS1-204	Forward: CTCCCCACAAACCTAAACA<br>Reverse: GTAGCCACAGAACGGTCAT
MELTF-AS1-205	Forward: GCGGCGCCTCAGATGC<br>Reverse: GAGTGTGAACGCTCAAAACGG
dChIRP-PLANE	Forward: CTGGACTGCTGCGAACG<br>Reverse: TGGCTGGGGCTGGACTA
dChIRP-intron 45	Forward: CTTGTGACTTTATTTTTGTGCGTGT<br>Reverse: CAAGGAGACAGATGGGCCAAG
dChIRP-intron 47	Forward: CCATCACTCGCAGGACCAAG<br>Reverse: TGTAAATGACAGAATACCACCACC
PLANE-BR/*NCOR2* gene	Forward: CTTGTGACTTTATTTTTGTGCGTGT<br>Reverse: CAAGGAGACAGATGGGCCAAG
RRBP1	Forward: AGGTCCTGGAGGTGCCATTTC<br>Reverse: GCCAGGTGTCCTGAATGATGC
DNHD1	Forward: GCCCATCTTTGATACCTTC<br>Reverse: CTAACCAGACCTCGTGCC
ADH6	Forward: TGTAAAGCAGCAGGAGCA<br>Reverse: CAGCAACCAGTTTAGGGA
SLC25A4	Forward: GCTCCTGCGTTGCTAAGACA<br>Reverse: ACAGGCATGAGCCACAGCA
PTPN4	Forward: AGTAAGCCCTTGGCACGGA<br>Reverse: ACATTGAATCCAAACCTCCC
hnRNPM-BS (intron 45)	Forward: GGCTTCTTGGCCCATCT<br>Reverse: AAGGCTCCCTGACTCCC
lncCyt b	Forward: TTGTTTGATCCCGTTTCGTG<br>Reverse: ACATCGGCATTATCCTCCTG
hnRNPM-BS (105067-105217)	Forward: GCTGTGAGGACTGGGTG<br>Reverse: TGGCACATGGGAAGAAG
hnRNPM-BS (158355-158512)	Forward: GGCGTAAAGCCATCGTG<br>Reverse: AGCCCGTGTTCAGTTCC
hnRNPM-BS (176814-177141)	Forward: TCTCGGTAATGCCCTTTG<br>Reverse: AAGCCCAGACCCTCCAA
PLANE-biotin-probes	AS1: ATGACTTGCTCTTTGGGTC<br>AS2: CATTCTAGTGATTCTGAGC<br>S1: GACCCAAAGAGCAAGTCAT<br>S2: GCTCAGAATCACTAGAATG

**Supplementary Table 12, continued**	
NCOR2 pre-mRNA-biotin-probes	AS1: TTGGACAACTGCAACTCTC<br>AS2: GTCTGCTGTTTGCAATAGC<br>AS3: TAAACTGTCTGCTGTTTGC<br>S1: GAGAGTTGCAGTTGTCCAA<br>S2: GCTATTGCAAACAGCAGAC<br>S3: GCAAACAGCAGACAGTTTA
PLANE-Cy3-probes	AS1: AAAGCAATTTGCAGCTAGC<br>AS2: ATGACTTGCTCTTTGGGTC<br>AS3: CATTCTAGTGATTCTGAGC
NCOR2 pre-mRNA-Cy3-probes	AS1: TTGGACAACTGCAACTCTC<br>AS2: GTCTGCTGTTTGCAATAGC<br>AS3: TAAACTGTCTGCTGTTTGC
PLANE (*in vitro* transcription)	Forward:<br>TAATACGACTCACTATAGGGGCTGTCCCACGCGC<br>CGGGT<br>Reverse:<br>TGCATGTTCTCCAGATGTCCTTTAT
AS PLANE (*in vitro* transcription)	Forward:<br>TAATACGACTCACTATAGGGTGCATGTTCTCCAGA<br>TGTCCTTT<br>Reverse: GCTGTCCCACGCGCCGGGTCCC
PLANE ∆DFO (*in vitro* transcription)	Forward1:<br>TAATACGACTCACTATAGGGGCTGTCCCACGCGC<br>CGGGT<br>Reverse1:<br>GCGCAGGTAGGACTGCTATTCAGAC<br>Forward2: CTGAATAGCAGTCCTACCTGCGCCC<br>Reverse2: TGCATGTTCTCCAGATGTCCTTTAT
PLANE 1-330 (i*n vitro* transcription)	Forward:<br>TAATACGACTCACTATAGGGGCTGTCCCACGCGC<br>CGGGT<br>Reverse: TTCAGACCCCTTCACCCCAGAAC
PLANE 331-751 (*in vitro* transcription)	Forward:<br>TAATACGACTCACTATAGGGGTTCTGGGGTGAAG<br>GGGTCTG<br>Reverse: GTTGCAGAACACAAGTCCCTCTCGA
PLANE 752-951 (*in vitro* transcription)	Forward:<br>TAATACGACTCACTATAGGGCAGTCACAGAATATC<br>AGGTGAGC<br>Reverse: TGCATGTTCTCCAGATGTCCTTTAT
NCOR2 intron45 (*in vitro* transcription)	Forward:<br>TAATACGACTCACTATAGGGTCTGTCTGTCTGTCT<br>CTCTCTC<br>Reverse: CTGCAGGGGGACAAGATGGG
NCOR2 intron47 (*in vitro* transcription)	Forward:<br>TAATACGACTCACTATAGGGTCAGGTCCCAGCGA<br>GCCA<br>Reverse: GGAGTATAATTCGCTTTTTAATTAG

Supplementary Table 12, continued

NCOR2-intron 45-∆PLANE-BR (*In vitro* transcription)	Forward1: TAATACGACTCACTATAGGGTCTGTCTGTCTGTCTCTCTCTC<br>Reverse1: GGCGGACAGCAGTGTGAGTGTGGGCAGGAGGGC<br>Forward2: GCCCTCCTGCCC ACACTCACACTGCTGTCCGCC<br>Reverse2: CTGCAGGGGGACAAGATGGG
