# Supplementary material for: The pan-cancer lncRNA PLANE regulates an alternative splicing program to promote cancer pathogenesis
Source: Nat Commun. 2021 Jun 18;12:3734. doi: 10.1038/s41467-021-24099-4 (PMC8213729; doi:10.1038/s41467-021-24099-4)
Supplement: Supplementary file 3 — Description of Additional Supplementary Files [file 41467_2021_24099_MOESM3_ESM.pdf]

Description of Additional Supplementary Files

File Name: Supplementary Data 1
Description: The short-read RNA sequencing showing transcript expression levels and the list of differentially expressed transcripts with or without PLANE knockdown in A549 cells. FPKM represented Fragments Per Kilobase of transcript per Million mapped reads. Genes with absolute Log2 Foldchange of >1 and *P* value of <0.05 were regarded as significantly differentially expressed.

File Name: Supplementary Data 2
Description: Quantitative visualisation of AS frequency through the whole *NCOR2* gene in representative samples was computed by integrated genome viewer (IGV).

File Name: Supplementary Data 3
Description: The hnRNP M binding sites were identified throughout the whole NCOR2 transcript.
