# Supplementary figures and images for: The pan-cancer lncRNA PLANE regulates an alternative splicing program to promote cancer pathogenesis
Source: Nat Commun. 2021 Jun 18;12:3734. doi: 10.1038/s41467-021-24099-4 (PMC8213729; doi:10.1038/s41467-021-24099-4)

## Slide 1
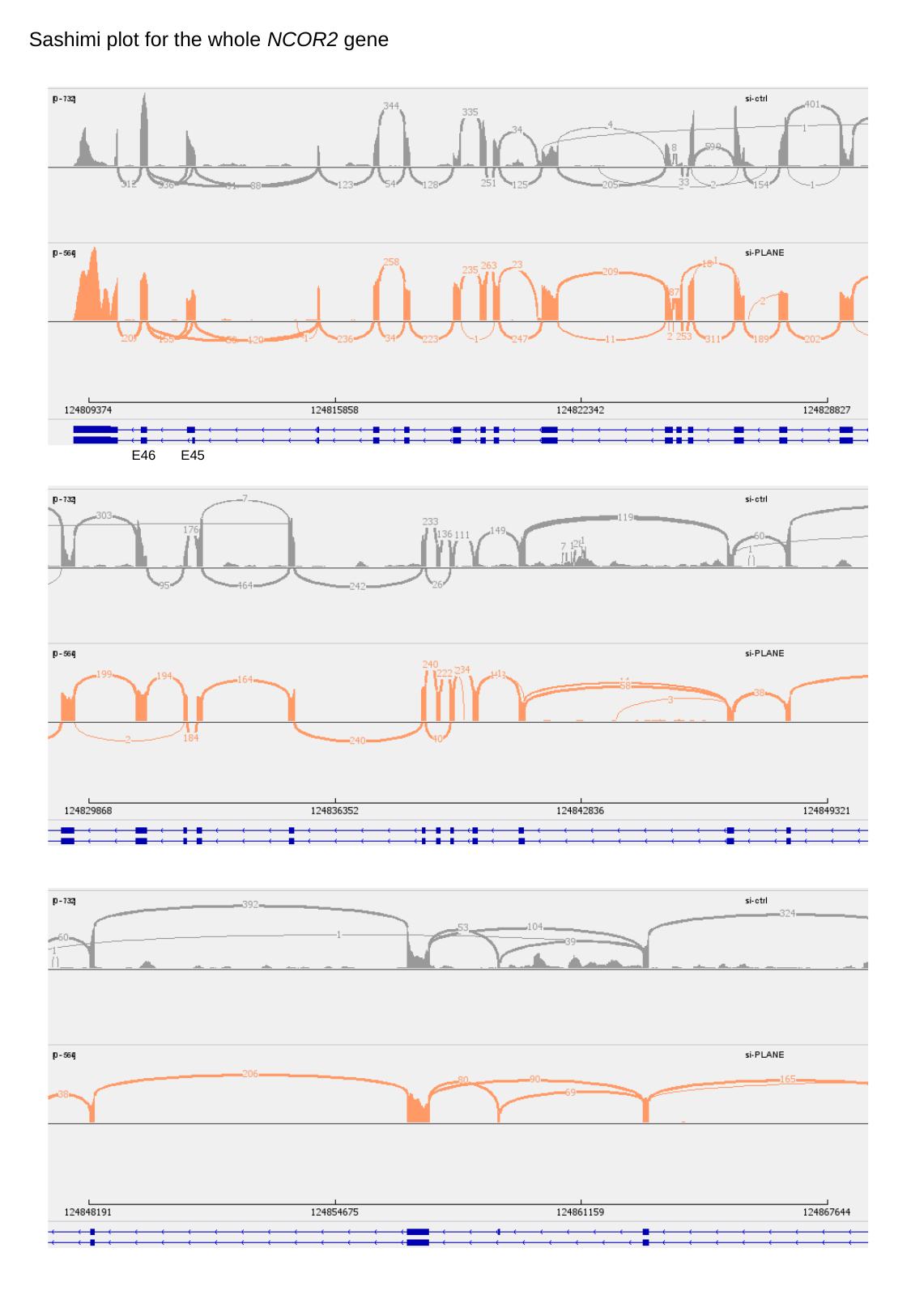

Sashimi plot for the whole NCOR2 gene
E46
E45

## Slide 2
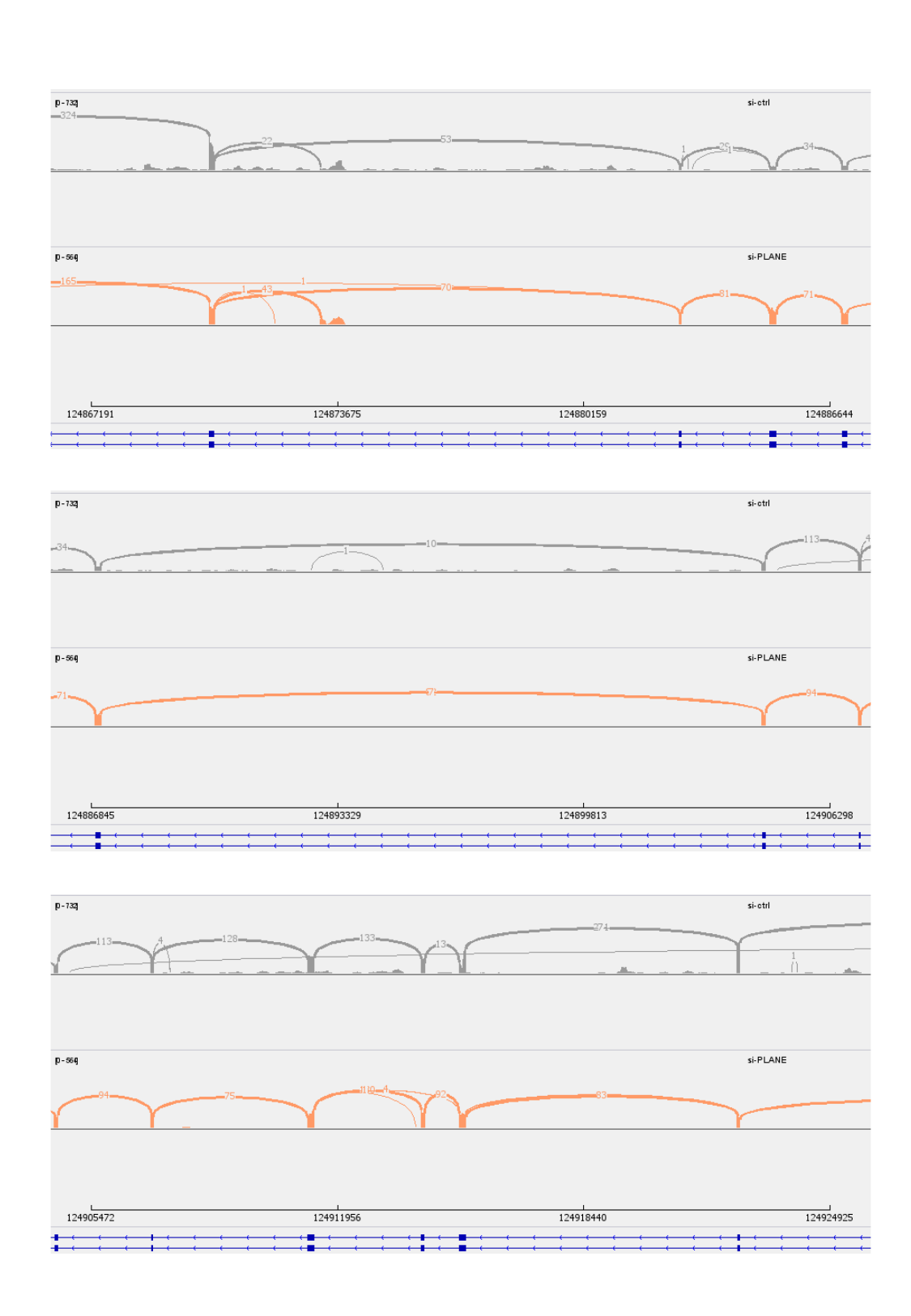

## Slide 3
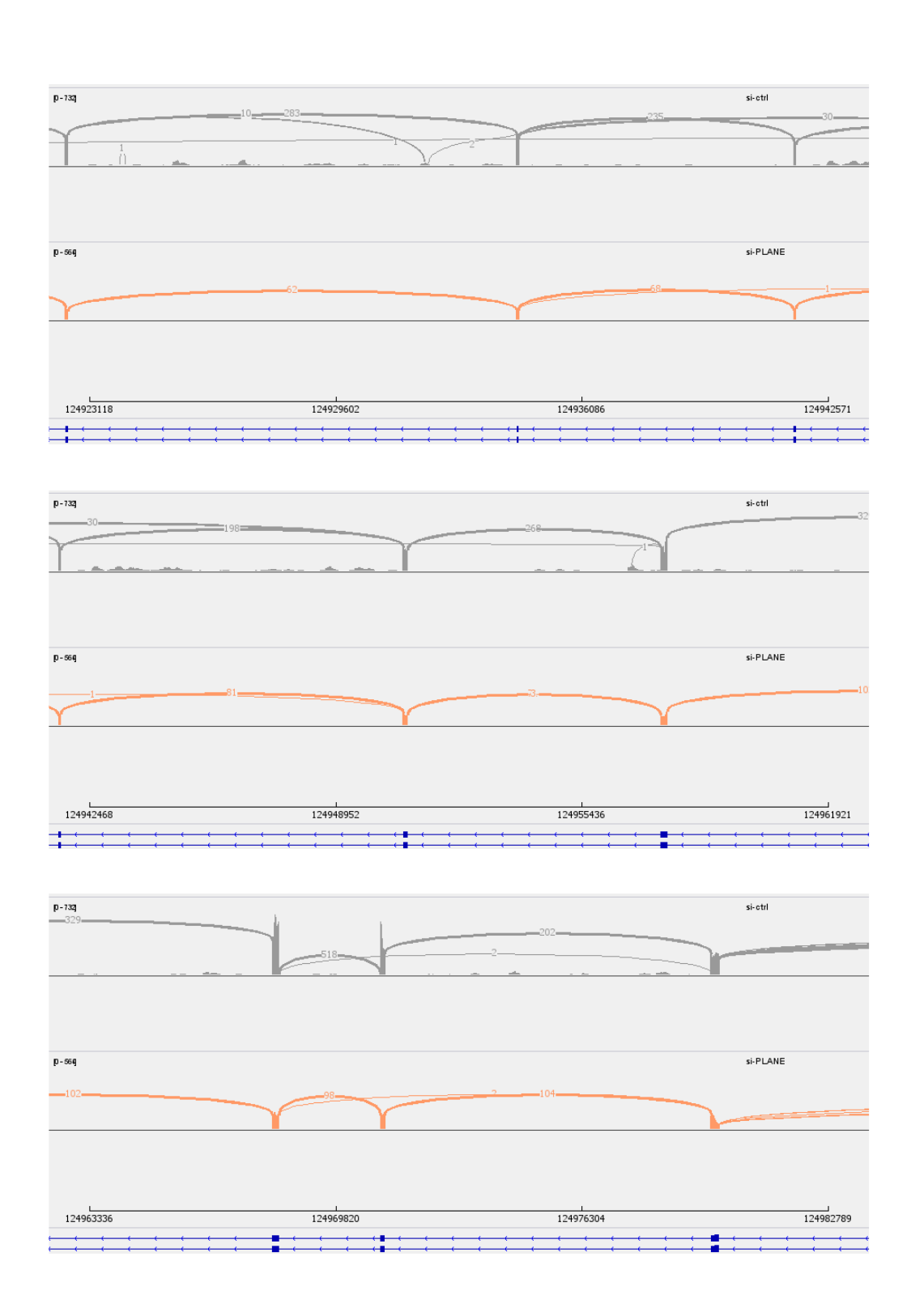

## Slide 4
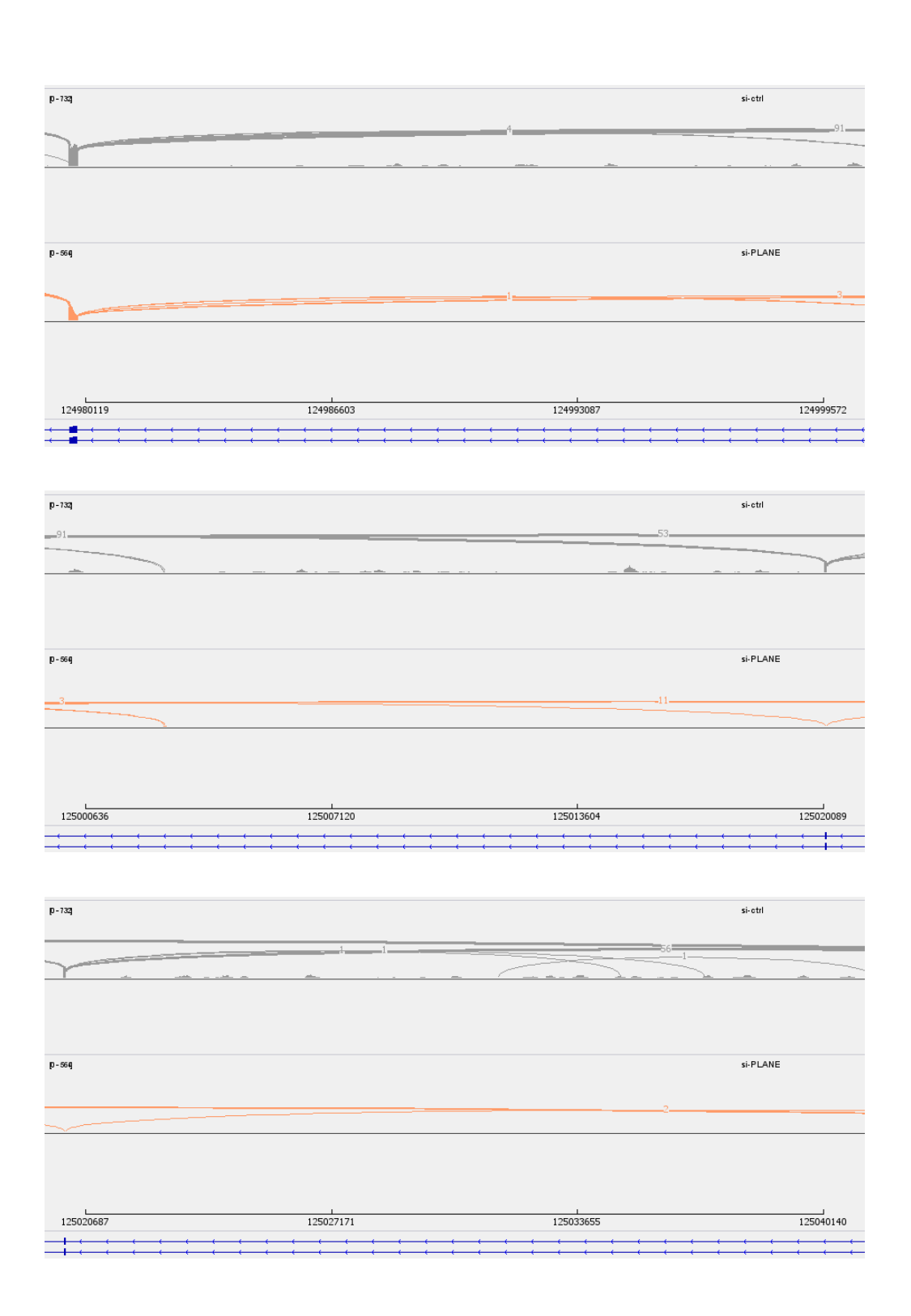

## Slide 5
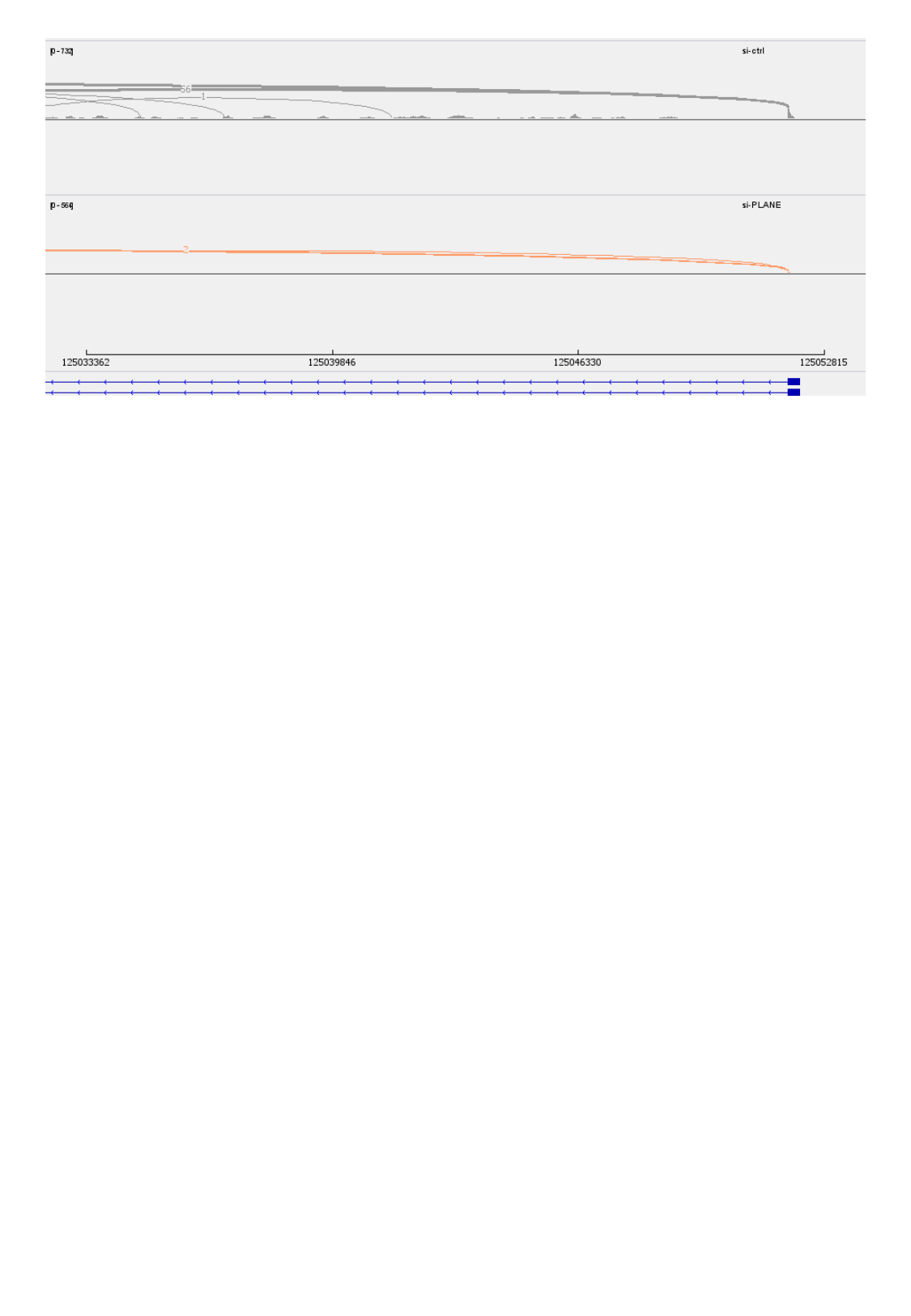

Supplement: Supplementary file 5 — Supplementary Data 2 [file 41467_2021_24099_MOESM5_ESM.zip › 287225_2_data_set_5574158_qt4lk4.pptx]
