# Supplementary material for: The pan-cancer lncRNA PLANE regulates an alternative splicing program to promote cancer pathogenesis
Source: Nat Commun. 2021 Jun 18;12:3734. doi: 10.1038/s41467-021-24099-4 (PMC8213729; doi:10.1038/s41467-021-24099-4)
Supplement: Supplementary file 6 — Supplementary Data 3 [file 41467_2021_24099_MOESM6_ESM.zip › 287225_2_data_set_5574159_qt4lk4.docx]

hnRNPM binding sites (UGUGU)，total: 515

# Three randomly selected hnRNPM binding sites are highlighted in yellow. Exons are highlighted in gray.

GAGUCUUUGAGGACACAGCCUCGCUGGAGGCAGUUUCUGGUAGGUUCAUGUCUGUCUCCA

CUUGGUGUCCCCAGGAGGUUCAUCCUGCCUGGACUUUCUCACGGGAAGUGGGUCUGGAAU

UACUCGGAGCCAGGUUCGGCCUGGAGUGUGUGGUGGGGGCCUCGGGGGGCGUGUGGGGUC

UGGCACAUCAGAGCUGUCCAGGGCAGGGAGCAACUUAGGGCAGUUUGCGGGGUCCCCCAG

UGAUGGGCCGUUUCCUGGCUGAGUUGGUGCAGUGAUUUUUUGAUGACACCUGAGAGGUGG

GAGGGUCCCUGACAGUGUCAGUGUCAGGAGGGUGGGCUUCCCCGGGCUCCGGGGUUCUUG

GUCACAUUGCUUUUGUGCUUUGGCGGUGGGCCCGCAGGUUUGGCCAGGGUGGGGCCUGUC

GAUGGGCCCAGGUGUUCGGGUGUUGGGUGCUGGAGGAAGCCCUGAGGCUGAACCACCACU

GUGGUCCCCAUGCCUAGCAGGCAAAGAGGUGGCCUGGGUCCUGGUCUUAUCACUGCUACU

UCUGUGCUGGGCACUGGGGCCAGGUUGCUUUAUCUCUCUGUAUCUCUGUUUCUCCAUCUG

UAGACUGGGCGGGGAGGCAAUAACAGUUUCCCCUUAUAGGGUUUUGCUGCGGUGAUUAAU

GCUCAUAGAGAGCAGUGCCUGGCCUGAGAAGGUGCCUGGUGAGUCAUAAUCACAGGUCGU

GCUUUUCACAGGUCACCCCAUUUUGUGACCCUCACAGCAACACUCCAGAAUGGGUGGUGC

CUCAUUUUACAAGUGGGGAAACUGAGGCACAGGUUGGUCACCUGUCCCAGAUCUUUCAGC

UGGAAGCAAAGAGCCAGAAGCAAAGAACCCCUUCUCACCCUUCCCAGGGAAUGCAGCAAG

UUUCUCCCGGAGUGCUCAUUUCCCAGCCAGGCAGAUGGUCGCUGAGGGCAUUGCAGGGCU

CGUGCGCAUGGAGGCUGGGGCACAUGGCGGGCACACAGCGUGUUCUGGCUCAUGACAGGC

UGUUGUCGGGAGAUUUCAUUCUUGUUCCAAAUACAGUCAUGUGCUGCAUAACGACAUUCU

GGUCAACGAUGGACCGCAUAUAUGACAGUGGUCCCAUAAGAUUAUAAUACCACAUUUUUU

UUUUUUUCGAGACAGAGUCUUGCUUGGUUGCCCAGGCUGGAGUGCAGUGGUGUGAUCUUG

GCUCACUACAACCUCUGCCUCCAGGUUCAAGCUAUUCUCCUGCCUCAGCCUCCUUAGUAG

CUGGGAUUACAGGCGUGCGCCACCACGCCUGGCUAAUUUUUGUAUUUGUAGUAGAGAUGG

GGUUUCGCCAUGUUGGCCAGGCUGGUCUCGAACUCCUGACCUCAAGUGAUCCUCCUGCCU

CGGCCUCCCAAAGUGCUGGGAUGACAGGUGUGAGCCACCAAGCCCAGAGAAUACCACAUU

UGUACUGUUCCUUUUCUGUGUUUGGAUACCUAGUGUACCACUGGGUUCUAGCUGCCUGUG

GGAGUCAGUCCAGCCACACGCCGUACAGGUGGUAGCCUGGGAACCGUCGGCUACACCAGG

GUUGUCCAAUCUUUUGGCUUCCCUGGGCCACAUUGGAAGAAGAAUUGUCUUGGGCCACCC

AGAAAAUACACUAGCACUAAUGAUAGCUGAUGAACUAAAAAAAAAAAAAAAAUCACAAAA

AGGUCUCAUAAUGUUUUCAGAAAGUUUACAAAUGUGUGUCAGGCCACAUUCAAAGGCAUC

CUGGGCCGGGGUUGGACGAGCUUGGAUGACACCACACAACCUGGGUGUACCGCUGGCACU

GAGGUCUAGGUUUGUGCAAGUCCACCCCCAUCGUGCUCCCACAGCCACAGCACCAGCUCC

CGUGCGUUUCUCAGAACGUGUCCCCGUGGUUAAGCAGUACAUGACUAUAUAUUCGUUUAU

GGAAUGGCUCUUUCUAAAGCACCUACUGUGUGCCAGGCUCUGUUGUGGGUGCUGGGAAUA

GACCUGUGGACAAGACGGCCAAGCACCUAGUCCUCCCUGCGGGGAGACAGACCAUGACCA

AAAGUCAGUAAGUGCGACGCUUAGCGGGUCUCUUGGUGGUGAGCACCAGGCUGAGGAACA

AGUGGUGAAGGGGUCUGGGGAGCCUGUGGGUGGGCUGGGGGUUGCAGUUUCAAAGUGGGG

GUCAGGGUAGGCCUCCCUGAGAAGGUGGCCUUUGAGCAAAGACCUGAAGUAGGGGAGGAA

GGAAGCAUGUAGGUAUCUGGGGGAAGGGUGACCCAGGCUGAGGGAACAGCCCUGCCAAAG

CACUGAGGCAGCUUGGAAUGGGCCUCCCGGGUUGCGCGAUUCUGAGUUACCUCGGGGGAG

UUUUCCUGGAGGAGGCCUCUUUACUUCUUCCUGAGCCUUUGGGGGCCCCCCACUAGGCAG

GAGGGAAGAUCAGCCCUGCAGGUCAUCUGCUUCCUGGGGCUGGGCCUUGGGCCCUAAGCC

CUGGGCCUCACAACCAGGUUUUGUCUUGGAGGGAGCAGGGGAAGGAGGAUUGGGAUUUGG

AGGUAGGAGAAGGAGGAGUGGGACUGGGGUGGGGGAGGAGGAGUGGGAUUUGGAGGGAGG

AGGGGAAGGAGAAGUGCGAUUUGGAGGUGGGGGAAAGAGGAGUGGGAUUUGGAGGGAGGG

GGGAAGAGGAGUGGGAUUUGGAGGGAGGAGGGGAAGGAGGAGUGGGACUUGGAGGGAGGG

GGGAAAGAGGAGUGGGAUUUGGAGGUGGGGGAAGGAGGAGUGGGACGGUGGCGGUCCCCC

ACAAAGUGAUGAACUCAGCAGGGCUUUCUGCCUAGGCUCAUGGGGCUUUGAAGUUGGAAG

GAAAGCGGCCUGGUCUGGGCUGUUUUUCCCAGCCUCCUCCUCCUCUGCCUCUCUGUCCCC

CGUCCAUCGGCGUCUCCGUCUCCGGCCUAAUGGGGAGCCUUCCUCCUGUGGCUGAGUUAU

CUGCUUGUCAUGCCAGUGGCCCACCCGAGGACGAUAAAAGGGCUUUUUGUCUGCAAGCAC

UUAGCUUCCUCUGCCGGGGCGAUCCAUCACAUCUGAGGGAGGCCGGGAAGGCAGACAGAC

GGUGGAGUGGGGCCUCCUCCUCCCCUGCUGAGGGAACCUGAUGCUCCCAGGAGCCCCCUU

GCAGGACCCAAGUGGCUCCUCAGCCCGAAGGCAAGGCCUGUCUGGGGGCCAGCAGGGGUG

AGUGGGAGUGGGGCCAUGGGGGCACAUUGAGGCAGGCAGAUGCUCUGCACUCCCCUGACA

GAGGACAGACGCUGCCCGGAUGCCCUGCCCCACCACACCCACUUGGCAGCUCUGUGGGGC

CCCUCUGGGACCCUCCAUGCUGGAGAGGGUGGGUGGGGGGUGCAGCCUUUUCUGAGUAAG

AACCGUGAUGGUAGAAGGGGGCAUGGGGGAGACAAGGGGGGACAGACCUCAGGGCCGACA

CUCGCUAUGCGUGCACUAAGCACUUUGUGGAAUUACCUCCUUAAAUCUCAGGGCGACCCU

GGAGGUGGGCACCGUCCUUAUCCCCAUUCUCCAGAUGAGGAAACUGAGGUACAGGGAGGC

GAUGUGGCAUCCCCAGGGUGCAGCAGCAGAGGGAGGGCUUGGCUCCAGGCUCCCAGCGGU

ACACUCUUCCCUGCGGACCUAGGACCUUAGAAGGGGGCUGUGGGAGCCCCUGGCCCCAAA

AGUGGGUUGCCCGUGACUCGAAACUCUGUGAGUGGAUUCUUCAGCUGGGAGUGGGGUGGG

GAGGUGGGUGUCUGGGAUUGUCUAUACAUUGGGGUGAGGGGUCCGGUGUGGGUGGGGGCU

GGGGUCUCGGGGUACCCUGGGCGGGUGAUCGGGGACACCGAAGGUGUGUAGGGGGAGGUU

UUAGGGCCCUGGCCCGGUGGGAUCUCACCUCUCGGGGGCUCUGGGAGGACCGGCUUUAAC

CCCAGUUGGACGGGGCCCUGGGCCCUGCUUGGGUGGGGAGGAGAGCAAUUCAGGGCCCCC

UGCCCUUCCCUGUCUGCGCCACCACCCUUCCCUCUCUGCCAUCCUCCUCUCUCUCCUUCC

CAGCCUGGAUGCUCAGCCCUGGGCGGGGGCUCCCUGCAAUCCCUUCCUCCCCUCCCCCUU

CCUUCCUGCUCCCUACCCCUCCUUCUCCUCCCUCCUUCUCCCCACAGGCCACCCUCAAAU

GACAGCAAUUAAUGGUGCCUGUGAUGGCGGGCUGAGAGGAGGAGGCUGACAGUUGAGCGU

GUCUGCCUGCGGCCGCCCGCUAAUCGGGCCCGGGGGAUGCCCCUCCUGCCGUUGGCUCCA

GGCGCCUGCCCUGCCAUCACUCAGAGGGGAGCAGGAGCCCUGGACAGGCCUGUGGGAGCU

GGUGCAGAGCCCCCAUCCCUGCAGCCCCCUCCCUGUCCUUCUAUUAUUAUUAUCAUUUUC

UUUCAUAAACGGGUGGGCUCUGGCUGGACUGUUGUUCAGCAUGAGUUGGUGGAUGCCUCC

CAGACGUCCUUCUCCUGAAAUGACCUCUCAUGUGCCUGUGUUGUCCUCAGGGCCAGCCAU

CCCCACAGGGCCCCAUCCUUUUGUCCCUGCUUCCCUGGGAGAGGCGGGUUGGGUGGAGCC

CAGGAACCGUGUCCUGUCCGGACAGCAGGCAUCAGUUAAAUGCCCCGGCCUGAGUCCUCG

GGGCCUGGGGGCAGCUAUUUGAGCCCAGAGCCUUGUCUGGUGGGGUGAGGAAGUGUGUUG

UGUUUGUCAGGGUUACAAGAAUCCUGGCCAGCUCUGUCCACAGUGGGCCUGGGCUUGACC

AUUUAUCACCUGGCAGCAGCAAUAAUGAAGCCCCUGCUGUAUGCUGGGGCCUGCAGCUGG

UAUGUUCACUUUGGUUCAUUUUACUUGGCAGUUUUCUGAGGAGUGUGGUAUUUCUCACCU

UGCUUUUGUAGAGGGGGAGACUGAGGGGCAGAGAGGCAAAGCGAUUUGCCCUAAGGGGCA

CAGUGCCCUACGUGACCUAGCUAGCAUAGGACAAAGCUGGGGUUCAACCCAGACCCAAAC

UGUCCAAUGCUCAUACAUCAGGGGACUUUGGGAAACUUGAGGGCCAGUUUAGGAACCAGC

AGACUGAUUUAGAAGUGAGGAAAUAGUCCCUGGGUACUGGGAAUGGAGUGAGUAGUGUUA

AUAUAUGCAUCUCUCUCCCUGUUCCACUUAAGAUCAUCUUCAUUGAGAUGUAAUUAACCU

AUAAUGAUGCACCCAUUUUAAGGGUUCAAUUGCAUGUGUUUUGACAAAUGUAAACAUUUG

CAUCACCUCCACAAUCAGAAUAAAGUGUUUCUGUUCCCCCCAAAUCCCCACCAGUCCCUU

GGCACUAAAUCUCUAGCACCCACCUCCACCCCCCACCCCCGACUAGAGGUACCCACUGAU

CUGCUGUUAUAGAAGCUUUUAACUCUUCUAGAAUUUUAUAAUAAAAUUAUCAUUUUAUUA

UAUACUAUACUUUGUUGUGUCUGGCUUCUUUCACUCUGCAUAACCCAUGUUGCUCAUGUA

UCAAUUCAUUCUUUCUCAUGGCUAAGUAGUAUUUCAUUCUGUGGAUAUAUUACAGUUUAU

CCAUUCAUCAGUUGAUGGACAUCAAAGUUUCCAGUUUUUGGCUAUUGGGAAUAAAGCUGC

UCUGAACAUUCUUGUGUAAGUCUUUGUGUGAACAUGUUUUCUUUUUUCUUAUGUGAAUAU

CUAGGCAUUGGGUUGCUAGGUCACAUGGUAAAUGUGUUAUUUUAGUUUAUAAGAAACCAC

CAGCUGUUUUCUAAAGUGGCUGUCCCAUUUUGCAUGCCCACCUGCAGUGGAUGAGAGUUC

CAGUUGCUCCCCAUCCUUGUCAGCACUUGCUACUGUCAAUCUUUUAAUGUUAGCCGUUCC

AGGAGACCCUAAUGCACAUGCGAUUACAUUUUUCAGCUUUUGCUGCCUUUUGGGCAAAGC

UGUGGUUCUGUGUUUUGAUUUUAUGUGUCUGGCCAGCUGGUGGGAGUCUAAAGGAUGGUG

GGAGAUAAUCUCGUGUCUGCCAACGCUAGCUCUGUGUUUUCCUUUCUAGUCUUUAUGCUG

CUGACUGACUGCUUCUCGUCCGGCUGCAGUGGUUGAGACCACAGUGCAGUGCCGGAUUGC

ACCAGUUGGGUUGUUUGGGGUUAGUUAGCUGUGAGUGUUGUUAUUUAUUUUUUUAUUUUU

UAUUUUUUUUGAGACAGAGUCUUGCUCUGUCGCCCAGGCUGGAGUGCCAUGGCAUGAUCU

UGGCUCACUGCAACCUCCGCCUCCCGGGUUCAAGCGAUUCUCCUGCCUCAACCUCCCAAG

UAGCUGGGAUUACAGGCAUGCGCCACCACAUCUGGCUAGUUUUUGUAUUUUUUUUUUUUA

GUAGAGACGGGGUUUUGCCAUGUUGGCCAGGCUGUUCUUGAACUCCUGACCUCAGGUCAU

CCACUUACUUUGGCCUCCCAAAGUGCUAGGAUUAGUGAGUGUUUUUUUUUUUUUUUUUUU

UUUUGAGUCGGAGUUUCGCUCUUAUCGUGCAGGCUGGAGUGCAAUGGCACGAUCUCGGCU

CACUGCAACCUCCGCCUCCCGGGUUCAAGCAAUUCUCCUGCCUCAGCCUCCCUAGAAGCU

GGGAUUAUAGGCAUGCGCCACCACGCCUGGCUAAUUUUGUAUUUUUAGUAGAGACGGGGU

UUCUCUGUGUUGGCCAGGCUGGUCUCGAAUUCCCGACCUCAGGUGAUCUGCCCGCCUCGG

CCUCCCAAACUGUUGGGAUUACAGGCGUGAGCCACCGCGCCCAGAGGUGUUGUUCUUAAC

ACUCAUCUUACAGAUGAGGAAACUGAGGCUCUGACAGGUCAGGUGACUUCCAUUCCGAUA

AGAAAUGAAACUCAUGACUCUGAGUUGUGUGCCCUUUCUCCUCCUUCAGGCUAGGAGGUG

CCAGCUCCGUUUGUUGCAGUGAUGUGUUUGCCAGUCCAGAGGGUCAUGCCAAGAAUUGGU

GGCUGGUUGGGGCCAGGACCACCUUUUUCCUAGGGUUUCCUCUGCUGGAGAGAUGAGGGU

GUGGAGGAGACGCUGUGCCUUCCGGAUCAAGCACUGUCCUUUGGCACUGAGCACUGUUGA

GUGAAUGUCAGGCGUUGGUUCUGCCAAGGGUCUUCCUUGUUCAAUCCUGUUGUCUUGGGC

AAAACCACACCUGCCUUCCGGGGAGCUAUUUUGGGGCAAGGGAGGAGCAUGGCAUAUGGA

AGCCCAGGAAUGGGCUGGGAUGGAAGAGGGGGCCUCCUUCUCCUUUUUCCUUGCCUGAGG

GGUGUCAGCUAAGAUGACUUUUCGGGGAAGGAGGCCUGUAAAUUAAAUAAGCAAUCCCGU

AAAUAAAUAAGUAAUGAAGUCAGCGGGGUGCCUAAUUACAUCAGAUUCAUCAUUGUGGAU

GGAUGCCUGCCCGGUCAUUGUACUUGUUUUUAAUCUUCGGCUCUGACAUGUGUCUCCUUC

CGUCUCAUAGUCAUCCCCACCCCAGCUGGAGGGAGGAGGCGGGGAGGCGCUGCUGUAGGG

GCUUCCAGAGAGCCUUGGUGGUGGAGGUGGUGGUGGGGUCCCUGGAGGGUUAUUUACCCA

GUCUGCCUGUUUAUGGCAGUCGUGGAAGGGAUGACGUCUGUUCCGCAGUGAAACCUCAGG

AGGCCUCGGGCCAUCCUGAUUCAGCAAAUCCCUCAUCAGGCUGCGCUGAAGGGUAGAUGA

CAAACAUGGUGCCCCGCACAGAUGGGGCCACAGCACACGCAGCACGCCCUGGAGGGCGAG

AUGGGAAGGACACGGACGGGGGCAGAUGACCUCAGUGCAAAGUCCCAGGGAUCCAGGUGG

ACCCGUCUUUAUGCUGCAGUUGGGUUUGUAAACCUACUGGAGCUUCGAAGUGGGAGCACC

CCAUGGCAGUGACAGCAGCAGUCCUAACAGGGUUUGUGAUCGUCCUUGUGUCACACUUCA

CAUGUUAUAAUUUAUUUAAUUGACUGGGCACAGUGGCUCACGUCUGUAAUGUAAUCCCAG

CACUUUGGAAGGCCGAGGCAGGCGGAUCAUCUGAGGUCAGGAGUUCGAGACCAGCCUGGC

CAACAUGGUGAAACCCCGUCUCUAAUAAAAACAGAAGAAUUAGCCGGGCGUGGUGGCGGG

CGCCUGUAAUCCCAACUACUGGAGAGGCUGAGGCAGGAGAAUUGUUUGAACCCAGGAGGC

AGAGACUGCAGUGAGCUGAGAUCGUGCCACUGCACUCCAGCCUGGGCAACAGAGCAAGAC

UCCACCUCAAAAAAAAAAAUAAAUAAAAAAUUAUUUCAUCACCUAAAAGCUCUUGUAAAG

UAGGGAUUAUCAUCAUCCCCUCUUUACAUAUGAGGAAACUGAGGCACAGAGAGGUUCCGC

AUCUUGCCCGAGGUCACACAGCCAGUGAACUUUGCACUCAAGUCAUCUGCCCCCGUCUGC

GUCCUUCCCAUCUCACCCUACAGGGCGUGCUGUGUGUGCUGUGGCCGCGUCUGUGCGGGG

CGCCGUGUUUGUCAUCUACCCUUCAGCACGGCCUGUUCAGGGAUUUGCUGAAUCAGGAUG

GCCUGAGGCCUCCUGAGGUUUCACUGCUGAAAAGAUCUCAUCAUCCCUUCCACGACUGCC

AUAAACAGAUGCCAUUCUGAACUGUAAAAUAAAAUCAUGUUUCACUAAGGACAAAGCAAG

CCCAUUUGCUGAAAUUAAAAUAGCUUCUUUCCAGAUCUUCUCUUUCUCUUUCAGCGGCCC

UGAUUGGCUGGCUCUUGGGGGACUCUCUGUCCUGCCUGAGAUAAUGGUCCUUAGACCCAU

UGUACAGUUGGAGAAACUGAGGCUCAGGGAGGUGGAGGAGCAUGAGUGAGACCACACAGC

UGGGAGGUGGUGAAGCUGGGAUUCUAGCUGCUUCUUACUGUUUGGUUUGGGGAGCGGGGG

CCACCUCAUGUGCCUGACGUGGAAGGGGCUGCCUGUCCUCUCCGAGAAGCGCAGGGAGCU

GUGUGCACCCCAGGCUGGCUUUGCUGGGGUUCUUGCUGUGGCUGAUUGGUUAGCGUCCUG

UCUGUCCGCGGGGGAGAGGAGACCAGCUGCCCCACCUCGUGGUUUCUCCUCAGGACCUGA

GCAUGUGCUGGGCAUGGACAGAGGAUGGGGGUGGCUGGGGGCAGUCUUGUCCUCGUUCGC

UGUCCAUGCAGCUCCAGGGUGCCCAGCCACAGCCCACACCUGUUCCCUCCAUGGUGCCUC

UCCAUAUCUCCAGACUCCAGGUCCUGUACGAGGCAGUGGGGCCUGAACCCUUGGGAUGUC

UGGGCCUCUACUGGGGGCCCAGGGAGGGAACACCCUUUACUUUACAGGGUGUACUUGUGC

AGCCGGACAGGGCCUGCGUUGCUGGGCCGUGGCUUGGAUGGGAGUGGGGGUUCACACAUU

CCCCUGGUGCUGACUUCAGGCUUGCGGGGUUUGGGGCUCUCCCUGCCCCUCACUGUCACC

UGGGGUGGAGAGAGGAGUUGAGGAAAGACCCACAUGAAGAAUUGUGUGUGUCCAUGGUGU

UCCUGGGGGUUGGAGCAAUCCAAGGAGGCUGCCUGGAGGAGCUGGAACACUCACAUUGAA

UAAUCAUAAGCCCAUCUUGGAGGGACUAAUGUGUGUACAUGCUUCAAACAGGACCUGGCA

CAGAGCAAAUACCAUAUUUACCAUAAAAGCUAACAUUUCCUGAUCCUUUUCUUGCCUCAG

UGCUGUGUGUGAGUUGCCAGUAUUAUUAUUAUUAACAUUAACUACAAUUUCUAACGUGCA

CCCCAUACAUACACGCAUGUACUCUUUGCAGCAGCCUUAGAAAGUAGGUGCUAUUAUGAU

CAGCCCUGUUUUCUAGUUAAGGGGAUUGAGGGCAGAGAGAGGUUAGGGGACUUGUCCAAG

GUCGCCCAGUUGGCAGAGCUAGGAUGUGAACCCAGAACGCCUGACUCCACGGCAGCUGCU

UUUCAUCGUGUGGAUGGUGCAGGUGCCACCUGCAGCCCUCCCUCUGCCCCGUGGGUACGC

CGAUCACUGCCGCAGCCAGCCUGGAUCUCUUGGCAGGGCCAUGCCAGCUGUGGGACAGCC

UCCAUGUGCCACGUAGGGCCCUCAGGCACGCAGCAGCUGCUCUGGGCAGUGUGGGAUGGU

CAGGACGUGGGACAUCUGCUUCCUGCCUCCCUGCAGCCAGAUUGGUGUCCCCACGGCCUC

CUCCUGGUUGAGGGGAUGGCAAACAGCAAACAAGAUGCGGCGCUUUGCCUUAUGGAUCAC

ACAGGCUGAUGGGGAGAUGGGCAGUGCCAAAAAACAAACACGAUUUUGCAAGGCAAGAAA

AUGCUGCCUGUGAAAUAAUACAGGGUGGGGAGGUAGGGAUGACUUCAGUUUCGGGGGACU

CAUUUUAGGUAAGAAUUCCAGGGUAGCUUCUGAGGAGGUGGCAUCUGAGCUGGGCCCUGG

ACAAUAAGGAGGCACCCACUAUGUGAAAGGAAGGGGGAUGAGGGAACAGCAGGCGCGAAG

GCCCUCCGGCCACCAUGUUUCAGGCAUGCUGAUUGGGGUUCUGUGCCUCAGUUUCCCACU

CUGUAAUGGCUGGAGCAGAGCAGGGGGGCCCCAGGAGGGCUUUCCUGGAUCUGAGACUGU

CCUGCAGACCUCCAGGGCUGGGCUGUUUUUCACAUUUAAGGCAGAAGUCAAAUUUAAUGA

CUUUUGCCUUUCCCUUUUGUUCUUUUGCUCCAUAGUUAAUUUCCAUGUAUUUACAUGGUU

UUAAUUUACAUGUAUUGGGCACGUGGCAUGUUCCGGACUCAUGUACGUCACUGGGAUGAG

AAUGGUGGAGAAGUUGGACAUUGCCUGUGCCUUCGAGUUUAGUAUCCAGAGGGGGAGUGU

CAGUUAAACAAGCAAGUGCUAUCGCGGGUGGUGAGGGUUGUGAGGACGAGGUUCAGGCAU

UCACCUGGGUGAGGCAAGAGCUGAAUUAGUCUUCCAUCAGGCCAGGCGUCCAGGGACGAG

UCAUUCAAGGGGAGACCUGGGGGUGAUUAGUUAGGGGCAGUUAGUUAGGGCUCCUGAGUU

GUGGCAGAGACACACUCGAGCCGAGUUAGGCUAUGACGGGGAGUUGAUGGGUCCUCAGAG

UGGCCAGGGUAGGACACGUUCAGGCACGGCUGGAUCUAGGUGCUCAAACAAUGCCUCUGG

GAGCUGGCUUUGCCUCAGCUGUGCCUCCUUCCAUGCGGGCCUUGUUCUCAGACAGGCUCA

UGCCACCAGCCCAGCCCAGCCAGCCAAGGAGAAGGAGAGUCACCUGGCAUCCCAGGAGGG

ACACUCCUUUCUCUUGUUUGAGUGAGGUCCUCGACAUUUGUUUCACUGUUUGCAACAGCA

AGAGGAGGUGGCCAAAGAAGGGAUAAGGGGAUGUCCCAAACUCAGACACAGGGAGGAAAG

GAGUGAGUGAGUCAGUGCAGGUCAAAGGGGAGGAGUGGGGAUUGUGGCUAACUGGCAUGC

UGGGUAUUCUGAUGGUUUUCCAAGAGAAGCUGGAAAUCUGGAUUUUAUAAGGAAUGGCCU

AGUUUUUAAAAAACAUUGAAUGGGCCAGAACAAGUUUUCUUCAUGGCCACAACUGGCUGU

CACCAUUUUAUAGCCUCUUCUUAGCAGUGACUUUUAAAGUAUUUUGUCCCCACUGUGAUC

UGGGUAGAAUGUCCCAUGGGCUGUUCCAGAAGGUCUCAAGUCUUGGGAAGUCACGUCUGA

CACCAUUGGAGAUGCCAUUUCUGAAAGUCAAGGCCCACGGCCACAGCAGUUUCUCAGAGA

ACAAUACCAUGUCUCCAGUGGGGCCUGACAUUCUUGGAGACAGUAUAGUCUAGUGGUUAG

UGUCGUAGAUGCGGGUUUCACACUUGCUGAGUUCAAAACCGACAUUGCCACUUCCCAGCC

CCGUCCCCUUGGGCAAGUCACUUCACUGCUCUCUGCCUCAGUUUCCCAGUAUGUAUUAAU

GGAAUGGGUAUGACAGCACCCCAUAGGAUUGUUGUAAGGCAAGCUUGUCCAACCUGCGGC

UCAGGACGGUUUUAAAUGCAGCCCAACACAAAUUUGUAAACUUUCUUAAAACAGAUUGUU

UUUCGGUUUUUUGUUUUUUUUUUUUUUAGUUCAUCAGCUAUGGUUAGUGUUAGUGUUAUG

UGUGAUCCGAGACAGUUCUUUUAGUGUGGCCCAGUGAAGCCAAAAGACUGGACACGCCGG

UUGUAAGGCACAGUGGAGGCCGCGUGAAGCCCACUGACCCUGGCACACUGAGAGCUUGGG

UGAUGCUGGCCGGCGUGAUGUGUCCUUCCCGGGUUAGGUCCCUGGGAUCCAGCUGGCUGG

CGUGGAGCAUUCCUGGGCACUCCUCUUUCCUCUGCCGCUGCCCCUGCCACCGGGGUGUGG

GUUCCCAGGAGCCCCAGAACAGGCAGCCACCUGCCCCCUGCCCCCAGCCGUCCUUGCCAG

UUGCUGGGUGCCCAUGGUAUUCUGGCGUGCCGGAUGGUGCUGCAGUUGGAAGUGAGUCUU

GAGUGAGCAAUUAACUCUGCCAGCUGCCAGCUCACAGCUGCUGUGGGUGGGGGCGGCCCC

UUUUAAAAAUAAAAACAACAUGCAGAAAAACAGCCCUGGAGUACCGUUGGCUUCCCUUAG

CCAACGUGGUCAGGGCUUCAUUUUGAUGCUUUUGUGGAGAAGAAUCCCGGGCCUGGAUGG

GGAAAUAGGAUGCAUUUUGGUCUGGGGAGUGUCUUUAUUUAGGGGCAGAGGUGAUGGCCU

GUGUCUGGCCUCCCCAUCCAGGAGGCAGGCCCCUUUCCUCAAGCUGCCCACGAGUGGCUG

GCUCAUGGAUGCCUGCCACAGGGAAGGGGAUUUGGGUUCCUUGGGGCCAGUUUUUAGCAG

GCUCAGGCCGGAGAGGGUGGCUGUGGGAUCUCUCAGGUCUGUUUCCAGCUCUGUCGGCUA

UAGGGGGGCCCUGGGGCCAUCCCGGAGAUCCCUGGAGCUCUCCUCUGCAGCUUCCAGCCA

GUUUUCUGGAACUAGCGGGAAAGGAAAGGGGCACCAAAGGUGUUUCCGGGCAUGCGACUG

CUGCCACUUUGAGGUCUUGGCUUUGGUAGUGGGUUGAGUCAUGGCCCAUGAAACAUAGGU

ACACUUGGAAACUGUGAAUAUGACCUUGGCCUUAUUUGGACCACAGGUCUUCGCAGAUGU

CACUAAGGUAAGGGUCUCAAGAUGGGAUCAUCCUGGAAUAGGAUGGGCCCUAAAUCCAAU

GACAAGUGUUCUAAGAGAAGACACACAGCAGAGAAGUCCACGUGAAGACGGAAGGAACGC

CAGGAGCCACUGGGAACUGGAAGACACAGAGAAGGCUCCCCCUGCAGAGUCUUUGGAGGG

UGUGUGGCCCUGGUGACACCUUGAGCUUGGAUUUCUGGCCUCCAGAACUGUGAGAGGAUA

GGUCGCUGGUUUCAGCCACCCAAUUUGUAAUAAUUUGUUAUAGCAGCCACAGGAAACGAA

UACAGUUGUGAACUUUAUUUUAAUCACUCAUGUAUUUUAAAAUUAAAAUGUAAAAUGUAA

UUUUGUCUUGUUCACUGCCAUAUUUCCAGUGCUCAGCACAUACAGUAGGGGCUCAGCAGA

UGCUUCUCGAAUGUCUGAUAGCUCCAGGCCUGGGUGAGGAACUCCUACCUGCUGGGAGUU

GGACCCAGCUUUCAGGAAGUCGGUAAACUGGGCUGGAGUGGUGUGCAUUCAGCAACACCA

UCUUUUUGUUUAUUUAUUUGUUUAUUUGAGAUGCGGCCUUGCUCUGUUGCCCAGGCUGGA

GUGCAGUGGCAUGAUCUUGGCUCACUGCAGCCUCUGCCUCCUGGUUCAAGCAAUUCUCUU

ACCUCAGCCUCCUGAGUAGCUGGAAUUACGGGUAUGCGCCACCACACCUGACUGAAUUUU

GUAUUUUUAGUAGAGAUGGGGUUUUGCCAUGAUAGCCAGGCUGGUCUUGAGCUCCUGGCC

UCAAGCGAUCCACUGCGCCCGGCCUAUUUAUUUUCAGUUGAGGUGAAAUUCACAUCACAU

AUAAUUUACCAUUUUAACAUGAACAGUUCAUGAGUUCUUAGUACAAUCACAAUGUUGAGC

ACCCACCCCUUCUAUCCAAAAAAUGUCCUCAUCACCCCAAAAGGAACCCUGUACCGGUGG

GGUCACUUCACGUUGCCGCCUCCCGCAGCCCCUGGCAGCUACCAAUCUGCCUUCUGCCUC

UGUGGACUUACCUAUUUUGGGUAUUUCUCCUGGACGGAAUCAUGCACUGUGGGUUUUGGC

UUCUUUCACUCCGCACAAUGUGUUUGAGGUUGAUCCGUGUUGAAUGGUGUGAGUCCUUCA

UUCCUUUAUAUGGCGGAAUUCCAUUGUGUGGCAUACAUCAAUAGAACCAUUUGAUAUCUA

AGUGGCUUUUCAGAUGAGGAAACUGAGGCUGCCCAGGUCACGGAGCCCUGGGGCCCGGGA

UUGAGGAGGUGCAGAUAGCCAGACGUGUCUGGCUCACGGUGCUGUCUCUUUCUCCUGCCC

CCACCCUGCUCCACUCGUAUCUGUGCCUGGCCAGGAAGCAGCAGUGUGUGGGGAGGAAGU

GAGUGGGGGUCUCUUCCAGCUGGAGCCGGAUGCUGAAGCGUAGCUGCGGCUGUGGGUCUG

GAUCUGGUCCUCAGAGUUCUGGCCCCCACCUCCCCCCUGGGCCCCACACAUGUAGUUUCU

GUUGACCUGGGUUGCGAGAGAGGCAGCAGGCAGGAGCUCCCCCAUUCGCCAGCUGAGCCU

UCUGAUGCCUCGAGAGGCUGGGGGCCUUGCCUGGGGCCACUCAGCUUAAGGUCCCCCUGC

CCCUUUCCAGACCCCCUUUGGCCCACUGUCCUGGCCCCUGUGGCCUGCGGUGAAUCAUAU

UCUGGGGGGUCUUCACCGAUCUUUCCCACCGUCAAAGCUCUGCUCAGCAGACGGGCUCCA

GGAAGCGGCCUGUCGGCAGAUUUUGGCCAGGGGAUGCUUUUUCGAAGUAUGGAGGGGUGG

UCGCCGUCGAAGGCUUGCGUGAUGGGGAGUUCUUUUGCUGAGCAGAUAACCAGACACUGG

CUUAUUCUGAAUAUGAGUUUGGUUUUGGCCACAGGCUCUGUCUGGGGGCUGCUGCCUGUG

GCUGUUUCUGGUGUUUGAUGAUUUGUGGUCUUCAUUAAGUCACUCAACAAACAAUUUGCU

GCGGAGUGUCUGGCGGGGAAGACACAGGAAGCAGAACGAGUGGCCGUGAAGAGACAUGCC

CAGUGUCAAACCUGCCCAUAGCACACAGUCGCUCUGAGGGGGUGGGAGGAGUUACCGUGG

AUUCCAGGGCCAGCCUUUGGCGAAGGUCUGGAUGCAGGUCUGUGUCCAAAACAAACAAAA

AUGAACAGAAGGAGGUGGUGAGGCCUGACUCGGCCGCUCUCAGGCACCGCUUCCUGGUGG

UGUUUGCAUGGGGCCAGUUUGGCCUGGGUGUCAUGGUCCCGCCUGGUCUUGGCGACGGUC

UGGGCGGGAAGAUGGUGCUGGAUCUUUGGGCUAAAAAUAUGCCCGUGCCUUCCUGUGCCU

CAGUUUCCCUACCUAUAAAAGAAGGCGUAAGAGUAGCAGAAUCCCACCUUUGGCUUUGGG

GCAGGUCUACGGAAACCCCCCAGGGGUGUUUCCUGGGGAAGGCUAGAGGGCCAGCUGUUC

ACAGGAGACAUCUGGCUUCCAGGGGCCUCCGCAGCCACUAUGCUCAUCCCCUCGUGGCCA

GUCUGGGCCCCCUGCCAGUCCAGUGGCCACGGGCCCCUGUCACAGGCAAAUCUGGUCUUA

GUCCUGAAACCCUCCUGUGACUCCUUGUGGCUGAAUUACCACGUGGAUAGCGGAGACAAG

CCAGGCUUGCCCAGUUCAAGUCCCAGCUCAGCCACUAGCUGUUGACCUUGGGCAAGUCAC

CUACCCUCUCUGGGCCAGCCUCCUGCUCUGUCUGUGUGUGGCGGCUCAGGGGCAUACCUG

CCUAUGCCGCGGUGGUGUGAGGCCAGCUGGUGAACCAGGGGACCCGCUUAGCACAGUGCU

GGGCACACAGUCCGAGCUGCUGGGCUCGGUGUGUGUGGCCACUGUUAUUGUUAUUAAUAU

UAUUACUCUCAAGAUGCUCCUGAGAGUAAAAAGGAUGACAAUGUUAUUGCUGGAGUUGAA

GGCCAGGCCCAGAGUCCAUCUGACCACUUCCCCUCCACUCCUAUUAGCUCCCUGACCUCA

UCGUCAAACUCUCCCCGCCUCGCCCCUGCGCCCCGGCCACACCAGCCUUCUCUCUGCUUU

GACAACACACCCGCGUGGUCCAGCCUCCAGACCUUCACAUCUGCCAUGCUUCUGCCAGGG

GUGCCUUCCUCACAUCUGCACCCCCAGACCCUUGUCAUCAUGUGGGCUGCUGUCCAGGGA

CACAUUCUUAGGCCCAACUCUUGGCUGCUAGGGGCAGGGAAGUGAAUCUGGAGACCCUGG

UGGAGCCUCCUCUGCCUCCUGGGAGCUCCAGCAGCUUCCUCUGGCGCCUCAUCUCUGCUU

UCCAGAGCACUUGGAGAAGCAGAGCUCAGCCUGCCCCUUCUACGGAUGGAAAGUUGACAC

CCAGAGAAGCUCAGACUUGUCUCUGAGUCACACAGCUAAUUGCAGCCAAGGUUCAGACUC

CUGGCUGACUACCAAGCCAUCAUUGUUGGCCUGUGUGUGUGUGUGUGUGUGUGUGUGUGU

GUGUGUGUGUGUGUAUUAUUUGGCCACGUCUUCUUUUCUAGGCCUGUCACCGAGAUGACC

CCAGAUCAGAAUGCCAGUGUGAGGGGGUCACGCUUUCUUUUCCUAGGAUUAAAAGGAGGA

CAGAAAUAUUUGCAAGGGAGAGCCUGCUCCUCUGGCCAUCUGGCCCUCGGGACAGAGGAC

GGGGGAGGGAUGGGCCGAGGCCUGGGUUCGGAACCAGGCCGGAGCCAAGGCGCCAGAGCU

ACGCUCAGGUUGGAGGUGCAGGUGCGGCUCGAAGCUGGACAGUCCGGGCUGGGAGGAAGC

GGGUUAAAGUGGGAGAAGCGGGGCUGGAGCUCUCUUUCGCCUGCCAGCGAGCGAAGAGCC

GAGCUAUAAAAAGGCCUCACAGUGUUUCAGCUCCCGAGUGUCGGCUGGAAGCCCGCCAGG

GUUACCAUGGCGAUGAGGAAUUAUUACUUACUGCCAAGGCUGGGAGAAAAAGCUCGUACU

UUUGGCUUCCAAACAAGAGGAGUGGACUUAUGUAAUUCCCUGUGUUUAUAGGCCCAGAGU

GGCAGAGGCGAGAACGGAUCGCUGGAGGCCCGACGUCUCGUUCACGGCCCAGCCGUGGGG

UCAGGCGGCCCCGCACUUGUCGCCGGUGGGCCUUGGCCUGCCCGGGUUUGGGGGGCAUCG

GGCUGGGAGCCUGGGGGGCCUGGCCUGGCCCUGCAGGGCCUCCAGGGCCGGGAUGGGAGU

CGUAGUCUCGCGGGAAGGUGAGGCCGCCCUCUGGGGCCGCCCCUCUGCGGGUGUCAUCCU

GGAGGAAGGAUGGGUCUGAUGUUGGCUGUGGUGUGCCUCCUGGAAGCCGGCAGCACAGCC

UGUGUGUGUGGACUCUGCUGGCCCGCUGGCUGGCACUUUUUUUCUUUUUCUUUUUUUUUC

UUGUGAGAGAGUUACUUCAAGUUGUGUUUGGAGCUGACGUGAGCGAGGUUGGUCAUGUGC

UCGGGUAAGAGAAGUCGCCGGGCUUGGAGUGGGCAGGGAGGUGCAGUGUGGCUGGGUCAC

CCCGGCCGGGGGAGGUGGGCGCAGACUGGGGUGGGGGGCUGUGGCCUGCAGGCUCUAUGC

AGGGGCGCUGAUUGGGGGCCGAGGAAUUCAUCUGGAAAGAGCCCCAGUUCUGUCGCUGGA

GGUGGUGGCCAGGGCUGGGGGCUGAGUGGCCGGCCGCAGGUCAGCCAAUGGGGGCAGCUG

GACCCUCCCCUGGCAGCGCAGUGUUGUCGGCCCUGAGCUCAGCGGGAUGGUGGGCAGGGC

UGUCUCAUCGGACGAAGUUGCUGCAGUCACGGAAAAGAGCGCUCGGAGGCGCUGGCCGGG

CAGGCCGGGGUGAGGCUUCUUGGCCGGCCCGCGUGGGAGGGGACCCGGGUGGGCACUGUG

CUCCUCUCCUUGGUGCACAUCCGGCGUCCCUGGGGGGGAGCAUGGCGAGCCUUGGGGGCU

CAGGGGGCUUCUGUUCCCGGAGUUGACUUCAGGAGGGCAUGUGAUCGUGGGCGCUCCCGA

GGCUCUGCCCUGGGGCUCCGCGGUGGGCUCAGGCAGGGGAGAGCCAGGUGCAGGCAGCGC

UGCAGCCCGGCCCCGGCGCCCGGCUCCCCACCGAGGUGGCGCUUGGCUCGGUGCCCAUGU

CUGGUUUGAAAUCAGCGCCACCGGCCCAGGCUUGCCCAGGGACUUGGCAGCGAGGAGCCG

GGAUAAACACUGGAAAGUUGGAAGGGCUUCCCGAGCUCACAAGGAGCCUUUAGUUGCAAA

CCGGGGGGAUGAGGGAGUGAUUUGUCCUGAGGUCUGGGUGCGCCGCGGCCAUAGGGAGGU

GGCGGGAAUGGCUCCUGGGGACCUCUGAGCCCAGGCUGGGGAAGGGAGGGGGCUGGGCUU

CCAGGCAAUGUUGCUCCUUUGCCACAGACCUGGGUUUGAGUCCUGGCUCCGUCCUCUCUA

UGCUGUGUGACCUUGGGCGUGUUGCCUCCCCUUUGCAGACCUCAGUUUCCUCGUCUGUAA

AAUGGGGCACAGUGGGGUCUACACAAGCAUGGGACUGACGGGAGAAGGAGCUGCAGUUUU

AGGGGGAACGCGGGGCAGACUGCCUGGGUUCCUGUCCUGGUUGGACACUUCCUGGCUGUG

UGACCUUGGGCAAGUCACUUACUGUCUCUGAGCCUCAGUUUUCCCAUCUGGCAAAUGGAA

GUUGCAUCUUUCCCCACCUCAUAGGCUGUAUGGCUUAAGGGAGUGAAUGAAACCUGCCUU

CUAGCUCAUAAUAGGCUCACAGUUGAUAAUUCACUGGCACUUUGUUUUCUCGAUGCUGAC

AAUACCGGCACCUGGUCUGAGGGUGGGGGGCCCCUGGGAUUUCAGCAUCUCAGGGCGAUG

GUAUUUAUGAAGUUGUAGUGGCCGGACUGAGGUUCCAGUCCUGGCUCGACCCCUCAAGGA

UGGUGUGACCUUGGGCAAGUCCCGGAAUCCCACAGAGCCUCAGUUUCCUUGUCCUUUACC

ACAGUGGUAGCACUGCAGCCUGCCGGUGUGUCUCGGGAGAGCCUGAGAGAAGCCUGAAGG

GGCCCGCACAGUGCUGGCCUGGGUGGGCCUCCGUAAUUGUAACCACAAGGUUGGAUUUAA

GUUCGAGUUCCGUCCAUUUUUCUUUUCUUCUUUGCAGAGAGCUCUCAUGUUUUGUAGAAA

CCUGGGUCCAGAUAAUGGGAGGUAGGUCGAGUGGGUGCUUCCCAGGCUCUGCCUUUGUUU

GGGAUCCCCAGGUCUCCCCCCAUGCCUGCCUGUUCUGCCCCGUCUCCCACUCAGCCUGAG

CCCCUCCGUGCCAGCCCUGGGCCAGGCACCCCCCACAGUGCCUGGUGUGGCCUGGGGACU

GGCGCGGGAGGGCCAGUUCUCCUGCAGAAGGUGGCAGCCGUCCUGUGUUUGUAAGUGACA

CCGGAUGCUGGGCCAGUGACUGGCUCAGGGCCUCCGCUCUUGGGCCAGCAGGAGCCAGUG

GGGAGUUCCCCGGGCGUCCAGGCCCCCCGCACCAGCUGCCCGCUGUCUCAUUGUCAUGGG

GCCUCUCCUGGGACCUCGAGAAUGGCCUCCGAAGGCGGGGGGGUUCUCAUCCAUGUCCCC

UUGUCACCCCCAGGGCCUUGGUGUCCACUUGGGUUGGCCACAGAGCUGUGUCGUGGCUGC

CCCUGACCUUUGCCAAAGUCCUCAGGGACUCGAUUUUCCUACCCGGUGGAGAAGAGUCCU

GGGUGAGGUUCAGAACAGAAGUCAGCACCAGGGCCUUUGUGACGAGGCAGCUCAGCAUGG

ACUGGUUGUGAUCACUGGAGUCUGCACAUGUGAUAAGAACACAUAGAACUAAAUACGCGC

ACACUCACACAUGCACACACAUACAUGCACACUCACGCACACACCCACAUGUAUACUCAC

ACGCACACUUGUGAACAUACACACCAUACACACAGUCACACGAACAUGCACACGCAUACU

CGCACACUCACACUCACAUGCACACUCACACUCAUAUGCACACUCACAGUCGUGCACACA

CACCCAUACUCACAUGCAGACACCCACACAGAUACAUGCGCACAUGCACACACACACUCA

CACAAGUGAGUGCAUUGGAAACUGCUGCAAUCUGCAUACGGCUGGCUAUAUCCAUGCUGC

CUUCCUAGUUAUGAUAUGGAUGAUGAAUUGUCGUUGUCUCAGGUUUGUCUCAGGAUGUUA

UUCUUGGGGGAGGCUGGGUGAAGGGCACACAGGAUCCUGGGAUCUUGAUUUUUUUUUUUU

UUUCCUUUUUUGAAACCGAGUCUCACUCCGUCACCCAGGCUGGAGUGCAGUGCAGUGGCA

CGAUCUCAGCUCACUGCAACCACUGCCUCCUGAGUUCAAGCGAUUCUCCUGUCACAGCCU

CCCAAGUUGCUGGGACUACAGGCGCAUGCCAUCAAGCCCAGCUAAUGUUUGUAUUUUUAG

UAGAGGACUCCCCCAUGAAGCUGGCCUGUAACUGAGCUUGGUCCCUUGGACAUCCCAGGG

GGUCCAGGCAGAGUUUGCUUUGUGCCUCAGGAUCACAGAGGGCUCCAGAGCAUCCCAGGC

UGGGGGACACGUGGUGCCUGCAGGGCCUGUGGGGGCCUCAUGACUGAGCUGCUGUAAGGG

UGUUCCUGUCAUCAGAGUGGCGUGGUGGAGGCUUGUGUCCCGUGGGCAUGGGUCGUAGCU

UAGGGUCUGGAGAGAUGGGGGUGGGAUAGGGUUUGGGGGCAUCCAGCCUCAUGAGCCUCU

UCUUUGCUGUUCUUUGGGGCCCCUGAUCAUGGGCCUGCCUCAGGACCUUUGCACUGGCUA

UAACCUUUUUUUUUUUUUUUUUUUUUGAGACAAAGUCUCACUCCCCCAGGCUGGAGUGCA

GAGCUGUGGCCACCUUUCUUUCAGGUUCAAGCGAUUCUCCUGCCUCCUGAGUAGCUGGGA

CUACAGGCGCAUACCACCAUGCCUAAUUUUUGUAUUUUGAGUGGAGACAGGGUUUUGCCA

UGUUAGCCAGGCUGGUCUUGAACUCCUGACCUCAAGUGAUCCACCUGCCUCGGCCUCUCA

AAGUGCUAGGAUUACAGGCAUGAGCCACCACACCCGGCCUGGCUGUAACCUCUUCUGGGA

ACACCCUUUCCUGAGACCUUCAGCCUCCCUGAUAUAGGCCUCUGCUAAUGGCGCCUCCUG

AGAGAGGCCUGAUGUCAGUUGUGACUCCCUGUGACACCUUCACUACUCCUUGUAACACUG

UCACCACCAGGGUCUGCGGUAUGCCCAUCACCCCUGUCCCCACCUUGUUUUAGUCUAUUU

GGACUGGGUGGCUCACAAACAAUAGAACUUUAUUGCUCACAGUUCUGGAGGCUGGAUGUC

CAAGACCAAGAUGCUGGCAGAUUUGGUGUCACAUGGGACGAGGGCCCGUUUCCUGGUUCA

UAGAUGGUGGCUUCUCCCAGGAAGAGGGGCAGGGCGGCUUUCUGGGGGCACCAAUCCCAC

UCAUGACAGCUCAUCACCUCCCAGAAGCCCCACGCUCUGACAUCAUCCCGUGGUGAUUAG

GUCUCAACAUGCAGAUUUUGGGGGGACAUAAACAUUCACACUCCAGCACCCCUCCAUCCA

GGAUUUUGUUCAUCUUUGUCACCGCUGUGUCCCCAGCACUGGGUAUGUAGCAGGCACUUA

GGAGACAGUUGUGCCUGACUCAGUGUCUUCCACGGGGCCUCAGCCUGUCCCCUCCCCAUA

AAUGGGGCCUCUCUGGUGAAGGAGGCCCUGCAUUUUGGGGAUGAUUUGGAAAACCAGGAA

AAGUUCUCAUGCUUUUUCUCUGACUGCCACUGGGGUACCCACCAAGCAUCCCAAGAGAGC

UACCCCUCCUAGAUACAGUCAUGAGAAGGAACCCAGGGAUGCUGUAGGUACGGGGAGUGG

GGGAGGGGUGGUGACAGCCUCAGCCUUGUGUGUUGAGGACCAAGGCGAGGUGAGCAUUCC

ACAUACCUUAGUUCAUUGUGUCCUUCCCAUAGCCUUGUAAAGUCGGCACAGAGUCACCCU

CUCCUGAUAAGGCACAGAGAGGGUAGGCAGCUUGCUCAAAGACACACAGCUGCAGGCCUG

CAGUAUUACCAACUGAAUGGCAUUUCCAAGGCAGUUGCUUCUUUGCUCAAGGUCAUGCUU

AGCAUGUUAAGGGGAUCAUCCUGUUUCAUCCCCAAAAGUCCCUCUACUAUCCCUAUCUUC

CGAGGCGGGUCCUGUUUUCUGGGGAUCUGGCUCAGAACUGGCAGGGGCCCGUUUCCCAGG

CUGAGAGAGUGCGCCUCAUUUAGCCCAGAGCAGAGGCCCAGAGAUGCCCACACUUCUGAA

GGUUGGGGGUCAGUUUCCAGUGUGGCUUAGAAGUUAGAGGGCAGGAGGGGCACCCCAGAG

UGGAGGAACUGCCCUCUUCCUCCGGAGUCGUUCAUUUGCAUGACAAUGAGCCCUUUGUUC

CUGAGGCCAGCCUCCUUCCCAGGCUAAUAACAUAAUUCCUGGCCUGUGAUGUCAUAAUGG

GCCCUUUCUGUGGCUGGGGUCAGAGGCUGGGUGGGCAGCUGUGUGGGCUGGGCCAGGCCU

GCUAAUAACCAGGGAGGUGGUGAUUGCCAGGGGCCUGUGUGCCAAGCUGCCUCCUUCCAG

GGUCUCGCUGAGUCCUCCCGAGAGAGCAGCGGGAGGUGGGAUUUGCUCCCCUGUUGACAC

UGGAGCAAGCUGAGCCUCCAGGAAGCCCUCCCUGGCCCCACCGUUUGUCUGGUCCCCCUC

GAUGAGCUGCUUCAAGCUUCUUGGCCUGUGGAGUGGGGGUGACUCACUGUCAGUUUGGCC

CUCGGCCGAGAGGCGUGGGCGUGAGAGUCCCUUGUUUCCUGUAAACUGGAGGGAGGCCAA

UACCCAGCGGCUACUGUGUUGGGGCCACUCGGAAAGCCAGGUUCAAACCUUGGCCCCACU

UGGUCCCCUGGGCAUCGGCUGAACUCCCUGUGCCUCCGUUUCCCCCUCUGCUUCAUGGGG

UUCCGCUGGGGCUUGGGCUCAGAGCUAGGCAGGGAGUGAGCUUUCAGGAAAUGGGCAAUG

UCAGAAAAGGCGGGGGCUGGUGGAGUGGGGUGAGUGGUGAUACCCCCAAGAGUUAGGUCC

AUGUCCUAGUAACCCAGCCCCUGUCAGAGAGAGACCGUAUUUGAUAAAAGGGUCUUUGCA

GAUGUGUUUUGAGAUGAGCUCAUCCUGGGUUACCCAGGUGGGCCCUGAAUCCAAAGACCA

GUGUCCUUAGAAGAGACAGAAGAGGAGAGAGCAAGACUACAUGAACUACAGGAAGACAAA

GGCAGAGAUUGGAGGGCUGAAGCCGCAAGCCAAGGAAUGCCUGGAGCCACCAGCAUCUGG

AAGAGGCAGAAAGGAGCCUCCCUUUGAGCUUCUGGAGGGAGUAGGGUCCUGCUGACGCCU

UGAUUUUGGACUUCCAGGCUCCAGAACUGUUAGAGAAUAAAUUGCUGUCGUUGGCCGGGC

GAGGUGGCUCAUGCCUAUAGUCCCAGCACUUUGGGAGGCCGAGGUGGGCGCAUCACUUGA

GCCCAGGAUUUCAAGAUCAGCCUGGGCAACAUGGCGAGACCCUGUCUCUACAAAAAGCAC

AAAAAUUAGCCGGAUGUGGUGGCGGCUUGCCUGUAGUCCCACGCUGAGGCAGGAGGAUCA

AUUGAGCCCAGGAGGUCAAGGCUGCAGUAAGCCAUGAUUGUGCCACUGCACUCCACCCUG

GACCACAGAGUGAGACCCUGUCAAAAAAUAAUAAAAAAAUAAAAAUAAAUCACUGUUGUU

UUAAGUUUCCUGGUUUGUGGUCAUUUUUAUGGCAGCCCUAGGAAAUGAACACAGCUGGCG

ACGCCAGUCUCUGGAGGUGGGGGAACUGGCCCUGGCGGGGGUUGGUACUGUUCCCCACUC

CAAGCCUGAGGGUGCAGGACAAGUUUGGGUGGCCGAGAACCCCUUUGUGCCUGGGAGUGC

AUGAAGCCCCAUUCGGGGAGUCUGGACUUUCCAGGAAGGACGCCUUGCUUGAACCUGCCU

UUCUUGCCGAGAACCAAAUCCACAAGGCCCUAGACCCCACCUGCCGCCUGCUGUUCGCUG

UCCCCUCAGACAGCCCUGCUAAGGGGACAGGACCCAGAGGCAGGAGGCUCUCGGAUUCUA

CCCCGGCUUUGCCCCAGGCCCCGGACGCCCUUUCCCCACGCGUCUGGUGACUCCUGCAGA

ACCCGGGUUGCACUCAUCCCCCAGCAAGCCCCAGGUUUGCCGGGUGCCCUUGACAAAUGG

AGAAACUAGGCCCAGAGAGGGCAGGCUACUUGGCAGAGGCCACACAGCUCCUUAGUGGCA

CUGACGGCCUUACAGUCUCGCCACGGGGGCUGGCACUGGGGCUUCCAGGAGUGGGGAUCA

GAGUUCUCGGCCACUUGGAGACCUGGAGUGGAGCCGGCCGGCUGUGUGGACCUGGGGUCA

GUUACCUGAUAUUUGGGACACUCGGCUGCCUUUGAUGGACCUGGCCUGGUGUAGGUGGUA

CCCCGAAAUCUGGGCUUUAAGCACACCCUGUCUGUGCUCUCAGCCUCAGUCUCUCUGUUU

GCAAACUGGUCUUUGAGCUUCAGCUCCACGUCUGCGAUUUUGCUCUUAGCAUGUUCAUGC

AUUCAGCAAACAUUUAUUGAGCACCUGCCAUGUGCAGUGUGCUGGGACUCUGGGGGACAC

AAAGGUGUGACUCUUGCUUCAUGGAUCUUGUGUUUGGGGCUGUGGGGGGAGAGGAAAUGC

AGCAGCACAGGUUGGAGUCCAGAUUCCGGGUUCUGAUGAGGGAGGUCACGGAGCUGGGCC

GUGAGGGAGGUGAGGAUGUGGCCUUUCGUCUCCUCCUGGGCCCGGGGUGGACAGUGUGCA

GCAGGCUCUCAAGUGGACAUGGAGAUGCCCACAGCCUGUCCGCGGCGGCCCAUGGGCACC

UCUGGCUGGCAGGACCUGGCCUGGUCUUGUGAGCAGGGACAUGGGAUGGGAGGGCUGCAA

GGCCUGGCUCCAGCCUGGUGGGAGAGACAACAGUCUCCUCUAACCUAAGCAGCAGGCAUG

GGUAUGCUGGUGACAGGGUCCCAGGACACAGUGGGUGAGCUGGACACAGGAGGUGGCUCG

CCUGGGUUUGCAUCCAGGCUGUGCCCUUGUUGGCUGUGUGACCUUGGGCAAGUUGCUCGG

CCUCUCUGUGCCAGUCUCUCCAGCUGUAGAAUGGGGAUGAUAACAGAAUCCCUCACGGGG

GCGCUGUGAGGAAGGAGUUGGUGCCACCAAGUGUUGUGUUGGGGACUGGCUGUGCCAGUG

GCUGCGAGGUGUUCCCUCCCGUGGCUGCUCAGGGCAGCGGCGUGGAAGAUGGAAGUGGCA

UUUGCUCAUCUUCCCGCUGCCCUGCUCCUCCCUGUGCAAAUAGCCUCAGUCUUCCACCUA

GGAAACGGGAGCUGUAACAAGCACAUGGCCCCAUGGAGGUCGGGAGGAGAAGCGGAGCUG

GGGUGCAUCAGUGCCUGUGGGGUGGGGGCUUGGGGUCGCCACCUGCUCCAGAUUGAGGCC

UUGGCUGCCCCCGAGGCCUGGAACAGCCACUUGUUUUACAACCCAGGAAACUGAAGGCCU

GGCAGGGAUGGCCCCAAGACAUGCCCAUGGGUACAGCCCAUGUGGUGGCUCCUGGCUGCA

GACAAUGCCUCUUUUUUCUGGCCUGGCGGGAGCAGCCCAGGCUUGCUCAACCUGGCCUAG

GUUUAUCAGCACAUUUGGUUCUGCGGUGCGGCUAGCAGACAUUCCCUCCAAGAGAGAUCG

UGAUGUUCCUCCGGAAACCGAGGUCCGGAGGCUCAGGUUACUGUGGGGGGCGGCCGUCGG

GGGGUGUGUCUGCCUCUGAGAAGUGACCCAGAUCAGAGCUGGGCUGUGCUGGGCAGUGUU

CAUCUGCAAAGCUGCCGCCUGCUCUUUCACCCAUGAGAGCCACUGACAGCUCAGGAAGGG

ACUGGGAGCCACCCACUUUCCCUUCAGGGUGACACAGGACAGGCCUCUCCCUGGCUCCUG

CCUGGCCCCUUCCCCCUACACUGAGUCUGGCCUGGGUCUCCCAUUCCAUUUCUCUGGCCU

CUUCUUGGCUGGCUUUGGACCUGUCCUCACCUACAUGGAUAAGGGACCUAGCGGGUGGAA

GAGAUCUGAUUCCAGGGAGAUCCUAUCUGAGAGUUUGUGUUUUAGGAGGGUUGCUAUCUU

AGGUCUCUUGGUUGCAAUGGACAGACUCCCUCUGCCUCCUGCAUUUAGCACAAAUAGUUC

AAGUAACUAAAAAACCUCAGAGAAGCUUCAGGCACAGUUGGGUCCAGGUGCUCCCCACUU

UCAGUCCCUGUUAGCCUGAGACUAAUAAUUGCAGUUCAGUAGCCCCAGUGGAAGGACAGC

UUCUUGUUCCCAAUAUUCCAGCAAAUAUCAUGGGAAUGAGUUUUGUUGCCUCAGCUUGAG

AGUUCAGAGUUAAGCCCCCCCCCCCCCCACCAAAGCCAGUGCCACUUUGGCUGGAGGGCU

GGGUCAGCCUCCCUGAGACCCCAGGACUGAGGGUGCAGGGGGGGUGGUUGCCCAGGGAAA

UUGGGGUGUGGCAUGCACGGAGUGGGGCCGGGUGGGCAGAGACACUGUGUCUUGCUCCCU

GUGUUGGAGAUGGGGCCAGGUUGGGGUCCCUGCCUGGCCUGCCUUUCAUGGCCAGUGUGU

GUGUGUCCCAUUGGGGUGGAGCAGGUGGCGCCGAUGAGUCACUGGGUAAGUUUCAUUCCU

GUUAUAUUUAAUAGUUGAGGGCCUCUUGGGUCCCCCCUCCAAGCCGUUGCUGGCGCUCCA

CCUCCUGUCCCCCCAUCAGCCCGCUUCAGCUCCCCAGCCUUCCCUCACCUCUCUUGUGGC

GUCCAGUGACCACGUCAUAAGCCACAGGUCAUGUCCUGGCAGCCCCUGGGCUGUAUUUGG

CUGUCAGACUGAUUUAUUGGGUUGGUUUUAUAGUCAGUGUUUUUUUAGAAAUAUCGGGAG

AUGUCUCAAAAUUGGGCUUCUUGGCUUUGCUGGAAAGAUUUGGAUGCCGUGGACCCCCUG

CCCCACGUGGACCCUGUUCCAGCAGGGUCUGGAACAUCAGGAAUGUUUGGGGGCCACAUU

CCUGGGUCUCUGCUCAGUGUUGGCUGGGGCUGAGCUGCGGUCACCCCCUUCCUGCGGGCC

CCUCCCCUCGGCGUGGUGCCUGCCUGGGCCCUUGUGUUCCGGCAGGGUCUUGGGUAUCUA

CAGCUCUCCUCUUGCUUUUUGUCCCUGCCCUUCUUCGGGUGGAUCAUCCGAUCCCAGGUA

GACGCUUAGCUUCAGCCGUCGCCACUCCUGGGCGCUGAAGCAUUUGUUUACUGUCUGAGC

UCCUGGCUGCAGGGGCCAUAGGACCUUUUGGGUUCUUGAUCACUGUCCCGUCAUCAGACA

UUUAUUGAUCAAUGCCCCUGGGGUAAUUGAAUGGCCCAGAUGCGUCCACUUCUGAGCAUU

GGACAGAGCUGACGUAAAGUGGAUUCUUCCCGUUUAGCCUGGGAGCCCAGGCGGCAGUGG

GGUCCUCCCAUCCAGGCCCUUUGUGGUCCAGCCUGGCCACUUGCUGGGGGCUGGGAUGGG

UUGGGGAGGCAGGAGGGGACCCUUGUUGAGUGGGUGGGCUCUGUCGCCCUGGACGCUGCU

GCUGGCACAGCUAUUCUUAGCAGCAGAAACAUUUGUCCUCAUUCACAUGUGGUGGAUGCC

UCCAGGGAUCUACCCUGAAAUACUAGAGGCGACCCCAUAAAUUCCUCAGAAAGCCAUCCU

UGAGGGCCUGCGUGCAUGGAGGAGAAGCGGGAGGAGGCGUUUCCGGCAGCUGCACAGCUG

UGGAUGUGUUUGCCAGAUGUUGGAGGCAGGGAAGUCGGUGGGAACCUUGCGGAGGCCGCC

UUCACACUCGGGGUCUGGGCAUGGACAUGAGCGGGUGGGACAGAGACUGCAGACUGGUGG

GCAGAGAGAAGGGCUCUGGCCCUGGCCUCAAGUUUUGGGGUCCAGGGGCCCCUCUGAAGC

AGUCAGCCCUUCCCUGGGCCCUGGCCUUGAGGCCACCUGGGUUGGAGGCAGCUGGCGAGG

GGAGAGCUCAGCUUAUCGGGCGCUUCCGAGGGAUCAGAGGUGCAAGUCGCUGCCCAGCCA

UGCCCUGUGGCCGUCAUGGGGUCCUGAGGUGAGGUGGGCCAGAGACAAGGCCUGGACGCA

UGCUGCACUCGGCUCUGAGGGCCUGCUGAGUGCCGGGGCCACUGGCAACACUUUGCCUUU

UAUGGGGGCAGCCUGAGCCUUUGGGGCCACCGCGGAACAACACAAGCACAACACUAGCAA

UCAGGGAGGUCUCCCUGGAGGAGGUGUUGUUUAAUUUUAAAUUGUGGUAGAUACAUAUAA

UGUGAAAUUUACCUUCAGCCAUUGGUAAGUAGAUAAUUCAGUAGUGUUAAGAAUAGCCAU

AUUGUUUAGCAACCGAUCUCCAGCACUUUUCAUGUGGCAAAACUGAAUCGCUAUAUCUGU

UAAACAGUAACUCCCCCUUCCUCGCUCUCCCCAGCCCCUGGUCCCAGCCAUUUUCCUUUU

UGUCUCUAUGAAUUCAGUGACUCUGGCCACCUCCUACGAGUGGAAUCGCACAGUGUUUGU

CCUUUUGUGGCUGGCUUCUGUCACUCAGCAUAAUGUCAGGAGCUGUGUUCCAUGUAGUCU

GGGGUUGUUGGUUCAUUCUUAGCCAUGAAGCAAAACGGAUGAAGACCCAGGACCCCAACC

CCGUCCGCAUCCUUUAGAAACCCCAGGUCCCACCGCCUCUCCAGGUGCUGUCCAGGAGCU

CAGCCCCAGCUCAGCCACCCAGCUCCCUGAGCCCCACUCUGGCAUGGCCUCCCUCUCUCC

UGCCGUCACAGCCCAGGGGCACGGGGUUAAGUGGUUUGCCCAGGUUACACAGCUGGUGCC

AGCGUCUCCCUCCCCACUGCCCAGACACCAGCCUCCUCUCUGACUCCUCACUUCCCUCUG

CAGAGAAGAUUGCUGUUGGGAAGGCCAGAUGGAGGAGAUGGGUCUAAUGGAAAACACGUC

CCUCCUGGGGCGCCCGGAACUUAAUGAGGAAGAGGCGGCAGAACCAGGCGUAAUGUUUGU

GAAGUACGCAAGUGGGGAGGGCCGCUCCCCGUGCAGGGCUUCUCGGGUGGAGCCACAGGA

ACCUGGAGUGCAGGGAGGGUUCGUCCUGCUGUGGUUUAUCUCCCUCUCUUCCUUUUAAAA

UUAAAUGGAAACAAAAAAAAAAGGAGGGGAGGAGGAAGUGAUUUUGAGCAUACUUCGCCG

UGGCGGGGGAGCAGGGCCGAGCUGGCUGCCGUCCAGCGGACAGGAACGCUCCAAUUAUAU

UGGAAACAGUAACGGGCCUCCUCGGAGGCUCCGACCAACGCCACUUCCCUUUCUGUUCAC

UGAGGGCGGGCGCCUGGGCUGGCAGGGCCGUGGGGAGGGCCGGGGGUUUGGGCACCCGUA

UCCUGACUGUGUGGCCUUGGGCAAGUCAGUUCACCUUUCCGAGCCUCAGUUUUCUGAUCU

GUUCAGUGGGGCGAAUUGUGUUUGGUGAAUGGAACUGGAGCGUCUGCUGCCUCUGGUGUU

AGCAUGAGAUAAAUGUGGCCUGGAGUGGAGGUGGGGCCUGCUUUGCCUGGGGUGAAGGGG

UGCAGGAAUGGGGAAGCUGGGGGGCUUGAAGCAGAAUGUGGGGAAUGAUAGAGGCUUUCC

CAGUGGCCGAGGAGCCUCUCUAGAGGAGGGGAGAGAUGGGGCUGGUGCUGGGGGUGGGUU

UUGCACCUGGCCUAUUUUCCCCUGGAAGCAGGAGCAGGACGUGGCACUGGGGAGACAGGA

AUAAUGGGCUGGGGCAUUUGGGCUCACUUGAGAAUCACAGGCUCCUCCCAGACUCAGUUU

GCCUAUCUGCAAGGCGAAGAUAGCCAUAGUGACUGGCUUGGUUAACAUCAUCGCCAUCAC

CUCCCUCCUUCCCCCGAGUGGUCGGCGUGGCAGAGGGCAGUAACAGCCACGCUCUCCUUC

UGGGGGCUCUUCUGUGGGCCGAGAACUGCACCGAGAUUCUUGUAGCUCUUUUUUCCAGUG

AAUCUUUCCAAGUGAAGCUCAGAGAGGUCAGGACCUCACCCAAGGUCACACAGCUGUGGA

AGUGGCGGAGUUGGGAUUUGGAAUUGAUCAGCACGUGGGAGCCCUUUGGAAUGGCAGUGU

GUCCACAGGAGGUGGCUUGGUUGAGAGGGCACUGGCUCGUUUCAGUGACCGAGGGGCUCA

GCCCACAGCCUCCAUAGCUCCUGCUCCUACAGGUGCAGCCCACAGCCUCCAUAGCUCCUG

CUCCUACAGGUGCGGCUAAAACUGCCGAGGAGGAGCCCUGCCUGGUGGGGGAGACAGCGG

UAAACAGGUGGCUGUGGCACAGGGGGCCGAGUCCAUGGGAGUGGCAGGGGGAGCGGGCCA

UGGCACCGGGGGGCCCCCUUGGGGUUGGGGGAAGGUUCCAAGGAGGUGCCAGUAGAGGCA

GGAGCUGAAGGCUCAGGAGAAGACAGACUGAACCUGCCCCAUUGAGUCCAACCAGCCAGG

CAGGGCAUGUAAACAAAGGUAGCUGAGUAGGAGGGGACGUGGCAAAUAGGGAAAGCCGAG

CCUAUCUGGUGUAGAUAGCCUCUGCCCACUUCCAGCCGGCCAUGGCUGGAACCUCAGUUU

CCCCUGUCAAACGAUUUUGUGGUAAAAUACGUACAAAAUAUACCAUUUUGGCUAUACUAA

AGUGAACAAUUAGUUUGCAUUUAGGACAUUCACGGGUUGUGCAACCACCACCUCUGUCUA

AUUCCAAAACAGUUUCAUCCUCCCAGAAGGAAACCCCAAACCCAUUAAACCGUCACUCCC

CUCUCCUCCCUCCCCACAACCCCUGGCAGCCUCGGGCCUUUGUUCUGUCCUGGGAAUCUG

CCCCUCCUGUGAAGUUAAUGGAAUCAUGCAGUGCGUGGCCUUUCUGAGCGGCUUCUUUGC

CUUUUGUGCCAUGUCUUCAGGGCUCACCUGCGUUGUGGCUGAGUCAGCGCUUACCUUUGC

GUGGCUGAAUCGUAGUCCAUUGAUGGACAGACUGCGUUUGUUUCAUCUGUUUGUCAGUAG

AUGGACAUUUGGGUUGUUUCUGCUUGUUGGCUGAUGUAAAUAGUGCUGCUGUGGGCAUUC

UAAUCCUUUCUGUUUGUUUUGUCACCAGUUUUCCGUUCCUUGGGGAUGUGUAGCUAGGAG

UGGCAUCGCUGGGUCAUGGGGUAAUUCUGGGUUUAACCUAGUGACUGUUUUCCACAGUGG

CUGCACUGCACAUCUGACAUUCCCACCCGCAAUGUGUGAGCAGCCCUCAUUAGGAGAAGC

CAGACAUUCCAAAUUUCCUGUGAAAUCCCUCUUUAUAAGAGCUGGUCUCUGGGGCUUAGC

UGAGCUGACCCUGGGGUGAGGAAGCAAGUCCCAGGAAACUGCCCAGAGGGAGCAAGUCUG

GGACCUGACGAGACGCUCCGGGCACCCCAGCUUUCUGGCUUUAACCUGGCCCACGUAUCU

CUACCCUGUACAUCUACCCGGGGUCAAGGAGGAGGAUUCCCAGGCUGGAGUCCUCUCGGC

CUUUCUCCUCGAACCCAGCUCAAGGGGAAGAUGUGCUGGUUUAUGCUGAAAAGGGAGAAG

UGGUGCCAGGUGGGGGACUCGGGAGAGUUGAGACCCUCCCACGCUGAGUCUGGGUUUGGG

ACUGGAGGCAGUUUUCCCUAGGGUCCUUCUUACUCAGCAGGCUGGGACUUGGGGAGCAGU

UAUAUGGGUUUGGGGGUUCCUGAAGGCGACAGUGCCUUUCUGUGUCCCUCAUGGAGCUGG

GCAUGUGGGAGGGCCUGGGAAUAUUAGCAGGAUGAAGACAUGCUUGGCCCUGGGGAUUGG

UUAGAAAUGUAAGACCAGAAAAACCCGGAAAAUGAAGCAGCUAAGUUGAUACCCAUCUGG

UGAUGAGUGUGUGCAUCCAUCCGCCCAUCCAUCCAUCCAUCCAUCCAUGCAUCCAUGCAU

CCAUCCGUCCAUCCAUCCAUCCAUUCGUCUGUCCAUCCAUCCAUCCAUCCAUCCAUCCAU

CCAUCCAUCCAUUCACCCGUCCAUCCAUCCAUCCAUCCAUCCAUUCGCCCAUCCAUCCAU

CCAUCCAUCCGUCCGUCCGUCCAUCCAUCCAUCCGUCCAUCCGUCCGUCUUCCAUCUAUC

CAUCCAUCCAUUCGUCCAUCCAUCCAUCCAUUCAUCUGUCCAUCCUCUGUCCAUCCAUCC

AUCCAUCCAUCAAUCCAUCAUCCAUCCAUCCAUCCAUCAGUCCAUGCAUCCUUCCUCCUU

UCUCUAUCCAUGCAUGCAUUCAUCCAUCCAUCCUCCUUUCCUCCCUCCCUUCCUCCUUUC

UCCUCCAUCCUCCCAUCUCUAUCCCUCUUCCAUCUUCUCCCUCCAUCCAGCCACCCCCUG

CUGAGCACCUGGGUGGGGAGUCUGCCAGUUGGGCCAGACCUGUCACUGGGUGUGGGCCGU

GGUCAGCAGGGCCUUUUGCCUUUUGUCUGACUUGGUCACUGCUGUAGCUGUGCUUGUACA

GUGUUUGCACAUAACAGAUGCUGAAAUAAUUCUCUAAUUGAUGAGAAUGGGUCAGCAACC

GGAGUUUGGGAUGGGUUUGGCUUGCCUGGUCUCAGCUGGAGGGCAGACCGCACCCUUGUG

GGUGGCACCCGGGAUUGAGCUCUUAGUAUUUGACCCUCAGCCCCUCCGGCAGAGCCAACG

ACAGGCUGGGGUUGGGUACCUGGCUCUUCCUUAGGGAGUGGAAUGACCUUUCUGCCAAGU

GAGGGCCAAGGAGUCUGGCCUCAUUCCUGCAGGCCUGCCGCUGUGCAUGGGUGGAGGCCC

GUUUCUCUAGGGCCCUGACAAUCGUGGCUGUCAUCAUUCAGGGUUACAGGGGCCCUUUUA

CCUGUGCCCCACACUCAGACACCUGUGCCCAGUGCUCCUCGACACAGGGGCAGCUACAGU

GUCCUUUGAACACAGAGACGCCAGAGGCAGACUUGCCACAGGAGACCGCACGUCAGCCUU

GUUUAAAGUGCACCAGCUGUGUCCUUCCUAGCUCCCUUGCAGUGAACACCCCAGCUUUCU

GGCUAUAGCCAGAAAGAGUGACCUCACAUGCCUUCGCUCACCCCACUCUCCCCAUCCAGG

CCUCCCUGCUGUCCUCACACACCAGGCUCGGUCCUGCCUCUGGGCCUUUGCACCUGCUGU

GCCUGCUGUGUAGACUCCCUCCUCUCCCCGGCGCCUUUGCAUAAAAGGCACCUCUGGUGA

CGCACCCCUGACUGCCCACUUUCAAAAUGUACCCCCAUUCCCUGCCUGCCGCCUCCUGGU

UCUGUUUUUCUCCAUCGUGCUAACACCUUCUGCCAUCCUCUUACUCCCGUCUCACCUGCU

GGAAUGGCAGCUUCACGAGGUGGGGGAUUUGGGAUCAGUAAUUCACUGCUGUAUCCCCAG

CACCUUUCACACAGUAGGUGCUUAACAAAUCGUGGUAGACAGAGUGAGCCACCAGGCCGG

GCAGCAGGAGCUCUCUCUUCACCCCCUUCCUCAUUGGCUGUGGGGGCCCCUCUGGACUGG

GCUGGGCGAGGUGCACCCUUGCCACGUCCCACCGGCUUUCCGCAUCUCAGGACUCACUCC

AGAGCUGGAUUCCUGAGCAUCCGUUCUGCGCCAGGAGUCCUGCUGAAGGUGGCCAUCAGA

UACUUGGCCAGUUCCCCAGUUUUUACUGCACGCCAGAAAGUAAACACCCAAGGUGUCAAC

AGCUCGACCCGUGGAGCUGGCGCUCCGCAGCGUCUUGUGCGCACAUCCCAGAACUGCUGU

CCCAGAGUUCCCCCCGGGCCGGCUUCUCCUCGGGCCAGACUGGCUGCAGUGGUUUUGUUU

AGUAAAUAUUUAUUAGAUGCCUACUGUGUGCUGGGCUCUGGGGAUACAGCAGUGAACAAA

ACAAGUAUUUCUGGAGCACCUCCUGAAGUAGGUAGUGUUCUCCCUUAAGGUACGUCUGCG

UGGAACCUGGGAGUGCGGCCUUGUUUGGAAAGGGUUUUUGCAGAUCACAUUAGUUAAGAU

GAGGUCACAACCGGAUAGGGGAGGGCCCCAAAUCCAAUAUGACGGGUGCCCUUAGAAGAA

GGGGAGACACAGAGACAGGAGAUGCAGUGGAAAGGCUGCGUGGAGAUAGAGGCAGAGGAU

GGCACGAUGCAUCUACAAGCCAGGGAACACCGGGGGUUGCCGGGGCCACCAGAGCUGGAG

GAGUGAGGAAGUAGGAAGGGCCUCCCCCAGAGCCCCCAGAGGCCGUGGGGCCCUGAUGAC

AGCUGGAUAUGGACUCGAGGCCUCCAGAACAGUGAGACAAUCCAUUUCUGUUGCUUGAAG

CCAUGCAGUCUGUGGUGGGGCAGCCGAGGGCGCUAAUACGCCUCGUGUGUGCCAGGUGCC

GUGCCUCUCGGUGGCUGUCACACAAUAGGUGCUUAUUCCACAGUGAGUGGAACAAAUACC

UACUGAGCGGUGAGGGAGGCCGUAUGUGGCUGAGCUGCAGGGCUCCUGGGGCCUGCCUGU

CUUCGCAGGGCCAGGCUGGGACAGUGGAGGCACGGCCUGUGUUCCCGCCGUGGUUCCGCC

CACCCCAGGGGGGCUUUGUGCCUUGGGCUUCUCAGAGUAUCAGAGGGUUCUGUCGAGUGU

GUGUGUGUGUGUGUGUGUGUGUGUGUGUGUGUGUGUGUGUGCGCGCGUGCGCGCGCGCUC

GUGCCGUGGGCGCUCUCUGCCUGGCAGGUUGGUUUUUAAUCCCGAGGCUCCCCCACCUGC

CACAGGUUCUUCCAAUGUCAGCACCCAUUAGUUCCCAGAACAUUUCAGGCACGUGCGGCG

CAGAGCCCCGCGUGCCUUUCAUCCUCCCCACAGCCCGCGGCUGAGCGCUCCGGGCACAUC

CUGUAGCUCCCACACCUCCACCAGCCCCCGCCUGCCCCUCACCCUGAGGCUGUAAUUCCA

CCCUCCCCAGGUCUGAACCAGGCCGCUCAAGACACAGAAAAUCUAUGGAAUGUGUCUCGG

UGUGGCCUCAUCCUGCCGGAGCUGGAGAGGAAUGCAGGGGUGGGAGGCCCCAUGGAAGCU

UCCAGGGCCUCCCGGAAAGGCGCCUCUGCCCGGCCAGGUGGGAAGAGGACACAGGUGUGG

UGGUUUCUGGCAUCACUGCGUUGCUGGCAUGCACCUCCGCUGGGCAGAGUCAUUCAGUGU

GCACCUACUGUGUGCCAGGCACCAGAAGGGGCUCUGGGUCCAGCAGGAGGCAAGGCGGAC

AGCCAGUCAGAGCUCACGGCCUGAGCUUUCCGCUGUGGAUUUGGAAACCUUACCAAAGCC

CGCAGUGGCCGGGAACAGGGAAUGUGGCCCACUUGGCUUCUGCUGCUGCCUGGCAGAGUG

CAGUGGGGGCGGGGGGCACUUGUGUGUUUGCUACCUCCUGGCAGGCAUGGCCCAGGGACA

GACCCCUGCAGCCGUGCAUGGGGAGCCCCUCCACCUGCCUGCGUGGGAUCUGCAGCCCUC

CUCCAGCUGGUUCAGGGGCCAGCAGGGGCCCUGGGGCUGUGGUUGGUGGCUUGGAUGUGC

AGAGGCCUGCUUGGGCCCUUGGUGUCCCCGUUGGGUAUCCCGGCCACCUGAGAUCUACAA

GUCCCUUGACCUCACCCAGGCACCGUCUUGUUCUCCUCGGAGGCCUGACCCUCCUGACUC

UGCAUGGCUGAGCACCUGCUGUUCCCCAGGCACCUCUCGGUAUGCCACAGGCACAGGGUC

CUCGCAGCGGGCCUGUGCAGCGGGACUCCUGCAGCGAGCUGUUUCCUCGGCCUGCAGAGU

CAGCCUCCCUCCUGACUUUCAUCCACCUGCCCAUUUCCCCUUCUCUGUCGCUGUUGCCCU

GAUCCCCCAGUGUCCCCUCUCUUGGACAUUUGCAGGAGCCUCCCCAGGUCUCCCUGAUCC

ACUCUUGCUGUCCCCAGUGCCUGCUCGUAAAACACUGACCCUUUACUCUCCCCCGCAACC

ACCUUCUGUGGCUCCCCACUGCCCUGAGAACAAAAUCCAUCCUUACCCGGGCCCAUGGGG

CCCUGAAUGACUCUGUCCCCACUUCCCCCCAUAUUCACUCAAUGAAUUUCUCAAACAUAA

ACGGCACCUGUGCUCACCCCCAGGCCCUCGCUCACCCCUGGGCCUGUGCUCACCGCCUGG

CGGGGGCUCACCCCCAAGCCUGCGCUCACCCCCCACCAGCUGGCGCUCACCUCCCUGCUC

UUGCUCAGCGGCGCCUCCAGCUGGAGCACUCUCCAUCCCCUCGCCUUCACCCUCUGGAAG

UUCUUCUUCCUCGUAUCUCCUGGCUCUCCCCAGGGCUGGGCAGAGCCCCUGGCCUGAGAC

CCUCCAGGGCCUGGACCCCGCUGGGUUCACAAGAAGUCCCCAGCACUAGCCCGGGGCUCA

GCAGGGACCACAGUGUGUAUGUAAAUAAAUGAAUGCCGCCCACCCUUUCUGACCCCACCG

CAGCCAGACGCAGCGGCGACAUGGGGCGUUUGUGUCUGAAUUCCCCCUCCAGCCCGGAUC

CAUCCUCAGAACCUCCUUUGGCCAGGACCAGAUCCUGGAGACCCUGGAGGGGCUCCCUCG

UCCUCUCCUGCCCCCAUCUUUGAGGAAAUGAUGUCAUCAUUUUUUUUGGCAUUCACUUCC

AAACCUGCUGAUGAAAAGUCAAAGCUUAUUUUUUUCCCUAUUCGCAGUGCUGCACACCCG

CCUCCUCUAUCAAGUUUCAAAACCUUUGCAUCACUGCCCUGUAAAACCCUGUAACCAUUA

GCAGGCCUCAUUCCUGCCUCACCCUGCCCUCCUGGUAGCCACCAGUCUCCUUUCUGCCUC

GUGGAUGUGCCUGUUCUGGACAUUUCAUAUCCGUGGAGUCAGGCAGUUCGUGGCCUUUUG

UGUCUGGCCUCUUUGGUUCAGCAUGACAUUUUCUGGUUAUGUGUGGUGUAGGACACAGCC

GUCCUUCGUUCCUCUUUAUGGCUGAGUAAUACUCCAUCGUAGGGAUGGACUGGAAUUUUU

GCUGGGUCUGCCUUUUGGCUGCUGUAAAUUCUGUUGCCAUGAACAUGCAUACCGUGUUUU

GUUUGAGUCCCUGUUUCCAGUUCUUUUGGGUGGAUGCCUGGGAAUGGAAUUGGUGGGUCA

UAUGGUUAUUCUAUGUUUAAAUUUUUGAGGAACCGCCAAACCGUUUCCCUAACAUUUGUU

UUUUUUCGAGUUGGCAGGAAACGUGGCUCAGGUCGGGAGGGGUUGGAAACUGGGUUUCCU

AAGCUCGUGGUGAGUGAGGGUGGCACGAGGUUACUGGAGUUGUUCCCUGUUCAGGCCCCA

CCGGCAUGUGAGGCGCUGGGUGGGGGGCUGGUGGGGGAACCCCUUGGUAGGUGAGGGGCC

GAGGUUCCAGCCCCAUGCUCCGGGGUGGGCACUGUGUAAAUCCCAGGCAGUGGAAAGCCA

GCCAGCCAGCUGCGGGGCACUUUAGGACGGUGUCACCCUCCCUGGUCUCUGGUGCCCGUG

UGCACCAUGGUAAGCGUGUGCCAGGGGCCUGUCACCCCCAGGCAGCAUCUCACUGGGCAA

AGGAGGAAAGAAAAGCUGUGGGUAAAUGAGACCUCUCGGCCACCUUGUGCAACUCAAAAU

CCAAUAGCAAUUUGGAAGUUGCCCGUGGUGCCCUUGAAAGAGGGGCAGACCUCGGGAACA

GCACCCUGCAGAAGUGACGGCCUUAUCUCCUCCGAGAGACCGGGAACUGUUUAGCACCUA

GAGAGAAGGGAAUAGAAUUUGCUUUGCUGCAUGAGAUGACCAGGGUCUGCAGACUUUGAG

CCGAGCUCUAGCAGGAAGAAUUUUAGAACAGGGCUGGGUCCUGGUGGCCACAGCUGUUCU

CUCUCCCAGGGCCAGACUCUUGUUUCUGGCUGCAGAACGGAUGUCUCAUUGCUCUUGGGG

CCGGGCAGGGCUAGCUGUGCCUUGCCUCCCCAUCAUAGCGUCACUGGUGACUGGGCUGCA

GCUGCAUCAUUGACAGUCCUGGUGACAGCAGUAUUAGGCAUUCUGGUUUGCUGAGCAUUU

ACUGUGUGCUGGGAGCUGCUGAGUGCCUGCUGUCCGCCACCCGGCCUUUCCUGGGGUCCU

UGCAGGAGGACUUAGGCAGUCGGAAUAUGCACAGACGUCUUCAUGUGUGUGGGAGUGCGU

UUGUGAUUCCGAUGCCUUCCUAUUUAUUCCACAUAGUACUGGGUUUCUGUAUACCUAUGC

UGAUAAUAAGCUUCUCUUGUAUGUAAAGUUAUGCCUAACUGAAUGAAAGUGACAGGCCCG

GGGCUAGACGGCUGUCGCCUGGGUAAUCUCUAUAAAAAAGUCCCUACGCGGGGGUGGCAC

AUGAAUGUCUGAUGUUUAGGGAACACUGUGUGUUUGGAGGUUCAGAGAGGGCAAGUGACU

UGCCCAAGGCCACGCAGCUUAGCGAUGGUGGAGCGGGACGUGUUCCCAUGUUCACGGGAA

UUGCGCCAUACAUCCUGUCUCGGGGCUGGGAAAGCCCACGCAGGCAGGACCGAGGUGGUG

GUAGAUGUGUCAGUCUUGGCUCCGGAAUUCCUUGGCUUUUCUGUGUUUGAAGGAUGGACA

GCAGCCAGUUGAAGGGGUGGCCUCCUCUGUGACUCCUUCUUUCCCUGUGUGAGCCCGAAU

UCUUGGUGGCAUGUGACAGAAACCCAGCUUGGGCUGCCAAAGACAAAACGUGCAGAAGGA

GUGCCCUGGCUCCCCUAAUGAAGUGAGCAGGCUUCGACCUGCCUCCAGCCACCAUCGGAC

ACAACCUGCUCUUACCGCCCUUUCGCCCUGCCUUCCUGCCGGUGGGCGUGGGUCUCAGGC

AGGCUCCUGCUGCAUGGUGGCCCCCAGAGCUCCAGGCUUUCAUUCCCAGCUUAGAUCCCC

AGCAGAGAGCACCUUUCUCCUGGUUCCAGCAGCAAAAGUCUCAAGGCAGAUUCUCAUUGA

CUCCUAUUGGGUCACAGGCUCAUCCUUGAGACAGUCACUGGCCCAGAAGCAUGCAGGACU

CUGAUUGGCCAGGACCGGGUCACAUGACCAUCCCUGGCACCUGGGGAGGGAGGGAGGGUC

AAGUCUACACUAAUGAGAGAUGAGGUGGGGCCUGGGAGGCAGUCAGGGCCCCCAACCUUA

ACCUUCCUCCCAAUGCCCCUCCACAUUAGGCCUCCAAGGGCUUUCCUGCUUGGAGAAGGA

CGGUUACCCUCUUCAGGUUGGGGGAGGGGGUGUUCUGAUGAAAUUGCAGGGCUGGGUAUG

CUCUGUUUCCCCUCCUGACCCCUGACCUCGUGUGGUGCAGCCUCAGUCCUCCGCCACUGG

AGGCCCACAUCCAGGCGCUGGGUCCCGGUGCCAGGGUGCAUUUCCCUCAGCUCUGGGGGG

UGUGUGCAAUUGUGGGGGUGCUGGAGAGGGCUUUGCAUGAGGCAGGACCUGAUGGACACA

CCUUUUCUGAGGGACUGGUUCAGGGAGAGUGUGGAGGGGGCCUUGGUCUGGGAUGGGACC

CUGUUGUAAGCCCAGCAGGUGGCGUGGAGCUGUGGCUGGCGGAGGAAGGGGAGAAGCCUU

GUUGGGGGUGGGGGGUGCUUAGGGGUUUCCUGGCAAAGGCAGCUUCGGAGUAGGGGUCCC

CGUGGCUCCCUUGCUGGGCCGUGUCUUCUCUGUGGGGCUGUUUUUCCAUCUGUAAAGCUG

CUAGCCCGCUUCGUGUUAUUCCUCUGGCGUGGCCACGAGUAGAUCCUCUCCCGUGGAAGG

CUCCAGUAUCUGCCCCAGGAGCCGUUUGGGGUACCUAUCCCUAGGCUGCAGGGCCCGACU

GGCUGUCAUUCUGCCCUUGGCUCUUCCUGGUGUAGCCCUCCAUGUCCUAAUCCCACCUCC

UAGUUUGCAUCUGGCUAACCUCAUAUCAGUCCUGUCCCAUGUCAUGGUGUCAGCCUCCUG

CACAGGGUCAUAGCCUUGCCCAGGUCAUGCUGUGGCCUGUGGCCCGUGGCCCGCCUGAGC

UGGCCCUGGUCCCUGCUGCCCGGGACAGCGGUGUGUGAUGUCCCUGCUGCCGGCCGCCCC

UCCUGGGACAGGUGCUUUCUGGGAAUUCUGCCCUGUGACUUAGAAACCCGGGGGCCAUGG

AGUGAAGUUGGAUGGGCUUAGGGGGGCUGGGAGCCAUCUGGGUGGUGGUGAGGGCAGCGC

AGAGUCCCUGUGCCUGCCGGGUGAAGGCCUGGGUGGGGAGGAGGCCUCUGGUCCCUUGCG

UGGCUUGCAUGGCUCGGAGGGACCUUGAAUGCCAUGCCUGUCUCCUUGUGCUCCCGGAGA

ACAGGUGAGUGUGGCAGUGGAUGGAGGAGGGUCCAGGCAGGCCCCUGGGUGCUCGCGUGU

CCUCACGGGACCACAGGAACGACUCGGGGACCUGUGCACGGAGGAGCCAGCUGCCCCGUG

GCUGAUCUUGUUUUUCUUUUCUUGUUUUCCCGCAGGUGCCAGUGACGGGGUGGCCCGUGA

GCUGAUGACGAGGACUGGCUUUUAAUCCUUGGUGGUGAUUAAGAGAAAGCUUAUUGGGGC

CUGGGAGCAGCUCCCCGCCGACCCCCACCACCAUGUCGGGAUCCACACAGCCUGUGGCAC

AGACGUGGAGGGCCACUGAGCCCCGCUACCCGCCCCACAGCCUUUCCUACCCAGUGCAGA

UCGCCCGGACGCACACGGUAAGGGGGUGCACAUGUGUGCGCCUGGGGCUACCUUUGAGAC

CCUUCUUCCAUUCCACUGAGUCUUCACCCCUUCCUUUCUCCUGGGAGCCAGGCUCAUGUG

GCUCUGAGCCUCCCAGGGCAGAGGGGAGGCCGCCACAGAGACCCCUGGUGUCUGAAGCCU

UUUCCAGAAUAUCUGAUAAAAUCUCUUUGCUCCCAUUUUAAAGCUGGGAAGAUGGAGGCU

GGGGCCAGGCUGAGCUGGGGCUCUGCCCUCUCUGGACUGGUAGUGGGAUUGGCACGCGAA

UGUGGAGCUCCUGCUGUAUUCACUUUCCUGCAGCCUAGCCUCACCUCUGCUCCCCCGCUG

CCGCAGUAUUGGGUAGGAUUGGGGUGGCUUCCAGGGGGCAAGGGGGCCGGAUAGGUCACA

GCUCCCCUUCCUUCCUGUCCUCCCGUUCCGUGUGUAUUCACCUUGGUGCGGCUCGAGGCU

CUGAGGUGAGGAGGCCCCACACAGGUUGCGUUUCUGCCUCCCUGACAUGAGGGUCCAGGG

UGCUGGCUGUGUCUUUGGGGAGCCCGCCCUGUGGCCAGGGCAGAUGGCUUGCCCUGGGAG

CAGGUGGGCCUCGCUGCCUGCCCACAUGUGUGCCCUGGUCGGGUCUCCUAAGCCGCACCC

CACCCCACCCCACUUCCCAGCGGGGGCCCCAUUUCCUGCCUCUUCUCUCACUGCAUGGUG

UGGCCAGGACAACGGGCCUUGGUUUCGCUCCUUUGGCAUUUAUGGGGUGCUUGCUGUAUA

CCAUGUCUCUGGGUGUUUGCCAAGCGGCUUUCAGAGUUGUGGGCCUCCCCCUUGUGACCU

CCUGGCUGCUGAGUGAGGGGAUGUGAGGCCUGGAGGUCACAGCUGGAGUGAAGCUGACCC

UGCCGGCCACCGUGGGGGUGCGUGUGUGCGUGUGUGUGUGUGUCUGUCCUGCCUGGCUGG

UGUGGUCCCCUCUUGUGAGAACCAGAAAGGAAGCUGUGUGGGAGGACGUUUCCAUCAGUG

AGCAGAAGGUUUUGCAUAUAAGCUCACUGGCCCUGCCUGGGGGAGUCCCUGACACAGUGG

CCUCCUGUAGGUUGGGCCCCUGGGGCGCUUUGCAGAGUCAGCUGUCCUCAGUGGGUGCGU

UGUGGAUCCCUCUUAGGACGGCAGUUCCCUGGGCAGAGUCAUUGCAUUCUCCUGGGCGGC

CCUGUGAAAGAGCAGCUCACGCUCUCAUCCCACUUCGCAGAUGAGGAGACUGAGGCCCAA

GAGGUUAAAUCACAGGCCUCAGGUGCGAUUGCCCUAAGUGGGGAGGCUGCCACAGCAUCG

GGGCUCCAAGCUUCUGUGUCCCCCCACUCCCCCGUGCCUCUAUUUAUUGAGCACCUACUG

CGUGCCUGGGGCUAAGGAAUAGACUCCCAGUGGGAAAUGAUUUUAUAUUUUGUCAUCAUU

GUCUUUAUCUGUCACCACCACCAACAAAAAGAAGAGCUCUUGUUAACUGAGCACCUACUA

UGGGUCAAAUGUUGUUCAAAGCCCUGAGUGUAUAUGGUUGUGUGCAUUCCCCUGAUCACC

CCAGAGUGGGACUAUCAUGCUCUGUAUUUUGUCAAUGGGAAAAUAUUUCACAGGGUAGGA

AACUGGACAAGUUACGUAACCUCUCUGAGCCUCAGUUUUGUCACCCAUAAAGUGGGGGUA

CUGAGAGUACCUGUCUCCUAGAUUCUCACGAGGGGUAAAGGGGAUAUGCCGAAUGUUCUU

AAGAUGGCGCCUGGCUCAGGGCCAGCCCGCUCUGUCCGUUUGCUGCUGCCGUUCCUUGGU

CUUGUUGCUGUACCUGCAGCUGCCCAGAGGGGAACCUGCCUGCCCUGGGGGUGCUGCUGG

CUUCUGGUUUCUUGUCCUGUCCUGUGGAGGUUCCCACUCUUCUCAACCGCCCUCACCCGG

CUCCUAGCCUUGGGGAGUCCUCAGGCUGCUGUGGCCUCUGCUCUCCCACCCCUCCUGCCC

UUUGCCCCAACUGGGCCCUCGGGGUCACUGGCUGACCCUAGACACCUCCCAUUCUUCUCC

UGCUGGGCCGUCAGUGACGCAGGAUGUGGGUGGACAGGGCCAAGCGCUCACCGCCCCCUC

CAGGCAGGAGGCUGAGGCCAGGGAGUGAGUGGGGCCCAGCCUGGCGGCCACAGGUGAGAA

CAUGUCUGUCCUUGUGUGGGGGCAGCAGGGGCCACCUGUGUGUGCUCCUCCCUGGGGGAG

CUGCCCCAUAUCGGACCCUCCUGCCUCAUGGUAGGGCUGCCCAUCCAGAGAAUCCUGCUU

GUCUCUUGAAGGGCAGGAGGAAGAGUUCUGGGCACUCUGAGGAGGGCCCUGCUGCCCUGG

GCAUAUUGCAGGGUGGAGCCGCUUUCUUGCUGUGGGUUGGAGGUGAGCUGGGGGCUGUGA

UCUGGAGGCCCCUCCCUGGAAGCCCCUCAGCCCUCAAACCCUUCCUGUCCUUUUAAAGGG

CAAGACCCAGUUCGAAAGAUACUACCCCCUUCCUGAGCUCCUCAUCAAGCCACAGAAAGA

CAGAUCCCCCCGGGUAAGGGGACAGUCAGUGGCGGGAACCUGGCUGUCCUGGUUCCUCGU

ACAGUAGCCGAGGCCAUUCUGCUGGGAUCCACGGGAUGCCUCAGUGACCUUCCCAGAUUC

AGGUCACCUCCACGCAUACUCCCGACAGGCCUACCUCCUGGGACAGCUCCUCCUGUCUGG

AGAGUCCUCUUCCUGGUGCUUUGGGCACGGGGCUGCUUUGAUGGCAGGUCUUCCCUGGAG

CCUGUGAGACCUCAGUGUGGCUUCGAACCCUCUCCGUGUUCAGCUUGCUGAGUGGCAUUG

GCCCGCUCACAGCCACGUGGAACGGCCUCAGUUUCCCCACCUGCAGUACCGGUGUCCUGG

UGGGUCUGUGCAUCGGUGGCAAUGUCUGCACGCAGCAAGCGCGGGGUAGUCAUCUCUUUC

CUCCUGGAAGGGUGGCAGGAGACCCAAGCAGGGCCUCUUCCCCCUGGGGAACCACAGGAC

CGUGGAUGCCGAGCUGGCGGUGGAAUGGUGCAGGCCGUGGAAGCCGCUGCCUCUGCCUGU

GCUCUGCCCUGGCGUCAUCUGCGGGCUGCUGGUGAGUGAUUCCCAGCACAAAUGGGAGGC

UUCACUUAGUUGGGUGCUUGCAGACAGAGGCCUGGACCCUGAGACCAUGGAAAGCCCCCA

GGUUCCCUGACUCAGCCUCAGCCAUGCUCCAGGCUUCUAUAUGCAUCAUCCCUGCCCUCC

CGCUUCCUACCUGCCGCAUCCCUCUGUGCCUCAUUCUCUCCAUCCUCAGGGGCCCAGCUU

CUCUCCGUGACAGUAGCAGGAUCCUAUGACUCUCCCUGAGGGUCCUGGGGAAGCGAGGGU

CUCCAGGGUUGAGGGUCCAGUAACAAAAUGCUCACAGCUUUUGUUUUCCCGGAGAUCAAC

GCCUCCUCCUCUCCUGGCCUCCUUUCAGGCUUUCUGUGGCCCAGCUGCAUUCUGCCUUCU

UCUUCAAGUCCUCUGGGACCCCUGAACACUCCUGAGCUCCUCCAGACCUUGGGCUAAACG

AAUGGGCUAAAGGAAUGCAGUUAAAAGCAGGGACACAGAGUCUGAAUCCCAGCGUGACUC

UUUUCUGCUGUGUGGCCUUGGGCAAGUUACUUAACCUCUCUAUGCCGCAGUAUUGAAGAC

UGGUUCAUAGCACCGGUUCUGGAGCCAGGUUGCUUGCAUUUACAUCCUGCCCCAGUGAUG

UCCUAGCUGUGAAACAGGGGGCAAGUGGCCUUACCGUCCCGUGCCUCAGUUUCCCUAUCA

GUUGAGCAAGGAUAGUUUUAAGAUUGUGAGGAUCCAAUUAGUUGGUAGGUGCAGAGUCCU

UCGCACAGGGUAUGGACAGAGUAAGCAGACAUUUGAUAUAAUCAAUAAUGUAAUGAUGAC

GAUGCUAUUAUUACAACAUCGCCUUUAGUAGCUUUUUCUGUCUUGCCCCAAGGGUACACU

UUGGGCUUUGCCCCUAGGGCAGGGGUGAGCACAACCUUCUCUGUAAAUAUUUCAGGCUUU

GCAGGCCAUGCAACCGCCCUUCGCGGCCCGUAUGGUGUGAAAGCAGCUCAGCCAGGGUGU

AAGGGAGUGGGCGUGGCCUGUGCCUGUGACACUGGACGCUGCUGCCUUGGCCUUGGGCCC

UAGUGUGCUGCCUCUUGGCCUAGAUUAGCUCUUAUCAGACUCCCUAUGGGAAGGACCGGU

CUGUUUCAUUUCCAGUUUGUCACAAAUCGAUAUGGGGUCCCACUGCACAGACGCAGGGGC

UCAGCCAGGCCCAGUCCGGCAGGGUCAGACAGAGUUGGUUGUGCCCUUGGAUGCCAGGGU

CACGCCAACCCCAGAGGUCUGCAUGCUGUCAUGUUCUGUGCCCACUUCGUUGCAGUUGGC

AACGUUGCAGACCUUUGGUGGAGUAGUACUGCCCUGGGCUAAGGCUCCCAGACCCUGGAG

ACUGCCUGGGCCAGGCUGGGGCAGGUGGGCGCGUGGGGAUCACUGGAAGAGGCUAGACUU

GGGAGGCUUUGCAGUGUCCUGAGUGCCCUGGGGGGGGUCUCUCCAUUAGCUUCAUGGCCC

CCUUGCAGCUCAGUUCCUGGUGCUUGGUGAUGGCUCUUAAGAGGCUGUUGAGGGAUGGUU

UGGCCCUCAAAAGAGAGGCUGGUGGGAGCUCCCAUGCUGGGGAAGAUUCAUUCAUUCAUU

GGGAGCUCCCAUGCUGGGAGAUCCAUCCAUCCAUCCAUCCAUCCAUCCAUCCAUCCAUCC

AUCCAUCCAUCCAUCUGGCAAAUACUUAUCAGGCGUCUCGGGCCCGUCCUCUUCCAGGCC

CUUGCAGUGCAGCUGUGAACAGAACAGGUAAGCAUCCUUGUCUCAGGGAGCUGACCUCCU

UGUGGGGAAUGGCGAGUUGGGAAGGAGAUGAGGGGAAGGGCUGAGGUUGGGGGAGCCCAG

AUUUAAGUAGAGAGUCGGGGAGGAGCCUCACUGAGAAGCUGCCUUUGGAGCUGAACCUGG

AGGAAGGGAGAGGGCCAGGCAUGUGGGAAUCUGGCUGAGGAAUGUUCCAGGCAGCAGGAA

CAGUAUGUGCAAAGGUCCUGAGGUGGGAGCUCUCUUGACUGUAGCCGCCAUCAUUCCGGG

AGAGACCCGUGAAUGCAGCCAGGGUCUAGGAAUGUGCUGAAUUCUUCCUUUGCUUUCCCA

UGAAUCCAGGGUCCAGGAACGGGCUGAAUUCUUCCUUUCCUUUCAGUGUGCAAUCCCAGG

CAUUCCCUUGACACCUUCUGGAGUAAGGAGCCCUAUGCAGGGGCCAGAGAGGGGCACUCC

UCUCCCGCAAAGGAACUUCUCCCAGUCCAUGCUCAAACAUCCCCCCGCAUCUAGCCCCUU

UCUCCUCUCUGCCCCCAGCCACUCCUCCCCACCCUUGGGACCCAGCCAAGAAGGCACCAG

CUGGGAUUCCCUCCUGGAUGAAUGGCUUUUUCUGGUGGCCUCUCACCUUUCCCAGGAGUU

UUAAGGGUGUAAUUUAUUGGUGGCCCUGAUUUCCAGCUUCAGAGGUGCCAUGGUCAGGGA

GAACCCUCUGAGGCCAGGGGGCAUCACCUGAAUGGCUUUUUCCUGCCUCCCUUUCUGAGC

AGCUGGGUCAGACUCCCUCUGCCCAAGUCAGGUUUGCUGCUGUCCUACAGGGACAGGUUC

AGAGCCUGGGGACUGUCCCCUCUUGUCCCUCCUCAGAAAGCCAGCUCAGCCUUUGCCUUG

CAUCUCAGAACGUACCCCUUAUUGCUGUGUGACGUGGACAAGCCACUUACCCUCUCUGUG

CCUCUGUUUUCUCGUCUUUGUGUGGGGCUUCUUGUAAUACCCACCUCACAGGGUUGCUGU

AGGGAUGAAAUUAACUAAUAUAUUUAGAGCGUUUAGCUGCAGGUACACAAAGGCAAUUUA

UUACUGUGGGGUAUUAUUGUUGUUUUUUAUGAUUAAUGUUGUCGCGUUGGGGAGCUCUCC

UGCUUGGAGACGUGGCGAGGUGGUUUCCAGCCUGGGCGUAUUCCAGCUCAUGGGACCCCU

UGGGCUCCACCUCAGGUCAGUUUCUCUGGUGGGGCCUCCCCAAUUCUUGCCUUCCCCUAG

AGCCUCCAGGUACUCUUGUUGGGGGAGUCCCUGGGCUCAACCCUGGUGACCUGCUUCAAG

CCUAGUGGUGGGGGCCAGUGUGGACCGAGUGUCUCCCAGGGUUUAGGGUCCAGUAACAAA

AUGCCCACAGAUUUUGGGCAGAAACGGCACCCAAGAUGCUCCUAGGAGGAUCUCCUAAAU

UGGGAAACGAAGGCCAAAGGGUUCAUGCAGAGGCCUCCCCAGCCCCCACUUCGGGCCUCC

UCCACCAUGGUUGGCUGCCGUGGGCACUAUCGGGCCGAGGCCCUCUUCCCAGGGACUGGA

CCGUCCGCCCUGCUUCUCUGCCCACUUGGCCUUGGUAUGGGAGAGCUGGGUGGAGAGGAU

CCUCCUGGCAGACUGUGAGGCCUGCUGCUUUCUUCUCACACCUCUGUGUGUGUGGCAAGG

UCUUUGCCUGGCAGCUGCGUCAUUGUCAGCCCAGUGGCCUCCUCCACUCCCUCCCUCCAG

CCCCGUCUUCUCCCUUCUCCUCCCACUGAGAGCACCUAGAGUCUUGUGCUAGACACUGUG

GGAUGCAGGAGGAGGCUCGUGCCUUUUCUGCCUUCUGGAGGCUGCUCUCAGCCUUGGGGG

AAUCCCUCAGAGAGGUGAGGGAGAUGAGUGUAGGAGGUGCAGGCCUCCUUUAAUCUGGGC

UGGGCACCCAUUUCAAAGAGGAGAAACUGAGGCUCAGAGGGUUAGAAUAACAGGGCCAAA

GACACACCUUCUGUUGGAGGCCUUGCAGCUUUUGGUAUCUACAUCGGACUGUCCAGGGAG

GUCCUUUCCCCAGCUUUUCCAGCUUCUGAGGCUGCCAGGGUCCCCAUGGAUUCCACUUGC

UCAUCCUUCCUGGCCUCUGGGCUCCCGGGCUCUUAUGUGCUGAUUUUGUUGGGUCUGCAA

CAGAUACAUUAUCAACGCACCCACCUGGCAUUGUCUCUGAUUCUCUUUGCAAAAAUAACU

GCCUUGUUGAAAAAGCCAGACAACAAAAGAGUGACUAGAUUUUUAUGGAAAAUUGGGAAA

AUUAAAAAAAGGUGCCAAGAAGAGACGAAGUCCCUUGUAAUCCCACUCUCUAAAGCCUUA

CCUGUCAGACAUCACUAACUGAUUGCCGCAAACAGCCUCACUGAGUGUUCCAGGCAGACA

GAACUAAUCUAUUGGAAUUGGUGCGUAAGUGAAGCCUGUCUUGUUUUUGUUUGAGAGGCU

UGUUACUGUCUUUUUUUUUUUUUUUUUUUUAAACACAGUUUAAUGUUUCUCAAAUCUGGG

UGUACUUGGCAAUUGGUAUACAUCUAAUUUGUUGAUAUUGAGUUUUACCCCCUAUGAUAA

UGGAUAAUGUGUUAAAGAAACAAGUAUUUGGACUCUUGUUAAGCAGGAAGACCGUACAGG

ACAAUUGUGCUAUCAGCGUCAAGACUGUCACGAUAAGGGAGAGAGACCAGGCUCAGCGCC

AAAUACGGCCAAGACAGCUGGGGCCUCAUAGCCUAGCCGCAGGGCGGAGGGAGGGGUCGG

UGGAUGGAAAAUUACUAAGAGGAGCGCUUGAGGGUGGGGGGUUCCUCUAGACUGACUUAG

CAGGAUGCCUGCAGCUGGGCAGAGCCCCGCAAGGCCAAGGCCAAGGCCGAGGCCCCGUAG

AGAGAGGAUGGGUCAGAGGAGCUUGACUCAGGUGUGGGCAAGGAGAGUCUUUGUCAAAUG

GACGACGGUGUGUUAUUAAAGGAGGAAGGUCUGCCUAAGGCUUACCCUUCUGGCUGUGAU

ACGAGCGGGGAGUUCCUCUUUUACAGGGCGGCCUUGGGGUCAGGGGUCGCUUUGUUCUGA

AACCUGGUCUUUGGGCCUCGCUGGCUGUCGGGUGUUUGUCGCCAUCACUUGCCACCAUCC

AUCCUUCUCCUGGCGAGUGAGGCCCGAGCCUCGUUCCCUCGCGUGGGCAUCACUUACCCA

CCCUGCCAAGAGCUGGGCAUCUAGGUUGGGCCUUUUCCCAGUUGUUGCUGCAAAUGCAUU

UCUGUUUUUACAAAUAAUGCUGCCCCGUAUGCACCUUCAGUCCUGUCUUCCCAGGCCUGC

AUUUCCAAGCUUCAGCUCCUCAGGCUGGAGGGACCCACAGCCUGGCAGGGGUAGGAUGGG

GUGGGAGGGUAGAAAAGCGAAGACAGCUUCCUCUACUCUGGCCCCCCAGUCAACGCCAGC

AUGGCCGGCAGACAGAGGCCAUGGUUAGGAUGGUUCCAGGCCUGUAUUAGCCUGUGGCCU

CAGACAGCCCCAUCAGACCUCCCAGGCUUGGCAGUCUCAUUUGGAAGGUGGGCGUGAUCG

UGGGUCCCUGGGAUGCUGUGUGGGAAGGAUGCAGCCCAGGGCCGUUCAGCUGUGUUCUUU

CUCCUCCUUGGUGUUCGGCCCCAAGGGUAGCCUCUGGCCAGACGGUCCCUGAGGUUCCCU

GCAGGACUUGGGCUUAUCUGCCUAGGGCAGGAGCGAGCCUGAGCAGGGAGUCCACGGCCU

UGCCCACUGUGCCCUGCCGUGCCCCGCCAUGCCCGUGCCCGCCCACGCUCACCACUCCUC

UGUCCCCUCCUGCAGGACGUCGGGCUCCUGGAGUACCAGCACCACUCCCGCGACUAUGCC

UCCCACCUGUCGCCCGGCUCCAUCAUCCAGCCCCAGCGGCGGAGGCCCUCCCUGCUGUCU

GAGUUCCAGCCCGGGAAUGAACGGUGAGGAGAGAGUUGCUGCCUCCCUGUCCAGGGCGUG

AGAGCUCCUUCUCAGAUGGAGAAGCAGAGGCCCGUGGCAGAGCAGGAGCACACAGGGUCC

UGUGAAAUGAGGGGAUGUCGGGCCGUGUGUUCACCCUUGACCUGGCGUGGGCGUGACUCG

GGGUGAGGAUGGGACGAGCAGGGAAGAGCCACGUGGCCCUCAGACUUCCAGCGUGGAACU

UCAGUGGCCUCUGUGGGUUUAGGCAGCAGUGGGGACCUCCAAGCUGGGCUGCAGCAGGGC

UGGGCUUCUGUGGGAAGAUUUGAGUCCACACCUGGUGCCCCUGGGGCCCCUGCCCUCUCC

UCCCAUUCUUCCUUCUUCUCCAGCUGGCAGAGGACACCCUCUGGUGUGUGCCUGUGCUGG

GCCAGCCUUACUUUCCUUGAUCCAGGUGGGAAGGGAAUGAACAGGUUAGGUGGUAGGGUG

AGAGGGAGGUGGGGAGUGGGAGGGAGGUGGGGUGGGGAGCUGGAGGGACUACAGCAAGGG

GAGGGCCAGGGCUGGGUCAGAGCCAGCUCUCCAUGCAUUCACUUGGGAUCGGUAGGCCAC

GUUCUUUCUCCCAUCUUCUCUAGGAGCUUCUGCUGGAGACGGCUGAGGUGUGGGGAAUUG

UGCCUAUUUCUCAGAUGGGGCAGCUGAGGCUGCGAGGUUGAGGGAUGGGCUCUUGUUUAC

UCAGCCAGGACUAGAAGUCGAGGCACCAGGCCCCAGCCCUUCAUGGUACCACGGAGGUGU

CCCAGGGCCCCAGAUCAGGCGGGGCUGGUGAACUCAGGUGGGCGCAGGCGUCUGGAGGAG

GGGAGGCCGAGGCAGCGUGCCUUCUGUACCUGGAUUGGUUUCCUGGGCCUGCCGUCGCAA

CGUGCCACAAACUGGGCGGCUUCAAGCCACAGAAGUGCAUUCCCGUGAUCUGAAGUCAGG

GCGUCCGCAGGGCCGCGCCCCCUCAGAAGGCUCAAGGGGAGGAUGCUUUUCUGUCUCCUC

CAGCUUCUGGGGGCUACAGGCGUUCCUCAGCUGUGGCUGCAUCGGUCCAAUCUCUGCCUC

UGCCUCUGCCUCUGUCUCACGUGGCCAUUUCCUUGUCUGUCUCUGUGUUCUCUUGUUGUG

UUACAAGGAUCCCAGUGAUCCUAAUCCAGGAUGGCCUCAUCUUAGCUUAAUUCCAUCUGC

AAUGACCCUAGGUCCAAAGAAGGUCUUAUUUUGAGGUUCCAGUGGCUGUGCAUUUUGGGA

GGAUGUUAUAUUUGGAGGUUCCAGUGGCCGUGGAUUUUGGGAGGACAUUAUUUGAGCCAC

UGCAGUGCCACUAUCAUGUUUAAGAGAAAACACCAUGCGCUUCCUGCCAAGCCCCAUGUA

AUGGUGAAGCCCUGGGUUCUGGGCCUUGAAGAGGGGGAUGUCUUGGGGCCCAGCUUUGGA

GGGACAAGGACUUGAGGGCCCUCCAGGGAGGUGAGCACAGAGGGGUGGCUGCCCCGCUCC

CCGGGGGGGCCAGCAACACGGCCCCAGCCCGUGCUGCAGUGGGCAGCAUUCAGUCAGUCU

ACAGGCACUGAUUGAACACCUACUGUGCGCCAGUACCUGGGGACAGCCAUGAACAAGACA

GGUGAACCACCCUGCGUUCAUUCAUGGGUGGGGGAGGUGGCCAGGAAACACCUGUUGUCA

CCAAGCGCGUGGGAGGGCAGUGGAGAGAGAGCAGACAGGCGGGUGUAGUGUGGUCUGGCC

UGGGACAAGGAGGCGCCAUCUGCACCAUGACAGGAAGGGGAGGAGCUCACAUGGAGGGCC

UGAGGUCUCAGUGCUCCAGGCAGAGCCCAGCAGGUGCAAAGGCCCUGGGGCAGGAGUGCA

GUGGCGGGACAGCGAGGGCCAGGCCAGGCAGGGCUUGUGCAGGAGGGGAACAGCUGUGGA

UUCCCUGCUUAGCCUCUGGGAAAAGCAUUUCAGCAAGGCCCAUAUUCAGGCUCUUUCUGG

UGGGUGAUGGUUGGAGCCUCAGAGGUGGAGGCCAGGAGUCCCAGCUCCGUGGGGUGUGCU

UAGUUAGGCAAGGCCAUGCCCUCUCUAGGCACACUGCGGAGGAGGGUUCAGGAGAGCCAG

UCGCUGGGGAAACUGGGCAGCUCCAGUCUCUGCACCAGGCUGCUAGGCUGAGUUGAGAAG

GUGGUUAGGCCAGGUUCAGCAGCAAGAGUUCUGCGAUCGGCCAGCGAUGUCUGCCCUGCG

UGGGUGAGAGGGAAAUGGCAUCACGGGUGCUGUUAUCUGUGUUAGGCUCACAGCGUUUCC

CGGAUGGACUGUGCCUGUGAACCACCCAAUGAUCUUGUUAAAAUGCCGAGUCUGACUCAG

UAGGCCUCAGAUGGUACUGGAGAUUCUGCAUUGUAGCCAGCCCCAGGUGGGGACGGUGGG

GUGGUCCGUAGCAAGGGAGGGGCAGUCACUCUGGGGUGUUCCAGUGCCACCGUAGCACAC

UCCCUCCCUGUAAAUGUCCUCGGUGAAUGUGGACCUGAAGGAAGGCAGCGCUGCAUUGGA

GUCCUGCCCUGCUGUGGGACCGUGGGAGGUGGGCUCCUGUGAGCACCUUCCUGGCAGGAU

CAUACAGAGCACGGCGCGCAGUAGAUGCUCGGCGCAGAGGGUGGUCGGGGCCUCGGGAGU

UCUCAGAGCCGCAAUCUCCUAUGUGACGUUGGAUGCCUCACCUCCUGCAGGUCCCAGGAG

CUCCACCUGCGGCCAGAGUCCCACUCAUACCUGCCCGAGCUGGGGAAGUCAGAGAUGGAG

UUCAUUGAAAGCAAGCGCCCUCGGCUAGAGCUGCUGCCUGACCCCCUGCUGCGACCGUCA

CCCCUGCUGGCCACGGGCCAGCCUGCGGGAUCUGAAGACCUCACCAAGGUAAGCCUGGGC

CCCCAGCUGGGAGCUCCUCUCCCGCUUGAGGUUCUGCUGCUGGUUGGGCUGGGUGGGAGG

UGGUGGGUAGGGGUGCAGGGAGGGGGCAGCUCGGGCAAAGGCUUGGAGGUGGGCCAAGCA

CCGGGUGCACAGAGCUGGGUGGCAGGAAGAGGGCAGGCGCUGGGUCUCUGGAGGUGCCAG

AGUGUGGGAAGAAGUGGGGAAGGUGUUCCAGGUGGGCAGAGCAGCAAGUGCAAAGGCCCU

GGGGCAGGAAUAGGCUUGUGGUCACGGGGUAGCUGGCGUGAGGGAGGGGAGAGCUGGGAU

GGGAGGGCCUGGGGUGCCGUGGAGCAGAGAGCUGCCAUUCGAUGGGGGUCAGGACCGUCC

UGUGGUCGGUGGGCAGCUUUCAGCACAUUCUGAGAGCUUCCUCGAGGGUGGGUUGUUUGG

UUCCAUGCUUGCUUGGAACAGGGGGCUCAGUGACCCCUGCCUCGAGCCCUGCACAGCAGG

CAGCCCCCUGGGCCUUGAACCCCGGCCCGCGAGGUGGACAGCUCAGGGCUCUGCCAGGCA

CCUGAGGCUUCUGGAUUCUUAAGGGUCAGGGAGGGGAGACUCCAGAGUCCCGCAAAGUGA

GAUGAGACAGAGUUGGCUCAGCACUAAAGGGGCGGUGCUUGGUUAGGACAUGGCCUGGAC

CCCUAGAAUGACAAAGCCGCCUUCUACUGCCCAGUCUGGCUGCUCCUUGGGCCACCGUGU

GGCACUGGGCUAGCGUGGGUUGAGUCUGCAGCCACCCAGGCCUGAGGGACCACGGAUGCA

CUUUGGGCAGGGGAGCCAGACAACUUCAGGCUCGUCUGCAUCUCCAUGGGGGGCUGCCUG

GCAUUGCAGGUGGGCCCAGGCCUCACCUGGGCACACUCACUUAUCCCAGGGCCCUCCGUC

CAUCUGCCCUCAGGUGACCCAGGUGCGAUUUCCACCACCCCCACCUCCACAGCUGUGCUG

CCUUUGUGCCUCUCUGAUCCCCAUUUCUUCCUCACUGAUCUGGGACCUUGUGGGCAGGGU

GUCACCUGGUCAUGUGGGUAGAGGGCUCGGCUGUGGGCCCCAUGCGUGGGAACUAUUUGG

CACCCGUGAGCCGGCAUGGGUGGUGGUCUGGCUGCGGGGCCAGGCCACCUGGGCCAGACU

CGGCCUUCUCUCGGGGAGAGAGGAUACCUGCGUGGAACCUGGGAUAUCCCUGGGGUGGGC

UUGGGCUGGCAGGAGGUGGGGGUGCCGUCCUGGGGCUGGGAUGGCUGGGCUUCCCCUCCU

GCUCUGAGUGGCUGUGGCCCCGACACCUGCACACCUGCCAGAUACCCACAGGGCUGCCUC

UGGGGCCAGGGGAGGGAGGGGCUGGGGUGUCUGUGUGGUCGUUGGCUGGCUUGGCCUCCC

UGCCACUGGGGCAGUCCCCGCCUCACCAAGUUGUCACAGGAAAGUGGAGGUCAGUUAAUG

AACUCCUAGCCAACAGACCACAUGCGCCUGAGAGCCCACUCAGGAACCUCCCCCGGCGGA

UGCAGCUGUGUUCUUGUUGGCACUCAGGGACUCCACCCUGGCCACCUCCCUGCUCUCCUG

CGUACUUCCAGUCCAUCAGCAAAUCCUGACACCUCUGCCCUAAAAUAUCUCAAUUCCAGC

CUUCUGCCCACACCUGAGGCAACCCCCGGCCUGAGCUCGUCAUUGCUCUCCAGUAACUUU

GCAGUAGCCUCCUCCUGGGUCUCCCUGCUCCUGCCUCCCUCUACUCCCUUCAGUUUAUCU

UCCAAGCAGCAGCCAGAGGGCUCUUAUAAAGGCAGACGACUGCAUCCCCCUGUGUGUUCC

CCCCUGUGCUCCAAACCAUCCAUGGCUCCUGUCUCAAGCAGGAAAAAGCCAAAAUCCUUG

CCAUGGGCUGCAGGUCCGGCGUGAUCCAGCCCCGUCACCUCCCUGACCUCAUCUCCCUGC

AGCAGCCCUCUCAACCGCUCACGUACAGCCUCCCAGGCCACCCUGGAGUUCCUCAAACAC

GCCAGGCACAUUCCCACCUGGGGACCUUUGCACCGGCUGUGCCCUCCGCAGGAGCGCCCU

CCCCCAGGCACAUUCCUCUUUCCUCGCUCCUUCCAGCCCUGGCUGCCCUCCAGGGCCUGC

AUGGCAGGGGAGACCUGUGGCUAGCCCUGUGGGGACUCUCGAGGCCACUCAUGGCUGAGG

CUGCAGCCCCACCACGUCCUCCUGGGGUGUGAGAUUCAGCCACAGGGGCCCUGCCGGCCU

UGAAGGGGGUCUCUGUGGCCUGGGGCUUGGGCCGCCUGCACUUGGGUCUGGGCUUGGGAC

UGGUGUCGAUGCCAGUUCCUCUCGGGCACUGCCCCCUCGGGUACCCCAGCCAGCAUCCCU

GCCCCGACGCCCAACCCUUCCAGCACAUCCUUCCUGCCCUUCCUCCCCUUCCUUCUCUCC

UUCUACACCUUCCUCCCUCCUUCUUCCUCCCUCUCUUUCUUUCCACCCAAACAGCCCCCC

UGUCCUGCUCACUCCCCUUCACCCCCUGCUUCCUCAGCUCCUGCCAUCUCUCCCUGCUCA

UCCCCUGGGGCAACCUCCCUACCACAGAGCCACUCCCAGCCACCACCCAGCGCCACCCAG

CCCAUCUGCCUGCGGCCAUCUGUCCUUGCCUUCAUUACCUGCCCCUCCCCCUCACCUCCC

AGCCGCCCCUCACCCAUGGGUUCUGCCUGCCAUCAUCUCCCACCAGUGGCCUUGCCCCUC

CCCCAUCCACUCACCUCCCCCAUUCCCUUGCUCCCCCACCCCCUACCCCCAUCCGCUCCC

CCUCCUUCCCCCACUCGCCUGGAGUCCAGGGUCCUGUGAGCCCAGUCAGCCCUUGCAGGA

ACUCACAACUCACAGCCGGACAAGGGCGCCUGGGGGUGCUUGGCCCGUGGCCAUUUCUUG

GCUGGAGAACGACACCUCCCACGUGUGUGUCCAGCCUGGGGCCUGCCACGCCUCCAGUGC

CCUCCACAGCCCUUUGAUUUCACUCCUCUUUGAAACAGAAAGUGGAACCUAGACGGUGGC

GGUGGUGAGGAUGUGCACAGAUUAAGCACCUGUGGUUUGCUGCUGUGGGAGUUUGCUGUG

GUUUCAGUAAAGCUCAGGACAUACCAGGCUGGAGGCUUGUUCUCUCUCCAGCUUCCACAG

AGGAUGCCGAUGGCAGGGCUGGUGGGGGAAUCGCAGGCAGUGUCCCCAGAGGCUUCAGUC

CCCCAGGGUCACUCAGCCACCCAGCUGUGUCUGACUGCCCAGGCCAGGGUGGGGCUGUGG

CCAGGUGGCAUCCUAAUCCUUGUUGUAGCCUGGUGCCCGGCCAGCUGUGACACGGGCCCC

UCCCUGCACAGCCCUGGGGCACCCUAAGCUCCUUAGCAGCCUCAGGGCAAAGGCCAUCCU

CCUCCUGCAGCGGGAGUAGGGGUGGGCGGUGGGGACUGGGAGACAGCCCCGGGGAGGGAA

GUGGCCACUCAGGCCUACAGGGUCCCCUUUUCUGCCCAGUGCGGCCGGAUUGGAUUGAAU

GGUUGACCCCUAGGGUGAGGCACCUCCUUUAUCAGGAGGCUGGCACGCAGGAGGCAUUCA

CGCAGUGGUAAGAAUGCUGCAGUGAUGGCGGGUGCCAUUAAGUGAGCGCCGACUGCCUGC

CACGUGCCAAGUCAUGCCCAGCCAGUGUCAUUCCCUGAUCUCUUCCCAGCCCAAGGAGGC

UGGGUCUGUGACCCCCUUUUGUAGACGGCCCCAGGCUGAGCGAGCUGGACGUGGGGGAAC

GGGAUUCAGGCCCAGGCAGCUGGGAGUGCACCCCAUGCCUGCCUCCAGGUUGACCUGCUG

CUGUGGGUCUCCCCUCUGGUCUCCCUGGUGGGGUGCCCUCCUCCCUCUGCCUGGCAUCCC

CCAUUAGCACUGGAAUCUGCUUUCUCCUCUUUUGUUUUGUUUUGUUGUUACAGAAGGCAG

GAGAGAAUUUUAGCUCAGCCUCCUGGGAGAGAUGUUUCCAUUUGAACAACUCCCCUGAAG

UGGGGCCUGGAUGGAUAGCAGGCCAGGGUGUGUGUGUGCAUGCGCGCGCGCGUGCGUGUG

UGUGCGCGUUUGUGUGUGGGUGCGGGUGUGUGUGUGCGUGUGCGCGCAUGUGCGUGUGUA

UGCGCGCAUGUGCCUGUGUGUGUCCCUGCGUGUUUGUGUGUGUGUGUGUAUGCGUGUGUG

CACACAUGUGCCUGUGUGCGUGCAUGUACCUGUGUGUGCGUGCGUGUGUCCAUGCGUGUU

UGUGUGUGUGCGUGAGUGUGCAUGUGUUGCACGUGUGUAUGUGUGUUUGUGUGCGCACGU

GUGUGCAUGUGUCCGUGCAUGUGCGUGAGUGUGCAUGUGUGUAUAUGCAUGUGUGCGUGU

GUUUGUGUGUGCGUGUGUGUGCAUGUGCACGUGUGUGUGCAUGUGUUUGUGCGCAGGCAG

CUCAGGGCCCAGCCUUGGAGGGUGACCAACCCCUGAGUGGGCAGUUGUCCCCCAGGUGCG

CGUUUUGUCUCUGCGCAGAGAGCAGGCCCGAGGGGUGAGUUGCUGCCUGCCCGCCUCUCC

AGCACGUCCGUGCGCUCUGGCCUGUGGGUUCUGGAGGGUCUCCAUCCCUUACCGUUGCUC

UGUGGGUUCUAGAGGGUCUCCGUCCCUUACCGUUGCUCUGUGGUUCUUUCUGCCUGCCUG

GCUUUCCCUCUUCUUGUCAGGAAUGCCUCUGCCUGUGGUGCGCCCUCUGGGAUCUCUCUU

AGUCUCUGUGUCUGUCUCUGUCUGUGUCUCUGCCUCUCCAUCUCUUUCUCUUUGUGUCCC

UCUAUCUCUAUCUCUUUCUGUUUGUGUGUGUCUGUCACCACGUCUCUGUCUUUCUGUCUC

UGUUUCUGUCUUCAUCUAUGUCCAUUUCUGUUUCUCGAUCUGUCUGUCUGUCUUCGUCUC

UGUGUCUGUCUCUCUCUCCUCUCUGCUUGGACCACAAGGUGGAGGUGUGAUGAGCUUGGU

GCUGGGGCCUGACCAGCCUGGCCGCAGUAAUUGCUCCAGGCCGUGAAGAGAUUUGGAAAG

UCUGAGCAAGGGAAGAUGCCUGGGAGAGCGAGUUGAGAGGUUUUCUGCAUCUGCUGUGUG

GAAUUCAGCUGGGCGCCUGAGGCAGGAAUUUGAUGGGAUUUUUCUGUGUGUGCCCCCUCU

GCCCCACCGUGUUCCUGUUUUCUGAUGCUUCUCUGCUCUUCAGCCUCCGGAGUUUUGUGG

CAUCAGAGACUUCCUUGGGAAUUCACCGAACUUUGUUUUGGGGACUUUAGAAUUGUCACA

AAUAGCACUAUCUCUGAAAAUCCCUAAGGGGGACAGACAGCCCCUAUCAAAUAUAAACAU

GAAUUCUGGCUCACAUAACUGAAGUCCAGCAUUGGUUAGCUUCAGGUAACAGCUUGAUCU

AGGUGACACGAUGUCCUCAGGACCCUGUCCCUUUUCAUCCCUUGGCUCUGCCAUCUUUCA

CAGGGUCUUGGUGUGUUAGCAGGAAGGAAGCCGGUGACUCCAGGCCUUCCUCCAGGUUCA

AGGCCAGCAAAAAGAGAGAACUCUUCCUCCUCCUCUCCGCAUUUCUAGCCCCAAACUGCU

UUUCCUCCUCACCAUGAGUGGGCCCAUGCCUGUCCUAGAACCUGUCAUUGUGGCCGGAGU

AGGGGGUGUAUUGGGAGUGCGUGAAGCUGGGCCCUGCUUUUUCCUGGAGCUGGCACAGGG

GAGCCCAGGGGCUUUUCUGCCUCUCUGUCUUCAGUUUUGCAUAAUAAACUUUUCUCUUUC

ACGCUAGCUUGAAGUUUUACACCAGGGGCAUCUGAUGAGUAAGAGGUACCCACUGCCCCC

UGGCAACUUGUGCGGGGUGUAUUAGUUUGUUGUUUUGUUCCCUGCAGCUUUGGGUGAGUG

GGAAGGUGGACAGUCAUCAGGAGUGCACCUGCAGGCCUGACCGCCAGGCUCCCUCAGCCC

CCGUCCUGGGAAACCCAGCACCAGGGGAAGCGCUCUUGGCAAUCCGGGUUUGCCUCUUCU

UUUUCAGUUGAGGGUCGGUUCACAAAAAAGUAACCACAUUAAAGUGAAGAGCUCGUUGGC

UUGUAGGACAUUUACAAUGUUGUGCAGCUGUCACCUGUCUAGUUCUAGAUCAUUCUCAUC

ACCCCAAAAGGAGACCCCCAUACUCAUUAAUCAGUCACUCCCUGUGUUAGUCUGUUCUUA

CACCGCUAAUAAAGACAUACCCGAGACAGGGUAAUUUAUAAUAGAAAAAGGUUUAGUUGA

CUCACAGUUCUGCAUGGCUGGAGAGGCCUCAGGAAACUCACAGUCGUGGUGGAAGGGGAA

GCAAACACGUCCUUCUUCACAUGGCAGCAGCAAGAAGUGCUGAGCAAAAGUGGGGAAAGC

CCCUUAUAAACCAUCAGAUCUCAUGAGAACUCACUCACCAUCAUGAGAACAGGACGGGGG

AAACCGCCACCAUGAUUCAAUGACCUCCACUUGGCCCCUCCCAUGACAUGUGGGAAUUAU

GGGAACUACAAUUCAAGAUGAGAUUUGGGUGGGGACACGGCCAAACCAUGUCACCCCCCA

CUUCCCCCUCCUCCUUUUUCCUGGCAACCACGAAUCACUGUCCAUCUCUCUGGAUUUGCC

UAUUCUGGACAUUUCUUGUAAGUGGAAUUGUACACUCUGCAGCCUUUUAUGUCUGUCCAC

UUCCACUCAGCAUCAUAUUCUCGGGGUUCAUCCACAGUGUAGCCUGGGUCAGUGCUUCAU

UCCUUUUUUUUUUUUUUUUUUAAUAAAGAUGGGGUCUUGCUAUGUUGCCCAGGCGGUCUU

GAACUCCUGGGCUCAAGCAAUCCUCCUGCCUCGGCCUCCAUGUCCAGCCCUUCACAUUGC

UUGUAUGGAUAUGCCAUGUUUUAUUUAUCUGUUCAUCAGGUGAUGGACUUACUUGUGCAA

UUUUGCUGCGAAUAAAAGCUAAACCCGAACUUAAAGAAGAACCCAUUAAAGACAACGGCU

UUUAUCCAUGGAGAGUUUAAAGGAAGUAAGUUUUCAGCAACUUUUCCAAGUGUUGCUAGU

GGUUGCCAGCCGCUGCUUUUACUCCUGGUGCUGCCUGUUAGCCCUGGGGAUGGAAAUCUU

CUUUUUGUUAAUGUUGCUUUGGAGAAUUAUUCCCAGGUUGGACGAGCAGUUUGCUCUCAG

AGUUUUAGGGUGGCCUGGCCCCCAUAGCCCUUCCUUCCACAUCCUUGUCCUUGCUCUGUU

AUCUCUCUUCUUGGUCCAGGUGGAAGAGCAGAGAUAGGAUAGCGGAGAUGUUUCUUGCCA

CCAGUAGUGCGACCGGGCUCUGGGUGCCAUGUUUGCUGCCACCUUUUGUCUUAAGAACCU

CAAGAAAUGACAUGGGCUGUAGGGAGAGACCAGCCAAGCACCCAAGGUGGACCGCAGUGA

GGGCCUGGGCGGUGGCGUGGGGAUGUCUCACGUCAGUUGGCACAGAAGGGGUGUUGGGCA

CUCUGGGUAGCGGAGCCAGCCUGUGCAAAGGCCCGGGGGUGCCAGACGCAUGAGCUGUCC

AUGGUUUGCUGUGGGUGGGGUCAGAUGCCCAUGGCCCCCUGCCUGCCUUCCUGUUGGGGA

GCAGUGAGGGCCCCACACCCACUGGUACGUGCAUACACACUCCUGAGGCUUUUUCUAGGG

AGUUUUUCUUCCACACACACUUUUGCCUUGAGUUUCUGGAGGUCCUGCACUCUGGGUCCC

UGGAAGGGGUGCUGCUUUGGCGCUUAUGCUCAGCCCUAUGGUUCUGUGGGGUGAACGGAU

GUGUGGCUGGGACCCCACGUGGAGUCCCCACAUGGCUCAGCCCCACGGUUCUGUGGGGUG

AACAGAUGUGUGGCCAUUUCUGACGUGGGAGCCCCUGCAGCCUCCCCCUUCAAGCACCUU

CCAAGGUAGGAUGCUGAGUGGCCUUGGGCAUGUCGUCAAACAGAAAUCCCACUUCCCACG

GUGGUUAUGACGGUUACGUGGAUUAAGAUUUGUGAAUUGCUUAGAGCAGGAGGCCAGCAA

ACUCCAGCAAGCUCCAAAUCUGGCCCUCAGUGUGUUUUUGUAAAUAAAGUUUUAUUGGCA

CACAGCCAUGCCCAUUUGUUCCCCGUUGCCUGUGGCUGCUUUUGCAAUACAAUGGCAGGG

GUGAGUAGAUGAGACAGAGACCUUGUGGCCCUCAAAGCUUGAGCUAUUUUUACCAUCUGG

CCCUUUAUAGGAAAGUGCCGGCCUCUGGCAUCCAGCCCCAGUGGCCUCCAGGCUCUGUUU

CUGCCUCUGCGUAAUGGAGAUGGAGCAGCAGUCCCCUCGGAGGGCUGUUGUGGGAUCCAC

UUCAUGGGGUGUGCAGAGGACUUCAUGGGGUGUGCAGAGGACUGGGAUAGGCUGAGCUGU

GAGCGGCCCCAGGUGUGAUUUCUAGGUGUCCCUGGGGGAGGUGGCCUGAGGAGCAUGGAG

GACUGCAUUGGGGCGAUUGAGGGAUAUGAGCCCUUUUCUCUGUUUCCCUCGAGGAGUCCG

UGUGUGUGGAGGGAGCACCGGGUUGUCCAGGCCCCUGCUGUCCUUGCCCCCACAUCUCAC

AUGGUAACAGAGGCCAGAGGCAAACCUAAUGUGACUCCAAGACUCUUGGCCCCUGUGGGU

CUUGCUGGGGCUCUGUGGGGUGAGGGGAGGACAGAGGCCCUGAGGAUGGCAGGAGUGGAC

AGAAGAUGAUGCAGCCUCUGUGAAUCCUGAGGUUGGUGGGGCACUCGGUGGCCCCCGGGC

UGCAGUUGUCCGGAGAAUAAUGCACCCUGUGUCCUCUGGGUGCAGCUGCUGCGGGGGCGG

GGAGGUGGUGGCCUGGCGCUGCUCUGGGCCUUGUCUGCCAGCAGCUGCCGGCUUGGCCCC

AAGGAUGGCACAGGGCGGGGGCUGGGUUCUGGAGCCCAGCUAGGGGAGGACCAUCCCAGC

CAACAUUUAUUGAGGCUUACGGUAUGCCAGGCUCAGAGCCAUUAACUCAUUCAAGACUCA

CAAGGACCGGGGAGGUGCCAGCUGUUCUCCCCAUUGGGCAGAUGAGGAACUGAGGCAGGA

AGCAGUUGAAUGACCUGCUGGAGGUCACGGAACUGGAGAGUGGCAGAGGGGGCUGGGAUU

UGAACCCAGGGCUGCACAGGUGAGGCCUGGGUUGUUUUUUCUAACCUCCUUAUCCUGAGA

GUGUUUCCAGCACAGCCUGGGUGCACCAGACUUUAGCUGGUGGGAUGAAGGCGUCCAUGG

AUAACUGAAUGCAGGCUUCCUUUGUUGUUAAGCACAUUUUCAAAACAGCUGUAUUACACA

AAGAGUGACUCUCAGUGAAAGAAGAUCAGAAAAAAUAACGGGCAAAUAAAAAGAAGAAAG

UAAAAAUACCCUCUUCUGCCCUGCUGCCGAGAUAACUCCUUUUAGCUUAACUGUAGCCCG

UUCUGAUGGCUUUUCCGUGUGUUUGGGAACAUAUGUUUUCUGCAAAGCCACGUGUGUGUU

GGCUGCAUGGUUCUGGAACUUGCGUGUUUGGACUGAGCUUCAUGGAUGAAUGUUUACAGA

GGGUAUGCAGAGGCCAGGCCUUGGGAGAAUGGAGGCUGUGGGUCUUCGUUCCUUGGGCUG

GUGGGGAACACACAGAUGGGGUAAGUGGAAGAAGAGAGGUGUGUUACGCGAACAGGGAGU

UGGAAGACUUCACAACUUGAGGAGAGGCUGGGAAACCAUUCAUGUAGCAAGAGCAUUUGA

ACCGCAUCUUGAAGGCUGUAAGGGACUCUGAAUUUUGGAGAAUGGGUGAUGGCUGGGAUG

GGAAUGGGAUCCUUGAACAGGUGAGGACUGUCCUGACAGAUGUCCAGGACUCUGGGCCUG

GAGUCAGGGAUGGAAACCCGGGGUGUAUUGGGGAGGGGGUGUGUAUUAGUCCGUUUUCAU

GCUGCUGAUAAAGACAUACCCAAGACUGGUCAAUUUAAAAAAGAAAGAGGUUUAAUUGUA

CUCACAGUUCCAUGUGGCUGUGGAGGGCUCACAAUCAUGGCGGAGGGCAAAGAGGAGCAA

GUCACAUCUUACAUGGAUGGCAGCAGGCAAAAAGUUGGCAACUACAUUAGUCAGGGUUCU

CUAGAGGGACAGAACUAAUAGAAAAAAUAUAUAUAUAUAUUACAUAAAGGGGAGUUUAUU

AAGGAGUAUUAACUCACACGAUCACAAGGUCCCACAACAGGCCAUCUGCAGGCUGAGAAG

CAAGGAAGCCAGUCUGAGUCCCAAAGCUUGAAGAACUUGGAGUGUGAUGUUCGAGGGCAG

GAAGCAUCCAGCACGGGAGAAAGAUGUAGGCUGGGAGGCUAGGCCAGUCUCGCCUUUGCA

CAUUUUUCUGUCUGCUUUAUAUUCGCUGGCAGCUGAUUAGAUGGUGCCUACACAGAUUAA

GGGUGGGUCUGCCUUCUCCAGCCCACUGACUCAAAUGUUAAUCUCCUUUGGCAACACCCU

CACAGACAUACCGAGGAUCAAUACUUUGCCUCCUUCAAUCCAAUCAGGUUGACAGUAUUA

ACCAUCACAGGGUUGUACAGACUGGGGGGUGUCCCCUGUGCUGACCCCAUGCCCGUUUUU

UUUCUGCCACAGGACCGUAGCCUGACGGGCAAGCUGGAACCGGUGUCUCCCCCCAGCCCC

CCGCACACUGACCCUGAGCUGGAGCUGGUGCCGCCACGGCUGUCCAAGGAGGAGCUGAUC

CAGAACAUGGACCGCGUGGACCGAGAGAUCACCAUGGUAGAGCAGCAGAUCUCUAAGCUG

AAGAAGAAGCAGGUGUGAAUGGGCAGGGGGAGGGGGAGUGUUUGUUCUGAGUCUCCAUUC

UAGCAGCAGUGACAGCGGUGUCACAGACAUGUGCCAAGCCCUUCCCAAGUUCCUGGGUGC

UCACUGGUUUUUAUGCCCUUUACGUUUAUUGGACAGGAGCACAUCCACAGAACUUUCUGC

CAUGAUGGAAACACUGUGUAUCUGCAGGAUAGCCAUGUUCCACUGUUGAGCAUUUGAAAU

GUGCCUACUGCGACUGAGGAACUGAUUUUUAAUUUUUAUUUAAUAUACGUAUUAAAUGUG

CCUUUGAAUAGCCACACAGGCUGCCGCAUUAGACAGCACAAGAAUGGAGGGUGAAAGUUC

AGGAUUUCUAGCCAGUCUGUCCAGGUUCAGAUCCUGUUUCUUCCAGUACCAGGCAACGUG

GGGUGGGCAGUCCCUUCUCUGUGCUUCACUUUCCUUAUCUGUAAUGUGGGGAAACAGCCC

CUGGCUCUGGAAUCUGAAUGGACUGGCCUCCUAACUGCUCUUUACUAGCUGUGUGGCCCU

GGAACCUUCAUCUCUUGGAGCCUUGGUUUCCUUGCCUGUAAAAUGGACAACUUGUAGAGG

AGGGAGCAAGAUCCCGAGAUUAAAAAUAAAUCCUCAUUGUCACUGUUGAAACCACACAAA

GACAAGCGGAGAAAAAGCUGAUAUAAAUAUUUAUAACAUUCAUUUUAUAACUUUGUUUUA

AAUAUGAGUUAAUAUACAAUUCUUUGAUAAUCUGCCUUUUCCCAGCUCUUCAUGGUUUGC

UCCCUGGUAUCAUUCAGGUCUCUGCUCAGAUGUCCCUGACACCAAGAGGCCACUGUCAGC

CUUGUACAGGAACACACACCCCCAACCCUCCUCUAUCCCGGGUUCAGCCAAUCCAGCCAA

UUCUAGCUGCUGCUGGUUUUUGUAAAUAAAGUUUUAUUGGCACAGCCACGACCACUUGUU

UGUGUAUCUGUAUACACAUGUGUGUUUAUAUGACUGCUUUUCUGCCACAGUGGCAGAGUU

AAGUCAUCACAACAGAGACCUGUGUGGCCUGCAAAGUCAAAAAUAUUUGCUGUUUAGGCC

UUUAAAGAAAAGCUUUGGGCUGGGCAUGGUGGCUGAUGCCUGUAAUCCCAGCACUUUGGG

AGGCUGAGGCUGGAGGAUCACUUGAGCCCGGGAGUUUGAGACCAGCGUGGGCAACAUAGC

GAGACCCUGUCUCUACAAAUAAUUUAAAACAUUAGCCAGGUGUGGAGGCACAUGCCUGUG

GUCACAGCUACUCAGGAGGCUGAGGCAGGAGUGCCUGAGCCCAGGAGGUUUAGGCUGCAU

GAGCCAUGAUUGCCACUGCACUCUUGCCUGGGCAACAGAGUGAGACUCUAUCUCAACAAA

AAGAAAUUUUGCUGAUCACUGAUCUGUCUCAUCACCCUGCUGGUGACAUCAUUAUUUGAA

UUAAUAUAGACCCAGCUUUGUUAUACAGUGGUCCUCUCCUUGUCUCCCUGUUAACUGCUG

UGUUCUGAGCAGCAUGGGGUACAUAGUAGGUGCUCAUUAAAAAUGGUUGAUUGAGUCAUU

GUGACACUGUCUGGGCCCCAGGGAGCGUCUAGACUAAUGGGAGAAACAGGCUCAUAUCUA

GUGCCCUCCAGACAAGGAAGUAGGGGCUCCAAGGAGGAUCAAGGGCUCAUUUUAGCCACU

GUAGGCAGGUGGGUGAAGGCUGGUGAGAGUAGGCUUCCAGGAAGAGGUGCCCUGGUAGCU

GGGUUUUGAAGGACGCUCCAGGGGGAGAGCAUUGAUUUCAAGUGUCCAUCGUGGCUGUCA

CGUGGUUGGGGGAACCAGGAUUUGAGGAUAGUCCAGCAGUGAGACCCGGUCCUGGAGACU

CUGCCAACUGUCCUAGGCUUCAGUGGGCAGUGGGUAGGUCUCAUGUUGGUGGCACAGGCA

UCCCGAGAGGGGCCGGGCCCCAGCGGCGUGGGCAGGCAUUGCCAGGGCCAGCCUGGUACC

GCACCGUUAGGCUUCCAGGCCUGGCCUUGGGGUGGGGCUGAAGCUGCGGGUCACUGGCAC

ACAUUGGUGACUGCAGCUGGAAGCCAGGUGGCUCAAGGCAGCCCUCAAUGGUUAGGUUUU

UGUUUGUUUGUUUUUUCCCCUCUAAAUUCCACUUUGGCAUUUUUUAAAAGUGAAAUUCUC

AAGCAUAAUUAUUUUAAAGUGCACAAUUCAGUGGCAUUUCGUACAUUUGCAGUGUUGUGC

AGCUGUCACCUCUCUCUGGUUCCCAGCCAUUUCAACAACCCACAGGGAAGCCUCAUAGGA

GCUAAAGCAGCCACUCCCCGUUCCCCUACUCUCUGUCUCUAUGGACUGACCUGUUCUAGA

CAUUUCCUAUAUAGGGCAUCACACAGUAUGCGUCCUUUCUGGCUUCUUCCACAUAGUGUC

AUGGUUUUGGAGUUUAUCAUGUUGUCUCUUGUCUCCAUCCUUCAUUCCUUUUUAUGGCUG

AAACAUAUUCCGUUGUAUGGAGAUGCCUCCUUUUGUUUAUUUCAUCAUCUGUGGCCAUUU

GGGCUGUGGCUGUUGUGAAUAGUGCUGCUGUUGAACAUUUGUGUUGAAGUUUUUGUUGGG

ACACCUGUUUUCAGUUCUCAUGGGUGUAUACCUAGGAGCAGAAUUGCUGGGUCGUGUCGU

ACAGUAACUCUAGGUUUAAUUUGCUGAUGACUUUCCAGGCUGUUUUCCACAUCAGCUGCC

CCAUUUUACAUUCUUACCAGCAAUGCAUGAAGAUUCCUGGUUUCCUGGUUUUCCACAUCC

UCACCGACCGCCCCCCAGCUUUUUUUUUUUUUUUUUCUGAGAUGGAUUCUCACUCUGUUG

CCCAGGCUGGAGUGCAGUGGUGCCAUCUUGGCUCACUGGAGCCUCUACCUCCUGGGCUCA

AGCGAUCCUUCUACCUCAGCCUUCUGAGUAGCUGGGAGUACAGGUGUGCAUCACCACACC

GAGCUAAUUUUUGUGUUUUUUGUAGAGAUGGGGUUUCACCAUGUUGCCCAAGCUGGUCUC

AAACUGCUGGUCUUGAGUGAUCUGCCCACCUUGGCCUCUCAAAGUGCUGGGAUUUCAGGU

GUGAGCCACUGUCCCUAGCCACACCAACCCUUUUUUAUCUUCCUUUAAAAAAUGUUUUUG

AUUCCAGCCAUCCUAGUGGUUGUACAGCGGUAUCUCAUUGUGGUUUUGAUUUGCAUUAGA

GAGCCCUUUAUAGCGUGUCACUGCUGUGCAGCAGUUUUUUCAGCAUUAUCUCCUUGAUCU

UCCAAACAACCUUAUGAGGUAGGCAUAUACCCACAGGUUCUGCUGAAGUAGACACUGAGG

UCAGGGAGGUGAAGGGCCCUGGCUGAGGCUGCAGAGCUGGUGAGCGGGGGGCUCAGGCUU

GAAUCCAGGCCCAGCAGAGCUAGUCACCCUCUUAGCUGGGUCUCUGUAGGUGUCCCAGGU

AGUAUUUUCUCUAGUACCUGAGGAAGGGCCACCUUCCCUGGCUGGAGGGAGCCUCCCUCC

UGCCCACCCUUUCGGGGCUGUGGGCUUCUUCCCCUUCUUGGCUUUGUUUGUUCCGUGCUU

UGGGGGAGCAUUUUGAUGCCCUUCUUGAAGCGACCACCAGGGGUCGCUGUGGGACUGAGG

CAGUUCUGCUCAAACCGCCAGCGAUGGAGUCUGUAAAAGCCAGCAUCCUGGAUUUUCUGG

CUUUUCCUGUGUUCACUUCUUCUUGGGCACCUUUCUGGGUUCAUACUGAUCUGCUCUGUG

GUAAUGUGCAGGCUGUGCGAGCGUCAUAAGUGGAGCAGAACCAUGUUCUGAUCUCACUUG

UGAUGCCCUGUCACAUUGCAGAAUGGGGCAUUGGCCUUCCAUCUGACCUUGGGCCAGUGC

CCUAAACCCUCUGGUCUUCAGCCUCCCUCUUGCUAUGAAGCAGACGGCAGCAUGAUGGGA

UGCUCUUUCCAACUGUGUUAGGAGGAGGGGGUUCAUACAUGGACAGAUGAGGAUGAGGAU

GAUGGGGGUGAUGAGGAUGAGGGUGAUGGGGGUGAUGAGGAUGAGGGUGAUGGGGGUGAU

GAGGAUGAUGGGGGUGAUGAGGAUGAGGAUGAUGGGGUGAUGAGGAUGAGGAUGAUGGGG

GUGAUGAGUAUGAGGAUGAUGGGGUGAUGAGGAUGAGGAUGAUGGGGUGAUGAGGAUGAG

GAUGAUGGGGGUGAUGAGGAUGAGGAUGAUGAGGGUGAUGAGGAUGAUGAGGGUGAUGGG

CGUGAUGAGGAUGAGGGUGAUGAGGAUGAUGGGGGUGAUGAGGAUGAGGAUGAGGGGGUG

AUGAGGAUGAGGGGGGUGAUGAGGAUGAGGGGGGUGAUAAGGAUGAGGGUGAUGAGGAUG

AGGGUGAAGAGGGUGAUGAGGAUGAGGGUGAAGAGGGUGAUGAGGAUGAGGGUGAUGGGG

GUGAUGAGGAUGAGGAUGAUGGGGGUGAUGAGGAUGAGGGUGAUGGGGUGAUGAGGAUGA

GGAUGAUGGGGGUGAUGAGGAUGAGGGUGAUGGGGUGAUGAGGAUGAGGAUGAUGGGGGU

GAUGAGGAUGAGGAUGAUGGGGGUGAUGAGGAUGAGGAUGAUGGGGUGAUGAGGAUGAGG

AUGAUGAGGGUGAUGAGGAUGAGGAUGAUGGGGGUGAUGAGGAUGAGGAUGAUGGGGGUG

AGGAGGAUGAGGAUGAUGAGGGUGAUGAGGAUGAUGAGGGUGAUGGGCGUGAUGAGGAUG

AGGGUGAUGAGGAUGAUGGGGGUGAUGAGGAUGAGGAUGAGGGGGGUGAUGAGGAUGAGG

GGGGUGAUGAGGAUGAGGGGGGUGAUAAGGAUGAGGGUGAUGAGGAUGAGGGUGAAGAGG

GUGAUGAGGAUGAGGGUGAUGGUGAUGAGGAUGAGGAUGAAGGGGUGAUGAGGAUGAGGG

UGAUGGGGGUGAUAAGGAUGAGGAUGAUGGGGGUGAGGAGGAUGAGGAUGAUGAGGGUGA

UGAGGAUGAUGAGGGUGAUGGGCGUGAUGAGGAUGAGGGUGAUGAGGAUGAUGGGGUGAU

GAGGAUGAGGAUGAGGGGGUGAUGAGGAUGAGGGGGGUGAUGAGGAUGAGGGGGGUGAUA

AGGAUGAGGGUGAUGAGGAUGAGGGUGAAGAGGGUGAUGAGGAUGAGGGUGAUGGGGGUG

AUGAGGAUGAGGAUGAAGGGGUGAUGAGGAUGAGGGUGAUGGGGGUGAUAAGGAUGAGGA

UGAUGGGGGUGAUGAGGAUAAGGAUGAUGAGGGUGAUGGGGGUGAUGAAGAUGAGGGUGA

UGGGGUGAUGAGGAUGAGGGUGAUGAGGAUGAUGAGGGUGAUAAGGAUGAUGGAGGUCAU

GAGGAUGAUAAGGGUGAUGGGGUGAUGAAGAUAAGGAUGAUGGGGUGAAGAUGAUGAAGA

UGAGGGGGGUAAUGAGGAUGAAGAUGAGGGGAGGGAUGAGGAUGACAGUGAUGAGGAUGA

GGACGAUGGGGGUGAUGAGGAUGAGGGUGAUGGGGGUGAUGAGGAUGAGGAUGAUGGGGG

UGAUGAGGAUGAGGGUGAUGGGGGUGAUGAGGAUGAGGAUGAUGGGGUGAUGAGGAUGAG

GAUGAUGGGGUGAUGAGGAUGAGGAUGAUGGGGGUGAUGAGGAUGAGGAUGAUGGGGGUG

AUGAGGAUGAGGGUGAUGGGGGUGAUGAGAAUGAGGAUGAUGGGGUGAUGAGGAUGAGGA

UGAUGGGGGUGAUGAGGAUGAGGGUGAUGAGGAUGAGGGUGAUGGGGUGAUGAGGAUGAG

GAUAAUGGGGGUGAUGGGGAUGAAGAUGAGGGGGGUGAUGAGGAUGACAGUGAUGAGGAU

GAGGACGAGCAGGGUGAUGAGGAUGCAGGUGAUGGGGUGAUGAGGAAGAGGAUGAUGGGG

GUGAUGAGGAUGAGGGUGAUGGUGGUCAUGAGGAUGACGAGGAUGAUGAGGGUGAUGAGG

AUGAAGAUGAUGGGGGUGAUGAGGAUGAGGAUCAUGGGGGUAGUGAGGAUGAGGAUGAUG

GGGGUAGUGAGGAUGAGGAUGAUGGGGGUGAUGAGGAUGAGGAUGAUGGGGGUGAUGAGG

AUGAGGAUGAUGGGGUUGAUGAGGAGCACCUGUGUGUCAGGUGCUGCGUGCUUUGCACGC

AUCAUGAGCUCAUUUCACCUUCACAACGGCUCUCUAGAGUAGUAACUCUUUUAUCCCCAU

UUUCCAGAAGAGGAAGGUAAGGCUUAGAGAGGUCCAGGAGCUUCUCAAGGCUACGCAGUA

AGUAGGAGUGGGAGUUGGGCUCCAGUCUCACCAGUGCAGCUGUGGAGUCUGCACAGCCUC

UCCUAGCACUGAUGCUGCCUGUAGGAACCGUAAUGGGUAAUAAUGCCGGCUCGCAGCAGG

CCUGACUGUGCCUGCCUGUGUAACUCAUUCAGUUCUCACCACAGUCAGAAUGGCGGCACG

GCAAUCAGCCCCGUUUACUGAUGGGGCAACUGAGGGCUAGCAGGGUGAGGCUGCUUGGCU

GUGCUUCCCUGGCUGGCAAGUCACGAAGGCAGGAUUUGAACCUGGCUUUGGAGCUUGGAG

GCUGCAGCUGGCCCUGGAGCUGGGGCCUCUCCCGGGUGCGGAGUCCCUGGGGUGACUCAC

AGGAAAUGUGUAGCAGCCUGACUUCCUGUGCGCGGCCGUGGCCCGGGCUGCAAGGGGGCC

UCCCCGCGCCCUGGGGGCUGCAGCCCUCCUUCCGCCCGCUGGGGUGCUCAUCCCUCACGU

UCUGCCUCUCCCCACAGCAACAGCUGGAGGAGGAGGCUGCCAAGCCGCCCGAGCCUGAGA

AGCCCGUGUCACCGCCGCCCAUCGAGUCGAAGCACCGCAGCCUGGUGCAGAUCAUCUACG

ACGAGAACCGGGUAUGUGUCCCCGCCCUGGCCUGCUGCCCCCCGGUGCUGGCCAUGAGGC

GCUUCACAGGGGGCACCAUGAAUCAGGCCUCAGUGUUUCCAUCUAUGUGGGGAGGGGACC

UCGCCACCCUCUCCCCCAGAGACGCAGGGUUUAUGAGUGGGUGGGGGAAGGUGACCGGUG

GAAAGCCAUCCUGGUGUGGCCUCUGGGCUGUUCUGCUCAGACACUCAGAUGGGUGUGGAC

UGUGGCUGGUCAGCAGGAUGCAACACAAAACAGAGGUUUUGGGCUCCAGGAGAGCACCAG

GGUCCAGCUGAUGGCCAAGGGUAAGGGGCUGCCACUUUAGGUGCUGGUGCAGAAGAAAUC

CCUUUAAGGCAAAAGAUAAUGACAUUAUGACCGCAGCAGUGAGGUUUCAUGGAGCAGCCC

CUCCUGUCAGUCCUGGAAGGGCAGGUACAGGUGUCAUUUCCAUUUUCCUAAGGCCCAGAG

AGGUUAAGUGACUUGUCCAGAGUCACACAGCUAGGAAGCAUUGGACCUGGAUUUGAACCU

GUGGGCUGUCAGACCACAGGGCCUGAGUUCUCAGCACUGGCUUCCACUGACACUGGGCCC

UGGAAGGGGCCUUUGAGAUGAUUGAAACACAGAGUCUGGAGCAGGCUGUGGAGGCCCAUG

GAGAUCCCACUGGGGGCAGCUAUAGUGGGUCUGGUCUUGCUGGAGGGGCUUGGGAUGCUC

AGAGCAGUGUCUCCAGGGGCUGGACCCUAAGGGUCUGUGCACCCCCAAGGUGGAACCUUG

UGUCUGGUGAGGUGGUAAGCUCCCCAUCUUCCCAGUCAAGCCACUACCAGCUGGGCUUUA

AUGAUUUACAGUGUACUUUUUCUAAAUGUACUCAGGAUCUGUGGAUGUUGAUCCCCUCCC

UUAUUUUCUGGAUGAGGAAACUGAGGCCCAGAGAGGGACUGGUCCUUGUGAAGCAUGACA

GAUGAGGAACCUCCUCUCCCUGCCUUGGCUUCUCUGGUUCCCCUCCUGCCUGUAGUCCCA

GCCACCCUUCCCAGCUGCCUUUUCUGUAAUAUCUUCCUUGGCCAAUCAGUGCUCAGAGUU

GAGGUGGGGGUGGGGAGGCCUCGAGUCACGUGGUGGGUGCCUGCAGUGGCAGCCUGAGGC

AGGGAACUACUGGAUCAACCUGUUUGGCUAUGCCUCCACCUCUAGAAACAGCGAUGUCAU

GCAGAAUGAGACAAGCCUGUUUGGGGCCUGGGAACUGUGUGCCCGGCUCACUCUCCCACA

CCCUGACCCCUCACCCCAAGCACCACUAGUAACAGUCAUAGGAGAGCCAAGGGGCCACAC

AUCCUUCAGCCAGCCAUCCUGGGUUCAAAUCCCAUUUCUCCCACUUCCUGGCUCUGUGAC

CCUGGAGCGUUGUACUUAAACCUUCCUGUGCCUCAUGCUCCACAGCUUUAAAACAAUGAC

AACGACGGCAUCACCUAGUACAGUUGGUGUGGGGACUAAAGGGGUAAUUUCCUAGUGCCC

UUAGGACAGACAGUGUCUGGCACCAAAGAAGUGCCAGCUGGUGCUAUUAAUUGUCAUUAU

CAUAGCUCUCAUUUCUUCUUGUGUUUGUUUACCUUGUGCAUUUAAUAGAAUACUAUUUGC

AUUUAGUAGAACUACUGUUAUUCUCUCAAUUUUGCUCAUCAAGAAACCAAAGCUCAGAGA

GGGAAGGAGACCUCCCUGAGAUUGCAGAGCAAGUGAGCUUUGAGCCUGGGACUUGGAGCC

AAGAUUGAGAUGGCUCCAGGAGCCCAAAGUGGAGGGGACAUGGGCAGGGCUCCUGAGAGU

GUCACCUCUACCUGCCCUCUCUGAUCCUCGGUGGAGAGCAGACACGGUUCUCUCCAGCAG

CUGUGGAGGCCAGGUCAGUGGCCCAGCACAUUGCUGCAGUGCCUUGGCCCUCUGCCUGGA

GCUCCUCUCCUUGCAAGGCUCAUACUGUCACUUCCCUCUUUGCUGAAGUGGCCCCUUCUC

AGCAAGACCCGUUUCAACCCUCUACUUAAUACUGAAAACCCUUUUUUCAGGAAAAGGAGU

UUUCAGGAUGACACCCGUUUUCCUCCUUUGUGGCUUCUGAUUCUCCUUAGCCCUUGUCCC

CAUGGGACAUGAUUUCUGUUUUACUUAUUUAUUCUGUUUAUUAUGAAUGGAAACUCCGUA

AGGGCAGGGAUUUUAUUCCUGCCCACUUUUAUUUCCAGUGGCAUCUCCAGCACCUAAAAC

AGUGCUCACCACACGGCAGAUGCACAUUAAAUAAUUAUUGUGCAAAUAUUCAGGCGGCAG

GAAGGGAGGGGAGAUGGAGGCGGGUGGCCAGCUCUGGCCUGUGGAAGAGGCCUCUGGUUU

GGGGGCGUGGGGGCUGGUGGGGAUCCUCCGCCCGGGCUGAGUCUCUGAGCCUCCAAAGCC

UGCAGCCUAAUGUGCGGGAGGCCCCCAGCCGCCUUUGGCAGGAGCAGCCAGGACUUACCG

CCCCUUGGGGGAUCAGUAGGAUUAAAUUUUAAUCCGCCUUCCUUCCCUGCUGGUUCCUGG

AGAGAUGGAGGACAAGGGUCUGUUUUUCGAGCCAGCCGGAAAACGCAGCUUCCCUGCCGG

GCAGGGCCCGCCUGGCAGUUCUGAGUGCUGGAGUCUGGCUCAUUAUCCAUUCGGAGCACA

UUGGUGAGGCACCCGCUGAGCGAGGCUUUUCCCGCCUCCCCGCCUCCUGGGAGCUGCCUG

CGUGGGUGGGGGCCGGGCAGGUGUGCACAGAUGGCAGGGCAGCCAUCAGGGUGGCUGCAG

CCUGAGAGCAGGAGCAGACGGGGUGGCCUGGGCCCAGGAGGGCAGAAGCGUGACCUGGAA

GUGGGUGCUGGGAAAGGCUUCUUGGAGGCAGCGGGUGGGCCGGAAGGAGGGCGUUAGGGU

GAGGGGGCAUGGCACAGGCCAAGUCCCAGAGAGGGGACUGGCGUCUCCUUUGAUCUUCAA

AGGUGGUGGGCACGAGGUAGGAGCCUGUUGGUUCCGGGACAAGGCACGCACUUGGAAUCU

GGAUAAAACAUACACAGAGGCAGCGCUAGCGAUGUGUGGAUACAUAAGGGGCAUUUGUGC

AGCACAGGUUGCUGGUACCGUAGGAAUUCAGGACACUGGGGAACAGUCAGGAUGGGCUUC

AUAGAGGAGACGGGCCUUGAGCUAGGUCGACUGGGGAAAGGUAGAAGGCUGCUUCCAAUA

GAGAUGUAGAUGGCUCAUUCACAAGGUGGAGAGGAGGCAGGAUUGAUGGAAGCGGCAACC

CCAGUUGCUGAGUAAUGCGGAAAUCAGCAGAAGACUCAGGUCCUUGUCUUCCAGUUUAAG

CGUCUUAAAAGGAAAGUGUUUAUAUGAUGUGAUUAGGACUCAGUGUGUGCACAGUGUGAU

CGGGACUGAGCAGGGCAAAGAAGAAUAAUACCAUUAACAGUCACCGUGAACGUGAAGCAU

UCAGUAGAGCCUUUUGGGGGUUUUGGCACUCCUCAUGCUUGUUUGUAGGGCCCAGCAUCA

GAUCCCCUGAUUGAAGAUUUUCAGAGCAGUGCUUCAGACCCCGUCCUGUGGCCUCAGCAU

CCUGGGCCAGGCCCCCACCUCUGGCCUCCAUGCCCGGCCACCUGGUCAUCAAAACUGCCC

CCAGGGUCCAGGGGUUCUUUCAGAUUGUCCGUUUCAGCCCCGCGAGGCUGCUGCACGCGU

CCUCUGACCUGCCGCUUUUCCUGCUCGUCACCCACCCUGGGAGAGCUCUGCACGGGGCUC

AUGGGCAUGGGGCUCUGUUUGCAGAUGAGGAAGCUGAAGCUCCGGGUAGGCCGUUGCCCU

GAGGCACUGCUGCAGCCAGCGGAGGGGCUUGGGUGCAGGACUGGGGGCUUGGGGCUUCUU

GGGUGAGUAGGUGCUGCCCCCAAUCUGCAGAUGAGGAAGCCAGUGUGGCAGAGAAGGGGC

UAGGAUGGGGCUUCCAGGCAGGGGAGAGGCUAGGGAGUGAUGAGUGUCAGGUCUGUUUCU

GUGCUCUAGAGGAGAGCCGCAUGUGGAUGUAUGUGUGGUUUGUGUGCCUGUGGGUACACG

UGUGUAUACGGGUGGAUGUAUGUGUGGUAUGUGUGUUAGAUACACGUGUGCCUUUGGAUG

GCUGUGCAUACUUGUAUGUGAAUACUUAGAGUAAGAGUCAGCUUGUGUGGGACGAGCAUG

UGGUGAUUCGUGCAUUGAUACAGCACACUUGGGUGUGUGUAUGCGGGCAUGUGUGUAUGU

GUGGACCUCUGGAUGGCUGUGUGCACAUGUGUAUAAGCGUAUAUGAGUCAGCAUGUGGGG

CCUGUGGUUAUCUGUGCACUUACACAUCCAUGCAUACACACGUGGGUGCGUGUAUGUGUG

CAGGUAUACCUGUGGGUGCUGUGUGCACAUGCGUGAACAUUCAUCCACGAAUUGGCACAU

GUGAGGCAUGUAUAUAGGUAUUCGUGCAUCUGUGCACCUGUGUGCAUACGCAUGUGUUCA

CAUGGGUACACGUUAGGCUGUGUGCAUGUGUGUACAGGUGGAUGUGCGUGAGAUGGCGGG

UGUGGGGCGGGUGUGGUUACAUCAUGUGUUCACUUGGGUGCACAUGUGUAUGUGCGUGCG

CCUUGAUGUGUGUGGACAUGUGUGUAUGUCUUUGCGUGUUCCUAGAUCUUGGCAUGUGCA

CUUGGUAACGUGGGUCUGGUGCUCCCGUGGACCCGUCUGCAUAGGUGACUAAGGACUUGU

GUGCCCCAGCACAGCUCCUAGGCUUUUUGGGCAUGGGAGGCCUGGCCACGCAGACCCAGA

GUCUCUGCAGGGGUGCCACUGCUCGAAUGAGAACGUGCCCGGAUCUCAGUUGGCUGGCGA

GAGCCCCUACAGGGGGUGGACGCCACGGUGAGCGGGGCCGCUCAUUGGCUUGAGUCCCUA

GGUUUUCCUUUCCUCUCAGAGCCAGGUGAGCUUGGGCAGUGGCUUCUCUCAGGCUUGGCC

ACCUCCCCUGCGGCGUCAGCCCCACCCACCACGAUCAGAGCAGAGACCUCGGCCCCCAAA

GGCCAGGAACGUGGGAGGACGUGCGUGGGGGCAGUCGGGUUUCAGCGGGAUUGAGAAAGG

CCUGUUUCCCUUCUCCCCCAGGUUGGGGCUGAGUCCCCUGAAUUCCAGCCUCCCAGCCGG

AAGGUGGCAGCCCAGCCUGAAGCCCUCCUUUGGGCCGGAGCAGUCCCUGAGUUUUGCCAC

CCUUGGGUGGAGUUAGUUCAGCUCUAACAAGUGUGUCCUGGCGUCCCCUGCAAGCUGGGC

CUUGGGCCCGCAUGGGGAAGAUGCAGACAGGAGCUCCUGUUGGAGCACUUGCUGUUUGCC

AGGACCAGGGGCAGCCAGGGAGACAGUGUCGGGAGGUGGCCCCCAAAGGGGCCGAGGAGA

UGUGGAGAUAUCAGCCACGGGAAUAUUGGGGUUGGGGAAUGUUCUAGAUGGGAGAGGUUC

AGGUGGGAAGGGUUUGGGAUUUUGCCUAGAACUGUGUGGAUUGGUGACCUCCUAUCCAAG

UGUCCUCUCCGAGCCUCAGUUUCCCCAUCUGUAAAAUGGGGCAGCCCCUGCACCCCAGAA

ACAGGUUACUGGGAGGCUUGGCAAUCCUGAUUGUGGCUUGGAGAGCAGGGAGACAGGGCC

UGCCCUCUCAUCCACCUCUAUGGAGGGGGCAGAGAGGACUCCGUGUCCUUGGAAAGGCCA

GGCACAUCCGGGAUGUGGACUGCUUCCCCCAUGGCCCUGGAGAAAGUGCCCACUGCCCUC

AACCCUGUGCUGGGCCCUGCUGGACGAUAGCAGCUUGGUGGCCAGCGCUGGCCUCUUCUG

GUCCUGGUGGAGAGGCAGGGAGGCCCUGGGCCAGCUGCAGCGACGUGGAGAGAGGACAGG

GUUCUGGGCUGGGACCGGACAGCCUGCAGGUACAAGAAUGCUUUGCCCCAGAGACCGCCU

GAGCGAGGGAGCCAGCCGCACUCCAUUUCCAGGUAUUUAGACCAUUCCUGAGCAUCCACU

GUGAGCCAGACACUGUCCAGGCACUGUGGGUGCAGCAGAGAACAGAGCAGGCACCUUGCU

CCUCAUGGAGUCCUCAUCAGGGGAGGUGGCCAGUAAGAUAAACAGUGGGACGCAGACCCU

UAUCCCUGACCGCAUGUGCUGUGAAGACAGCCAGGUGGGAAAACGAGUGGUCGAGGGAGG

UGGACCGCCCUGCUGCGGGGGGUCAGGGAAGGCCUCUGUGGGGAGGGGAGGCUGAGUCAA

GACAUGAGCAAGAGGCAGGCGAGAGCAUGUGAAGAUGUGGGGAACAGCAUUCCAGGCAGG

GAACGGUAGGUGCAAAGGCUCAGAGGCAGUAGUGGCUUUCAGUGUGCAGGGGACAGUGAG

GGGCCAGCAGAGGUCAAGGUCCUGUGAGGGCUGGGACCCUGUUGACCCUGGCGAGGAGUU

UGGAUUUGGUGCUCAGGGUGGCAGGAAGACAGCAGCCGAGGAGGUGAGGGGGAAUUUUUG

GCAUGAGGCUGAAUCUGUGGCCUGCGUCCACGUUCCCCAGCCAUGCCACGUUCUAGAGGG

UAUCUCCUCUGCAUUCAAAGAGGGAAGGGAUGACAUGCACUUCACUUUGCUCCCUGCAGA

ACGUCCUUGCAGAGGUCAUGGGAGUUUGGUACCCUGGGUGAGUGUGGCCGUUUCGUGAAU

GGGGAGACUGAGGCCAGACUCAGUCAGGAGACUCAGAUUGCUGAGGGUUAGAGCACAGCC

GACUCCCAGAUGUUGGGCAGGUGUGUUGAGAAAGGCCAGCCCAUCUUGGGACAGGGCUCC

ACAGGCCCACCUCCCCAGGAGGCUGACAAGCAGGACCAGAGAAGACCGUGGAGCAGGAAC

AAACCCAACACUGGGAGCAGGGGAUGUGGGGGCAGGAGGUGAAGGAAACACCCCAGAGAG

ACAGGAGGAGGUGGGGCUCCCUGUGGCUUUGGCAGGUGAAGAAGGGGUCUUCCUUGCUCU

CUAGGUGCUAGCUGUGUGGCCGUGUAGUUUCUAGUGGCUUGGCCCUUUCCGUUGGUAUCA

CUCUUUUAUCUAGAUGGACAGUUGCUGCUGAUCCCCACAGAUCAUACACAAACUCCUAUU

CAUCCCUCAAAACCCAGCCUAUAUAGCCCUCCUGUUACCUUUUUGGGAGAGUGACUUGUG

CCCCACUUUCACACAUACCUUUAUCUGCCUGAGCAUACUAAUCUGCUGUUACUGCUCUUA

ACCAACAGCCAUUGUUUGGGUUUAUUCCAGGCAGGGGCCAGGUCUCAUUCAUUCCAUGUC

UCUCCCCAGGUCCCAGUGUUUGGGAGCUGCCCUGCCCCCAUGCUACCCCAUACCCAGUCC

UUCACUGGGCCUGGCUUAGGGGUUCUCCCAUCUUCAGUACCCCAGGGCAGACUCUUUCCU

GCUUCGAGGGUGUGGCUGGGACAUUGACUCUCCGGGUGAUGUCUGCCCCCAUCCACCGGA

AAUACUAGUCCCAAGCCUAGGGGUGGUCCUGCAUGGGACCUGGCCCUUGGCUUCCUCUGU

CCGUGGAUCCUGGAGGUCAUAAGAAGGGCUUCUUCCCUGUGUUUUGUUUCUGUGCCUCAG

UUUCACCAUCUGUGAGAUGGGGCUACUCCUGCCUGUACCUCCCAUGGCGCUUAUGCGCAA

UUGACAUGUUAAGAACACACCGUAUGCCCGACGCUUGGCUCAUGGCAGGUGCUUGGAGAA

GUGACCAUCGCUGUAAUAGUUCUCCCCUGCUCUUCUGGCCCCCAGCCGCACCUUGAGGCU

GCAGGUCUCGGGGUUUUGGCUCAGCCUGAAGGUGGCCAAGAGCAUGGCUCUGCUCUUGGU

GGGCCGGGCUCUGGGCUGUAGGCGGAGGCAGCUCUAGGUUCCAGCUUGAGCCCACCUACA

UACCUGCUGUCUUUCUCCGGGGGCUGGGGAGGGGGAGUGAUGCUGGCCUGCCACGCCCUC

CUCUCUCUGCUGCAGCCCGGGAGCCUGGGCACCUCCGCCCUGCGAGGAGAUCUUGGCAAA

GGCUGCGUCAAAUUUUUGUUGGCAGCCACAUUUCCCAGGGCUUGGGGUCACCUCCUUUUG

GCCAAUCUGGUUUGGGGUCACUGGGGACAGUUUCAGUAUGGUGAUGGUGACUUCUGCGGC

CUGUCCGCCUAGUCCUUGCCAAAUACUGAGUGGCUGCCCCAAUGCAGCUGAGUCACAGCA

GGGGCUCGCCCGCGUGCCAGCCUGUGAACACCCAUUUGCCAGCACACACGCAGUUUAGCC

UGGUGCAGGGGGCACCCAAACCCACGCAAAUUCUGCCCCUGCCCUCCGCCACAGCCCCCA

UGAAGCCACCUUCAGGAGCCCCUCCGCCCCCCACCUCCCUGUACCCGGCUGGCCUGGGCU

GCCCUCCAGGCCUCCUGUGAGUGGACCCUGGUGGUCCCAGGCUGCGGGCUGGUGGGCCUG

GCCGAGGCACAAUGUCUGCCUUCACACCGAGGGCCGGGGUGGGGGCCCGGAGGUCCAGCC

UAUUGAUGGAGCGGCCACCGAGCCCUGCGCCACAUGUUCCUGUUUUCCUAAUAAGUCCCC

AGUUGUGAGUGGGGAGGAGGCGGGGAGGCCCCGGGGCAGCCCAGAAAUAAUCACAUGAUU

GUGAAACACAAGAAUCCUAGAAAGGGUAUCUCCGAGCGCCUCUAUCUCACCCUCCCUCCU

CCUCCUCCUCCUCCUUCCCUCGCUGGCUGCACUAGCUCUGAGGUAAUUGUAGACAACCCA

ACCAGCUUCCAGGGUGAGGGAAGGCAUCUCAGGGCUGGCAGUGUCGGCUGGGCUGGUGGG

GAGCGGUGCCCACGUCGCUGGGCCCUGGGGGAGCGAGGCCUGCGCGCCUGCCGGGGAGCA

GGCUUCAGUGGACCCGGCGCAUUUGCUAAAAUCGAAACUUGGCUGGCUUGCUGGGGCCGC

UGUGGGGGUAUUGAGGCUCCUCUGUGUUUUUUUCCAGCGUGGGGAGGGCCUGCAGGGGGC

CCCGGGCCUCCCUCCCCCGUGCUGAGGAUCAGCAUGCUGGACUGGGCUGGGCAGGUGCCC

GCUGCUCCGGGAAGCCUCUAUUUAUAAUCUGUGUUUUUUUGUUUUUUAAAAAAAUUCCUC

CUCUUCGCCAUCACCCUGCAGAAGAAGGCUGAAGCUGCACAUCGGAUUCUGGAAGGCCUG

GGGCCCCAGGUGGAGCUGGUGAGCUGGGGUACAGGGUCAGGGGCUCAGGGGAGGGCGGGG

GUGGCUGGAGAGGUGGGCAGGGAGGCGGGAGGGUGCGGCGGAGGAGGAAGUCAUCUAUUA

CCAAGCGCUGCCCAGGCGGCGUCCGCGGAGGAAGCAGGAAACCCACCUUCCUGAUGAGAG

GACGGCAGAUAGCGGGUGGGCGGGCCUGGCUGGGCUGGGUGGGGGUGGGGAGUCUUGGCA

GCGAGCGCCUCCGGGGCCACCUGCGCACUCCUGUGCAUACCGUGAAGCCCGGCCGCUCGG

GCUGUGAGGAGAGUCACACAUCCGUAGGCCGGUGCGCUUGCUCCAGCUGAGCGCGGAGAG

CCGGUUCUCUGGGUCACACAAUCUCGGUCCACUCCCAGGUGCACCGCUUCAUGGCGGUGU

GACCCUGAGCGGGUCUGUUACCCUCCCUGUGCUUCAGUUUCCUCAUCUGUAAAAUGGGAA

UCCUAACAGCACCUAUUCCACUGGAUUACAGUGAGGAGGAGCUGUGUUAAUAUUUUUCCA

ACAUCGGGUGCCGCGCCUGCCUUGUAAACAGUAAGUGCUGGGCAUUAGCUAUGAUGAUGA

UGAUGAUGAUGAUGGCGAUGAUGGCGAUGAUGGCUGUCACUAGUGUCCUUUUGCUGCGGA

AAGAUGCCAUGUCAGCAGCAGAGCUGGAAAGCAGGCCCCAGGCCUCCUGGAGUCCCUGGG

CUGAGGAUGGGUGGAUGCAGAAGCCCUAGCCCCCAGAGAAUGGGCCGCAGCCCCUGCCUG

CCCCACAGCCAUCUGUGCCCACUGAGCUGGUCAGCAUGGAUGGCCGUGGUGGGCACUGAA

GGUAUAUGCUACCCACAUGUGUGCCGGAUGAGGGAGAGUCUCUGUGUGCAUGGUGGGCUA

GGGUGUGCGUGUGAGUGCGCCCAGGGUGCAGAUAAAGGCUUAUGCCUUCGCCAUGUGUAG

UGGCUCAUACCUGUAAUCCCAGUGCUAUGGGAGGCCAAGGCAGGAGGAUUGCAUGAAGCC

AGGAAUUUGAGAUCAGCCUGGGCAAGAUAGCAAGACCUUGUCUCUGCAAAAAAUUAAAAA

AUUCACCAGGUGUGGUGAUGCACGCCUUUCGUCCCAGCUACUUGGGAGGCUGAGGCAGGA

GGAUCGCUUGAGCCCUGGAGUUCAAGGCUGCGGUGAGCUAUGAUCACACCACUGCACUCC

AGCCUGGGCAGUAGAGUGAGACCCUGCCUCAUAAAAAAACGAAACAGAAGUCUGACACCU

UCAGAGCCUCAAUCUAAACUCAGCUCCACUGCAUGUUUGCCAAGUGGCCUUAGGAAAGGG

GCUGACCUCCCAGUACCACAGUGCUCUCUCAUCUGGGACAGGCGGAGGCUCUAGGGCCUC

CUUCCAGGGUCUUCCCAGGGUUAAAGGCAGUGCUGCCUGUUAAAGGCUCACCUAGUGCAG

CUCCUGGUCCCUACGAAGCGCUCAGUAGUUGUGAACAAUUUGGUGAUGAGCUGCUCCCCC

UGAAGUUUCUGCAGGCUGCUCAGUUUUGCAGUGAGAGCUCCCCAAAGCCCGGGGUCCGUC

UUUUGAGGCCACUCAGGAACUACAAGCCCCGGGCUCCCAGGCCCACUCAGAAGGCAGAAG

CAGGGAGGAGAUGGCAAGGGCUCCUCCAGCUCAGAAGCAGGGCCCAGCUGCCCACCUGGC

UGGACUCAGGAACCUCGCUGGGCUCCCCCACCCCUUCAGGGCUCUGCCCCCUCCAGGCAC

AUCCUCGGGCCUGGGCGAUCUGGGUGGUGAGCUCUGGCCUAGGGUCGUGCUUCUCUCAGU

GGAAGUGAUAUCUGGGGAAGUUUUCAGCAGCAGCAGCUGCUGCUUGCUUGUUUUUUUGUU

UUGUAAACAGCUUUAUUGAGGUACAAUUCACACGCAUGCCAUUCACUCAAAGUGUCCAGU

UCAGUGGGUUUUAGUGUAUUCACAGAUGUGUGCAACUGUCACCACGGUCAAUUUUAGAAC

AUUCCAUCAUCUCAAAAAGAAACCCUUCUGUCUCUAUGGAUUUGCCUAUUCUGGACAUUU

CCUAUAAAUGGAAUCUUGCAAUAUGUGGUCCUUUGUGUCUGGUUUCUCUCACUCAGCAUC

AUGUUUUCGAGGUUCAUCCGUGUCGAGCGUGGGUCGGUCCUUCGUUCCUUUUUGUGGCUG

AAUCAUAUUUCACUGUAGGGGUAGGUCACGUGUUGUUUACCCACUCAUCAGUCGGUGGAC

GUGAGAUUGUUUGCAUAUUUGGGGUAUUGUGAAUAAAGUUGCUGUGAACAUUAGUGUGCC

GUUUCCAUGUGAACGUACGUCUUCAUUUCUCCUGGGUGUGUACCUAGGCGUGGAAUUGCU

AGAUCCUCUGUUAACCCUGUUUAGCCCCUUGAGGAACUGCCCAACUGUUUUCCAAAGUGG

CCGAAAAGUGGCCGUACCAUUUUACAUCUCCACCAGUAAGCUGCCUCUUGCCUCGGUUUC

UCCUUUUCUGCCAAAUAGGGAUGGCAGGGUUGGAUGAAGUUCCCAAGCAUUUCCAGAUUU

CCGGGUCCUCCCGCUCCCGUUUCCUGAUUGGUCCCCGGGGGCCUCCUGGGGGAAAGGGCC

UGUGUCUUAGGGCCCGAGGAACACAGGCGAGGAAGCCCCUGUCCGUGGGAGUCUGUGGGC

AGAUGGGGGUGGGCAGCUGCUGGUGUCAGAAGCUUUGGAACCAGGGACCCCACAGUGAGU

CUUCUCUGGUUCUUUGGAGACAAGGGGACGGGGUUGUCUUCCACUUGGGGAUCUCAGAGC

AGAGGCUUCCACAAGGCUGAUGGCACAGGAGGCUAAGAUCUAGUGUUUGGACUCUUGAAG

GCCCCUGGCUUGGUGCCAGUCAGUGGGGACAGGGAGGACGCCUCACAGAGCUGUUGCCGU

CCCAUUUUCUGGAUGAGGCUCAGUGAGAGGAGUGCCUUGUGCCGGGCCACACAGCAAGAG

AUGAGGCGUGGAGGUGUGCUGAGCCCCUGGGAGAGCGGCCGGCCAGUUCUGCACAGAUCU

GCCAGGCCCGAGUGGCCACAGGCAUCCUCCAAGCAGAGGCAGCUGCAUGGUGCACCCUCU

GCAGGGUCCUGUCUACCAACUGGGUGCCAGAAGCCCGGGCCAGGGACGGCCUGAGGUGCU

GUGGGUCACAGAGUCCAGUUCCCAGGGUGUCUUCCAUCAGAAGCUGCAGUUCCAACGCCC

CUCCCCUCGGCAGGGACGGAUCCCUGUGGCCUUCCUGGAGGAAUUUGCAUUGGAAAGGAU

GAAAAUAGCACCAGAGGUGCUAACCAGGGGUCGGGGGCACUGAGAUGGUGGGGCACGGCC

CCUCCAGCCCAGCUGUGGGGACUGGACCAGGCUGGGAGCGGGCGCAGUGGUGGAGGGCGU

GCCCAGCAGCCCCGACCUCUCUGCCGCUUUGAGUCAUUGCUUCCUUCCUGUUCCAAGGCG

UUAUUUUAAGAAGCUGGUGUCACCAGCGGGUUGAGGGCUGGGGCUGUCACUUCAUUUAUU

GCCAGCUCAGGCCGCCUGGGACUCUGGCUCUUCCUGGUGGCCCUGCUGGCUGGCUCAAAG

CCCGAGGGGGGCCGCCUCCUCCCUGGCCCCUGCUGGCUUUCUUAGUCACCACCAUGCCUC

AUCUCUGCCUGCGCCCUGCGUCUGGCACCUGCUCCCCUUUGCCCUCUUUCUGCCCUCUGA

GUGGCUGUGCCUCUGAUUUUAGAGGAGACGGAGGCUGAGCAAGAGAGAGCUGGACCUUGG

GGCCUCCUGGGUGCCGCCCCUACCACCCCAGGCUCCACACGCAUCUAGGCCUCGAUUCCC

UCACUGGCGUGAGUCCCCCCAGGGGUCAUUUCUCCCGGGAGGAUAAGGCCUGGGGGUGAG

GGACAGGGGCCAGCCAUGCCCCCCAGCCUGUGUGGCUGUGGAGGUGCCUCAUGGCCCCAU

GUGGGGACCCCGGCUAGCCUGGGCUGGGAGCCGCCAGGCCUUUGUGCAGCCCUCUCUGCA

GAGGAUGAGGGGGAUGUUGGGGAGUCCCCUCGGGCCCUGCGUCCCCUCAGCAAUGCACCU

GCCAGGACAUGUCACUGUCCCUCCCUCCCCCCACUCCCUUCUGGGCUUUCCAAGAUGGCU

GUCCUCAGCCUGUUGCCAUGGCGGUGCACGGCAUUCCAGGCAGCUCUGGGCCCAGAGCCU

CCCCUGGGCUCCUGGGGUUCAGGGGCCUCGGGGAGUGGCUGGUGAGUGCCUCCCAGGGGG

GCAGUUCCUGUUUAGAGGCCGCAGCACCCCACAGUAUCCUCAGUCCUUCGGGGCCUUCGC

CUUGUGGGCCCCUCACCGAUGGCCGCCUUGAGAAGCAGGGGCUGUGUCCCUAUCUACAGG

GCAGGGGAGGAGGCCACCAAGUGGAAGUGGCCUUCCUCCCCUGUGGCUUUGGUGGGUUGG

AGGCUGACCGGGCUCUUGCUGCCCCCGGGAGCCGGUCCUGCAUUUCUGCACCCGCCCUGU

GCCGCAGAAGCACUGUCCCCUCUGCCCACCUCGCCCUCCCAGGGCUGCGUGCUGUGGGCU

UCCAGCCCCUUUCUGGCCCCCUUCGGCUGUUUGCUAUCUUUAAAACCAAAACAAGACAAA

CACCAAAUUUCACCAAGUCUGAAAUUUUCUGGGCUAUCCAAAAUUUCCGUGAAGUAGGUC

CUCUUUGACCUACACCCAUAUUCCAGAUGAGGAAACCGAGGCUGGAGUGUUGAGGGCACU

UGUCCAAGCUCACAGCUAGUGAGGGAGGAGGCACCAUUUAAAUUGGGGCCUAUCUGUUUC

CAAAACCCGAACCUUUAAGUGAUGUCCGCUUGCACCUGGCCUGCAUUUGAUUGGCAUGUG

GGGAACAUAAAGGAGCCAGUGUGGUUGCCCGGGUUACAGCAUGGGCAUCUUCACAGGGCC

CCUUGCCAUUCCUGAGACGAGGAUUCGCACCCUCUGCAGGGAUGCUGCACUGCACCCCUU

CCCUCCACUGGGAAGUUCUGCAGUGAGUCUGACCGUGGGCUCUCUUGUUCCUCUGCCCAC

UUCUCCCUCUUGUUCAAACUUCCAGGGUCAGGGUGGGGAAACUGCCCUCUGUUCAGGGCU

GAGAACCUUGGGAGGGGAGCCUGACCCGGCACGGGGAGAAAUGGCCCAUGGCAGCUGCAG

GAUGACAUCUCUGUCCCCAGGAGAGUAGCUGAGCAGGAGCCUGGAAUUGCCCGAGCCCAG

UGGUGAAUUCUCUAGGAGGGGAUCUGGGGCUACAGGGGGAAAGGCGACAUUGUCACCAGC

CGAGACUGUCUGUGAGGCUCUGUAGAGCUGCAGAGGCCCUUCCCUGGGGAUGGGUGGGCG

GGGGUCUGGGUGGCCCACUCUGCCGGCGUCAUCAGGAGGUGUGUUCAGCUCUAUCCCCAG

GGAAGAGACUUGUGUGUCCCUUCUGUCGCUGCAGCCCUGCCCUGAGGCUGCAGGACUCCA

CGUCUCCAUCCUUCCCUGUUCCAUCUGAUCACUCACACUCUGUACCUGAUCUUCCCACCA

UAGUCACCCAUGGCCACCCUGGGAGGCUCCUGCUGUGUGCAUGGGUGUGUGAGAGGGAGA

GGGACAGGAAGAGAGGGAGACAGACAGACAGAAAGACAUACACACACACACACAGAGACU

CAGGCAGAGAGGGGAGCAGACUCCUUCCUCCAGCGCUGGGGCUGAGAGGCGGGCUGUCAU

UCAGGCUCAUCUCUUACCAGCUGUGGGCAAAUGUUGUAACCUCUUAAGGACUCAAUUUUU

CUUUCUGUACAGUGGGGGAUGAUUGUAUCAGUUACCCGGUAUGGUUACAUGGCUACUGCU

GGGGAACAAAGCAGCCCCACAUUUAGAGGCUUGAAGCAGCAACUGUUCACUGUUUCUCAC

CUGUCUGGAAGGCAGCUGAGCAUUUCUGCUGGUGUGGGCUCAGCUCUUGCACCUCCGUGG

CCAGCGAGGGAGUCGGGGGGCCGGCGUUGUAGAUCAGCCCAGGCUGGGUUGCACCACGCU

GCUCCUUGCCUCUCUGCCCGCUUCCUGCAGGCCAGGCUAGGCUCAUUCUCUUGACAGAAA

CAGCAGAAAUGCGAACUCGCAUCUUCAAGCCUCUGCUUUUGCUAAGUGUGCAGCAAUUCC

ACUGGCCACAGCAAGGUCAUGGGUGGUUACAGGGAGGCGUGAAAGGUGGGUGCCAUUAGC

ACAGCCACUGCUCCCUGAUGGCGGCAAUGUGACUGGCACCUCUGGGCACUUCAGCCUGCA

GGUACCAGCCUGAGAGCUUAGCAUGCACUGUCUCGUUUGAUCUUCACAAAAUCCUAUGAG

UUUAACCCCAUUUUAUAGAUGAGGAGAAAUGGAGGCACAGAUGGAAUAAGUAACUUCCCA

GGGAGACCCAGCUAGAAAGUAUCAGAACCGGGACUUGAACCUAGGCUGUGCCCUUGGUUG

CUAAGCAAGGUUCUUCCAUCUGGGAGGGGACACGUGGCAGCUGGCUUGGUCCCUGCACCA

UCACAGAUGCUGCCGUGCUUUCUGCGUCUCCUGGUACGCUCUUUGAAGCCCUGUGCUUGC

UACAUGUGGGCCCUGCCUCCCUUUCUCACUGCUGUGUGGGAUUUGUGGUGUAACCGAGUC

CUGGUCUCCUUAGCUGCUCUUCUGGUGGUGGACGGCUAGCUUGCUUCUCACUGGCCGCUG

UGAGCAGUGCGUUAGGAACAUCCUAGCGCCCGUCUCCUGGUGGAGCACAGUAGGCAACUG

CAGGAGCAUGGGGGUCGGAAGCGGGGGCGUCGGUGUUCGUUACCAGGUAGUGGCUGUUGU

CUCUCUGGGGGAUGUGUGCCCAUACACCAUGCUCCCACCAGCUAUGUGCCAGCCUUCCUG

UCACCCCAGACCUCACCAACACUUGGCAUUCAACGUUUUUGUCUUUUGCUGGUCAGUGGG

GGUGGAGUGGUAUCUUGCAGCGUUGGGACGCGCGUUUCCAUGGUGAGCUAGGAAGCCAAG

UGGCUUUCCACGUUUGGUGGCCAGGGCCAAUCUCUGGGGUGUGUGUGGGUUUGUCCGGGU

GUGCUCUGGGCUGUGGCCCCUUCCAGGCCAGCACUGCUGAAUGCCCCGCAGAGCAGCCAU

GACGACCUUGGUGGGGGUGGUCACAGUACCCACAGCUGUUCCCGAGGAACCAGUUUCAGC

CUGUUCCUGGCUGGGGCUGCGUGCUGGGUCCCUGUGUUCUGUUUCAUGCCUGCCUGCGGC

UGAGCCCUCACCCAGAGGACAGCUCCUCCGUGCCAGGGCUGUGGCUCCUACCGGUGGGCU

CUGUAGUGGCGAGUGGCCAGCCAGGCUCUGAUGUGCUUCUCUCUCUCCCUUUGCAGCCGC

UGUACAACCAGCCCUCCGACACCCGGCAGUAUCAUGAGAACAUCAAAAUGUGAGUGCUCG

CGGGCAGCCGUGCAGACACACAGAGGCAGGGUGGGCGAGCAGGCUCUCAGCAGCCUGCAU

GGGAUGUGGGACGUGGGCUCUCUGUUCCCAUCAGGGGCUCCCGGCCAGGGCCAGGUGCUC

CCGGAGGGGACAGGAGACAGUCCUAGGUGGCACCUGGGGUGGGGGUGGGGAGCAGAGAGA

GUGUCCCCCUGAGGUUUUGGGAGACAGGCUGGGUUUGGGAGGGUGGGCGAGGGAGCUGCU

GGUGCACAGGUGGGUCUGGAGAGACGGAGGUCAGAGGUCCCAGGCUGUACAUCUGCAUCC

UGUUCGAGCAGGAGGGAGCUGUUGCGGGCAGAAGGCAGCCGGCUCAGGGAGAUGGGGAGU

GGCCCCAGAGCAGGCGGGGGCCUGGUGUUUUGGGCUCUGGCAUAAGCAUGUUUGGGAAUC

ACCUGGUGUCUGCACAGAGAGGUCCCUCUGGCACAGCCCACUAUGGGGCAGAUCAGAGCA

CUCAUCCAGGCCCUGAGGCUUUGGAGGUGGCCAAUGUGAUGUAUUCUAAUGAUGGGGGCC

UUUUCCCAGACCGGUGCUAGGAGGGAAGAAAAUGUCAAUUAAGUGCCACUGCCAUUGGGU

GAGGCGGUGCUGGAGGCUGGUAGCAUUGUAUCUUCAAGCGGGGUACAAGGCAUUGGAGGU

GUGGCAGCUGGAAGGGGGCUGGCUUUUUCUUGGGAUGCAUUAACGGGAGCAUAGACUGUU

GGGCCGAGGAGGGCAUGUUAUCUCAUGUUAGUGCCCAGGAACCCAAAAGGUAAAAGGGGC

UGCAGAUCUUGCCCCUUGAGGAACAUAUGGCCACGUGGUGGGCUGGUAGGUUGGAGGUGA

GACUCUUCAGGAUCAUAGCUGUUGAAUGUUCAAGGUUCGUCAUGGUGAGAGGGUACAGGC

GUUUGAGUGUGGCUGCAAAGGGAGGCGUUUAGCUGAUUCGGUGGGUAGUUCAGCCCGGUC

CGAUUGUUCAAGCGUUCCUGGGAUGGAACUACAGAGUGUAUAUGCGGUGGUGAGCUCCCC

GUUCCUGAAAGCGUGCAAACCUUCCAGUGUUUUCACACAUCUGCUGGUGCUGGACUAGUC

GACGGGGAGAUCGCUGCUGUUGUAGUUUCCAGAGUUGCGCGACUGGUCCAAGCUCAGGUG

AUCAACUUGGCCACCUGGUCAUUGUUGCAGCAGCCUAGAAAAUCUCUCCUUGCUAGUUGA

GGCCCUUGGCCUUCUCGGGUUCAGGAGCACCUGACAAAGAUACUUCACCAGCGUGGCUGG

CGGGGGGGUCAUCCUCAGCCAGUGGCCAGCAGGGGCUGCAGAGUGUGUCAGGAAGGGGCA

UGUGUUCUGCGCCAGGGGUCCCGGGCUGGUACAGAAGGCCCAUGUCAGAUGCAGCCUGGG

CUCCUCAUCGGCCUGGCGGGCUCAUUCAUUGCCCAGGUCCCAGAUCCCUUUUGACUCGAC

AUAGUUUGUCUUCCAAUUGGGCUACCCCUGUCCCUUCUGCAGCUUCCUGGGACCUGCCUU

GCUCUGGGGCCCCUGUCUGGGGUGGGAAGCCACAGAUGGAGGGGUGCUGAAAGCAAGGGG

GACAUUCUUAGGACCUCAUCACAGGUGGUUUAGGGGAUGAGGGUGGGGUGUUCUCAACUG

AGUCCAGGCUGUUUGGGGCAUUCCCUCAUUCAUUCAUCCCAUCUUUAUUGAGAGCCUGCU

GUGUGUCAGGUCCCGUUCUAGGCCUUGGCUAUUCAGCCUUGGAUAAAACAAAGGCCUCAU

CCCUCAUCAAGAUUUGUCUAGCAGUGGAGACAGAACCAGAAUGAGUUAAGUAAACAAAAC

AAGAGAAUGUCAGCUAAUACAGCCGGGUGCAGUGGCUCAUGCCUAUAAUCCGAGCACUUU

GGGAGGCUGAGGUGGGAGGAUUGCCUGGGGCCAGGAGUUCAAGACCAGCCUGGCCAAACA

UAGCAAAAAUUGACAAGAAAUUAGCUGGGUGUGGUGGCACGUGCCUGUGGUCCCAGGUAC

UCAGGAGGCGAAGGAGGGAGAAUCGCUUGAGCCCAGGAGUUCAGGGCUGCAGUGAGCCGU

GAUUGCACCACUGUACUCCAGCCUGGGUGACAGAGCAAAACCCUGUUUCUGUUAAAAAAA

AAAAAGAAAGAAAGAAAAAAGAAAGAAAAUACCAGCGAAUCUAAAGUACUAUGCUGACAU

AACUAUCUUAAGCUCUUUACGCCUUCAUAUACCUAGUUAUGAUUUUUCUUUUUGACAUAA

UAAUUUUUGAUAGCCAUAUUAUUAUACUGACAAUUUUACAUAGGUUUUUUUCACUUAAAG

UGAGUGAUAUUUUAAGCUGAAUACUGUGGUUGAUUUCAGGUAAUAAAGACAGCCAUAGAA

UCUAAACAAAAGAGGAGCAAGAUGCUCAUAUAGAUGACACCAGCAAAUAGAUAUUGCUUC

AUCUUUGUGUAAGUAGAAUAAAACUGUAAGCAUAUUAAAGCCAGUGCUGUAUUUGACAUC

UCAUAAUCUUCCUCAGAGAAUGACUGUGGUCCGUUGAAGUGCAUUUUGACUAAAUAGGUA

ACCGCCUCACUGAAUUUGUUCCAAAUCUAAAUCACAAACAAACAUGUUUCUAUUUAAAAC

AACUAGAUGUGCUGGGCGCGGUGGCUCACGCCUGUAAUCCCCGCGCUUUGGAAGGCCGAG

GCGGGCAGAUAACUUGAGGUCAGGAGUUCGAGACCAGCCUGGCCGACAUGGUGAAACCCC

AUCUCUACUAAAAAUACAAAAAUUAGCCGGCUGUGGUGGCGUGUACCUGUAGUCCCAGCU

ACUCAGGAGGUUGAGGCAGGAGAAUUGCUUGAACCUGGAAGAUGGAGGUUGCAGUGUGCC

GAGAUCACGCCACUGCACUCCAGCCUGGGCAACAGAGCAAGACUCUGUCUCAAAAAAUAU

GUGUAAUAUAGUAUAAUAUAAAACAAAUAGAUGAAUAAUUAAGAUUUCCUACACUGUAAG

UAGUCAACUUCUGUUAGAAAAACCUUUGUGUGUAAAUUGUUGGAACUGAUUUGUCCUAAU

AAUUUUAGUUUUUUGGAUAUUUUCUACUAAAAUUAUCAUCCCUUCUAUUUUUUAGAUUUC

AAUCUGGUUAUCAUUAAAUUUCAUUGUGCAGUCAUUCAUUUUUUAAAAAUUCUAUUACUC

UGGUAAGUGGGGUAAUGGGAGAGAAAAUGAGGGGGUGACAUUAGCCAGAGAGGUCAGGGC

AGGCCAUGACAGUUAAGGGGACCCUUGUAGGCUGAGCAGCAGCUGGCUGUGUGGACCUCU

GGGGAGUGUCCCAGACAGGUGGAACAGGAGGUGCAAAGGCCUUCCUGAGCUGGCAGGGGU

CACAGAGAAGGCUGGCAUGGCCCAAGGAGGUAGGCAAGGGAGAGGAGGGCACAGGCCAGG

UCACGGGGGGCUGGUAAGGAGUUCCGGUUUUAUUAUCUGUUGGGAUAAUAAUGAUGAAGU

UUAUAAUAGUGAUGAAGUUUAUCCCAGGAAGCAAUGUGAUCAGACUUUUGUUUUAAAAGU

UCACUUUGGUGGCUGCAGGGAGGAAGGACCUGGGGGUAGGGGUGAGAAUACCAUGGGAAG

GCCCCUGCAGGAGCUGCCGAGGUAGGUAGUGAGCACCCUGUCACUGGGAGGUUUGCAAGC

UGAGGGUGCAUAAUGUUCAGGAAAUCUGCAGUGCCCAGGAAGCUGCCCUUGGCAGCCUCU

GCACCCUGCACUCCUUGCAGGCUCUGAUGUUUUGUGACCGCUCAUGCUAGACUGCAGAAA

CUGUCUCGUGUCCAGUGCUGGGACAUGGGAAGGGAGGCCCCGUGCAGGCCCCGUCCCACC

AUGUGGCUGGACACAGGGACCUCAAGUGGGACCGCAGCUCGUCCUGGCUGUUUGACUAGC

UGUGUGUCCCUGUGGGAAGUCACUUGCCCUCUAGGGCUCAGUUUCAGACGUGCUCACUAA

GGUCAGUGCUCAGCACGUACAGCACAUACUACUGGGUUUUUGGUUCUUCUUUCUUUUGUC

UACAGAGACCCAGACUCAUUCAUUCGACAAAUAUUUACUGAGCAUCUACUACACGCCAGG

CACUGUUCUGGGCCUCUAGGAGAUAGGAGUGAAUGAGGCCUGACAUUCUAGUAGGGGAGG

CUGAUAAGAAUAAAUACAUUGUGAUAAGUGACUGCCGCCAUGGGACAAUGAGAUGAUCCA

GCCGGGGUCAGGUGGGGCUCCCAGCUUCCCCUGGAGCAGCCUCCCUGGCGCUCACGGCCU

CCUCGCCCCCGCUGCUGUCAUUGCUUCUAGGUGGGAGGUCGUUCCCUCCCCAGAGACCUU

GCCCCGUCACUUUAAUUUCUCUGCGUCUGCAUGCUGGGGCCGCGGAGUGAAAGUUAAUUU

CACGCUUGACUUCCUGCCGCACAGACGAUUUCGGACGCGUUGGAGUCGCCCGUAGCCGCC

UCCGCCGCCCUCCCACCGCCACCUGCUUCGGGCGCUGCUGGUGUCCCCUGCUGCAGGAGG

GAGCGCUCAGACCACCGGGGCGGGGCCGGGGCAGCCGCAGAGGGGCAGGAGAUGGGGGCU

CCAUCGAGGGAUGGGCCCCACCCUGGGCAGGAUGCUCCAGGAAUUCCCGGGAGUCUAUGG

AGCCCGCGUGGGAUGUGGUGAAAGUGCGUGGGACCUGGGGAUGAGCGGCAGAUGGCGCUC

AGGCACAUUCUGCCUCUGCCUGCCCGGGGGUUGCCCUGGUUCACCGGCCCCCUCAGGCCU

GGAAGCCGGUUUCUUCCGUUGCUCGUUUUUCUCUUUUCGCAGCCUGAUUUCUUCCCUCCU

GCUGCCUCAUUUGGGGGUCGGCUGAUGGGAACUGUCAGACCCGGGGCUGUGCAGGCGGAA

AGGGGGAUCAGUCAGAACCCAGGUUACUCUUAGCAGGUCUCUCUGGUCUUGUUCAGAGAC

AUCAGAUGACUCCCCCAGGGUCACACAGACAAAUUCCUAGACCAGUCCUAACUGCACAUC

CAGCCAACUUCCUCUCCUGGGUCUCUACUCCAGGCCCUGCCCCUCCCUACCUGGGUGACC

UUAGACAAGUCACAUCACAUUGUUUGGCACUGUCACAGCUUUUGGUUGGCGCUCAGUGAU

CAGGAGGUUCAGACCAUCACCCCAACCCGCAGUCUCUUCCCAAGGUAUCCGGGCCCUGCA

UAUCUGCCUCCCUCUUCCUCUCCCCUGCAGCCGGGGUCAGGCUCUCCCUGAGCACCCCUU

CUGCCCUCUCCAGAUUCACUAUUGCUGCCUCCCUCCCUAGAACCCAGGGCUCUGCUCAUU

UUCUUCUUCCCACCGCAUUCCUCUGCUUCUCUCAGGCCUCAGCUCAAACCUCCCUUCCUC

CAGGAAGCCUCCCCUGAUUGCCCCCAAGCUUUCCUUUUCCAGUACCCCAUAAACCCCAGG

ACCAGUCCCAGCUAGGGUCCAACCGGACUCCCCUGGAAUACUCUCAUAAUUCCUGCCUCC

UUAUCCUGCCCUCCUAUGUCCUGCUUCUCCUCUCCAGUGCCCUGGCAGAUGGCAAGACUA

GGUUUCCUUAUAGGGUGUGUGUUUUUAAACCCUGACUUCUUUGCACCCCAUGAGUGCCUC

UACCAGCAAACUCAUCACAUGCUGCUUACCCCAUGCCUCCAGAGCCCUCCUACAGACUGU

GGUCCUUAUGCUUGCCGGACAGAAGCUCAGCAGAAAAGAUUUGUCUGCAGUAAGCUAUUU

GCAUCUUCAUUGCCUAAACACUGCUUGGAACAAAAUAAACACUUAAAAAAACGUUUGCCA

GAGGAAUGUUGAACACGUGGGCAUCAUAAACCAGGAGUCCCCCAGGUGGCUGCCUCCAGG

AGCGGGAAGGAAGGUUUGAGUGAGGAGGGCUGGUGUGAGGCAAUAGGGAGUAGUAGGGGC

UGAGGAACGGGGAGCUGGGCGACCUUUGAAAGGAGCAGCCAAGCCUCAGUUUCCCAGCCU

GUGGCCAUGAAGGGGUGUGGCCCGAUGGCCUGAUGUUUUCAAGAGAAGCUUUAGGUUUGG

CUUUUUGUGUAACAGCCUCUGGUUUUCAGUGCUGGCAAUGAAUUUAGGAUUUUAGAAAGC

ACCAGCCGGGUGCGGUGGCUCACACCUGUAAUCCCAGCACUUUGGGAGGCUGAGGCAGGC

AGAUCAUGAGGUCAGGAGUUUGAGAUCAGCCUGGCCAAUAUAGUGAAACCCCGUCUCUAC

UAAAAAUACGAAAAUUAGCUGGGCGUAGUGGCACGUGCCUAUAGCCCCAGUUACUUGGGA

GGCUGAGGCAGGACAAUCACUUGAACCCGGGAGGCAGAGGUUGCAGUGAGCCGAGAUUGC

GCCACUGCACUCCAGCCUAGGUGACAGAGCAAGAGACUCCAUCUCAAAAAAAUAAAAAAU

AAAAAAUAAAUAAAUAAAGCACCACAGCCCAACACAACACCCUCAGAGGCUGUCUCUGGC

CUGUGCACUGUGCAGUUUGCACCCUGCGGACUUGACAUGUGUUUGCAGACUUGACGUGUC

CUCUUGCGUGGUACGUGGAUGUCUCUGGGUGGCAGGCAGUGUCCUUGCUUUGGGAGGAAA

CCUGGAUCAGCCAGGGUGGAUUCACACCCUCAGUUGCUGCCCAUCGGUGGCAAACAGACG

GCUGCAUGCACAUCACCACCACCCCGAGGCCUCAUGCUCACCGAGUCGUUUUCCAGCUGC

AGUGACAGCACCGCGGUUUGUGGAAGAUCACACGCCGGACUAGCUUCUCAGCGCUGCCUU

AACACUCGGCCGUGAACCGGGCAGCUGAAAAUGGCAGGAACUUUCUUCUCUCACAGUACU

GGAGGCCAGCAGUUGGAAAUCAAGGUGCUGCAGGACCACAUUCUCUAGAAGGCUCUGGGG

GAGGCUGCUUCCUUGCCGGUUCCAGCUCCUGGCGGCUGCCGGCCUCCUUGGCGUUCCUUG

GCUGGGAGAUGCGUCACUCCCGUCUUUUGUAUUCGUGUGGUGUUCUUCCCGUGUCUGUUC

GCGUCAUCUCCCCUCCGUGUGUGUCUGUCUUUUUGUCCAAAUUUCCCCUUUUCAUAAGUC

AUUGGAUUAGGGCCCACCCCAGUAACCUCAUUCUAACUUGCUUCUCGCUCUAAAGACUCU

GUUUCCAAAUGAGGUCAUGUUUUGAGGUGCUGGGGCUUAGGACUUUACACAUAUCAGUUU

UUUUGUAGUUGGGGUUGGGGGUGACAUUCCCACCUGUACUAGGCAGUGUACCACCUGCAA

ACCCCAAAGUCAAUUUUGUAUGGUCUUCACCACGGUGUUUCAAAGUGGAUUCUAUCUCUC

UUCUUUUGUGAGAAGGUGGAACUAUUUAUUUUGAAGUAACUAGAUUGUACGUUGUGCAAC

AGAAGAGUUGCAAAUACGCUCCACAGAGUUCCCCCACACACGUCACCUGGCACCCCUCAU

GUGGACAGCUUAUAUAACAGCAGAGAAACACUUACCACAACUAGGGCCUGCCAUGGUGGC

CCACGCCUGUCAUCCCAGCACUUUGGGAGGCCAAGGUGGGAGGAUCACUUGAGGCUAGGA

GUUGGAGACGAGCUGGGCAACAAAGCAAGACCUCAUCUCUACCAAAAAAUAAAUUAGCCA

AGUAUGGUGGCAUGUACCUGUAGUUCCAGCUACUCAGGAGGCUGAGGCAGGAGGAUUGCU

UGAGCCCAGGAGUUCAAGGAUGCAGUGAGCCAUGAUCGAGCCACUGCACUCCCGCCUGGG

AGACAGAGUAAGACCCUGUCUCUAAAAACAAAAAUCAAAACAAAACAAAACAAAAUACUA

AGAAAGAAAUAGUGGCACAUGACCCUCUGCUAAACUACCAGCUGUCUUUGCAUUUUCACC

CGUCUCUCCGCCAACGUCUGUUUUCUGGUCCAGGAUCUGAUUCAGGUUCUCACGUGACAU

UUGUGCUCGUGUCUCUUUAGUCUCCAGCCCGUGACGGCUUCUCAGCCUUGCCUUGUUUUU

CACCACCCCAGCACUUUUGAAGAUCACUGGUCAUGGCUUUCUUUGACAGGUGUUUCCUCC

UGGUAAGACUGGGAUGGUGGGUUUUGGGGAAGACCAGAGAGAGGUAAGGGGGCUGCGUGA

UGCCCACAUGAUAGGGGGCUCUGCCCUAGGUCUCCGCUGUCGCCUUAGAGUUAUCCCUCC

UUUGCUCUGUUCUUUACUAGGGAGUCACCAAGUCCCACCCAUCUUCCAGGGCAGGGGAGU

UAAGCUCCGCCUCCUGGAGGGAGGAAUAUAGAUUUGUGGAGCCACCGCCAUGAUUCAUAC

AUACUUGCGGGGAGAUUCUUUGAGACUGUGCAAAUUUCCUGUUGCUCCUUAAAAUUUUGC

CAACCAAUUUAGCAUUCAUCCCCGGAUCUUGCCAGCGGUGGUUGGGUUAUUACUGCGUUC

UAAUGACGAUUUUCUGUUUCCCUCCUCUCUUGGACAUUUAUUCAUUGGAACUCUAGAAGA

AAGAUCUGUCCCUUCUCCUCCAUGUAUUGAUGUAUUGAAUCAUUUGUUUGUAAUCACAUG

GACUCGUGGGGAUUUGUUUUAUUCCUGGGACGCUAAUCUAGUCCCAGGGUUAUUUCUGUU

GUCACUCACAUUGUUCCAGCUCUGGCAGCCGGAAACCUUCAGGCUGGGUUCUGUGUCCUU

UUGACUUUUGUUUUCUUUUGUUUUUGGCAAUUCUUGACUUAAAGUCACUCUGGGCUCUUC

UUGCAUACUUCCUGGCCGAGCGCUGGAAUCAGCCAUGGCCCGGGCAGCGCGGUUUCUGGG

AUUGGAGGAUGGUCUUUGCUGAGAUAUGGGUGCUGCUGUGCUCAUGGCGCCUGGGCCUUC

ACUGCUUCUAGGCCUUCACAGCUGUCUUUUGCAUAAAAGACCAAACCCUUUGCCAUGUCG

AAACUCACAGGACCCUGCAGGACUGGCCCUGCCAGUCUUGCUCACAGCCCCUCCUCCGUC

CUCUCUGCCCCUCCCCAGCACUGUCCAGCCACAGGGCCUUCCUCUGUCUGUGCUUCCGAU

ACACAGAGUUUGUUCCUGUCUCCUGCGGUGCCCUCCAGCUGGAGUUCCCUUCCUUCUGGA

UCUUCCCAGAGCCGGGUCCUUCUGAUGACGUAGAUCCCAGCUCAAGGCCAUCCUCUCUGG

GAGAUCUUUCUGCACCACGGAAUCUAAAAUAGCCCCUCACACCCCGUCCCCCAGCACCCA

CUAGCCCACCAUUCCAUUUAAGUUCCCAAAUCCAGGAUUAUCUUGGUCAUCUUGCUUAUU

GUCAGUGUCACCUCUGUGAGAGCCCUCAACUGUGUCUUGCUCCCUGCUGUGUCCCCAGCA

CCUGGAACAGUGCCUGGCACAUAGUAGAUGCUCAGUAAACAGUUAUUGAAUAACAGAAGA

UGAGGCUGCGAUGCCAUAGAUUUCAGAUGAGGAAACUGAGGGCUUCUGAGGUGUGCUCAG

AGACACCCUGUUAUGAGAUGAAGGGUGCUGGUCCUAGCCUGUGGGACUGAGGACCCCCAU

GGCCCACCCUGGCCGGGGGGCGGCGGCCAGACUGAUGGAAGGUGGGGAGGCCCUGGCCAA

GUCACCACCAGAAUGACCCGGGGACUGAGGUGGAUUCCAGGCCCUUGAGCCCCAUGAGGC

UUCACUCGUCCGUCAGAGCCUGGGGUUCCCUCCUUUUCCUGUUUCCAAUGGGGCUGGGGU

CCCGGGGGCUGGACAUAGCGUGGCUCCCAGUCAGUAGCUUUGGAACUUUUGAGGGGGUUU

GGACACUGCUGAGAAUAUGAAAAAGUUGGGGAUCCCCUCCCCAGAAAAGCGGGUGCCACU

GAAUGCCGCGGAUUGGGGACAGACAGCAAGUUAAGGUGCACAGCAGGCUCCUGAGGUCGC

UGUGGCCCCCCAGGCCCUGGGAGGCCAGCUGCAGCACCCUGCUUUGUGUGUUGGAGACCG

UGGGGAGGGGCCUGGGAAGAGAAGAGGACGGUCCUGGGGUCUCGGUGCAGCAGUGGGUGG

GGGUGUCUUCCAGCCCUCAUGGCGUGGGCUCCAGAACCUCCCGCCUCUACUCUCCAGGGC

UCCCUCCCUUUGCCUGUCUCAGGGUCCCUGUCCCCUUUCUUCGGGUCUUGUCUCUUUCCC

UCUCCCCACUUCUCUCUCUUUCCCUCUCCUAGUUUCAUCUUCCUCUGAGUCUCUGUCCCC

UUCCUUUGGAUCUUGUCUCUCUGUGACUGCCUCUCUCUCUCUCUCUGAUCCUCUCUUUCU

CAUCUCUCUCACUCCAGUCUCACCUCUCAGUCUCAUCUCCUGUCCAUCUCCAGGUCUCUG

UCCUUUCUUGGAUCUCAUCUGUCUAUCUAUCUCCGUCUCUGUGUCUCCCCUUCUCCCAGU

CUCUGUCUCUUUCUCACCUCUGUCUCCCUGUCUGUCUCCCUCUCUCUGGGUCCCUGUUUC

CCUCUCUGGGUCUCUGUCUCCGUCUCUCUCUCUCUGGGUCUCUGUUUCCCUCUUUCUCUG

GGUCUCUGUCUCCCUCUCUCUGGGUCCCUGUUUCCCUCUCUGGGUCUCUGUCUCUCUCUC

UCUCUCUCUGGGUCUCUGUCUCCCUCUGUCUCUGGGUCUCUGUCUCCCUCUGUCUCCGGG

UCUCUGUCUCCCUCUCUCUCUGGGUCUCUGUCUCUCCCUCUGUCUCUGGGUCUCUGUCUC

UCUCUCUCUGGGUCUCUCUCUCCCUCUGUCUCAGGGUCUCCAUCUCUCUCUCUCUGGGUC

UCUGUCUCCCUCUGUCUCUGGGUCUCUGUCUCCCUCUGUCUCUGGGUCUCCGUCUCUCUC

UCUCUGGGUCUCUGUUUCCCUCUCUCUGGGUUUCUGUCUCCCUCUGUCUCUGGGUCUCUG

UCUCUGGGUCUCUGUCUCUCCCUCUGUCUCUGGGUCUCUGUUUCUCUCUCUGGGUCUCUG

UCUCCCUCUGUCUCAGGGUCUCCAUCUCUCUCUCUCUGGGUCUCUGUCUCCCUCUGUCUC

AGGGUCUCCAUCUCUCUCUCUCUGGGUCUCUGUCUCCCUCUGUCUCUGGGUCUCUCUCUC

CCUCUGUCUCUGGGUCUCCGUCUCUCUCUCUCUGGGUCUCUGUCUCCCUCUCUCUGGGUC

UCUGUCUUCCUCUGUCUCUGGGUCUCUGUCUCCCUCUGUCUCUGGGUCUCUGUCUCCCUC

UCUCUGGGUCUCCGUCUCUCUCUCUCUCUCUCUCUCUCUCUCUCUGUCUCUCUGGGUCUC

UGUCUCUCUCUCUCUCUCUGGUUCUCUGUUUCCCUGUCUCUCUGGGUCUCUGUCUCCCUC

UCUUUCUGGGUCUCUGUCUCCCUCUCUCUCUCUGAAACUCCCGUCUCCCAGGACGUGCCU

CCUUCUCUUGGAGCCUGCAGUGGUGUGUGUAACCUGCUUGGUUGAGACCCCAUGGGCCCU

GCCCUGAAGUCUGAGACCGCCCCCGCCCGGGGGUUUCCUGAAGUCCAUGCCUGGUGGCCC

CACCAGCUGCCCCACACUGCUUGUGUCCCUCCCCCCGCAGCAGGACUGGGUGUGCUGGAG

GUCCAUGCACAGCACCUGGUUGGAGCCAAUCCUGGGGCCACACAGGCCACACUCUGACAC

CCGGCCUGUGGGCGGCAGCAGGUCUCGGGGUCUCGGGCUCUGUGGCCUGUAUUCCUAGUU

GGAGGCUGUGGCUGUUUCUCCGUGGCCAUCUUUCCGUGGGCAGAUGUGGCUGCCGGGGUG

CAUGUGGGCGGGGGCGGGAAGCCACAGGCCCCUCGGCUCUGGGAACCCUCUUGCCUGCAC

ACUGGGCUCAACCUAAACGUUGGCGGGGGCUGCCUCGCGCGCGGGGGAGUAAGGAUGCAC

GUUGGCAGCUCACAGGUCUCUCUGGGGAUACAGCACGGGUGGGUCUCAUCUCCCUGAGAA

CCAAUAACGGGGCAAAUUUGGGGCUCACUCUCAGCAAACAGGAUGGCACGGGGUGCAGCG

CCUGAGCCCGGCCGGGCUGACCUGUCUGCCGCUUCCCUCCUGCACAGAAACCAGGCGAUG

CGGAAGAAGCUAAUCUUGUACUUCAAGAGGAGGAAUCACGCUCGGAAACAAUGGGUAAGU

CCACACCGUGGCCCCCAUCAGCUUUCCUUGAGAUCUGGGGAGAAUCGAGCGCACAGGGGG

CCACACCUGCCGGGGCCCUGUCAGUUCCGCAGCUGUGUCCUCCCUGAGUGUCCAGGCUGC

GGAGGAAAAGCCAAGGCCGGCCAGGAAAGGAGGAAAGACAACAGCUGCUUCCAGAAGGCC

CCAGGGUGGGUGGGGGUGGGGGGGCUCUAUGGCCCCUCUUCCAGCCUGGAAGGGAAGAAA

GCAAAAUGUUGGCCUGGAAAAUUAGUUGCUAAGCCCUGGUCACGGGACCUGUCCUGGGCU

UUUCAGGCAGAAAGAGAGGUUUUGGAGAGAGGUGGAGGAUCAGGGUGGGCUGUGUGCCGG

GCAGCAGGGGCCGCAGGCGCACCCUUACAAAUGAGCCUUUCCUGGUGCACUGGGCCCAGG

UCCAGGCUCAGGGAAACUGAGACAGUGGACAAGCAUUGAGCCCCCUUUCCCUGAGCCUGC

AUUUGUGGCCAGUGGCCUCAUCCAAACAGAAAAGGAUGCUGAUUCCUGCUUCUCACUGUA

GGCUGUGGAUCUCAGCCCACGGCGGGGAGGCUGGGUCUGCCAGAGGCAUGUGGUGGUGGU

UUCAGGCGGCCGUGGCCAUCACCCAUUCCCCGGAGGAGAUCAGGGUUGAGUAUUCUCCCA

GCUGUCAGGGGAAGAAGCGCGGACUCAGAUGGGCUUCAACAGGAAAGGAAUGGCUGUAGC

UGGAAAUGUCUGUUGGGUGUAGAUGGCAGGAAUAGUCUUUCAACUCAAGUCUUGCCUUCA

UUUCAUUUCAUUACAUUUCAUUUCAUUUUAUUUGUUUAUUUAUUUAUUAUUUAUUUUUGA

GAUGGAGUUUCGCUCUGUCACCCAGGUUGGAGUGCAGUGGCAGGGUCUCUACUCACUGCA

GCCUCCGCCUCUCGGGUUCAAGCAAUUCUCCUGCCUCAGCCUCCCAAGUAGCUGGGAUUA

CAGGCGCCCGCCACCAAGCCCGGCUAAUUUUUGUAUUUUUAGUAGAGAAGGUUUCACUCC

AUUGACCAGGCUGGUCUUGAACUCCCGACCUCAGGUGAUGCACCCGCCUCGGCCUCCCAA

AGCGCUGGGAUCACAGGUGUGAGCACUGCACCCAGCCCCUUCAUUCCUGUGCAGUUUUUU

CAACUCCUUAUGUUAGAACAUUGACAAACUGAACCUGAAUUCCCAUAGCAGGUGGGAGCC

GGAGAGAGCUGUGCUUUCUCGGAUGGUAUCACAGAUGCUCUGAUUAGGCCAGGAGAGGGC

CUGCGUCCUGUGAACCAGUGGCUGAUGCCGGGGAGAUGGAGUGAGGCUGCCUGGUUUUGG

CUCAUGGGACUUUCUCUACAGUUGGGGAUGAUGUCAUUGACUCCACCCAAGCCACGGGGC

UGCUGGGACAUGGUAGGGAAUGGGAUAAGGCUGGGGAGGCCCUGAAAAGGUCCCCACCUG

GGGAAGAGGCGGGCUGGAUGGGGACUGCUGCCUUGGGAUUCUUGGAUAAGAGACGCGGCA

AGGGCUGGGAACUAGGUUUGCUGGAGGCUGGAGGGGCUCCCCUCAUACCUGCAGCCCCCU

GCCCCACCUCCUCUCUCCCUCAGCUGUGUUGAGGCAGGGCCUGCGAGGCAGCUCCCUGUA

UCCUUUCUGGGCCUCCAGAGAGGCCCUCCCAGGCGCAGGCCUGGCUCAGUCAUGCCCAGC

GCCUGUAGGUCUGUGGUACACAGUAGGUGAUGACCAAGUCCUGCUUGAACUGAAAUGGGC

CGGGACAGGGUGUUAGCUGAUCCAGUGCCUUUGGGGCCUGGAUGCCAGUAGUCACGGAGC

ACCUACUGUGUGUGAGGCUCGCAGUUGCUGUGACACGGGCCCUGUCCUCAGACCUGCAGG

CUGCAGGGACAGGUCGUGCGUAAGGACGAGGAGGAUGCCCGCACACCCUGCCCUUCCGGU

GCCUGUUGUGUUGCAGGCGCUCUUCUGAGUGCUCUCCAUACAUUAGGGCAUUUAGUCCUG

CGAGGUGGGCGCUUGGUCAGCCACAUGUUUCAGAGGAGGAAAUCGAGGCACAGAGAGGUU

AAGUAACUUGCCCUAGGUCACACAGCUGGGAAGUGGUGGAGCUGAGAUUUGAACCCAGAC

CACCUGGUUCCUGAGCCCAUGCUCUGAACCCCCAAGGGGCAGUUCCGAGAGAGUCCAGGA

GGGUGUGUGGGGUAUGGUGGUGUGGUGAGCGGCCACAUGGUGGGGCUGAGCAGUGCCUGG

GGAGGGGGACCCUGGGCAGGCAUGGGGUCCUGGGCAGGUGUGUGGGCACGUGGGGCCAGG

UGAGGGGCUUAGCUGGGGCAGGAGGUCCUGGCUUUGGAAGGAGGGGCUGUGCUAUAUGGG

AGGAGGGUCUUCCCUUGGCCCUGCCUGCCUGCUCUGGCCCCGAACUUGGUUGGGUACUAG

AGGGAGGAGACACUUCCAGCCAGAUGCUGGUGCCUGGGAAGCUGCGGGCAGCCCUCGGGA

CCCAAGCCCUGCUCAUAGGUGAGGAGAGGUGGGCUGCCCAGCAGUGCCUUGCCCAGCUUC

CUCAGAUCCCCCACCUUGACUGGAUGGUGCCCACAGGCACUUGGGCUGGUGCGAGAGGCU

CUUGAAACGAGCGUCCAAUCUGUUUAUCAAGUUAAGAGAUACCCACACAGGGCUGCUGGG

UGCCCGGGAGUCACAGGCCAGGAAACAGACCGGUAAUCUGGGGAGAAGAGCUGCACAGAG

GGCUAGACUCUCGAGAUGGGCUGGGGACCUCAGUGCUGAGGUGGGAAUGACCAGAAAGGA

CUGGCUUUGUGCGGAUCUGGGAGUCAGGGUGUCAGGCAGAGGGCAUAGCAAGUGCAGAGG

CCCUGAGGCAGGAACCAGCUUGGGACAGAACCUGGCAGGCCAGCAGCAGGAGCCAGGUCG

CGUGGAAACAUCUGCCCUCAGGCUAGGAUGGAACAUUCAGGUUUUAUUCCGAAUGCAGUG

GGACACCGAAUUUAUUCUUAAUACAUGUAUUUGUAGUAAAGAAUCCAAAGUCCUUAUUCU

AAGAAAUUUGACAGAAGAGUACAAGGCACAUCUAUAUACCCACCACCUAGAAUCAGCCAC

GGUUACCAUUGCCUUGUAUUUGCUCUGUGUGUAUAACAUGUUUGUUGUUGCACUAUUGGA

AAAUAGGUGCAGAGGUGCCGACCUUCACCCCUAAUAAAUACUCCCCCUCUGUCUCCUGCG

AGGAAGGAAAUCCUCUCAUUUUCACCUAAAAUGACUGCCAAUUCAGCGCGAUCUUGCCGC

CUCCUUGACAGCCCCCCAUGGUUCCCCCCUUGGGGUGAAGGCUCCUCUCGGGAGGGCAGG

AGAGGAUCUGAUUUCCAUUUAAAAGGAUCGCUGCAGCUGCUGGAAGGAGCUCAGGCUGGA

GGUGGAGUUGGGGUGGGCUUGAGUUCUUUAAAGAUCGCCAGUGGCUCCUUGGGGGGUCUG

UGGAGGGGGUGGAGGGAGAAAUUGGCUUAUUUAAAGCUAGAGAAGAGGCAGGCAGAAACC

AAGGACUUCCAAGAAGGGUCCGGUGUGGCUUUGUGGCCCAUCUUAGGAGGGAUGGAAACA

AAUUCAACACCCCCUGAAGCUGCGGUGGCGAUUGGGAUGGUCUCUUUGUUGUGGUAUAAG

AAGCCGAAGUCCCUGACUUCAUUGAUUUCAGUAAGGCCUGGGGAGGGGGGCUUGGCCGAG

AACUCUGAUGUGCCUGCCUCACCCCCACUGGGGAGUCAGACCCCCCUCAGACCCUGGGAC

CCCCACACCUUAGCAGGUUCCUGGGGACCCCCAUACCUCUUCCCAGAGAGCAGGGCCUCG

UCCCUGUCAUGGGAUCCUCAGCACCAGCACAGCCCCCCAAGCCUGCCACAUCUCCUGGGA

AGCUGAGCUGGACCCUCUGGAGGCCCAGUUGGCUGAGAGACUGGCUCAGCCUGUGGUCCC

AGAGGCAUGGCUGGCCUGCUUCUUCUCCCUAGUGGAUUUGCGCUCCUAGCACCCCGCAGA

CUAGCUGGCGGGGGUGGGGGGCGGGAGGGGGACGGCUCCAGUCUUUCCCUGUGUGUGUGU

GUGCGUGUGUGUGUGUGUGUGUGUGUGUGUGUGUGUGUGUGUGUGUGUGUGUGUGUGUAA

GACAGAGAGAGGGAGGGAGGGAAGGGAGGAAGGGAUCUGUUUCCUCCCCUGGCCAGUGGG

UCUGCCCUUGUAGAAAAAUGCCAGUGAGCAUCUUCCCUGCUACCCAGGCCAGGCCGGAGC

UGCCCGCCCUGCGCUGUCCGAGGCAGACCGAAUGCACACCCAGCACAGUGGACAUAGGCA

CAGGCUACAGCAGGUGGCACUGUGGUUAGACCCAGAGGGGGACUUCCUUGUUCUGGGCAG

AAGGCAGAGCCCAGUGCUUGGGGCCAUGGAGGUGUCUGGAUGUCCCCUCAGGGACACUAC

UGCUGGAGGCCUUUGCCACCCGUGCACUCCACGAGGGACCCAGCUAGAGCCGGGCCGUCU

UGCCAGCGCCCUGCCGGCAAUGUGGACAGGCUGAGGCCCCGCUGGCCAGUGGCCUGUUGG

CUUCCGCCCGCUGGUCUGCACCAGUCACCACGUGACUUUCCUCUGCUGGCUUGAGGUGAG

CUUUUCUCACCCAUGAGCCUGGGGCCUCUUGGACAGGGGCCGAGGUGGCAUGGGCUGGUU

GUUGCCGUCCCUGCAGUCCCCAUCCCUCACUGGGGCUGUCAGCAGCCACUGCCCCGAGCA

CGUGUGGCCGUUGUCUGUCACCAGAACUGCCUCUUGAUCUCCAGCGCGUUCCUGGCACAG

CCUGCAUGCAUGGGGUUAGGUUCUGGGCCACCCAGCACCAGCCUGGCCACCUCGUGGGGA

CCGUCAUGGUCACGGGGCUGUCGAGGAUGGAGGGCACAGCUUGCCCUUGGUCAGCAGCUG

AAAUAUGGGGUGGGGUGGGGCUGAGGUGUCUGGGCUCCAGGACCAGAGAGGGGCUUUCUG

ACUGUACCGAAGCACCAAGUGGGUGUUUGUGGAGGCUCCCAGAUCACACCGUCUGUCCAC

CCCCAGCUGUUAGAGGUGGAGUAACUGGGGUUGUGUGGGUGGCUCGCCCUGCGUCCCCUU

CCCCGCCCCCACCCCCCGCCCCCCGCCGCCACUUGCUCAAGUCACCUUGGAGAUGUGGAA

GCCAGGCAGCUAUGCUCUGGGCUGAUUGGGGAUCUCUUCUCAAAGGACAUGCAGCAGGCA

GCACUGUGGUUAGAUGUGGAGAGGGACUUCCUUGUCUUGGAGGGAAGGCAGAGCCCCCUC

UCUGGGAGCUGCUGGGGUCCUCACAGCUGGUCCUUGGGGUGAGGCUGGGGAAUGAGGAAC

UGCUUGGAAUGUGGUUUUCUCUGGCGGCUGCCCCUCCCCAGGGCUGACGGGCCCGCAUGU

UGUACAGCACAGCUUCCUGUGGUGUGGCAGUGGGCUCCAUCCGGGCUGUCUGGUGCGGCC

GGAGCCCCAGCCGCAUGUGGCUGCUGGACACUGGAAGCAUGGCUGGGGAGAACUAGAGCC

CGACUCUUAAAUUUUACUGGAUUUGUGAUUUCAGUUGAGCCACACAUGGUGGGUGGCUUC

CAUUUGGGACCAGGCCUCUGCAGAGGAGGCCAGGUCUGGGUGGCCGGUUCACCAGGAAGG

GAGGUGACCGUGUCCUCAGGGGAAGUCUGUGGGUUUUCUUCAGGGGCCCCCGUCUGCUGG

AUAGCCAUGCAACUAUGUGCCUGUCUUUCUGUCGUCUGCCUGUGUGAUGUGUGUGUGUCU

GUUGUGUGUCUACAUGUGUGACUCUGUGUAUGUGUGUCCAUCAUGUCUGCCUGCGAGACC

CUGUGUGUGCAUCUGUUUGUCUGCCUGCGACUGUGUGUGUGUCUGUCAUAUGUCUGCCUG

GAUGACCGUGUGUGUGUGUCUGCCUGCCUGUGUGACCGCCUGUGUGUCUGCCUGCCUGCC

UGUGUGAUGGACUGUGUGUGUGUCUGUCAUAUGUCUGCCUGCAUGACCAUGUGUGUGUGU

CUGCCUGCCUGUGUGACUGACUGUGUGUCUGUCUGCCUGUGUGACCAUCUGUGUGUCUGG

CUGGCUGGCUGUGUGACUUAUGUGCCUGUGUAUAUAUGUGUGUGUGUGUGUCUCUCUGUG

UAUCUGCCUAUAUGACUGUCUGUCUGCCUGUGUGACUGUGUGUCUGUCUGUCUGUCUGCC

UGUGUAUCUGUCUGUGUGUCUGCCAUGUGACUGUGUGACUGUGUGUCUACUGUGUGUGUA

CCGUGUGGGUGACUGCAUGUGUGUAUGUGACUGUGUGUGUCUGUGACUGUAUGUGUGUGU

AUCUGUGUGUCUGCCUGUGUGACUUGUGUGUGCCUGUAUGUAUGUGUGCCUGUGUAUAUC

UGUCUGACUGUAUGUAUGUGUGCCUGUGCAUAUAUCUGUGUGUGUGUGUCUCUCUGUGUA

UCUUGUGUGUGCCUGUAUGUAUGUGUGCCUGUGUAUAUCUGUCUGACUGUAUGUAUGUGU

GCCUGUGUAUAUAUCUGUGUGUGUGUGUCUCUCUGUGUAUCUGCCUAUAUGACUGUCUGU

CUGCCUGUGUGACUGUGUGUCUGUCUGUCUGUCUGCCUGUGUAUCUGUCUGUGUGUCUGC

CAUGUGACUGUCUGUGUGACUGUGUGUCUACUGUGUGUGUACCGUGUGGGUGACUGCAUG

UGUGUAUGUGACUGUGUGUGUCUGUGACUGUAUGUGUGUGUCUGUGUGUCUGCCUGUGUG

ACUUGUGUGUGCCUGUAUGUACGUGUGCCUGUGUAUAUCUGUCUGACUGUAUGUGUGUGU

GCCUGUGUGUGCAUGUCUGUGUGACUUUGUGUAUCUGUGUGUGUCUGUGACUGCAUGUGU

GUCUGCCUGUGUGACUUGUGUGUGCCUGUGUAUAUCUGUCUGACUGUAUGUGUGUGCCUG

UAUGUGCAUGUCUGUGACUGUGUCUGAGUAUAUGUGUGUUUGUGUGACUGCAUUUGUGUG

UGCCUGUGUGACUUGUAUGUGCCUGUGUGCUUGUGUGCCUGUGUGUAUCUGACUGUAUGU

GUGUGCCUGUAUGUAUGUGUCUGUGUGGCUGUAUCUGACUGUAUAUGUGUGUGCCUGUGU

GACUUUGUGUGUGUGCGCACGCACCUGUGUGUCUGCCUGCAUGUGAGUGGGCAGGGGGGA

GGCGGCGGGUGCCAGGUGCAGGAGCAUCUUCCGCAGUGCCCCCUGCGCUCUCCCAGGAGC

AGAAGUUCUGCCAGCGCUAUGACCAGCUCAUGGAGGCCUGGGAGAAGAAGGUGGAGCGCA

UCGAGAACAACCCCCGGCGGCGGGCCAAGGAGAGCAAGGUGCGCGAGUACUACGAGAAGC

AGUUCCCUGAGAUCCGCAAGCAGCGCGAGCUGCAGGAGCGCAUGCAGAGGUGAGCGGGGC

CUGAGCCCAGGGCCCCCGACGUCAGGGCCCGGGGUCAGCUCCAGCAUCCUCAGUAGAGAU

GGCCUGGCCAGGAAGGAACAGAACAGCACAGCAGCUUCUCAGGCCAGUGGCCAGGGCCCU

ACCAGACCCUGGCCUGUGUCACGUGGAGCCUCUUUGGCCUAAUACUUACUUGGAACUUAA

AUACAUUUUGGGGACAAGCACUUCUUCCCAUGUGCCAUAGCGUGCACCUGCCAGCUUCAC

UCAUUUCUGGGGCCUGCACGACCCCUGAAGGCAGCUGAGUUUGAGAUCCCUGGUGUGGGA

CAAAGGGGAGAGACGUGGCUAAGAGACCACGCUGUCAGCCUCCCCCAGUGGCUGUGAGGA

CUGGGUGGGCCCCAUCUGAGAGGUGCCUGGUGACAGGCAGCUGUGGUUCUAUGGCCAUUU

UUCUUUGGAUGCACAGAUAAGGCGAUUCAUUGGUAAUGAUAACAACACCUUUCACCCUUA

ACUGAAGUCACAAGCCAUGUCUCAUUAAGUUCCCCACAACAGCUCUAGGCAGCCAGGUGC

CACUUUUGAAUCCUCAUAUGACAGGGGAGGAAACUGAGGCACAGAGAGAAACACUUCGGC

CCAGGGCCAAAGAGGCAGAGCUGGGCUUUGAACCCAGAUCUUUGUAGCUGGGCUUUUCAG

GGUGUUUUAGGAGAGAGGAAGGGACUGAGCUGCCCAUGUCACAGGGUUUGGCCUUGCUGG

GUUGGGGUGGGGGCUCCAUGCCUCCCCAGGACCCCGGGUGUGGGGCCGGCCCCUCUGCUG

GCCGCUGCCCACACUUCUCCUCUGGGGAGGGCGUGGGCUCAGGGCUGCGCCACAGGGGUC

UGCUAGUGUCCCCGACCACCAGAAGACCGGUCCUGAGUGUGUCCCCUUGGUCCCCAGCAG

GGUGGGCCAGCGGGGCAGUGGGCUGUCCAUGUCGGCCGCCCGCAGCGAGCACGAGGUGUC

AGAGAUCAUCGAUGGCCUCUCAGAGCAGGAGGUGAGUCCAGGCCCUGACUCUGGCCUCAG

CUCCUUUUCCCUGGCAUCCCCCGUGAGGGUGCAGAGGCCCUAGCCAGAAACCACCUCCCG

GGUGGUUUACGUCGAGGGGCUUUGCCACCGAAGCGCGGGUGGAAUCAAGGUGGGUAUUGA

GGUACCCGUCCCAGGCCUGCAGGGGCGAGCAGAGAGAGGGGUGUUAAUGUGUCUGAGAGG

GAGGGUAGCAGGACCCCCUGCAGGGAGGGCCCUGGCGCUCCCCCAUCCAGAUGCCACAAC

AGGGAGGGGGUUGGGGGACGAGGACCCUGCCUCUCACUCUUCCCAUCUCCUGCCUCCUUU

UGGAACUUCCCAUUGGCUGAGUCCAGCCAGAAGCCCAAGGGGCGGGAGUGCCUGUGGGUU

CCAGGGUCAGCCUUCCAGGGCCCAGAGCCUGGCAGGGUGGGGGGAGCAUGGCGCAGGCCG

AAGAGUUCCCAGCACGCCAGCUGUCCACGCAGCCACGACCUUCUCGCAGGCACCUCAGCC

CUCCUGGCUGGAGCAGGGCCGGUUUCCCCUCAGGCCUCUUUGGGGUGCCAGGACACCCUG

GGGUCAGCCUAGGUGACCCUGGCUGCCGUCACUGGGGCCCACAGAUGGCCAGGAAGCAGA

GAGGCAGCCUUUUCUCCUGGGGAGGAAGUUGGUGUCAGCCUGUUCCCUCCCGGGUCCUGU

GGGUCAGGUACCCAUGAUGAGACUACCCCCUGCUCCUAACACCACAGCAGACACGAGGCA

GCCACGGCACGUUUCUUCCUUCUGGGGACUUUUGUCCAGGGAGAGCUUUUGUCCAGAUGG

GAGAUUAUAGCCCUUUAGCUCAGCACAGCAGAGCCACGCAUGGCUAUUGAAAUCAAAAUU

AAUUAAAAUGGGAUAAAAUUAGCAAUUCAGUUCCUCGGUUGCAUCUGACACAUCUCAAGA

GCUCGACAGCCGCUUGUGGUUGGCAGCUCCCCUCCUGGGCGGAGCAGACAGCGAACACCU

CCCUGGCUGCAGAGUGCUCUGUUGGACAACAGAACGCGCUGCCCUGCGAUGCUGAUGCUC

AGAUUAGACAGAUGGAAGGGGCCCCAGGGAGGGGGCGGCUGGUUUAAGUAGCAGUUUCUC

ACCAGAGACUCUGAUUCAGCUGGUCUGGGGCAGAGCCCAGGAAUCCGCAUUUUUACCCAG

CCACCCACUUGAUUCUGACGCAGGCAAUCCAAAGAUGAAUCUGGCCAUUAAAACAUAAUU

UUUCAUUUUAAAACUUCUCGUGGUACGUUUUUGUUUACAACAACAUCCAGUUGACAGAAA

ACAGAGCUCAGAAAGCCAUCGCAUUGCACAGCCACAGGGCCCGGCCCUCGAAGGCUGGGC

GCCGGAGCUGGGCCGUGUCAGCCCCUGUCUCACCGAUGCUGUGCACUUAUGGACAUCCCG

CAGAGAGGCUGGCUGUGGGUUUAGUCUGCACUGGAAGGAGGAGGCCGGUGGCCCUCUCCU

CUGCAUUCCUCACCUGAUGGCCAAGGGUGUGGCACCUGGGCUGAUUGUUGCAUGUACACA

CACACACACACACACACACACACACACACACACACACACUGGCCACAUGGGAAUGCAGUC

AUCAGAGUCCUCAGGGACAUCAAGGAUGCUGUGUCCGGGGUGAAGGGUGGAAGUGGAAUU

UGGGACAGAAACUUCCCCAGCCAGCCUGGGGUGACCUGAGGUUCCCCCGGGAGUGUGCAA

GGGAGGGGGCUGGGCCGGAGGACAGGCACUUCCCAUCCUUCUGGGAGCUUCCUGUGGUCA

GAGCACAUGGCCGGCAUGGGAAGGACAGGAAGGCAGCUGUGGGAGGGCAGAGCAGAGGUC

CUGCCUGUGGCCGUGCCGGGGGUCCUGCCAUUAUUACAUCCCUGGGAUACCCGAGGAAGG

UCGCCGUUCCGUAUCAGGGGUGUAGGGGACAGCUCCAAGGGUCUGGGAGACAGAUGCCAG

CCUCUUGGGGGGAUUCUCUCCCACCCCGGGGUCAGAGCUGUGCCCCGCACCCGACCUGAC

CCCUGGCUGGGAGUCCUGCACAUUGAGUGAAGAGGUUGGAUGUGGGCGGCCGGGCAGCUG

GAUCGUUUGAACUGGCUGACCUUGGCUACGCGGAGUCCUCGCCUACCUCCGGAGAUGGGU

AAUUAAUCGCCAGUAAUUGGCUGGCUGCGAGAUUUGGGGAAGUGAGCCCAGCUGAGUGGC

GGCUCAGAGCCUCUUAAUAGUCAUCCCAGUGCAUUAGGAGCGGCCGCCUGACUCCUGCCG

CAUGCAGGGCUGGGCAGGCCAGAUCCCAGGUGGGCCCUCUGAAGGAGCGGGGUGCCGGGA

GGGGGCGUGUAGUGUGGGGGCAGGAAAGACCUCUCCACCAGCGAGGCGCUGACCCUCUCU

GCGCCCCUGGGCUGCCUGCAUGCUGUGGGGCCAGCUGGACCCCAGGGCCCACUCUUGCCU

GCGGAACCUGGAAGGCCAGCCUGUCUCCCGGGCUCCCUGGGCCCAGCUGGGCUGAGCAUC

CUUCCUGCAGUGCUUUAUGGCGCUUGCAUUUCCAAAGGGAAGAUUCAUGGCUCCCCUCCC

ACUGCAGAGAAGGCGGCUGCUGCUCUCUGCAUUGAAUCUACAGUCCUGCCCACCAUAAAC

CUGUCGGGGAGGCCUUUACAUUCUCCCUUCCCCCAGCAUGAGCUAUUAUUAUUUUUAAAC

UCAUCAUUCUCCUUCUCCUUGGUUUCGCGUUUUCCCUGCUCUGGCGCAGACCAUCCGUCC

CCUCUGUGUCUUCCCUGGGUCCCCUGCGCCGGCCGGAGCACCUCGUCCCUGGGCCUCUGA

CCCUGCCCUCCGUUUCCCUGCAGAACCUGGAGAAGCAGAUGCGCCAGCUGGCCGUGAUCC

CGCCCAUGCUGUACGACGCUGACCAGCAGCGCAUCAAGUUCAUCAACAUGAACGGGCUUA

UGGCCGACCCCAUGAAGGUGUACAAAGACCGCCAGGUCAUGAACAUGUGGAGUGAGCAGG

AGAAGGAGACCUUCCGGGAGAAGUGAGUCCUCCAUCACCUGGCCUGGCCUCCCGGCCCCC

CACCCCCAUCCUGUUGAGGCCUGCGCUGUCUCCCGGCAGCCACCACAAAUGAGCAUGCCG

GGGGGUGCUUAAAACCGCAGGGAUUUACUCUCUCCUGGUUGUGGAGGCCGGAAGUCCAAA

UCCUGGUGUUUCGUGGGGUUGGUUCUUUCUGGGGGCCGUAAGGGAGAGCCUGUCCCUGAC

CUCUUCCCAGCUGGUGCUGCCAGCAUCCUUGGCCUUCCGGGGCUUCUAGAUGCAUCCUUC

CAUGCUCUGCCUUCGACCCCACAUGACCUUUGUCUCUGUGCUUCUCCUCUUCUUAUAAGG

ACACCCGUCACACUGAAUUUAGGGUCUAGCCUAACCCAAUACAACCUCACUUCAACUAAU

UACAUCUGGAAAGACCCUGUUUCCAAAUAAGGCCGCAUUCUGAGGUUCUGGGUGGCCAUG

AAUUUUAAGAGGACACACUUCAAUAUGGUACAAAGUGUAAGGGCAGCUGGAUGUAAGACG

GGGCACUGCGAGUCGGAAGAACAAGGCAGGAGGGACUUAGAGGCUGGGCCGCAGCCUUGG

GCAGGGCGGCAGCUGGCCAGUGGCUUUUGGUGGUUUCUAUUUUGUUUUGUCUUUGACAAU

AGCUGCUGGCUGAGUGGGUUCGUGAUCCCAUCACUGUGCCUGUGUCUUCUCAGCUUCAUG

CAUUUUCCCCCUCCUGUCCCUUAUGACAGCUCAGCCAGGCCCACCCCAGUACACAGCUGA

UGACACGGAGGCUCAGAGAGGUUCAGACAGUGCACAAAGGCCACACAGCAUGCACACAAU

GGAGGCAGGGUUCAGACUCCUCCAGCUGGAAUGGAGAGGCUGUAUAGGGAAUGGUUUGUG

AAUGGGGCCAGGGAGGGGGGCUGUGCAGAGCAGAGACGGGAGUCGAGUUCUCUCAAAAGC

UGUGUCUGAGGGGGCCAAACCCCACAGUGAGAAUCUGCGAUGUGACAUCCAGGCAGAAGG

AGACCUCCAUCCCUGGCCCGGGGUCCCCAGAGGAGAUCGUGGGCCCUGCUGAGCCACCCC

ACGCCUUAGGGACAGUGUGCUAUAAUUCCGGGGAAUCUAAUCUUUUGGCUUCCCCAGGCC

ACACUGGAAAAAGAAAUGUCUUGGGCCACACAGAAAAUACACUAACAUUAAUGAUACAUG

AUGAGCUAAAAAAAAAAUCACAAAAAAAAUCUCAUAAGGUUUUUUUUUCCUUUUUUUCUU

UCUUCUUUUCAAGACAGAGUUUCGUUCUGUUGCCCAGGCUGGAGUACAAUGGCACAAUCU

CUGCUCACUGCAAGCUCUGCCUCCCGGGUUCACGCCAUUCUCCUGCCUCAGCCUCCCGAG

UAGCUGGGACUACAGGCGGCCACCACCAUGCCUGGCUAAUUUUUUGUAUUUUUAGUAGAG

AUGGGGUUUCACCAUGUUAGCCAGGAUGGUCUCGAUCUCCUGACCUCGUGAUCCGCCCGU

CUCGGCCUCCCAAAGUGCUGAGAUUAUAGGCGUGAGCCACCACGCCUGGCCAAAAAUCUC

AUAAGGGUUAAAGAAAGUUUACGAAUUUGUAUUGGGCCGCAUUUAAAGCCGUCCUGGGCU

GCAUGUGGCCUGUGGGUUGGACAAGCUUGCUCUAAUCACUUGCACGUAGAGGGCUGGGUU

CCCUGCCCAGGACAUAGAGCACCUCCUCAGGACCUCAGAGCUCUGGGAGGCAGGAGAGUG

GGAAACUGAGGUGUGGACGGAGGCCUCUGUGCUUGGCCAGGAGGGGACGCAGGGAAGAGA

CUUUGUCACUGCCCGACGUUCACCCGGCAGGCUACUGAGCUGCCUUGGGGAAGACCCUGC

CUCCGGACAAGAGCUUCCUCCAAAGCAAUGACACUCCUUCCCCAGUGCCCUGGGCUUUGG

UCCAGGGUUGUGGCCCCAAAGAGGUGCCAGGCAGGACCUAAGGGAUGGGGUGACUCUGGG

UCCCCGGCAGGGGUGAGUGGACCCGCAAGUGCAGAUACCGAACUCAGAAAGGACACAGUG

GCUAUCCAGGAGGGUCUUUGGUGGAAAACAUUUUUUAAGAGUAGCUUUUGAUUAUGGAAA

UGUUGAAACGUCCAUAAGAAUAGAGGGGUGGGGUAAUGAGCCCUGGAGUGCCCACAGCAC

CGAGUGAGUGGUCCUUCACAGGAAACAGCGCUUGGUUCCUGAGCAGGAAUACUCGGUUAC

UUAAUGGCUCGUGCCUGCAGCCUCACCAUAGCAGGCACGAUCUUUCUACGUCAAAUCAAA

GUCUCCGUCGAAUGCUUCUUACAGUUUUGAACGAGGGAGGUGAUGGUGGGACGGUCACGG

GUGUUAAGAAUUACUGAAUUACUGUUGCGUUUGAGAUUCACGUUCCCCCUGGUAGGCAUG

AUGUUUUAGUGUAUGUAUUUUUUCUGAAAUACAAAAAAAAAGGUGUUCUGGGAGACUUUC

UGCGGCUUCAGCCUUUCGGAAGAUCUCAUGUGGCUCUGCAGGUCUGAUAAUGUCACCCUC

UGUUAAUUCAGUAACCGAAGGGUGGGGGACCCAGGCUGGGCUGCUAUCUGCCGGCCCGUG

GGGCUGGCGUAACUUCCAUCCUGUGUGAGCCGCAACUUGGCCACUUGCAUGCCUCAAGGA

CUGUGGGCUUGGUUUGAGGUCUCAUGUGCAGAGGAAUUGAUAGCACUCGGGGCUUUGAAA

AUCACCUACUGACGGCAUGGGGCUCCACCACGUGCUGGCCAUGUGACCAUUCCCCCUCUG

AACCUCAAUUUCCUCGUCUGUAAAAUGGGUAUAGCGACAGGGCCUCCCUUGUGAGGUAUG

CAGGGCAUAGGAAACUUCUAAUAAACGUCUUCCAGGGGUGCAAGUGAGGUAUUGAGGGAG

GGGAACAGGGGACCUAGGAGAUUCCCAAGUUUGAUUCCUGGUCUCUUGGGAGCCCCCACU

GUGCCUGGGGAAACCCCGUGGGAGGGGUUUGCAGGGAAGGAAAUGUCACUGGGACAAGGA

GAGGGAGCGGAGAGGAAAGGACUCUCCCUACCUGGAAGAGAAGGGCCUUAGGAUGGCUCC

GGCCUUGCCAGCCGCCCGAGGUGGGAUCCCAGGCCAGGGAAGCCUGCCAUUCCUCACUGC

GCCGCUUCCACCGGGAAGAGAGCAGAGACUGUUCAGGAUCCUCGGAUGUCUGGCUGGGAA

GCAGGCGCGGGGCCUGCUGGCCUCCUGCCCAGCCAUCUGGAGGGCCAGGCAGGUCGGGCA

CUGGUGCUCACUCAGCCUCCCUCCGUGGGAGCCCACAGUCCAGCCUCACUAGCAGCCAGC

CGCUGCUCUCCCUGCGAAGGCUGUGGGGCUGUGGCACGUCGCCGGGACGCCUGGGCCUGG

GGCCAGGGACUCCACCCAGCACCCCUGGGGUGGAGCAGGCCUUGACCCACAUCCCCGCCC

CCACCAUGCUCCCGCUCCUUUGGCCCUCACAUUUCAGCCUGGGCCCAGUGGUUUCCCAGU

AAUUCUCCUGGCUACGCAGGAAGCCAGUUGGGACAGUGCCAGCGACCCGCCACCGCCCUC

CGACUUAAGUCCAUGCUUGCCGCCUCCUUGGCUGGCCAGCCCCCUCCUGCUGCCCCACGG

GCACUCAGAGCCUCUGCUCCCAGCUCUUCUGGGGAGGCCCAGCAGCCUGGUGAGCUAUGA

CCCCACUGGUGGGGCCCUGCCAUGUUCCCAAGCAGACCCUGUGUGGGCUGGGUGAGGCCC

UGCUUCCCAGAUCCAGCUGGAGAGAGAAACAAAAGUGGAUUUUAAAGGGGGGGGAACCCC

ACCAAAGAGCUGCAUGUCGUGUCCUCAUUUUUCCUGGAAGCCGCCUCCAGCAGGACAAAC

AAAUAUAUUUUCAAAGGCGCUAAAGCCAGUGACUCACUCCAAGGAACGCCCUCUCUUACC

CCUGGGUCCCCACCCCUCCCGUCUGCCGCAGCAGCCCUUCCCACACCCCCCUGGGCUUAA

UUGCUCCAAGUGGGGCGGUGCCCGCCAGGCCCGGCCAGUGGGGAUGGCAGGCGCCUGGGA

GCCGAUCGGCUGCCCCGCAGGAAAGCCCCCCUCGGCCAGGUCUCACGCCCACCCUUCUCG

UCCCGCAGGUUCAUGCAGCAUCCCAAGAACUUUGGCCUGAUCGCAUCAUUCCUGGAGAGG

AAGGUGAGUCGCUGCCCGCCCCAUACCCCUUCGGUCUCCACCUCCGUGGGCAACUGCGUG

GACUCAGGGUGGAGGCCCUCCCUCCUUGCCCACAAGGCCCUGGCCUGCCUGGCCCCUUGC

UCCAGCUGCCAUGCUCCCCUCCUCCCUCCCUCCCUUCCUUCCAUCUGGGAGAUAGAUCAG

UGCAGGCCCCGCCCUGGCCUCCUGGGCCCAUGGAGGAGGAGCCUCAGGUUGCUGAGCACC

UGGGAAUCCCGAGAACCCCAGGGAGAGCUGCACGGCCAGGCUUCCUGGAGCCCCGUGUGC

CCGACGGGUCUCCUGCCAGCUCCUCAAAGGCCACGUGGAGCGUCCUUGCAAACUGGGGGU

GGAUUCUGGAAGCACUACGUGCCUGGCAUCCCAGUGAAGUUUUCGUAGGCAGGAGUGGCG

UGCCUCAAAGGGACUGGAGGGACCAGCUUUUCUAGGUCCCUCUGAGGGUCACUGACUGCU

UUCUGACACCUCUAAUGCCAAACCAGACGUGUGGCCUGCAAGUCCCCAUCUUCUUUGGAC

AAACCUUUGUGUGGACCUGAGGCCUAAAGCCCACUUUGUGUCAAGCCCAGGCCCGGAGCA

GCUCCAGGUGGCCGGUAGACACAAAGUGUCCCCAGAGAAUGGUAAAGCCGUUGUUUUGCA

CCCACAAGGCUGGUUGGACUGUGCCUGGAGAGGCUGCAGGUGGGAGAAGGUGGCUGGAUG

AGGCUGCAUGUUGGACGUGGCCCCUGGGCCUUCCCCCAGCAUCUUGGGGUCAGAGGAGAA

AGGCUGCCGUCCGUGUGUCUUAAGCAGCAUUCCCCUGAAUCACGUUUCUCAGAGAUGGGG

ACCUGCCCACCCCAGCUUGGUGGACACCCUGCUCGGGCUUCCUGGUUGUAGGAGGGAGGC

CAGGAGGAUGAGGCACUUAUUUUAAAGGACAGCUGUUCCCAGCGCCUGCCCCUCAUGAGC

UGAUAACCGUAGAAGGAGAGAGACCGAGCAGAGUGGGGAAAAAUCUCUCCAUGCCUGAAA

AACCAGUGUGGAGAAACAAGCCCUAUAAAUAGCCCAUCUUCUGAGUGACGACGUCUCCCU

CAGGGAGUGGACGGCACGGCGGACUCUAGGUUAGGGCUCCCCAGGAUGGAAGCAUUUCAG

AAACUGGCCAUCGAGCAGUCCUCCUUCAGGAUGAGGGGAUUAGAAGUCUUUGUGCUGGGG

AGUAAACUUCAUGAUGUUCUUUGGCUGAGAGGCAGCCCAGGACAAGGCUUCAGUAGAGCU

UCCAGAGGGGUCUGGAGCAGGAGCUGCCAACGGAGAGGCCGGCGGGGACAGUCAGGUCUU

GACAGUGAGUGAGGCUGACGUCUGGCUGGGAGCCAGGUGGACCAGCCAGUCUGUGCCCGU

CUGCGGGGGCAGCUGCUGUCCGGAGCCGGGCUGCCAUUGCUGAGCGCAGGAAGGAGCUCU

CUUGAAUUGUUAAAGGAGAAGUCGGGCACCCACAUCCCCUGAAUUGCAAAUAUUGGCAUC

UGAUUAUAAAACGUCUUUCUCGAUUGCCAUGUUUGUGGGCUGGGUUUAGCCUGGGGCCGC

CUGGGAGCCCCCCUGGCGGUCGUACUUGGCAGGAGGCUGCCAGCUGGGGGCUUUCUGUUG

AGGGCAGUGAGAUUUGAGGCCCAGCUCAGAGGCCUCACAGAAUGCCCAUUGGAGGUGGGG

UCCUGGCUUGGCACUGGCCAGAGCAUUGCAGAAACAUUUUACAUUGUAAGGGGAACGGCU

GUGGGCAUUGGCAAGCUGGGCUCCAUGCCCGCAAGUGGGGGCAUCGUGGCAUCACUUUCU

GAGGCUGCCCCUUUUACUCGUCCCAUUUGGCCGGGGUUCAUGGAGGGCUGGUUGCUCCGG

CUUUCAGACCCAAUCUACAUGGAAAAGACCAAGGCAGAGAAAAGAAGGAAACCCAGGAAG

AGGCGCGUGGGGCGUGGGGCGUGGGGCGGUCCCGAUGCACAAGGCUUGGAAGAGGCUAUU

CAGGACCUCAAAUAUCAGGGAAGAGAAGGCUGAAGCCAGGGAAAAGAGAACCCGGACCUA

UGUUUGGGGAAGGCUUUGAGUUUGCCCGGCUGUGGGCUGGAGUCCUUGUUUCCCAUAAGG

GCUGGGGGUGACAUGGCGACGUCAGCGCUGUCAUUAACCAUAGCAACAGCGGUGACUGUG

AUAACAGGAAAGACUUCCAUCAUGUUUGCUCUGGGCCAGGCCUCGUCCCAAGUACUUUAC

CUAUGUAUUAACUGAUCUGAUCUUUGUGAUAAUCCUGUGAAGUAUGUACAAUUAGCAUUC

CCAUUUUACAGAUGAGAAAACUGAGACACAGAGGUUAGGUCAUCUGUCCGAGGUCACCCA

GCCGGUACGUGGAAGAGGCAGGAUAUGAGCCCAGGAUGUGAGCUCCUGGCUCCUGCCCUG

AAUGCCUGUGCUGUACCCUGCUCAGCGUCCUCUUUCUCUGUCCCACAGACAGUGGCUGAG

UGCGUCCUCUAUUACUACCUGACUAAGAAGAAUGAGAACUAUAAGAGCCUGGUGAGACGG

AGCUAUCGGCGCCGCGGCAAGAGCCAGGUAAGAGGCAAGGUGGGGGUGACUGUCCUUGCA

GGCUCCCUGCAUGCUCAGCGGCCACUUGCUUGGUGGCCAGCACCCCGCAUGGCUGGCGGU

AACUGUCUCCAUGAGUUGUUGUUGCUUGGCCACCGGCAGUGUGAGUGCAGGCUCCCCUCC

GUGGUAGCAGCAGAUGUGGGGAGACCCCCUCUGGGGUGCUCACCCUGCCAGGCUAUCUCG

AGCCUCCCGUAGUGGCCAGGAAGAUAGCUGGGGAGAAACAGCCACGGGCCUCCUCAGCCU

GUGAAAUCCCAGACUCCUGACUGGGUGACCUGUGAUCUCUGUGCGUAAAAAGGCUACAUU

CCAAAAGUAAGAGCUUCCCAGGAUGACUUGGGGUAGUUCACAAACUUCAUGUUUAAAAUG

UUAAUGGUUGUGUGUGUUUAUCUUAAUACAUACUUGAAAGAAAAAAUAGCCAGUACUUCA

GCUCGCCGUUUUUUCAGACGCCGUUGCUUGGGAUGAGACUGAAAGAUUAUUUACGCUAGA

GCAGAGGCUGGCAGACUGGGACUCUGGGGCUAAUCCGGCCCACCAUCUGUUCUUGUGAAU

AAAGUUUUAUUGGAACACAGCCAUGCCUGUUUGUUUACAUAUUACCAUGGCAGCGUCCUU

GCUGCUCUGGUGGAGUUGCAUGGUUGUGACCAAAAGUGUAUGGCGUACAGGGUCUCAAAU

GUUUACUAGCUGGCCCUUCAUAGAAAAAGUUUGCUUUUUCCUCCGAGUUAGAGGAAAAGG

AGUUGGUUUGAAAAAAUAUCCAGUAAAUAAUGGUAGAGAUGAGAGGCUCAGUGGCACGCC

UUUGAAAGGGGGAUCCUGGGCGCUCGGUGUUUGGGAGAAUUUGUUUUGCAUCCGGGCUCU

UGAAGCAUCGUCUCAGAGCAGAUGCUGGGAGCACCACACAGAGGAGGCGGGUGGAGGCAG

UGGGCACUCUGGCAGCUGCUGGAUUGGAGUCGGUCGAGGCAUUUCCUUCCUGGGGUCCUC

AGUGGCUGGGGUGGGGGCCGGGUGAGAGUGACAAAGGCCAAGAAUAGCACGAGCCGCGUG

GAGCGUCCUUGCAAACUGGGGGUGGAUUCUGGAAGCACUACGUGCCUGGCAUCCCAGUGA

AGUUUUCGUAGGCAGGAGUGGCAUGCCUCAAAGGGACUGGAGGGACCAGCUUUUCUAGGU

CCCUCUGAGGGUCACUGACUGCUUUCUGACACCCCUAAUGCCAAGCCAGACGUGUGGCCU

ACAAGUCCCCAUCUUCUUUGGACAAACCUUUGUGUGGACCUGAGGCCUAAAGCCCACUUU

GUGUCAAGCCCAGGCCCGGAGCAGCUCCAGGUGGCCGGUAGACACAAAGUGUCCCCAGAG

AAUGGUAAAGCCGUUGUUUUGCACCCACAAGGCUGGUUGGACUGUGCCUGGAGAGGCUGC

AGGUGGGAGAAGGUGGCUGGUACUCCCUAUAGUGGCACCCUCUAGCCCCUCCCCAGAGCU

GCCCCGCCUUCCCUUUCCCAGGCUGCCAGAGGUCGAGACCCAUAAGGUGUUGAGACCAGC

CAUCACGGGUUGGGGGCGGGGUCCCAGUGUCUGCCGUGAAACAGCCACUUUCCUACAGCC

UAAGAAAACUCCCAUGAGGGAAACGAGGCUGUUGUCAGGAGCAUGAGCCACCCCUCGCCG

CAUUCCCAACAUCCAGCCACGAUGCCUGGGGCUGAACUCGGUCCUUAUCCGCAGGGAUCG

UGGAAGGGUGGCUGCGUUUGUGGAGAGGGUUUUCUUGUCUAGUUUUCUGUAGCCAGAGUU

GACCAGUCUGCUUGCCCUUCUAACAGAUGUGUAAAACCAAGGCCGAGUGGCACAGAGCAU

GUAUUCCAAAGACGUAGCUAUGUUUUAUUUUUGUUUUUUUGGGAUUUGUUUUUUUUUUUU

UUUGAGAUGGAGUUUCACACCGUCGCCCAGGUCAGAGUGCAGUGGCGCGAUCCCAGCUCA

CUGCAACCUCUGCCUCCUGGGUUAGAGUGAUUCUCAUGCCUCAGCCUCCCAAGUAGGCGG

CAUUACAGGUGCGUGCCACUGCAUCCUGCUAAUUUUUUGUAUUUAUAAUAGAGACAGGGU

UUCACUAUGUUGCCCAGGCUGGUCUCGAACUCCUGACCUAGAUGAUCCACCUGCCUUGGC

CUCCCAAAGUGCUGGGAUUACAGGCAUGAGCCACUGUGCUCAGCCGCUACGGUGUUUUUG

AAAUUGAAGUGCAUUCAGGAAUUUUCUAGACUGAGAGGCAAAAAGGUAAGUGCCAGUCAG

GGAGUAUAUAGAUGCUGAGGUAUGAGAGGCAGCAGGGAGCGGUGGGGAUUAUGACAAAGG

AAGGGUGGCUAACGGCCUCAAGAGGUAUUUGGAAAACAUCAUCAGGCGUCUGUUGGGCCC

CUACUGUAUGCUCAUUCUCUUGCUGGCCUUCCCAGUUCCCCUCCCAUGACCUGUUUGGGG

GCAUUGAGGCUGGGCUUGUGGUGGGAGCCCUUCCUGGUAGCUGGGCUGGGGGCUCUGAAG

AGCAGAGGCCCAUCCCCUCCCCCUGUGUGAGAGCCACAUUUGCACAGAGGGAGGUGGGGA

GACCAUGGUAAUUUAAGAAGGGUGGGGGGUGUCGGUGCCCGGCAUAUCCUGUUGUAGAGG

CCUGGCCUGUACUGUGGUAGAGUGAGUGUCCAGUAGAGUGAGUGUCCGGCUUGCUCUUCG

UGGAGUGAGUGUCCGGCCUGCUCUCCGUGGAGUGAGUGUCCGGCCUGCUGUCCGUGGAGU

GAGUGUCCGGCCUGCUGUCCGUGGAGUGAGUGUCCGGCCUGCUCUGCGUGGAGUGAGUGU

CCGGCCUGCUCUCCGUGGAGUGAGUGUCUGGUCUGCUCUCCGUGGAGUGACUGUCCGGCC

UGCUCUCCGUGGAGUGACUGUCCGGCCUGCUCUCCGUGGAGUGAGUGUCCGGCUUGCUCU

CAGGAGUGAGUGUCCGGCUUGCUCUGGGCGGAGCGAAUGUCCAGCUUGCUGACUCAUCCA

UUGAUUGAGCAUUUAUUGUGUGCAGGACAUGGGGAGCUUCAUGCUGGUUGUUCAGAGCCA

UUGUAAACAGGUGGCUUCAAGACAGUGCCGCGGGUCUCCCUGUGCGGCGGGUCUAUCUGU

GCCACGGGGUCUCCCUGGGCCACGGGGUCUCCCUGUGCCGCGGGGUCUAUCUGUGCUGCG

GGGUCUCCCUGUGCCACGGGUCUAUCUGUGCUGCGGGGACUCCCUGUGCCGCGGGGUCUA

UCUGUGCCGCGGGGACUCCCUGUGCCGCGGGUUCUAUCUGUGCCGCGGGGACUCCCUGUG

UCGCGGGUCUCCCUGUGCUGUGGGGUCUCCCUGUGCCACGGGUCUAUCUGUGCCGCGGGG

ACUCCCUGUGCCGCGGGGUCUAUCUGUGCCGCGGGGACUCCCUGUGCCGCGGGGUCUAUC

UGUGCCGCGGGUCUCCCUGUGCCGCGGGGUCUCCCUGUGCCGCCGGUCUCCCUGGGAGGU

GGCAUUGGUGGCACAUCCUUGAAGACUCCGUAGAAUAAAAGGCCCCAAAGAUGAGGUUGA

GGAGCAGCCCCACAGGCGGACAGGGAUGGGGCCCUCAAGUGCAAAGGCAGGGAGCAUGAG

AGACAGCGCGUGUCUUCAGGAACAGCAGGGGGCCUGAGUCUUUGGAUUCGUGGGGGUCCC

GGUACCUCCCAGGGCCUUAAAGGAACAAAGAAAGGGAGAUUAUGUGACCCUCUUUCUUUU

GCUCAUUUUUGAUGGGUAAAUAGAGAUGUUUUUUUUUACCCUGUGAAAAAUAGAAGUGCA

AACUCGAUAAAUAACAGCUCACACUUGCCUUCGUGUCUGGGGAGGGAGUAGGGAGUGGUG

AGGCCUGUGGCUCACUGCAGAGCUUGGGCCCCGUCUCAGCUCCAGUGGACGCUUGGCCAU

UGGCCCUGAAUUGCCAGGUGUUCUGAUCUUUGAUGACGAGCUGGAAAUCCAGUUUUAUGU

GAAACUGCCUGAUUUUAAAUCUUAGGUCACAUUUUUUAAAAAGGGAAAAAACAGCUCAAC

ACUGUAUAUGCCACACAACUCUUACUUGCUGUUGAAAUCACCCCCAGCCCCAAGUAGAAG

ACCUCUGGGUUAGACUACAGUGAGACCAGGGGAGGAGGGCAUUAGAUGUCUGGGGCCAGG

CUGGGGCACCGGGGAAGCCCUAGAAUGUUCCAUAGCAGGGAGGAAACAUGCCCCUCUUUA

CACCGGAGAUGUCCUGGUUUAGCCGUGGUAUCUGCGGUGCCUGGGAGUUGGUGGGGCUUU

GCUGGUGAGUUUUUAUCCCCUGCUGAAGCUGCCGUUCCAGGCAGGGGCUCCCAGGAUGGG

GAAAUGAUGCUUGUCUGCUCUGCGUCUCUCAGAAAGAUGGCAGAAAGCAAGCCGGGCAGG

AGGUCCCUGUCUGUCUCCAGGGCUCGCCCCUUCCUCGGAUCAGCUGCGUUUGCUAAGGAG

GUGGUGGCGUGGCCUGGGCCCUGCCACCCCCCACAGGCCUGGUUCACAACCACGGGGUAA

ACAGGGGUUCUCAAGUGCUAACUCUGGGGCUGAGAGUCCUCUCUGCCCCAUGGCAGCAAG

GAAAAUCAGCGGCAGCUUCAGGCUCAGCCUGUCUUGUUAAGUGGAGAUGCAGUGACUGCC

GCCAGCUGAGCGUCUCUGUCUGUGGCUGUAUUUGUGGAGCCACUGACACUGCUGAUCAGU

CUGCCCCAGGUGAGGGCACAGCAGGCCUGGGGUGGGCCCAGCACCGCUGCUUGGAAGGCU

GCGGUGACGGACGGGAGGUGCAGGCAGUGGUGGUCUCUGAGUGCGCCAAGGCUGUGAGCA

CACAGAACCUGCAGCUCCUUAAGGAGGGUGGGGAGGUGAACUGAGACCACUCCCCACCCA

CUACCCCGCUGCCUCAGCAUGGCUGGCACCUGCAGGCUGGGACAGGCAUCUGGGACUCUU

GGUGACUGUGGGCUGGGGAGCCACUGCCCUGUGCUCCAGGCAAUGCAGGCACCCAGGGUA

CCUCUGUGGGCCGGUUCUUCUUUCUGACCUAUCCUGGCAGCUCAGGCCUUGUGAUCAGAU

GGGAAUCCCUGGCCAUGGAGCACACGAGCCCCUGCCCAGCCCCACUCCCCUCACAGCUGC

CCCAGGACACUUGUCCAUGCUUCUCUCUCCCUCAUAGGUUUCUGAGUGAGCAGACACAGC

CCCUUCCCCCUACCGUGACAUGGGCCCCCUCCCCAGGCACCACGGGAAGAUGGAGAACCC

GGGUCUUCCCAGCCAGUGAGCCCACCCACCCUCAGGGGUAGGGUGCCCACUGCCAGGAGA

UGCUGUCUGCCUCUUCCCCGAUGUCGUCUAGUGGGAGGGAGCUCAAGCUCCCUUCUCCGU

GAUGUUUACCUGGAGGGCAGCAGGUCGGGUUCUCCCUCCCUUCCUGGGCCCUCUAGUUUU

GGAAGAAGACUUAGGUUAUCUCAGCCAAUAUGCAUUGGACCCCUUGAUGGCUGGUGGGGG

AUGGGACCUGGGAGUCCAGUGAGACAUGGGAGCCUUGUCAAAAUUCCAGGGGCACCUGGC

CCUGCUGUGUGAUGGGGAUGGGUGCUGCUGGCCGCUAUGGUGGCGAUGGCGAUGAUGGUG

AUGAUGACGGCGACAGUGAUGGUGCCGGCCAACAGUCAUGCAGCACUAUCGCCACACACU

GUGAUGAUUUCAACCUCACUAGUGUUGGGGACAGGUGUGUAAUUAUCCCCACUGGCCAGA

UGAGACAGAGGCACAGGGAGGUGAGGAAGCUUCCAAGGAGCCUGCUGAGUGGCAGACUGA

GGGUUCUAGCCUGGCACAUGCAGCCCGGGGAGUUGGAGGGAGGCCCAGCCGUCCCCUCGG

CAGCACACUGGCCAUAAAGCCAGCCUUCUGGAAGCCAGCCCUGAUCCGCCAGGUAGGACA

GGCCUCUCCUGUGGUCUGUGCAGGAGCCCUGCGAGGGCAGGCGGCAGAGGGGCUCAUGAU

GAGGCUCCAUGUCAGUGGGGAGUCGGAUGCUGGAUGCGGGGACCCCAGGUGAGCCAGGCU

GGAUCUGUGCUGUGGAGACACCCUCUGAUAUGGGGUGGGUGGGAGUCCUGGGGUGGGGGG

GGCUCAGUACCCCCCGCCACCACCUGUGUCUCUUCCCCUGCCAUGUGUCCCUCAGCUCCU

GGGGACAGCAGCUGGCCACUGUCUCUUUCUCUCUCAGUGCCUCCGCCGUUACCAUAUAUG

UCUUUGGGGAGUUGCUGAGGGCUCUUCCUCAUCUCCCUCUCUCCCGCCUUGUCUCUCCUC

CCCAUCUCUGUCUCCCGCUCUGCGUCUCUUGCGCUCUCUCAGCACCCCCACUCCCAGUGC

ACAGCCCCUCUCUCCUGGGUUGCUGUGGAGGCUGCUGUGGGUGGCCGCAGAGAGGCUGUG

GUGCUUGUGGUCCUGGCGCGGUGCCCGUGGCUCCAGGCCACAUCUCUGCUGAAGACAGGC

ACGAGUGGCCUCUCUCAUGUCACCAGGACAGAGGCCGCUCGCAGGGGCCUUCCUGCCUCG

UGUCCCCAAGCUUGCUCGCCCCACAGACCCCCCAGGAGGUGCCCAUCGGAUGCACAGUAU

AUGCAUGUUCAUCCGGGGGCAUUUAUUCAGUGCCUACUGUAUGCCAGGGUUGUCAGACUC

UACCUUCAGAUGCGAAUCCGACCUUAAUCCUUCCGAAAAUAGGGUAAAGGGAUCAGCCGC

GCCUGGCCCAUCUCAACACCCCAGACUGCCCUGAUUCCACGCUGCAGAGGUGGACACAGG

GUAGGACUUCCGAGGUGUCGGGGAGUGGGCACGACCGCCCCUCCCAGGGUCAACAGCGCC

CACGUGCCUCCUGUGGGUUUCCUGAGUUGGGGGGUGCAUGGCCCAGGACCAAGGACAUGG

GUGCCCCUCCUUUCGCCAUUUGUCAAGGUGGGAUUGAAAUGGAGGCUGUGGUGGGAUCCC

GAGGGAGGGGAAGCCUGUGGGCUGGGGGUGAGAAGUGACUCCAGGGACACUGGACCGUGC

ACUUUCCCCGGCAGCGAGGCUUCCGACUUCCUCAGACACCACUGACCACGCUGGGAUGGC

UGUAGCCAGCAUCCUGGGGCUGUGCCCUCCUGGAGUUCCCCCUGUCUGAGGCCAAGGUCU

GCUUUGUGAGGGGACGUGUCCCAGUCUGAGUCCUCAGAUGUGAGUGCAUGUCGGAAUCAC

CGGAAGGGCUGGGGAAACCCAGUCACUGGGCCGCACCCCUCACCCCACAAUAAGGCCGAG

AUCCAAGACUCUGCAUUUCCAAGGAGCUCCCAGGCGUUGGUCUAGGACGCUGCUGAGAAC

CUCGGUGCUGGGGGUCAGCCCCUGAGCAGGGAGCAGGGUGGCUGAAGGGGUUGUUAAGCC

CGAGGUGGGCUACCCCACAGCCCAGACCACCCAUGGGAGUACACUCAAGCUGGAACGGCC

CUUCCCAGGUGUUUGGGGUGGGCAACGGCCUCCCUGGCCAGGGCCCAACUUGGAUGAGGC

AGCUCUGAGCUGAGUUAUUGUCAGCGCGCCUGACAGCCGAUGGCAGCCAGCCCCUUCUAG

AAGGGAGGUCUGGCCAGUCCAGCCCAGGAGGCAGCCUGUGCAUCUGCAUGUUUGCAAAGC

CACGCUUCAUAGAUGCUGUGGCCACCCGAAUGCUUAACCACAGCCCAGCAGCAAGACGCC

AGUGCCACCGUGUCAUCUCAAGGACACCUGCCCUGUCUGAAGUGCUUGCACCAGGCCCUU

CUCCGCCCUGUCACCCCGACACCAUGUGCCCUGUACCAAAGCAGUUUGUGUGUCAUUUCG

AUGGUUACGUUUCUGAAACUUGGAUGGUUAUAAAGUGGAAUAUGAAAGUCCUUGAUUAAA

AGUGCUGGCCAUGGGACGUCUCUGGACUCAGACCCGCGCUGUGUGCGGCCGCCUUCACUC

UGGCCCACCUUGGCAUCCAUGGCACUGGUGCUUAGCCUUGUGGCCGGCUGGAGGCGAGGG

AUGUGCAUGGCUGUUUAGAGUGGUGGGAUUGCCUUCUUGUUUAAUGGAGGGUGCCAUCCU

CUUUCUGGAAGCUUCUCUGUGUUCUGACACGGGGAUGCAGGCCCGGGCAUAGGCUUUUGG

AAUGCUAGCCCCUCCACACCCACCCCCAUGAGACCCCACAGGCCUGAGCUUUGGCAAGGG

AGGGCCGAGCCCCCACCUCCCGGAACCUGGCCCCUGAGAUCGGCCGUGAGCGGCUUUUGA

GCCUGGAGGCUGGGUUGAGGCCCGAGCAUCCUCUCUUGGGCUCCGAGCCCCAUCACCACC

ACCCUGAGGUGAAUCACGGACUGUGGGGCGGUGCGGGGAACAGCUCAAGCGCUGGCACAG

UGGUUUAUAGUCCAUGAUUCAUUGAUGGAAGGGCCGCGUUAUCCUAGUGAAUUGCAAAAC

CAGAGCCCAGGAAUGCUGGGGCCUGACUGCAGACCGUUGGGGAGCGAGGAUGUGAGGCUG

GCUAGGCCAGGUGGCAGGGCCCGGCCCCUGGUAGCCAAUAUGGCCUUCCCGCAGCGAUGG

GGGUUCAUGUGGCCAAGGGGGCCUCUCUUUCUGCAGAGAUGGGACACUGAGACGGGAACA

GCCCGUCCUGGGCUCCAGUCAUCCGGCCUGGGUCCAAGAAGGAAGCCCAGGGAGGCCGAG

GCUCCGCCCCCGACUGCUGCCGUCCACUCUCCACUGUUCCCCAUCUCUGUCCCUCUGGGU

CUGCUCUGCCCAAGCCCCUGGCUCUGAAUCUCCUGCAUUUUUCUAGGUUUCUCUCUCUCU

CUCUCUCUCUGUGUGUGUAUGCGCGCACACGGGUGGCUACGUCUGUUUCUCUUCCCAUGU

CUUCCUCCCUCUUUCUAUUUCUCUGUGUCUUUAUCUCUGUGUCCACGUCUCGUCUGUUUC

UCUGCAUGUGCUUUCCCGUCUCUCUCUCUUUCCCCCAUCUAAGUCUUUUAACUCUGUCUC

UAGCUCUCUCUCUCUCUCUCCCCUUCUCUCUCUCCCCCAUCUCUCCUCUUCCUCCUCCCC

CGCCCCCUCCCCUCCUCACCCUGUCCCUGGGUCUCUCCUCUCACCCUUCCUCCUCCAGGU

CUCUGCUCCUCCCCCACCUUUCUCCCUUUCUCUCUCCUCCCUCCCACUACAGCCCCCUGU

CCCCUGCCUCCUCCGUCUGUCCUCUCCCAGCUCUUUGCGGAGGGACUGGUGACCUUGUUC

CCCUUGCUGUGGCACUCGGACAGUUUGCACAGGGAACAGUAUGACCGGGAAGGCUGUUCC

AGGGCAGGAGCAGCCAGGCUUGGUCCCUGGGGAGGGGCAGCCGCUGUGCGGUGGGUGUUG

GUAUUCAGGGCUAGCUGCCUGCCCUAGGUGCUGAAGUCACUUGAUGGUGGGGACCGGUGG

CAGGGCGGGCCAGAUCACCCCAGGCUGUGUUUCAGCUCCCAGGCCGAGGGCUUGUAUCUU

GUUUCAAACCUGCUGCCUGCUUGGGGCUGGGCCAGGAUGGGCCCACAGCCUUCCGGUGGU

GCCGACUUUCCCAGGCCUGCCAGAGGAGGUGGCUGGGUCCUCUCCAGUCAGGGCCAGCUG

GGCUGGGCAGGAUUGGGUGCAGUUUUAGGGUUGAAGUGAGCAGAUGUCUCUGGUUUCCUG

CCCAGCUUUGUGCUAGGUGGGGAGACAGUUGGGGGCUCCCCACGUCCUGCAGUCAGGGAU

GUUGCUCCCAGGGAAGGCAGCUGGUGAGUGGGCCGGAGCCCAGGGAUGGAGCAGACCGGG

CUGCAGGUUGAGGAAAAGACCAACAUGGCCUUUGCAUGGGCCAGGAUGCAGUGCAGGGGC

GAUCGCAUCAGAGAGGCAGAAGGAGGUGGAAAAUGUGAGCGCUCCCCAGGCAGCAGAUCG

GUCAGGAUCGAGGGGGCCUGGGGGACAUCUCAAGCUGGGCUGCUGGAGUCAGGAAGUAGU

UCCCAGAACAGCAGCCUUGGUGUCUGUGGCAGCUGGAGGCACUGAGGGAGGGGCCCUGGC

AGGCUCACAGGCGGCAGGUUGUCCCUGGGGGAUGGGUGGUGUUUGAGUCUGUUCUGCAGG

GAUGUCAUUCUAGGUGAUUCCCUCGUGGACAAGACAAACAAGGUCACCCCGGUGCGGUGG

CUCAUGCCUGUAAUCCCAGCACUUUGGGAGGCUGAGGUAGGUGGAUCGCUUGAGCCCAGG

AGUUCAAGACCAGCCAGGGCAACAUGGUGAGACCCCAUCUCUACAAAAAAAUUAAAAAAU

UAGCCGGGUGAGGUGGCUUGCUGCUGUGGUCCGAGCUUCUUGGGAGGCUGAGGUGGAAGC

ACUGCUUGAGCCCAGAAAGUGGAGGCUGCAGUGAGCCGUGAUCAUGCCACUGUGCACCUC

AGCCUGGGCAACAGAGCAAGACCCUGCGUCCAAAAAAAAGGGCAAACAAGGUCCCUGGAG

CUGACACCCAUGGGGAACUUGGACCAUGUGCAAGCCUCCAAAAAAAAGGGCAAACAAGGU

CCCUGGAGCUGACACCCAUGGGGGACUUGGACCAUGCGUAAGUCAAUCAGCAUGGCAAUU

UGAGAGAGUGUUGAAGGGCAAACAUGUAAGCCAGGGUCAGGAGGCCACUGCUGGGCAGGA

GGGCAGCUUUUAUAGGGAGCUGGCCGCAAGGCUUCUCUGAGGAGGUGUCCCUUGGGCUCA

GACCUGAGUGAUAAGAAGGGACUGGCCAGGCAGAAGUCGGGGGAGCACCCCAGGCCGUGG

GCACAGCACGUGCAAAGGCCCUGAAGCAGGAACAUGCGGGCCAUCUUCCAGGAGACAGGU

GGUGGGCAUGGGGAGAGCUGCCCUGAGGGAGGUGGGCCAGGGCCAGGAAUCCUGGCCUCA

GAGCAGGAGCAGGGACUGUGGAUUUGAUGCCGAGUUGCGGGUCGGGAUCUGAAUUAGGAU

UUACAACAGAGCCUCGGCUGGCACAGCACACAUCUGUACAGGCGGAACAGGCUUCCAUGU

GCGUGUUAAAUGUUGCUCACGUUCUGUCGACACGCAUCUUUCUGCCGUAGAGUGCUGGUA

AAUGUUUAACAACCAGCUUUCUGUAGGGGGAAGCUGCGUUUGUAGCAUGUGCCGGUUGCC

AUGGUGUAAAUAAAUCCUCUCACCGUGCCCAGUGCCACGUGACAACGUGCUGCCGCUGAC

UUGGAACUGGGGAAAGAUGCCCCUAGCACCAGGCUGUCUUAUCCUGUUCUCACCCUGUGC

AUGGAGUUGGCGGAAGUCACCUCCACGGCACGGAUGACUUCGUAGCUCCAUGUAGCCAGA

UGAUCAGGAAGCAAUGCCCUUGGAGUGCUUGUAACCUUCGCUUUCUUUCUUUAAAAAAAA

UUUAUUAUUUUUUUAGAGAUGGAGUCUCGCUCUGUCACCCAGGCUAGAGUGCAGUGGUGC

AGUUGAAGCUCGCUGCAGCCUUGAUCUCCCAGGUUUAAGCUUUCCUCCCACCUUAGCCUC

CUGAGUAGCUGGGACUACAGGGAUGCACCACCUAAUUAAAAUUAGCACCACCAGCUAAUU

UGUAGUGACAGGGUCUCACUGUGUUGCCCAGGCUGGUCUGAACUCCGAGCCCUAGACAAU

CCUCCUGCCUGGCCCUCCCAAAGCACUGAGAUUACAAGUGUGAGUUACCUUGCCAGGCCA

UAAUUUCUUUAAUUGUAAGGUUAAGUGUUUCCAUUUUUAAUAAUGGCCAUGAGCUGGUAG

CCACUGGCUCCAGCACGCCUUGGUAUAUCUUUUUUUAUUUAUUUUUGUUUGAGACAGAGU

CUCACUCUGUCACCCAGGCUGCAGUGCAGUGGCGCGAUCUUGGCUCACUGCAAGCUCCGC

CUCCCGGGUUCACACCAUUCUCCUGCCUCAGCCUCCUGAGUAGCUGGGACUACAGGCGCC

CGCCACCAAGCCCAGCUAAUUGUUUGUAUUUUUAGUAGAGAUGGGGUUUCACCGUGUUAG

CCAGGAUGGUCUCGAUUUCCUGACCUCAUGAUCCGCCGGCCUCGGCCUCCCAAAGUGCUG

GGAUUACAGGCGUGAGCCACCGCGCCCCGCCACGCCUUGGUAUAUCUUAUAAAAAGACAA

CCCUUAAACGCACUGAGAUGGCCUCCCCUGACUGCAUUUUCCCCAGCAAGGACAGGAGAC

UGAAUCCGAGGGACAGAGAGCAGGUGUUAGGGAAGAGCUGCCAGCAACAGGGAACCCUGU

UCUGCCUCGGCUCUGACCCCUGGCCCCACUUUCCACACCAACCCCAGGCUCCUGGAAUCC

CCAUCCCUACACUGCCAGGAGGUGGAAGGUGUCAUGAGGAUGCGCCCUCUCAGGGGCACC

UGGCACAUGGUAGAUGCUCAGUGAAGUGUUUGUUGAAUGAGUGCAUGAGAUUGAGCCACA

GACGGUGUUGCUGGGUUUUGGGAAGUUCUCCUGACACAUUUGUAAGGACUUAGCAUCUGA

GAAUUAAAUCUCAUCAACCCCUACGUGGGUGGUCGCAGCACCUUCUCACUGGACUGUCUC

CUCUCAUUCCCGGAAUCCACCGUCUUCCUUCCUGCGACCUGCUAUUGGAUUGCUCUUUAA

CAUGGGGUGGGUCAUGUGACUCCCUGCGACCCCUGCACCUUUUGAGAAAGGUCCCCGGGG

GCUCCCACCGCCCUCAGAAUGCCUCCUGUAUUUCUGAGCAUGGGUGUUAGCCCUGGCCAG

GCCCUCCAGCCAUGCCCCAAAGUGCCACUUGGUAGGCACAGGGGCCAUUUUCCCAUUGCC

AUGCCGCAGAGGCCUCCUGCUCUGCCAAAGCCCCUUCCCUCGCCCUGCAUGUCAGCACGC

CUUGCUGCCUCAUCUGCAUCAUGAAUGUGCCUUUUGAAUGUGUCCUCGUCACCGGCGCCU

CCAUCUGGCCUGUCUUUGUCAACCUAAAUACAAGCUCCAUACAGUUGGGGGCCACAUCUU

UCUGUGGCUGGUGUGCCCCUUGGGGGUUCAGGUUAUGCUGGCGGAAGGAAAGAAUCAGGC

AAUGGCAUCAUUUGGUUUAAAUGGGCAUAGGGAGGAGGCUGUGAACCCCCAGCCCAGGGC

GGAGGUAGGGCACCUGGCUGUAGGACCUUCUAGGAAAUGCUGUGUGACCUUGGAUGAGUU

ACUUAAACUCCCUCUGUCUUUGCAUUCUCAUCCAUAAAACUGGGAUGAUACCAGUUGUUG

GGAGGAUUCAAAUAACAGGUAGGAAGUGAUUAGGAGAAGAAGGCCUCAUCAAAUGUGCUC

AGCGAGCUUUUGCUGUUUUUAAUUAAGAUAUUUUUCUGCAUAGCCCCCAGUCUGGGGCUU

CUGCACAGGCCCAGUCAUGUGCUGCUUGUCUGGCAGGCUGAGGGUUAUGGGUUCAGUGUC

CAGAUCUGGGGGCAGGUGUCUCUUGGGGCUUUGGGCCAGUGGCUCUGGCUAACCAUAGAU

CCAGUAGGGUUUGUCACAGAGUUGUACUGUGGUUUGGGGGGAUGUUUCUGACCACGCACA

GGGCACAAACCCACUGUCAGCCUGACCAGUGGCACAUCUGAAAGGCAGAGGUAGCCCUCC

ACCACGAGGGCGGGGUCCUUUGAGAUAGUGGAGCUGGCUUCUAGUUCUUUCUGAUUCUCC

UUUCAGUCCCCUCACGUUGAGGGAAUGGUGCCCACAGGCGUUUUCUGCUGCACAGAGUCC

UUUAUGUGCCUUGUGAGACAAGGAUUCUUGAAGGCCUCACUCCUCAGUGGGAUCUGAGGC

CAGCCGUGGCAGCUUCUCCUGGCGCAGGUUAGAAAUGCAGAGUCCCAGGCCCCACCCCAG

ACCGACUGAGUGGGAAUUGGCAGGGGAUGUGUGUGCACCUGCAGGUGUGGGUAACACUGG

CCUGGAGCAGAGUGCUCUAACGAAAGCCCACAGGCCAAAUCCGGCCCACCACCUGCUUUU

GUAAAUAAAGUUUUAUUGGCACACAGCCGUGCCCUUUUGUUGAUAUAAGGGCCGCUUUCA

CAAGACAGUGGCAGAAUUGAGAAGGGGGUUGACAGAGACGGCAUACUUAUCCUCUGGCCC

UUACAGAAAACGUUUGCCAGUCCCUGGUAAAGCACUGGUUCUCAAAUCUGGCUGCCCAUC

GGGAUAACCUGGAAUUUAACAUACGAACGCCUGGGUCUCCGCCCAGAGAUUUGGAUGUCA

UUGGUGUGGGGUGCGGCCUGGGCAUCAGGACUUCGCAGGGCUCCCCACGAGAUCUGAGUG

UGCAGCCAAUGUCAUGGGACCUUGAGAACCAUGGCCCCCAGUGGCCCGGGAAGCCCAGCA

GGUGCAGUUCUGCCGCUCUAUGGACAGGCCCAAGACCCAGCAGCCCAGCCUCGCCAGUGU

CGGCUGCUGCACUUGCUGCCCUAAAACAUAGAAGGCUUUGGGGGCUGAGCCGGCUGCCGG

GUCCCCCCAGCUCCUCAGGGGGAGUUGUGAGCUGGGACCUCCCUCUCCCUGCAUAAUCUC

CCAGCAGCGAGUAUGAGCGGCCCUCAAUCAGUCCCUCGGCCUCCCGGGUGGCCACUGCGA

GACCCUGCAGAUGUUCAUCAUUAAGGCUGCCGGGGUCUGUCCCACUGGAAAGCAUAUUUU

AAGGCCAGGGCUGCUCUGCUAUUCCUCCUUUUCAUCUUCCCCCACCGGACUCACUCAGCG

UGGGCUGCAGGGAUGCCUCUCCCACAGGAGAUGUUGAGGCCACAGGAAGUGAGGAGCCUG

UGCACAGGGCCCUAGGCCUUGGCUGCAGGGUGGCCCAGAAGGUGCUGUGCUAGUGGGUGU

UGCUGCACCCACUCCAACCAGAGGGUGAGGGUGGAAGGCACCCCUGCCCUGGGGGCUUGU

UAACAUGUCAGGGAAGAGGAGGCGGCAAGGCUUGGUGCCAGGAUUGCACUCGGUGCCUCA

GUUUCCUGUUCUGUAAGAUGGACAGGUGGGUGGGUAGGGGAGACCAUGUGGGACGGAGCC

AGGCACGUGGUGCCAUGAGCGAUCGAAUAUGGAGACAUAGGGGGUGACGUUCCAGGAGCG

AGGGGGUUGUGGGCAGGCCCUGGCGGGGCUUGUAGAGUCUGGAGUGGGAGGCAGGUAGGG

AUAGGUAGAUGGGGCGUGCUGGCAAGUCUGCAGAACACAAACUGUAGAUCCUGGCUGGGG

GUUGGCUGAGACUCUCAGCGGGGGCUCUGGGGGCAGCAGGGCCCCAUCCCCCACACUCUG

GGCCGUCAGCCUUUGCUCCGAGACCUCGAAGCUUUGCCUCUCUGAGCCUGUUUCCUCCUC

UGCAGAAUGUGACCAGUGAUCACUGCUCUCCUGUUUCUGAGGCUGGGAUAGUGUUUGCAA

AGCACUUGGCAUGGUGCAGGGGUGUUACUCGUGGCGUUUUCAUCUCCAUCAGGACUUCAG

AGGGGCCAGGAGCCGGCCCAUAGAAAAUGGACAGCACAGUGCUGGGCACAUAGUCAGCUC

UCGGGGAUCUGUGGGGAUUUUCUCCUCUGUACCAGCCUCUUCUUCCUUGGGGCUUUUCUG

AGGACUUGGAGGAGAGCUGCGGUGGGGAAGGCUCGUAGCUCAGGGCCUGGCCCAGAUGCA

UCCCUCUCCCAACUGGCCCCUGCCUGCGGCCUGAUGGGUGUGGAGCUGCAUUUACUGGCC

AUGCCGGAGCAGACAUUAGCCUGAUUCGUUGGUCCAUGGGUGAGGCCCCAGGGGGUCAUG

GAGCCCCCACCAGCCUAGGUGCAGGGGCUGAUGGGCAGAUGCUCUGGGAAGGAGGCUCCG

UGGCUGCUGCUCCCAUCGGGAGGGACGCCACAGCCCAGGUGGGGACUUCUUGGCACAGAC

GUAGCUGAAUGUGGUCUCAGGAGACACCAAGCCCGUGUGAUUCAAAAGCCCCGGCCCUGC

CCAGACUCUGGCCCUGGGCUGUUGCCAGGGACAUCUGACCCGGGGAGACGGCAUCCAUGC

CGGGCAGCCCAGGGGCCAAGGCUCUCUCCCCAGCCAGCCACAUCACUCCUCCCUCCCCGG

GUCCCUACCUCUGCCCACAUCGGGCAAAUGUGGACAGAAAGGAUACUGUGUAGGAACAAG

CCAAAAAAACCCUCCCUGCCCCAGCUUCCACCUCCAAAAGAGGCUUCACCUGCCGGCUGU

UUAAACGUUACGUUUGAGGGUUCGAGGUCAGGAGAGGGAGAACAGGUUGAUCCCAGGAUG

CCGGGCACCUGUUGGGGAAUCAGCAGCAACCUGGGGGCCGGGGGGUCUGUUCCAGGUCGG

GCAACUCUGCCCCUUCCUGGCUCCUCUUACAGAACAUGGUGGCAAGGGAUUCCUGGGGAA

AAGAGGCAGAUGCAGGCCCGAGGCUCUGCCCUGGACCUGGCAGCAGCCUCAGGGAGCCCA

CGGUUGCCUGCAGCAGUCAGACACUUGCUCUGUUUUUCUGCCCCAUCCUAAAUGAAAAGA

GGCUGGAGAGAGCGUUGUGUGCGGGUCAGGCACCUGUUCCUGGCAGGAAGGAAAGGCAAG

AGAAGGGAGUCUCUCUCACCUCCCCGAUCCCUCCUCUUCCAUCUGCUUGGAGUUCUUGCU

CCCUGGUUUGAAACAUUGCCGUGACUGUGCCCCUGGUACACAUUAAAAGGCUUUGGGGUU

UAUUUUUAUUUCGCUUGGCUCAGAGGGAUGGAAAGAUGGUCUGGUUUGGCAGCUGCCUGC

AGGGAGGACCUUGGGGUGUCCAAGAAAAUCAGUGUGUGCUUUAAAAGAAAUCAGGCGGGA

UGACAGGAAAUCGGGAGGCGGUGCCAGGAGGAGCUCACACUCCCUCCUCUAUCUCGGUGA

GGGGAGGGGCGGUAGGCCCAGAGUGGCUGGGACAGGUCGGGGAGGCAGAUGACAGGAAGA

GGCCCAAGUAAGGAGGCUGGCUUGGCUCCCAGCCUCUCUGAUCUGCUCUGUAAAAUGGGC

UUGUUCUGUCCCCCUGGGGUGCUGGCAUGCGAGGGAGACCAGGCCCAGGCAGCAGAGCAG

CCACCUCCACGCAGGCCUGGGGCCUUCCUGGGCUCUGGGGAAUCAGGGGCAUCCCCCCAC

CUCCCCGUUUUACAGACUCGGAGACCGAGGCUUAGAGAGGUGGCCUGAGCUCUGUCGGGG

GCAGGGCAGGGCUGGGGCAUCAGGGGUGUGUCCCCAGCUUCCCAGAUGCUCAGGCACCCC

UGCCGAGGCUCACCAGGGCACCUCUCUCAACCCAGAUCACAUCUGCCCCUCUUAAAUAUC

CGGGCCAGAUGCGGAUGUAAGCAUGGUGAGGAUUCUCUCAUUUCAACCUCCCAGCCCUCA

GCUGAGGCACAGGCAUGCUGAAUACCCCCAUUGUCAAGGCGAAGGAGUCGGCACAGAGAG

GUUAAGACCCUCAUCCAAGCUCACACAGCCACUGUGUGGAAAGGGCAGGAAUUGAAGCCA

GCACUUCUGACACCAAUGCUUCCUGCCCCUCACCCCGACUCCGCCUCCCCAGGCCCCUAG

GGCGGCAAGACCCCAGGCCGUGCCUCUCCCCAUCUCAUGCCAGUGGGGCCUGGCUUCCCG

GUUGUUUAUGACCUCUUCUGGGUGGACUCCACGGGUUUUCCUCCUCCUCCCCCAGCUCAG

GCUGAGCCCAGGUGCAGAGAUUCACCUGUUCACGGUUUUCUUCCCUCCCCACUCCCCUCU

GCCCUCCCCCGGGGUCUGACAGCAGCAGCAACAACAGCAGCAGCAGCAGCAGCAGCAGCA

GCAGCAGCAGCCCAUGCCCCGCAGCAGCCAGGAGGAGAAAGAUGAGAAGGAGAAGGAAAA

GGAGGCGGAGAAGGAGGAGGAGAAGCCGGAGGUGGAGAACGACAAGGAAGACCUCCUCAA

GUAAGGCAGCCUGCUGUCCCCUGCAGGGCCCGGCCGCUGACCCAACCCGGGUGGCACACG

GUUCUGGGGGACCCUUUGUAGAGACGGGUGGGGUGCCCACCAAUUGCCUGUGAGCAGGGG

GGAGGGCUUUCUGCUCGUUUGCCCCUGACAGCCCCCCUUCCGGAAGCCCACCCAUCGCCC

UACUCCUCCUCCCUGGCUCCCCCCUCUCCUCCGCCCUCGCUUUCUUUCUCCAACCCUCUG

GAGACUUUAGAGACUGAUAGCUCAUUUGAGCUCCACUCCUGAGCUAAUAAAUCCAGGCAA

UGACUUUGGAACUCUCUGCCUCCCUGCUUCCUCCCGCUCUUUCCUCCUGAGGCGCCAGGA

AGGGGGAAAGGGGGAUCAGAGGCCCUCCUGGAAGGACUAGGUGUGCAGGCGGCUGCUGUC

ACCUUUAGAAUCAUCGAUGGCAGUGUGAGUUCUUGCAGAUCAAGGCACAGCCUCACCGCG

GGGGCCGUGACAGCCUCCUGUGCCUGCCAGGGGCUGCAGGAGUGCAGCCAGCUGGAGAAA

GGGGGGCGGCACGACUUAGCGCCAGGUACCUGUAAUGGGCUCAGGAAUUUGGCUUCAGAG

AACAUCUGGCUUCCUCACCCCGUCUCCCAGACGCUUCCAGCAAGGGCCUUUCAGGAAGAU

GAAUGGGAAGGCAGAGGUGAUCAAUCUGAGCCUCCCGCAGUAAAACUCUCCCCAGUCACU

UCUGCGAGAGCCCCAGCUCGGGUAUCGAGGUUGUGAGCACCCCCACACACCACAGCUAGG

CCUGCAGGCUAGGCCUGCAUAAAACCAGCAAGAGAGUUUCCUCCUGCCAAUGUCUAAGAU

GAUGCCCGGCCCUUGAUAAUACUGAAGGCCAAUGUAUUGCCUCUUAUAGAGCCCCUCUGG

AAAUAGCUCCUCUGUGGCGAAUAAAAUGGCCCUAUAAAUUGCUGCCAGCCCACGCCCCCA

UUUAGUUCACCAAAUAGUCUUGCAGACAGUAUCUCUCUAUUUGGUGCAUAAAAUGGUCUU

GCAGAUAACCACCAGCCCAGAAGAAAGUAGCCUUGCAGGAAAGCUCCCAGUACCUCCCAU

UUGGGGAAUAAAAUAGCCAGCCAAUUAACUACCUGUCCACAUGGCCUUAUUGGUGAAUUA

AGUUGAUAGCCCAGGUUACACCCCCAUCCUUUCUUUUUCUUUUUUUUUUUUUUGAGACAG

GGUCUUGUUCUGUCACCCAGGCUGGAGUGCAGUGGUAUAAUCAUGGCUCACUGCAGCCUC

GACCUCCCAGGCUCAAGAGAUCCCCCUACCUUAGCUUCCCAAGUAGCUGGGACCAUCAGC

AUAUGCCACCACGCCUGGCUAAUUUUCUGAUCUUUUUCUUUUGUAGAGAUGGAGUCUUGC

UGUGUUGCCCAGGCUGGUCUUAAACUCCUGGGCUCAAGCAGUCCUCCAACUUCGGCCUCC

CAAAGUGCUGAGAUUACAGGUGUGACCCAGCACACCUGGCCGCAACUCCCUUUUGAGAAA

UAGCAUGGUGUUGUCCUGCCAGCUGUACUCCCACUUCUGGUGAACAUGGAGCCCCUGCAG

ACUUGCCCCUGGCCACCCUCACCUCAGGCCUCUCCCCCUCUUUCCUAGCGUCCGAUUAGU

GCCAUGGCUGGCAGCCAGUGCAAAUCCAGCUAAAGAAACAGUCUCCUCCAGUGGCUGUUU

GAUUUCCCGGCUCGGGUGAAGCCAUCCUAUCUCCCCUCUCUGGCCUGUAGGGAGAAGACA

GACGACACCUCAGGGGAGGACAACGACGAGAAGGAGGCUGUGGCCUCCAAAGGCCGCAAA

ACUGCCAACAGCCAGGGAAGACGCAAAGGCCGCAUCACCCGCUCAAUGGCUAAUGAGGCC

AACAGCGAGGAGGCCAUCACCCCCCAGCAGAGCGCCGAGCUGGGUGAGCUGGGGGCCAGG

GAUGCGGGUGGGGAAGGGGCUGGAGACACGGCGGGUUGCGCUCAUAUGAAAGUUUCGUGC

AAUUUGAGUUAAUUGGCAACAGCCAAGGGGUUGGUGUAAGUCAUGGUUGGAGUCAAAUCC

CAGCGCUACCUACUGGAUGUGCUGUGUGGCCCAGCAUGAAUUCCUGAACCUCUCUGUGCC

UCAGGGUCCUCAUGUUCUCAGUGGGAGGAAUCGGUGGUUACCUCUAACGUGGGGGGACAG

UUGUAGUCACUUGCUAUGUGAGAAGCACCUAGAACAGUGCCUGGGGUGUAGGAAGAGUUC

AGUGGCUUCAACCACAGUGAUUUCAGAGCAGGGAGAUGUCACAAAAAAUUCCCAGUGACC

AGUUCCGGAUUUUUUGGUUGUCGUUUUUAAAACAGAAGAUCUGGCCACGCUUGGCCCAGG

UUCCCAUGAGGUGGCUGUUGUUGGGAGCCGAGUAACCACCACUCCUUCUUAGAAUGUCAU

GGGCUCUACUUGUCCCCACCCAGCCUCUACCCUGGUAGGCAUCUGGUUUUGUACCUUCAC

UUCCGUGGGCCUCAGUUUCCCCUUCUGUUGAGAAGGGUGUUCAUCCCUCACUACCCUCCC

AGAAAGUAGAAAAAAAUGAGAUGAUGUGAGUGGCAGUUCCCAAGAUUCGCCGCAGGGGGC

AGCGGUGUGCGUCUGUUGUCUCUACUUUGUUUAGCUGUUAGGGUCCAGCCCUCCAGCCAC

UGCGGGAACCCACCCAGGGGCCCAAGCGCUCAGGCCGAAGCCCCGCCUGCCAGGGCUCAU

UCUCAGGGUGGAGAUGCUGCUUCAUCUCCCACAGACCCCCACCGUCCCCUGGGGUCCGGG

UCAUGCACCUGUCUCUUCUUCUCGUGGGAUGUAGUUUGGACCUAUUUCAUAAAUGGCCCA

CUCGAAGCUCAGAGAGGAAACGUUCCCUCUCUAAAAAUUUCAUUGUAGCUGGGGUUAGAA

CUCAGCCCCAUUGGCCACAGAGCCUAUGCAGCUCCUGCAGAUAUUUUCCAGCUGCCUCUU

GGUGGCGGUGUACUUGGAGGCCCAGGGUGAAAGGGCUUUCUUCCUGCCUGAUCUCUGGGG

AUAGUCCUCUCGGACCCAAGGCUGAUGCUGGGGGCUCAGCCGCAGGGUGGAAAACACACU

CCUAGGCCUUCCAUGUCUCUUCUGGCACUGGGCACCCUCAGCACCCCUGGCACCCCUGGC

UUCUGUAGGCUUCCUCUGGGAGUCCUGUGGGGAGGAAUGGGAGCAUCGAUGAGGUCCCAG

AGAGGCCCAGGCUGUGGCUGCAGAGAUGGGGGAGGCUGGGGUGGCUCUGGCCUGUCGGGG

CUGAUGGGGCUGCCCAUGGGGGUGGGGGCCGUCUCCCCUCUUCUGCUGGAACCUUCCGCC

ACUCCUCAGCCAGCCUCUGUUGGACACAUACUCCCCCUGGCGCAGUUCCAGGUGCUGAGU

CCAUGGAGCUGACGUCCCUCGCACCCACCCCACUCAUGGGUCUUAAGCAACUGGAAAGGA

AGGGCCAGGGCUCACAUCCAGAGGAGCUCGGCAGACAGACCCAAAAAUAGUGCCACUGCA

GAGAUGCAGCCACCUCCAGCCAACCCCAAGGCCAGGUUGUGUCCCUUCCCAGUAACACCA

GAAGCCCCUCCUUGUCCUGGAUGAGUGGGGGUAUGAACAUUCCCUCUGGAGCAGGGCUUC

UCCGCCAUGGGCGAUUUUACUCCCCCACUAGGGGACGCCAGGCAGUGUCUGUGAACAGUU

UUGAUGGUCACAAUUGGAGUAGGGUUGCUGGAGUCCAGCGUGCAGAGUCCAGGGUUGCUA

AGCAUCCUGCCGUGAUGGGACAGCCCCACAGUAAUAGAAGAGCUGGGCUCCGAGAGGGUC

CUCGGUCAGAUGAGGUCCCGGCCCACCUGAAGCUUGUGUCCCACUGUCGCCACGUCACCA

GGUGACCCCUUUCCCAACAGAGUGCAGGCAGAGGCAGUCAUGGUCAAGGCCCUUGACUGC

UUCAGUGUCCCCUUCUAUAAAAUGGGGUGAUCGCAGGUGCCCCGACUUCAGCGGCUUGUG

UGGAGGUUGGCCCCGACCAACGGCACGUGGGACUGCCCAGCACAGCUCCAGCCCAGCAUA

AAGGCUCAGGGAGUCAGGUGCCACGGUGGGUACUGGCUGGGGAGGGGCGUCUGAAAUGUG

GCCUUCCCACGGGGUUCAGAGCCGUGCGGUGACACCGUCAAGGCCUGGUCUACUCCCUCC

CUGGCUCAAAGGAGAGAAAAGGGCAGAAGGCAAAGUGCCUCCUCUCCCGGCUCCUGCCAA

UAACCUGGCAUCUUCUUUUCAGCCUCCAUGGAGCUGAAUGAGAGUUCUCGCUGGACAGAA

GAAGAAAUGGAAACAGCCAAGAAAGGUGAGGGGUGUGUGGCGGCUUUAAGCCUUGUUGGU

UUGCAUCCAGGGCGCCUGAAGCUCACGUGCUGGGGAGGGUGUUGACACAUUUGGCCUUGA

GCCAGGGGCUGUGGUGAGGCCUCUGGUGAGGUUCUGGUUCUGGGCUGUUCUCAGGUUUGU

CACCCUGGAGCCUCAGUUUUACCUUCCAGAAAAUGGGUCUGUGCAGCUGCGCUGUCAGGC

UUGUCUUUGUGACUUGAGGGCGCAGCUCAGAGAGACACAGCUUGUGCUGUGCAUGUAGUC

AGCGAGUGGCUUCUGGGUGACCUCUGUGGCCAGCACCUCUGGGCCUUGGGGACAGAGCAG

UGGCAGCACACCUGGGUAUCUCGGGACAGUCAGAGCUUGCCGGCGGCCAUCUCGGUAGAG

UCCUGGGGUCAGGGAGACCUGCAGGGCGUUCAUCCAGCCCUUAGAGCCCCAAAGGUCCUA

UCCUGGGCUCAAGUGUGCUGGGGCUUUGGAAACCUGUCUCAGGUUUGAGCACCCCAAUCU

GGGUGACGGGUUCUGGAGUGGAUGGGGACACCCAGGCCGUUGGGGACAGUCGUGGGAGUG

GCUCACACGGCAGGGAGCCAGCAGGCAUUGGUGCUGGCAGCAGAAGCCAUCCCGAGCAGA

UGGCCUUGUGAUGUGCAGCACCGUGGGGGCUGGGGUCCAGUGCCUCCUAAGCCGGAUCCC

CUCUCAGCUGUGCUGUCUCCUGGCCAAGAUCUUUACUUCCCAGCACAGUGACCUGCCUCU

GGGCCUCGGUUUGCCCGUCUGUUGAGUGGGAGGGUCGUCUAGGUUGGCUGUGCGGAAUCA

GAUAACCCUGUGUGAGGGCCCAUGGUGGGCUGAGUGGGAGCGAAUCUCCUCCCUCCCUCG

GCCGUGCCUGGCCUGGGGCAGGGGCUGUCGGAGCUGGCGCAUCACUGGCAGCACUGCUGA

GGCCGGGGCAGCACCUGGCUCCCACGGCACGCUCACUCUCGGUUAUCUAAGAGGUGGGGC

CUGCUCCUGUCCCCGCCCAUGUCCCUGUUUGUGGGCAAGGAGCCAGGAAGCAGAGGCCAA

GGAGUGCCACGGGUUCCGGAGCUGGUGUGUGGGGCUGGGAGCAGGCCCAGGCCCCCACUC

CCCGUCCCUGUCCCGUGCUUGGUCCCGUGGCCCUGCUGGAGUCGAGGCUGUCUGUGACGU

GCGGUUUCUUUGUGCAGAAAGCUCCAGGUCGUGACUUAUGGAGGGAACAAAGGCCCUUUA

UAGCAAACGCUCUUGGCACAUAAGGGCAGGUGUAUCUGAGAACAGCCUGGAUCACGGGGC

UCCUGGGCCCGGCCCCGUCCUCCACUGUAGUUGUGGGGGGUGCUGAGGGUCCCCACAGCC

CUGAGAAGACCCCCACAGCCCCCGCCCUCACCCCGGGACCCUGCCCCUCGCCCUCAGAGC

UCCUCCGGGGGCAGUCUGCCCUCCGCCCCCCAGGCUGAGGUGAACCUGGACCCAGAGAGA

CCCUCGCACCAUUCAGGAAGGAGGGCCUGCCUGCCGCUGCAGGUCAGGCUCUGUGUGUGG

UGAGUGAGAGGCAUGCCAGCGGGGCCUGGGCACUGUAUCCAGCUGGUAGGGCCUGGGCAC

UGUAUCCAGCUGGUAGGGCCUGGGGCUGCUGUCAGCCCCCAGAGAGGCCACAUUGUUGUU

UCUCUGUCUUUAAUGUCCACUUGGUGGUUUCUGGCAUGUAUUUCAUUAUAGGCUGCAAAA

GAAGCUUUAUUGAAAUUGUGUGAUUCCCGCCGCCUUUUAUUUAAUUUUGAGAUGAUACAC

AUGGCACGAAGCUCAGCAUUCUAACCACUGGAAAGUGAACAUCUCAGUGGCAUGAAGUAC

AUCUGCAUUGGUGUGCAACCCUCACCACUGCCUAGUUCCAGAACUUUUUCUCACCCCAGA

UGGAAAUCCCAUACCCAUUAGCAGUCCCUCCCAGGGCCCCGCCCCAGCCCUACCAGCCCC

UGGCAGCCACUAAUGCACUCUCUGUCUCUGUGGAUUGGCCUGUUCUGGGCGUUUCCAAUG

AAUGGAAUCCUACAGUUUGUGGCCUUUUGUGUCUGGCUCCUUUCUGGGAGCAUGAUGGUU

CUGAGGUUCAUCUGAGUAGCUUUGUACGGCUGAAUAAUAUUCCCUCACCUGCACGGACCA

CAUUUUGUCGGUCGUUCAUCUCUUGGGGGAUGUGUGGGUUGUUUCCAUGUGGCUGUUGUG

AAGAGUGCUGCUGUGAGCAUUUGUGUAAAAUUGUGUGAGAUUUUUAAUCACAGGUGAGUC

CCUGGGAUCUCAGCCUGUUGGCUGGGAGAUUUCCCAGAGCACCCACCCAUCGGAGGACUC

CGCGUGGACAUCCCCAAACGGUCAGCCCAGGGUCCCACCGCCUCUCCAUCCCUUCUCGUG

UCUCGUGUUGUCACUACCACCUCCUCACCGGUGACCACCCCCGCCCCCACCGCCCAUUGC

CUGAGAACUGGGAAGACAGUGCCUGCCCUCUGUUUGCCUUCUGGAAGGGCAGAUCCGGAG

GCUGGGAGGACGGCUGGGGAUGACUCACCGGCGGAGAGGAGCCCGUGUCCCACCAUCUCA

AGGUUGGGUCCUCAGUGAUGACAGUGAGCUCAUCCUGGGUCCUGCCCAUGGAGACGGCAG

UGAGCGUCCCCUGGGGGCCGUGCACGCGGAGGCCCUGCUCAGUGACAUUUGUAAUAAUGU

CGUGAGCAGCAGCAUGUUCGUCUGCCGUGAAUUACAGCAAUCUGGACAAUUACUGCUCAG

GGUGGCUGCUAAUGGCUGUGUGUUCCAGGCGAUGGCAGGAGGGAGCGGGGGUGGGGCGGG

GAGACAGGUGCUGGCAGGCCCCAGCAACUCCUCCAUAGGGGGUCGGGGUGGAGAAGCUAG

GAACGUUCUGGAGCCCCCGCCCAGAAAACCAAGAGUUAGGAAAAGGGACUGGAGAAGCGG

CUAUUGCUGAGGCAGAUGGUCUCUGCCCAUCCUUUACCCAUCAUUUCAGCAACAUAGUGC

CUCACUCUCUGUGUGGCAGGGGCACUCAGGAAUGAGCCAGCCCCACCCCUCCCUCGGGGA

GCCUGGGCAGGUAAGCAGCCAGGUUUCCCCAGGGCCUUCGGACCCCAGCCUGCCUGAAGC

CUCUGGAAGACUUUAGUAGCAUGGCCCUGGAGGAUGAGCAGCAGAGGGGCCCCAUUCCUA

GGGAGAAACCAGGUCUCAACCCCCUGGAUGUUUCAGCUGUGUGACCUCAGGCAAGUCAGC

CAACCUCUCUGUGCCUCAGUUUCUUCAUCUGUACAGUGGGAUUCAAGGAGCGCCCACUGU

AUGUUUCCUGUGGCUGCCAUAAUAAAGGGUCAUGAACUGGGUGGCUUAAAAUAAAUGUAU

UCUCUCACUGUUCUGGAGGCCAGAAGUCCAAGUUGAAGGCAUCCAAGAGGCUGCAUUCCC

UCCAGAGGCUCUGGGGAGGAGCCUGCCUUGCCUCCCCCAGCCCCUGGCGCCUGCCUGCAG

CCUUGGCAGUCCCUGGCUCAUGACCACAUCCCUCCAGGCUCUGCCUUUGUCUUUGUGUGC

CUUUCUCCCUGCGUGUCUCUGUCCACAUUUUCCUCUUCUUACAAGGACCCACCCCAAUCC

AGUAUGACCCCACGUUGACUUGAUCACAUCUGCAAACAUCCUUUUUCCAAAUAAGGUCAC

AUUCAUAGGUUCUGGUGGACACAAAAUGUUGGGGGGAUGCUCUCCAACCCAGCACACCCA

CCUUCCUGGGGUUUUGGUGAAAGUACACAGAAGAGCUGAGGGCAGUGCUGCCCGGGGCAU

ACCGACCUCCCCAUCACUUUGUAGCUACAAGCAGCUCCUUAGCCCCCUGACCUUGGCCUG

GCCCAUCUGUUUCACCCUCAGUUACCAUCGUUCUCUCAGAUGAGGCAGGGAUGCUACCUC

CCAGGGGCACUUUAAAGACGGAGCGAGCCACAUAGGAACAGUCCUUGGUAUACAGUAGGC

ACUCAGAAAUGGCAGGCCGCUAGCUUAGGGCGCAGUCUCCAGACCCUGCUCCCUAGGUUU

GGGUCCCAGCUGUGCCUCCUGCAGAGUCUGACCCUGAGCAGGUACCUACACUUUGGGGCC

UCGGCCCCCGCCACCUCUGUCUUCGCCCCGGCGAUGGGGGUCGGAGGUAAGUCACCUGCC

AAGCAAAUGUACAGUGACAACUGGAGGAACUUCCAUGGUGCUGGGGGCCACCAGUCAGAG

GGCUACCCCGAGGAAGGGGCCGACACCCAGAGCACGAAAUAAUAGUGAAUAUCACCCAGU

AACACCGUGGAGCCCAUCCUGCGUGCUGGGCACUGCUGGAAGCUUUGUGUGUAUGAAGUC

ACUUUAUCUCUACCAACCUGACUAGGAGGCAGGUGCUGUUAAUACUUCUGUUAUUCAAAU

GAGGAAACCGAGGCCUAGAGAGGCUUAGUGAGCAGCCUAGGGUCACACAGCUGUGAAGUG

GUAGAGCUGGGAUUUGAACCCAGGCACUCUACUUGCAGAGUAGGAAUGGGGUUGGCAUGA

GAGCUGAACAGGUUAGUCCAUGGGCCAACCUGUUGACCAGGUAACAAAGGCAGGACAGGC

AUGUGCAAAGGCCCCAUGGCAGGGGCAGUGUGGCCCCAUGAGAAGUGGUUGAAUCCCAGA

AGGUAGAGGGGCCACCGGGAGCCUGGCCUUGCCAGCAUGGGAUGGAGUGUAGCUAUUACU

CUAGAGCGUGGGAGGCCACUGGGGAGGGGCUGGGGACAUGAUGGCCAUAGGGUUUCUGGU

GGGCAAGUGAGCAACCUCCCUUCUUGUGUGCAUGCGGCUGUGGCUGCCUGCCCUGGGGCU

UGUGCUGCCUGAGGACCGAGAAUGUUCAGGAGGCAGCUUCCCCAGGAACCGGGCAGCUCC

GGGGAGCUGGCCGCCUGCCCUCGGGCCCAGCGCCAAGUGGGGAAGGAGGGGCAGGGCAGG

AGCAGGGCUGGGAUGAGGGACUCGGUUCCCCUGGGAAAGGUCAGCCUGGCCUGGCUUGGA

AGCCUGACCCAGCUGUGCGAGAGGCCCGAGGCUCUGCUGGCAGCUCCUCCAGGCCCCAGC

CCUCUUCUCUGGCGGGCAGAGAACCUGCUGUGGAGGGGGUUGGGAGGCUCCCGGUCACAC

CAUUGUCACACCAUUCUAGACCGGGAGUUGGCUCUGGCCCGCCUCCGGGCUGCCAGUCCU

UCCCAAUCUCCAGGGAGCGGCCAACAGUGGGAGGAUGUGCAAGACCAGGCCCUGGUGGCU

CCCAGUAAGCCCGGGCAGACCCCAGAGGCCUCCCCCAGGACUCCAGCUGCCUUGUCUAGU

GGCCUUGCCACUUCUCACAGGAGCGAGGCAGGAACCACCCAUUGCUGUGCACAUGGGCUG

GCCUGACGGGCUUUUAUAGAUGAAGCGUCUAAGGCAUGGGAAGGGGAGGGGUGAGCCCCC

AGCCUGGAGCCUGGAGAGCAGGACCUCCUCCACUACAUGUCCACUGGGAAAGCCUUGGGC

UGUGCCCACCAUGGGCCCCCCAAAAGAGGAGCGCCUGUGUGCUCUGCCGCACAAAGCUGC

CAGAGCUUACAGUGGGGUGGGCGGGCCGGUAGGCAGUGCUGCUGCUCCUGGGUUUGGGUC

UGCCCGGCUACUUCCUGGUGCAGCUGUCCGUCAGGGCCCAGCAGGAGGCGACACUCGAGC

UGUGUGACUCAGGAGGGGUGGUAUACCAGGCAGGGACGGUGCGGUGCCUCGGGGCUCGCG

UCCAAGGAGACUGUCACCACGUGUGGUCUGGAGAGAGACCACUAGGGAGGGUGCAUGUCU

GGGGCUGGUUGGCCUCCCGGCCGGAUAUGUGAGAAACAGUCUGUUGUGAUGGUUUGGAGG

GAGGAAAAAAUUGGGGUUUCUGCAGGAACUUGGGGUACAGCCAGACACAGGGGCAGGGUC

UGGACCACACUCUUGCUCCUGUCAGCACCCUUGAAGAUGAAAUCAGGCAUCAACAAGCCC

CCCUUCCCUCCUAGGGGAGACCCAUGAAUCCGCAUCCACAUGGCUCCUGACUCAGGGAAG

GAGCACCUGGCGGUCGAUGCGGUAGUAAUGAUGCCUGUCGUCACGUGGCGUGGCUGGUGC

UCACGAACAAGGCAGGCCCUCCACACGCGUGUGUACACACGCACGCAUACACACGACUUC

UGCUUUGCACAGGAGAAGACCAAGGCCAGAGGGAGAGCCAGUGCUCACCCAGCCCACUGC

GGUGGACACAGGGCAUGGGGUGGUCUGUCUGACCCCCCAGGCUGGCUUCCAACUUUGCUC

UGCUCUGUAGGAAAACGUGACAUUGAAAGGUGUAACGUGGCACUUCCGGAAAUGGUAGGC

GAGACAGCGCCAGGUCGUAUGCAGGUGACUUUAUUUCAUCUUAAUGAGUCUGGGUUUUUU

UGAGAGCUGGGAGAAGACGUAGCUUUUUCACAUCGUACCUAUGAUUUACGGGUAUAAUUG

CUGAUGCGGAGGCUAAGGCAGAUAGCGAGGCUGAUUUUUGAAAGAGUAGAUGGAGAGAAA

AGAGUGGGGAGGUGCAAGUCCAGGUGUGAUGUUAUCCAGCCAACAUCCUGAUAGGAUUUC

AGGAUGGCAGGAGUUCGGGAAGUCCUGACAAGACACGCAGGAGUGCCCGGCCUGUCAAGG

GUGUCAAUGCGGAGGCACCGUCCCUGUGGAACCCCCCCCCCGCCAGGGCUUGGGGUUUCU

GAAAUGAGGAAGUGGCAUGACUCAGGCCCAAGACGGCUGGGUUCUUUUCCUCCCAACUUU

GAUAGGGUUUUAGGCAGAGCAUGCUGAGUUUCUCGGGGAAAAUGGCAGGUGGGGGGCGGU

GGUCGAUGCACCCCUCUGGCAGGCAGCCUGUGAGUCUUCAUGGAAGGGGUGGGAAACGUC

AAGGCUGGAGGUGACUGGCUGGGGUGGGGAGAGCGAGUGUGUUUCCAGGGUUGGGGGCUG

ACUUCCAGAGAAAACAGCUGCAACAUCCCCAGAUGCAGGUGGGCGGUGGUGUGGUCCCCU

CGGAGUCCUAGCUGUGGAUGCAGGUUUGCAGACCCUUGGAGAAAAGCAGCUUGGAGGCAU

CUGAGCGGCAGCACAGGCCUCCCUGAGGGCCAGAGCACCUGAGCCGAGGAGGCUGAGUCC

UGGCCCCCACAAGAACAGCUCGUCAUGGGCCCGCGGAUGGCAUGUGGGACGCUGCCCCAU

GGGUGCCCAGAGGAGGAUUAGCCCAGCGGGGCCCCAAGCACCCACUCCCGUGGCCGCCGG

GCCCCUGCGCCUGGCUCUCCACUCGGCGUCUGUGACUCAGACCUCCUGGCUGGACUUCCU

GUCUUGGUCAAUUUCACUUCCUAGUUCCUGCCUCCUGACUCUUCAGAACGAAACUCCUUC

CUUAGAAAUUAGAAGUGGGGUCGGGGAAUCCAGACCACAGGGCAUGGUGGCAGAAUCUGC

CAUCUCCGCCCUGGCACAGAGUGUUGGGGGCGUGUGCCUGGAUGUCAAGGGGGAUACCCC

AGCCUCUUUUGGGAGGGUUCGGGGCCCCCUGCAGAGCUAAACGCACAGCCCCCUCUUCCU

UUGCAAGCUCCUUACCAGGGACCCAGACGGGUGGCAGAGAUGCCCAGGGUGCUCAGCAGG

CCCCGGACACCUGCCGAUGAGCAGUGGGACGAGGGAGCUAAUUUCAGAGGCGGUGAAAGC

UAUGAAGAUGAUCAAACCCUGGGAUGGCAGGGCGACGGGGGGGGGUCACUUUGGAGGGGC

GGGCAAGGAAGGCUUCUCUGAGGAGCCAUCACUUGAGCCAAGACCAGAGGGAAGACAAGG

CACAUCUGUGAGAAAACCCGUGGAGGCAGCAGGAGCAGCUGGUGCAAAGGCCCUGGGGCA

CACACGAGUGACCCAGAGAGAGGCUGGGCGGGUUCAUGGAGCUGGCUCCUAAGGGUCACC

UGGGCCGUGCUAGGGCCUUUGGAUUUUAUUCCAAGAGCUUCAUGUGGUCACUGGAAGAUU

UUAAGCAGAGGAGUGAUUUCACGGGGUUGGCAUCCUUAAAAGUGCACUGAGACCUCCCCU

GCAGAGGGCAGAGGGCAGGACUCAGGUGGAGAGGCCCAGAUCCCUGAUGUGGUUGAGGUU

UGUUGCCCUCCCCUGGCAUCGUGCCCCAGGACGAGGGCUGGGGGUGCAGGGACAUUCCAG

AGACGCCUUCUGCUCCCCAGAUUUUUAGGGGGCAGGAUGGAAGGGCUGGGAGUUAAAGGG

AAAAGCAGGUUAAUGAUCCCAGAGUCAGGGAAGUCGGUCGUGACCUGAAUCUAGGAUCCA

AAGUUCAGCUCAGUUAACUUUCAACCUUCUGGGUGGCUCCCCGGCAUUUCCCACAGUGUU

CCUUCACGGCUGCAGGGGCUCUCGGAGGAGGACGAAAGGGGCAGCUGUGGUCCCGAGACC

CCCCCAGCAGCAGAGGGCUCUUUGGGGACCCCAAAUCCCCAAACCCUUCCCCCUCCCCUA

AAUGGUUGGCGGUCUCAUGUGCACCCUGCAAAGGCUCCUCAAAUCCCGGGAUCCCUCCGG

AAUGCGAGGCUAGCCCUGCCCACCCACAGUCCCCGACAGAGGGCUCGGUCAGCCUCGGCA

GCAUCAGGCCAGGCAGGGGGCAGAGCUCAGGUGGAACUGACACGGAGGGUGGGGGGACGG

GCGGCGGGGGAUGAUGGCGGAAGAAGCUGUUUGCUCCCUGUCUGCCGGGGGUGGAGAAGC

UCUGUUUUUCUGGAACCCUCAUCUGUUCUUACCACUGCUGACGCCGCCACCGCCGCGGAU

GGGGAGAGAGGGAGGGGGAGCCAGUGCCAAGUUGGCCCGCCCCUUGUGAACCUGGGCCUC

UGCCACGCUCCCCCGCCCGCCUGCCCGCCCGCCGCGAGCCUCCGUCUGGAGGUCACAUUC

AGUCCUGCCGCGUCUCGCUGGGUCAGGCAGCAAGAAGAUCAGAGCUGAGCCCGGCUGCGG

GGGACACGGCUGCCCUGCUGGGCCUGGGUCCUUGGAUUUUGCCCGGCCCGGGGGUCUGUG

GGCUCUGGCCGGCAUUGCUCGCUGAGCGUCCGCACUGGGCACAUUGGUGCUGGAGGAGCU

CGGCACGCUGCGCCCCGGUGAUACCUGGCCCAGGGGCAUCCCACACGCACCACUGACAAC

UUCCCGCCACCCACCACCCUCGGGGAAGGGCACCUCGCGCGCCCACAGCGGCUUGGGGAC

GGCGUCAGAGGUGGCAGCGGCCGCUGGAGGAGCCCCGGGCCACCACAGGCAGGCGGACUU

GUGAGGAGCUGCCUCCCGCCCGCCCCUCUCGAGCCAGCCUCCGCCCGCCGCGUCCCUCCC

UCCCUCGCUCCCUCCCUCCCUCCCUCCCUCCCUCCCUCACCGUGGGACGGAGAAGGAUGU

GAGGCUGAGCUGAGCCGCCAGCAGACGCCAACCAGCAGCCUCGCAGAGCCGACCGCGGCC

GCCCAGCCUGCCGGCCCACUUCGGCCCGCUCCCUGGGGCAUCUUCCCAGCCCUAUGUUUC

CGGAAAACUUGGCCGAGGGGGAGACGCGAAUGAGAGGAUGUGAGUGAGUGGUCCUGGGGA

GGGGAUGGGGGACUGCGGGUGCAGGGACAGGGACCGCAACGUGGCCAUCUGGGAUUUGAG

GCUGGGCAGCUGGGGAGUCGGAGUUCUGGGCCCCUGGAGUGGAUGGGGGUCCCAGCCCAU

CCUUUCCCCCAUGCCCUCUCCUCUCUCUCCCUGCUGGGCUGAAGCUCUCCUGGAAGCAGG

GGUGGGACCUGGAUCUGUGCCUGGGAAGGUCUGUGAUGCUGUGUGCACAGGGGGUCUCUC

CAAGUGGACGGGGGCUUCAAGCUGGGUCUGGCGGGGUGGUCUCCAAGGCACGGUAGGGUA

GAAAGUCUUUCUCUCCGUGCCUGGCAGCCUCUGCCUGGGCUUCCCAGAACAGGAACCAGA

GGAAGCCAGUGCCAGCCUGCCCCUACCCCGCAGCCCUGGCCGGCACUAAUGGGACACCCA

CUCUGGCUCCUCACACGGCAGUUAAGGAAACUGAGGCCGGAGAGCAGAAGGGCUGGCCAA

GACUGCUAGCGAGCUCUGGCCAGGCGCCUGGAUGACUCUGCCCAGCUCCUUUGGGAGCCG

UAUGUUUUCAGGAAGGGCUGGGUGACCCCCACCCCCACCUCCAUCCCCUCUCCUCUGCCC

UCAACCCACUCCCACUGCCCCCAUGAUCGGAGUUAAUUAGGGUCCCCAGCCCCCAGCCAG

GCUCCUAGCUACCUCCUUAUUUAUUGACUGAGUUUGGAAAUGUCAACCUCCCCAGUGUUG

UUGGGCCACCCUCACCCCACCCCCUGAGCUCUCCAUGCCCACCCCACCCAGCAGGCCCAG

AUGGGAGACCCGCCCCCUCUCGCCCUCUCCCACCCGGGUGCCGGGUCUGUGAUCCCUCUG

GCCCGGUGUGUUUGCCUGGGCUCUCUUGGCCCCGGCUGGGGAAAUAUAAAUAAACGCGCG

GCCCUGUCCGCCAGGUUCUGGAAGUGGCAGCUGCUGCUGGCGCGGGCGGAGGGAAAGGUC

AGGGGCCGGGUGCCACUUGAUCCCACGCCCCGGCCCUCCCCUUCUGCGCCUGUCCUUGAG

GAACCCUGAGGAGCGGGCUGUGAGGCCACGUGGCUCCCAGGGCCACGGCAGGCGAACAGG

UGGCCUGUCAGUCAGUGCCACCCCCACGCCACGGGGGCCAGAGGACCCGGAGGGCAGGAA

GUCUGGGGCCCCACGCCCGCGUUGCCCCCCUUGGCUGGUCCUCUAAGCUUGGGUGAGUUG

GCGGGACCCUCUGAACCGAGAUUUCCCCCUCCUGGGGAUGGGAAAAUGGUCACAGCCAUG

ACAGCAAGGAUGAGACGGAACCAAGUCUUUGAAGGGCUUUGCAAACUCUGUGUGUCAGUU

AGCCGUGCCACGGGCUGAUGUGUUGAUGUUUCUGCUGCUGUUGUUUUAUUUGCAUUAGAG

CCUUGUGUGUAGCAGGCAGGCAGCCAGCACGUGGGUGGGUGGGUGGGUGGAUGAAUGAAU

GAAUGAAUGAAUGAAUGAAUGAAUGAAAAUACCAACACAGACGGCUGCCCCUCAGCUCCC

CAACCUCUGAACAAUUUUAGCUGGAGCUUGGCUUGCAGCUUGAUUUUAUUCAAGGAAAAU

GGUUAAAACCUUCAUUAUGGGAAAUCCUUCAUUAAAGUCCUUACAAGUGGAGCUCCAGCU

CAGAGAGGCUGCCAUGUCCCGAGCUACGUGAUUCAGCCUUGGGGCCUGCUGCCCUGGAGG

GGCACCCUCUUGCCGCACAGACCCCUAGGUCAGGAGCACUCCCUGUCUCUCCCACAGCCC

UGGGAAAGUGGGCAGGCCCUACCUGCAUGGACUGGCUGGGCCACACAAGGUCACGCCGCU

GGAGACACAGGCAUAAAUAUAGAGCUGCCUGGCCUGUGCCUGUGUGUGCCGCAGCUCGCC

GGGCAGACAGGCCGAGACCGGGGGCUUGGCUUCCCAGGCACUGCCUCCGGUGCAGCAGGG

GCUGUGUUUCUGCCGAGCCUGGUGUUUGUGGCCUUGAAACUAGAACCUGGGGGGCGGCAU

GCUCUGGUUCUGGCCUGAGACCUAGUGAUGGAGGGAGCUUUUUCUGCCGAUUCCUGGAGG

GUCUCUUCCCACAGCAGGAGGUCUGUGAGGCUGAGAGAACCUCCUGGUUGUGCGGGUUGG

GGUUUGGGAGCAAAUUCCAGCUGCGCAACCAAGCCUGGUGGGCCCCGAGUCCUGAGUGGG

CCUUUAGGUGCCCUUAGGGAGUUUGGGCCCUGGAGGUGCCCCAGUUAACCUGGGCCUGGA

GGCUCCUGGCCCCAGCUCAUCCCCACCCUGACUUCCACCCUCCCAUCCCCUGCUCACCAC

GUCCCUUCCUUUCGCUGGGCCGGUGUGGCCGGAAAACAACCCGUCUCCCGAGGCCUCGCC

UGCAGCUGUGAGCGAGUGUCAGGGCUGGGUCACGUAGGCCAGGCCCUCCGCCCAGCAGGA

GAGGUGGGGCCUGGGCAGAGGCUGUGCGUUCUGGAUAUUCACAGCCCUCCGCCCCCAGAC

UCCCGCGCAGUGGCCUCGGUGGGACUUCACCUGUCGCUUUUGGCUCCUCCAGCCCAGGCC

UUUCCCAUGCAUCCUGGCCUGGAGUCAAGUGACUGAGAAACCAGACCCCAGAGGCCAGGA

CUGCCACCCUCUAGUACCUUCCCCGCUAGGGGACACGUGGCCCAUCACUGUCUCUUCUUC

CAGAGGUCCUGGGCCUGAGCAGGGAGCCCCAGGCUGGGUUUCUGCUCCAGGCCCGUUUGC

CGCCCAGGCCAUGGAUGCAGGCCCCCUCUGCGUGCCGGGCCUGGCCUCUUCUCACUGCCC

UCCUCUCCUGAGACAGGUCUCCUGGAACACGGCCGCAACUGGUCGGCCAUCGCCCGGAUG

GUGGGCUCCAAGACUGUGUCGCAGUGUAAGAACUUCUACUUCAACUACAAGAAGAGGCAG

AACCUCGAUGAGAUCUUGCAGCAGCACAAGCUGAAGAUGGUGAGCCCCCAGUCACGCUGC

ACCGCGCCUCUGGGAAGCUGGGAGAGGAGCCCAGACUGCCUCAGGAGAUGUCUCUGCACC

CCAGGAGGCAUGGGCCAGGGAGGGGCUAUUAUGCCGCCUUGUUAGAACUCAGCUUCUGGG

GGUUCAAGCCCGGGAGUACCCCGAGAUGGCUGCAGGCGGCAAUGCUGCUAUGAGUGUCCC

UGCCUUGGAGACGGGCCGGGCCAGGCUGCCCUCCACCCCCACCCUGCACCCCACUCCUCU

CAGACAGACACCUCUGGGGGCCCUCAGCCCUGUCUCACGGUGUGGGGGAAGGAGCGCGGU

GGACCAGACUGACUCAGCCAGGAAUUCGGGCUGUCAGCGCGUUGGAAUGAGGACCCGGAG

GCAAAUGGGUCCUGAGCGAGUGGCUCCCCUGCCCCAUUUGACCUCACAGCUUUAGGGCUG

CCGUGGUUGGGGAGGGACAGGUCUGGGGGGGCGGGGGCGGACGUCUCAGCCCCAGCAUCC

UGGAGGGCUUGAGGCAGGCCACGGGAGGCUGAGACCCUUCCCUCCUCCACAAGCACAGAA

GGUGCAGGCUCGCCUCGCCAGCGUGUAAAUGGCCUCUGAGGAGCAGCCGCUUCUUCCACA

GCUGUUUCUGGGAAUUGAGGAGCAUUUUGGGGAGACCUCUGCUGCCUUCCCUCUCUCCUU

CCCUUCCUCCUCCUUCCCUUCCUCCCUCCUUUCUCCCUCUUUGACACACUUGCCAAGCAU

UGUCCCCUUUUUGGAGCUGGGGAAACUGAGGCUUAGCCAGGCUCAUGUGCGACUGAAUUG

CGAUAUGCACCCACAGUUAUGAGAUCAGCACCCACGCUCUUCAUCGCUAGGCUGCGUGUU

UCCUCAUCGAUACAGAAGCACUGAGGGAGGGGUUGCAUAGCUCCCCUCCCCCAGGACUAC

UCCUCCCCCCGCCACUCCCAAACCAUGACAUCGGGCUGCCUUGUGCCUGGGUGUAAAGUG

UGUGCAGUGGGUUCCGGGCAGGCCAUGCUGAAUUGCUCCCUCAGUGCAACCUGCUGAGGC

CCAGAGAGGGUGAGCAGGCUGUGAGGUCACACAGCCUGCAGUGGGAGGACCAGGUAUAGC

CUCCCCCUGUUUGAACUCCGCCCAAGCACUGUGCAGAAUUUAAGGACAUCCUUCCACCCU

CCCGAGUGGGUUCUUAGUGAGUCAUGUGUGCGCGCGGCCGCUGGUGGCUCCGGGACCGAG

GGGCCACGGUCCCACGGCCCUGUGGUCAGGGCUGAGAUCCACCCUCUGGCCUCAGGGAGG

CCCCUCCCCAUCACUUCCAGUUAAGCAGUGCCCCGCGUGCCCUCCACAUUGGCCCCUGGG

AUUUGUCCCCAGAGCUCAGGGCUUCAGGCAGCGCCUUUUCCUUCUGGAUGCUUCGGGCCU

UCUGGUUGGGUGUGGAGGAAGGCACCGCAUCUGGCUGAAUGUGCAGAACCCCGUCCCUAA

CACUUGCUGAAGUCCCUUGUGGCAAAGAGGGCCUGAGCCAGAGCAGCCUGGGGGCGCAGA

AGGGGGCCUGGGCCGGGGGAUGUUGUGGGCCAUUGAUUCUGUCAGCCAGGUCAGGAGUCU

GAGGUGGUUCAUCCAGGAUGACUUGGGGGCAGGGCUUUGGCCCUGAGCAUCUCCCCAGGA

ACAAAAAGGGCCUCCUGCUUUCUCCCCAGCUGUCCUUUCACUUUCUCUCUCCUCCCCCCG

CCUCCCCUCUCUGGGGCUCCCUCCGCCUGUCCCCCUCACCCCACGACCAUGGACGUCUCC

CCCCGCUGCAGCCUGCUGCCACACAGCCUGGCUUUCCAGGCUUCCGCAGGGUGUGGCCCC

ACUGUACCCCUAGAGACUCCAAAACCUGAGAGUGGCUUGGAGGACCAGAGACCCCAGCAC

GGUGAACCAUCCCAUUAGGCCCAGGGCGAGACUGGGGUCAAGGCCCAGCUUGUGGAUUUG

GGCACCCCUGUGACCUUGGGCAGUGAAUUCAGCCUUCUAAGCCUCAGUUUCCCCAUCUGU

AAGGUAGGAGAAUAACAGUUGUUAUCCAAGAGGUCCUAUGAGGAGUGAACGAGUGAUUAC

CUGUGAGGUCCUUAGUGCCUGCCAGAGGGGCAGCGCUCCGGGAGGGCGACGUGCCCUAGU

UACUCUUACUGCUGUGGUUAUUGUUAUUUUAUCUGGAUGCCAUCUACCCCCUUCUCAGGG

CCACCUGAGGCCCCCUCUUUUUUUUUUUUUGAGUUGGAAGGUUUUAUUAGACUAGGAGAU

UUGUGGGAGGUAUGAGCACUGGGCCAAGGUGGCACGGGCCAUGCUUAAGGCACCCUGUUG

AGUGUGGUGACCACCAGUGAAGCCGCUGGCGUGGACGAACAUGCAGCCAGAGAUCCCACU

GACGAGGGCGCCUCUUGAACGGUGCGUGCAGGCACAGCCACAGUGUGCUAUGGGUUGGGA

GCAGGGACUGGGCCCAGGAGAGCUGAGUUUGAACGUAGGCUCUGCCACCUACUAGCUGUG

UGGCCUUGGGGAAGUGACCUGUGCUCUCUGUGCCUCAGUUUCCCCAUCUGUGAAAUGGGG

ACAAUAAUGGAACCCACUUCAAAGGGCUGUUAUGGGAAUACACUGGGUGGCUGCCUGCAG

AGUGCUGGGAAUGCGGCCUAGUAUGCCCCAGACACUCUGCUAGUGAGAGCUGUGAUGGUG

CUGGUGGUGGCACACUCACGAUUGAUUGAUUCAUUCAUUCAUUCAUCCAUUCAUUCAAAG

AGGCAUCCCAGAAGGAUGGUGCAGAGCAGGGCUUUGGAGCUGGUGGCUUUGUACCCCAGC

UCUGCCGCUUAGUGACUGGGUCCAAGGAUACUGGGCUUCACCUCUCCGUGCUUCAGUUUC

CACCUCUGUAAAAUGGGGAUAAGAAUGGGACCCAGUCGUAGGGCUGUUGAAGAACAAGUG

UGUUUCUUUUUGUAAAGGGUGGUGCCUGGUCCUUGGUCAGCCCUCAAUAAGCAUUCAGUA

UUAUCCUCACUGUCCAUUCAGCAGCCCCAUAUUCAGCGCCUGCUGUAUCCCAGGGAAGCA

AUGGCAGCUGCCCUGAGGGCAGAGACAUGGAUGGGGUUUUCCUGCUGGGCUGUGGAGCUA

ACACUUCGUUAGUGGACCGUGGAGGGAGUGGGGGCUGCAUUGAAGGAUUCUGAGCUGAGA

CACAGCCCCCAUCCAGGGCUGGACUCAAAGUGGGUCGUCCUAGUGUCGAUGAAAGUGAUU

CUGAUCCGAAUGACACAGUCAAAGCUGAUAAAGAAGGUGGCCCUUCUGUCCCCUGACGCU

GAAGACUCUGGAGGCGUGGACAGGAGGGGACCCCGAGAUCCAGGUUGGGACCUGGAUGAG

AAUGAGUGAUUUGGGAGUGUGGGGUUGGUUCUCGCUCCUGGCCGACCUUGAAGGAGCACC

CUCAUAUCGCGGGCUGCAGAGAAAGCGGAGCAGAUGCAGGGACCCACUACAGGAAGUGGG

GCUCAGGCCAGGCCAUCCGUCUUGACAGUAGCCGUUCUUGGUGUAGUGGUGAGCUCCCCA

UCGCUGCAGUAAGCAAGCAGGUCUCACCAGGAGAGGUGCCGCUGAACCUGGUGACCUCCA

CCUCCUGCCCCUCCCCUGUGCCUUUCAGUCCCAGGGAGGAGGGCAUAUCCCAGUGAAUCC

UAACGCAGUAUCAGCCACCUCUGGCUGCUGGCCCAGCCCUGUCUGGAGUACAGGAAGACA

UGUCACCCUUGGCCAGAGAAGGAAGGAGUGGGGAAGCAGAGGGUCAGUGGGUGUCCCCUC

CCGCCUUGCACUAGGUCUGGCCCGAGUGUGGCCGCAGGCGACGUCAGCAGGAAGCAGGAU

CCGGCGGCCGGGCGGGCGGGGCAUGUCCCGGAGUUAUGUAACACUUGGCAUCAGUGUCUU

CUACGAGGUUUCCCUCCCCCUCCUUAUGGCCGAAGGUUACCCUUUUCUCCCAUCUGUGAA

UUCAGGCCCCUGGGGCUCGGCUUCCACGUUCCUCAGUCUCUGUCCGCCUCAGCCUUGGCC

CAGCCCCGCCUUGUUUUCUGCUGUUAAUCUCUUCUACCUGAGGCUGCACAGGCCUGCAGA

GAGAUGGUCCAGCCACGAAAUGUUUGAAGCUGGCCUGCAUUCAAAUCUGAGCUCCUCAAC

UCAUGAGCUGUGUGGUCUUGGGCAAGUGUCUUUGCCUCUCUGAGCCAUUGUCACUGUCUA

UCAGGUUCAUUCAUCUAAAGACAUAGUUACAGAGCGUCUGUUCUGUGCUAGACACUGUUC

CAGGUAUGGUGGGAAUACAGCAGAGAACAAAGAUCCCAGCCCUUGAGGAGCUGGUGCGAG

AGACAGGUUCACACACUCAAUUUCAGAAUGGAGUCUGAAGAUGCUAUGAGAAGAAUGUAG

UAGAAAGUGAUUGAUGGUGGGGGUGGGAGGGAUGAGCUAAUGAUGGAGUGAUCAGGGAAG

ACCUCUCUGAUGUCUGAGCUAAGGCCCGAAGGAGGUGAGGAAGUGAGUUAUGCAGAUAUC

UAGGGGAAGGGUGUCCAGGCAGGGGAACAGCCAGUGUAGAGGCCCUGUGGCUGGGGCAGC

GAGCAGGGAGGGCAGUGUGUUUGUAGCACAGUCUCUGAGGAGGGGAGAGGGGAGAGCAGC

UUGGAGAGGCAGGCAGGGUCCAGGGCUGGAUCACACAGGGCCAUAUGGAUAGGGGGAGGA

CUUGGGGAUUGUAUCCAGGUGUUGGAGAGUUUGAAGCAGGGGGUGGCAUGCGCUGAUUUG

CAGAGCAGCUGUGCAGGUCCAGUAAAUUAAUACCAGAAACCGCUUGGCGCAUAGUAGGUG

CUUAAUAAACGUCAGGCAUAAUGAUUAAUGCCAUCACUAGCAUCUCCUAUUCUACACACA

CCUCCUGAUCCCACAGUGCCGGACCAUGUGUGUAAGGAUUAACUGUAACUCCUGGGACUC

UCUCUGAGACCCAGCUUUGUGGGGAUUCAGGGUGUUGGCACCAUCCCCGUCCACUGCCUC

AGAGACCCCACGCCGCCCCCCGCAGAGACACCUUGCACUCCAGGAUGCUGGGCGCCACCA

CCAGGCCGACAGGAGACCCCUGUUCCCUGCGAGACUCUGAGCAUCUCCCAUGUCCCAGCC

ACCGCCCGGGCAGCUCAGGGCACCCAGCCCUGAGUCUUUCCUGAGCCUCUGUCUGCCAAA

AAUAGAUUCGGGAAGCAGGGGGAGCCCUCGGCCGCAGCCGCAGAACCCACAUGCCACCAC

UUUCUCAGGCCGCCACAGGCUCGAACUCUCCUGGGAGAGGCUCAGGAUUCGGGGCAGGGC

UGGGACUGGGCCCAGGACGUGUGCGGGGUUGCAGAGCUGGGCAUCCCUGUGGGCCCUGUG

GGCACUGGCAGGGGCAGGGCUGUGGCCCUCUGGCCGACUGUUGGUGUCGGAAACUGUGAC

UAACGGACGAGCAGUCGUUUCCCUUUUCCUCCCUGCGUUCCAAGGGGCUGUGCUCAGAGG

AACCCUGUUUGCUCAGGAAGAAGGCAGGACAGGAAGUGAUCCCACUCCCUGCCUGUGGCU

GGGGCUUGGAGCCCGAGACCCCCUCUCCUCCCCUCCAUGAGGCCCAGCUCCAGCCUGGCC

ACGGUGAAGGGCUGGGGUGGGCGGGGCCCACCUGGCAUUUGCCCGAGAGCAGGCUGUGUG

GUCUGGACGUCAGCAGCUUUUUCUUGGAAUCCGGGUGCACCAGCGCAGAGGGAUGUGUGA

UUCCCCCUGGCUGCCGCAAGGAAGUACCACAGACCGGUGGCUUCAAACAACAGAGAUGUA

UUCUCUUCCAAUUCUGGAGGCUAGACGUCUGGAACCAAAGCGUCCACAGGGCUGUGCUCA

CAUUCAAGGCUGCAGGGGAGCCUCUUCCGUCUUCCAGCGACUGCUGGCAGUUCGUGACAU

UCUUUGGCUUGUAGCUGCAUCUCUCCACAUGUGCCUCCACUGCCACAGGGCCUUCGUGUC

CCUGAAUCCGUGUUUCCCUUUUCUUCCAAGGCUGUCAGUCCUGUUGGAUUUAGGGCCCAC

CCCACUCCAGUGUGGCAUCAUCUUAACUAAUGACAUAUGCAGGAUCCCGUUUCUAAAUAA

GGUCUGAGGUUCCGGGUUGAUGUGAACUUUGAGGGACACCAUUCAACCCAGUACAGGGCU

CUUGGCUGAGAGCUGCGGGCACCUCCAGGUGUGGCGGAGGGCCCACCUCUUUCUCAGCAA

CCCCAGAGGGAUCAGGCUGCCGCCCCCCCGUCACACCAGGCUGUGGUGGGCACCCCCCAG

UGUCUUUCCAGUCCCCCUGGGAGUCCCCAGUCACUUCGAGAUGCUGCCUCCACGAUGCCC

CUGCUCUCUGCUGUCUAUGCGUUCAAGCCCACGAAGGCUGGCCUUUCACAGUUGCUCAGC

AAGUCAGUCAACAAACGCUUUCUGAGCCUCUGCCUGGCUCAGCGUGGGGACAAAGAGACA

CCCACCCCAGUGAGCUACCAAGACGAGUGGUUUGAUGAUGGUGCCUAAGCUGGGGGAAGA

AGAGUGGGCUCUGUGUGUGAGGAGCUGGGGUUCCCAGGAGCAGGGUGCGGUGCGGGGAAA

GUGGAGGCCCGACGAUGAGGGCUUCUUUCCAUUCCUGGACACACCCGGCAUGUUCCCCAC

ACUGGGGCCUUUGCACCUGUGACUCCCCGCACCUGCCACUCUCGUCCAGAGCCUGGCCUG

GCCCAUUCCCUCCGCCGUCAAAUCUUGGCUCAAAUGUCACUUCCUUGCAGAAGCUCUUCC

AGACUGUUCUGUCCAGAGGAGCCCCUGCCUUGUGCUCCUUGUCCCAUCCUGUUUCACCCA

CGCGACCCUCUGAUCCUCUGAGUGCUGCUUCUGUGUGAGGAGCACGUUGCCAGUCUCCCC

AGCGGCAGGGCAGCCUCCAAGGGCAGGACCUUGUCUACCCCGUGCCUUGUGUCCCCACAG

CCCUGGUAUGCAGAGCUGGGCACACAGUAGGUGUUCUGUAAAGGUUCAGGGAAUGAAACG

CGAGCUUCCCAGGCUGGCCUGUUCCCCCAGGGACAGGUGAGACUUGCAUAGCAAGGAAAA

GCUUCCUGUGUUCAUUCACUCCACGUGUGCCGAUGAAGCUGACAGGGUGUCCCCAGGAGG

CGUGGUCACUCGCUCUGACUAGGAGACUCCUGGGCAAUGUCAGGCAGGUUCUCUCGUGCU

CAGGAAGGAUGUCAGGGUGGCUGAGGGGCCAGCCUGUACCCAGAGUGUAUCUGGGGCUCA

AUGGAAGAAUAAAGCCCCCGGUAAAAAUAAACCCUGGCACGCUUGGCUGCCGAGGUGGGG

UGUGAGGGGGACGGUCACCCUCAUUGCUACAUGGCCGGGAUCACUGCCCUUUGCACCUGC

GGCGAGGCCCCAGGCAUGGGGAGUCCCCGAGCUGACAGCCCAGGCCCCAGUCCUGAUCUG

CUGCUUCCCUGUGCCCCAGGAGAAGGAGAGGAACGCGCGGAGGAAGAAGAAGAAAGCGCC

GGCGGCGGCCAGCGAGGAGGCUGCAUUCCCGCCCGUGGUGGAGGAUGAGGAGAUGGAGGC

GUCGGGCGUGAGCGGAAAUGAGGAGGAGAUGGUGGAGGAGGCUGAAGGUGAGGGCUGGCG

CGGCUUGGAGGUGGCUGGCAGCUGUGGGUCCGAGCUCUGAGGGAGGGCAUAGCGGGAACU

GAUCCCGGCAGCCUCCUCUCCCCUGAGUCAUCUGGGUCCUCCUUAGAAGGGGCCUCGACU

CCCUGGAAGGUGCGGGCCUGACUUGCCAGAAACUCACCAGUAAUGGGCACAAGGAUGAAG

GCCAUAACAAUACUGAGAAUAAUCAGCUUCUCUGGGAGCCCCAAGCCUCGCUCAGCUCUU

CUGAUGCCCAUGAGUCCAGUCUUAUGCUUGGGUGACCAGACGUCCAGGCCAGCCCUGUGG

GUGCCUGCUGUCUUUCACCCCAUCCGAUGUCACAGUUUAGAAAAAAAUUUACUUUGGAAA

AGUUUUUUUUUGUUUUUUUUUUUUUCAGACGGAGUCUUGCUCUGUCGCCCAGGCUGGAGU

GCAGUGACGUGAUCACGGCCCAGUGUAACCUCUGCCUCUCGGGUUCAAGCAAUUCUCCUG

CCUCAGCCUCCUGAGUAGCUGGGAUUAGAGGUGCCCGCCGCCACACCCGGCUAAUUUUUG

UAUUUUUAGCAGAGAUGGGGUUUCGCCAUGUUGGUCAGGCUGGUCUCAAACUCCUGACCU

CAGGUGAUCUGCCCACCUUGGCCUCCCAAAGUGCUGGAAUUACAGGUGUGAGUCACCACG

UCAGGCCCGAAAAUUGUUUUUUGACCAGAACUCUUUUACAAGGUAACCAGCAUAAAUAGA

ACCUAUCUGUCAGUCGGUAAAUAUUGUACAAGUUAUAUAGUUAUUUUCAGUGUCUCCUUU

UAUUCCCAAGUGUCCCUAUUUGAGUGAUAAAUAAUUCAGUCCCCUUCUUUGGUGUCCCCG

UUUUCUCUAUGGGGAAACCGAGGCUCAGGCACUUAAAGGCCUGGGCCCAGGCGCCCCAGG

UACCACGGAGUGUGUUGUUUGCACCAGCUGUAGACUAGACAAAUGCCCCACCAGGUGAGU

GCUGCUGAAGAGAGGGCGUAAAGCCAUCGUGUGCAUGGUGCUGGUGGGUCCCCCCGAGUC

CCCUCAAAUGCAGGACUGUGACCACAUGAGCUGUGUGUUGUCCAGGGCUCCGGAUAACAC

ACGGCUCAACUUUGUUAUCAGUGUGUAUAGACCCGGGAACUGAACACGGGCUGCCGACAC

CUGGCCCCGCACUCUCGGUCACGAGUGCAUCCACCUUCGACCCCCACCCUGUGCCUCUUU

UUCCCUGUCAAAGGGGAAGCUGGGGGUCUAGACUGCUCCCCCUGUGGCGUGACCGAAGGC

CCCUGGAGGACCUCAGCCCUUCUCUGCUGAGUGUCCCUGACUCUCCUUUCUGACUCUCUG

AAGGAGAAACAAGCAGCCCGGCGGAGCCCAAGUCCUUGGUGAAUUCAACCCUCCUUCCUC

CUGCCCCGUCUGGGGUCCUUCUUGUUGUUCCUGUUGUCCCUCCCUCUCUGCCUUUGAACG

CCCUGGCUUCUCCCUGGCAGAAGGUGCAAGUCACCUCCUGGUUCCAUAAGCCCAGCCUGC

AGAUAAAUCCAGGCUCCUCCAGCCCGCAGGGUUUCACCAGCCCGAAUCCCCGCUUCCCGC

UGGAAAUGGGCAACUGGGCCCUUGGGUGACUCAUCUCAAGUUGGGGGCGGGUCAGGGACA

CUGAGUCUGGCCUCCCCACCAGCUUUGGGUCUCUCUGACCCAAGUUGACCAGAAGCCUGG

CUAGUGUCUGAUGGAAGGAAGGCCUGGGCCACGAGUCCCUUGGGUCGUCCCAUUUUGGGU

CCUGGGUCUGUAGCCAGGAGAAGACCCCGGGAGCAGAAACCCAGUCCCCUCUGUAAAUAU

CCCUCCAGUGUCUGAAUGGGCGGGAUGCAGGCUUGGUGGGAGCAUUUCAGGAAGCCAAGC

CAUGGCCACACAUUAAAACCGGAGCAGACGCCACCCAGGACUGUGUGGCAACGACCCCCG

CAUCGCUUGCAGAGCUUACGUAGCCCCGUAGGGACGGGGAGGGGCAUGCUCUGCUGGGUG

GGGGAGGAGGGAGGGCUGAGGUGGCUGGAGCUGAGGCUUCUGUGACAGUGGUGGUCUUCC

GUGGCUGCUCUGAGAGAUCAGGGACGGCAGGUGAGAGGCCCUGAUUAAGCCUCCUGGUGC

CCAGGGUGUUAGCCCAUCUUAAGGACGAGGAGCCCAGGGGAGAGGAGAACCCCUCCCAUG

GGGUGUCCCAGCACAUAGCCGGUAGGCUCGGGGUCUGCCGGCCCAGCCAGCGCACGCUGC

GUGACCUGUCUCUUGUGCUCAGGAUGGGGAGGUGAGGCAGGUGCUGGGUUGGAGCCUGAG

GUUGCCCUGCCUGAAUCUCCCCGGAGACCCAGCCAGCCCCAGCGGCACUGCAGGCCCCUC

CCCGGGGGCAGUGGAAAGCGUCUUGUUGCAUCUGGGCUGGAAAUACACCAGCGCGGGGCU

GGGAGUGAUACCCUGGCCCAGUGGUUUCUCCAGUAAUAAUAAUAGUAUCCUCAUAGUGAA

UGAAGCCAUGCUGUUCUCCGCAUUUGACAGAUGGGGAGACUGAGGCAUAGCGGGGUUAUC

CAAGGCGCAUUUGGUGACCGGGGUUCAAACCCAGGCAGCCCGGUACUGGGGACCUCCAGG

CAGAAAGGAAAGGAGGCCAUCUGGUGGUGUGUUUCUAUUUUUUAUUUUUGGUGGUUUUUU

GGUCACUCUUGUUAAGCACCUACUGUGUGUCAGAUGCUGCACAAAGCACUUCACCUGGAU

UCUAGGGCAGCGGUGGCAGAGUGUUCCCUGGGAUUUGUUAGAAGUGCUGAUUCUUGGGCC

CCAGUCCACACGUACUGAAUCAGAAACCUCGGGGCAUAGUGCAGCCACCUGCACUGUCAC

AAGCCCUUGGAGUGCUUCUCAUGCAGGCUCCUGCCCCAGACCCCCUUGAUAACUGUGAGA

CCGAGAAGUUAACUGCCCAUUUUGUAGAUGAACAAACUGAGACCCAGAAGUUUCAUCCCU

GGUCCUGGGGCCCUCAGCGUUGGCCUCCCAACGUCCUGUACUCUGACUGUGGAAGGAAGG

GAAUGACAUUGCUGAAGUCACCAGGCAGAGGAAGGCCAGGGGUUAAGGACACAGGAAGCU

GGGCUCAGUGCUCUCCCCUCCACAGCGUGGCCUUGGGCAAGCUGAUUAACCUGUCUGCCU

CCAUUUCCUCAUCUGCACAUGAGAGUUGGUGGUCCCAGUCUUAGAGGUCUUAAAGGAGUC

GUGUGUGAAGAACUCGGCACGGAGCCUGGCCUGUAUGCAGCCAGCGUUGCUGCUGUUGCU

AUUGUUUCUAAAAAGGGUGGCCUCUGCUCUGGGGCCUGAAGGGAGGUGAGAAGCAGGAGA

GACUGGAGGGUUGGUGGGGGGGCAAUGAAGGCUGAAGAGGCCCCAGCAGCCCCAAAAGCC

UCUCCCUGCAACCUUGCCCAUCCCUGAGGUCAACACCUCCCACUCCAGGCAUCCUGGGAG

CCUGGUUCCACCAGGAGGCUGAUGACUUAGGGCAAGGACACCCAGAGUGGCUGAAGCUCA

CUUUUCACCCUGAACAACCCCAUUAUUGGCUGGCCUUGAACAGGCCAUUGGACCAGCCUG

CUCAGGGCUAUUGCAGGUCACCUGUGGCCGCUGGCCAGAGCCCAGAGGGAGAGGGCUCAU

CGGUCCAUGGAGAAGGGCAGUCGGGGCAGGAGAGCGAGCAGCAGCGAGGCCUCACUGCUG

UGCACUGGGCUUCAGGCCCCUCUUGGCCACGUUGCCUCCUCCCUCCCCUCCCACUACUUG

GUGCUGCCUCACUUCUAACUCUUCUCUGACUUCCUUCCAGCCUUACAUGCCUCUGGGAAU

GAGGUGCCCAGAGGGGAAUGCAGUGGCCCAGGUACGAAUGUGGCCGGCUGGCCUGGGGGG

UAGGGCCGGGCCGGGGCGGGCAUCCCAAGGUCCCCGGUCCUCCACAAGGAACCCCGCUUC

UGAGCACAUGCCUCUUUCUGUGAGCGCCACCGUUUGUGCUCCGCGGCCUCUGGGCUUCCU

UCCCAGCACAGUGGCUGUUCCCCAUGCUGGUCCCGGGCUCACUCCAGCAGACCAGUUUCC

CGUCUCCACUGCUUUGCUGAUUUUCACUGGUACGCUUGGGUCUCCGACCGGGUUUGAUUU

CCCUGAUAGCAAAUGCAGGGGUUUUCAAGUAUCCCCAUUUUACAGAAAAUGGGAGACAUU

GAGACUCAAAGAAGAGCCGGGUGUGGGUUUGGGGGCUAGAGAGCCAGGAAUGCGGCUGGG

CUCUCCUUGCCAUCCUUGGGUGCGUGCUUUCAUCCUGCCACUCCGCCAGCACAGAUCGCG

CUCCUGCUGUGUAAAACCCAUGCUGAGGCCGUUGGUGUGGGCUGUGCCCGGGGCCCCUGC

ACACGCACUGGCCUCGUUGUCCACCGUGCCCGGGGCCCCUGCGCACGCACUGGCCUCAUG

AUUGUCCACCGUGCCCAGGGCCCCUGCACACGCACUGGCCUCAUGAUUGUCCACCGUGCC

CAGGGCCCCUGCACACGCACUGGCCUCAUGAUUGUCCACUGUGCCCGGGGCCCCUGCGCA

CGCACUGGCCUCAUGAUUGUCCACUGUGCCCGGGGCCCCUGCGCACGCACUGGCCUCAUG

AUUGUCCACUGUGCCCGGGGCCCCUGCGCACGCACUGGCCUCACUGCCCACUGUGCCCGG

GGCCCCUGCGCACGCACUGGCCUCACUGCCCACUGUGCCCGGGGCCCCUGCGCACGCACU

GGCCUCACUGUCCACUGUGCCCGGGGCCCCUGCGCACGCACUGGCCUCACUGUCCACUGU

GCCCGGGGCCCCUGCGCACGCACUGGCCUCACUGCCCACUGUGCCCGGGGCCCCUGCGCA

CGCACUGGCCUCGUGAUUGUCCACUGUGCCCGGGGCCCCUGCACACGCACUGGCCUCAUG

AUUGUCCACUGUGCCCGGGGCCCCUGCACACGCACUGGCCUCAUGAUUGUCCACUGUGCC

CGGGCCCCUGCACACGCACUGGCCUCAUGAUUGUCCACUGUGCCCGGGUCCCCUGCACAC

GCACUGGCCUCAUUGUCCACUGUGCCCGGGUCCCCUGUACUCACACUGAGCGCAUUCUAC

ACUGUCAUGUGACGAUGAUGGUGGCGAUGGUGGUGCAGCAGCAGGCAGUCACCAGGCAGU

UGCCCUUGCCCUCACAAUUGCCAGCUUCUCCGCCCUGUGAGAUGGGAACUAUUGUUCUCC

CGUUCUGCAGAUGGGAAAACUGAGGCCCAAGAGGUAACCUGAGUUUCCCGUGGCACGCAG

CCAGUGCGGGGCAGAGCCGGGGUUAGAGCUCAGAUGAGAACAUGGAGACCCCCAGGACGA

AGGCCUUUGCCCACGGCCACCCUCAGGCUGGCUCCAUCUGUCGGGGGUGCUGACCACCAG

GAGGUCCUGGGGUUGGGAAUUAACCAGGUUAUGCCAGCAGGGUCAGUCUGGGAGAAAGGC

GGCCAGGCGAGGGCAGCAGCUGUGGGUAGGGGCCUGGUCAGGGGCUUUGUCUCAGGGGUG

GGGCUCUAGGCAGCAGCCCAGGUGUGUAGGAGCCUCGGGGCUGUUUUGGCAUCAGGCGAC

AGAGCGGCCUCAUCCGGAGGGUCAGGGCCAUGCAUCUCUUCUGGAGGCCCCGCCAGACCC

UGCUCAUCCCCUCUUCCUUCUCGCCCUGCACAGCCACUGUCAACAACAGCUCAGACACCG

AGAGCAUCCCCUCUCCUCACACUGAGGCCGCCAAGGACACAGGGCAGAAUGGGCCCAAGC

CCCCAGCCACCCUGGGCGCCGACGGGCCACCCCCAGGGCCACCCACCCCACCACCGGAGG

ACAUCCCGGCCCCCACUGAGCCCACCCCGGCCUCUGAAGCCACCGGAGCCCCUACGCCCC

CACCAGCACCCCCAUCGCCCUCUGCACCUCCUCCUGUGGUCCCCAAGGAGGAGAAGGAGG

AGGAGACCGCAGCAGCGCCCCCAGUGGAGGAGGGGGAGGAGCAGAAGCCCCCCGCGGCUG

AGGAGCUGGCAGUGGACACAGGGAAGGCCGAGGAGCCCGUCAAGAGCGAGUGCACGGAGG

AAGCCGAGGAGGGGCCGGCCAAGGGCAAGGACGCGGAGGCCGCUGAGGCCACGGCCGAGG

GGGCGCUCAAGGCAGAGAAGAAGGAGGGCGGGAGCGGCAGGGCCACCACAGCCAAGAGCU

CGGGCGCCCCCCAGGACAGCGACUCCAGUGCUACCUGCAGUGCAGACGAGGUGGAUGAGG

CCGAGGGCGGCGACAAGAACCGGUGAGUGGGCGCCAGGCAGCCCUAACCUUGGCUUUUGU

CUGCAGACACUGAGCAGCGACUACCUACUUAGACAGCCGCACCCAGUCUGGCUCUGCCUG

CUUUGGGGGAGCCCGAGGUGUGGUUAGGGAGGCAGACUUGGAGUAGAGUCCAGGAAUGAG

AGAUGCAGGCCAGGAAGGGGCGGAGGUGUGGCCGGGAGGACCUCCCCAGGAGGUGACAUC

CAAGCUGAGACCCGGACAGAGGAGGCAGCCCUGGGCAGCGCUAGGGAGAGAGUGUGCCAG

GCAGCCUGCUCGCUUCCUAGGGCCGCCCUCACAAGGUAGUUCGAGCCAGGUGGCUUUUAA

CAAAUACAGUCCUGGAGGCCAGGAGCCUGGGGGCACCAUGCAGGCCGAGACGCAGCCUCC

GAGCCUCUGGGCAGAUCCAUCCCGCCUCUUCCAGCUCCUGGCCGCUGCUGGCGGUCGUGA

UUGCUGGCAUGCCACUCCGUCUUUGGCUCUGCUGUCACAUGACCUUCGCCCUGUGCAUCU

CUGUGUCCACACUUUCUUCUUCUGAGGACACCAGGCAUUGGAUGGGCAGCUGUCCUAAUC

UAGUGCAGCUUCAUCUUAACUUGAUUAUAUCAGCAAACAGUCUAUUUCCAAAUAAGGUCA

CAUUCACAGAGACCCUGGGUUAGGACUUGAAUGUACCUUCUCUGGGGGGACACAGUUCUA

CCCUCAGCGUGGUGGGAACAGCAAGUGCGAAGGCCCGAGGUGGGAAUGAGCUUGGUGGAU

UCAGGUGCCAAAGGGUGGCUCAGGGAGGGGGCAGCCUGGCAGGACGGGGGGCAGCUCUCA

CGGGGGUGGGGGCACUGAGUCACGCCCUGGCAGGUGGGGGCUGCAUUUUGUUCUCAGGGC

AGUGAGAAGGCCUUUACGAGCCUCUCCCAAGAGAGCCAGAUGUGAUUUACCCAUGAAACC

AUGUGGGGGAUUUGCUGGGAGUGAGAGGCACAGUCAGGAGGCAGCUGCAAGUGCCCUGGG

GCUGGUGAUGGAGAUGCGAGCGUGGUGGCCGUGGGGUUGGGGAAAAGCCACAGGUUCAAC

AUGUUUUUGGUGGCUCAUCUCCCAUCAGCCAAGGGUCCAGGGCUCAGGAUCACAGGUGAU

CACCUUGGAUCACAGGUGCAGAGGUUCCCCUUGCAAAUUCCUCUCCAGCUGGGGUGGCCU

GGAGGACCCCAGUGUCGUCCUGUUUGGGUGUUGAUGUCUCUGAGGUGUGUUCUUUCCUUU

CUGGCCUCUGUCUUUGCUCCUGGCCUGGGUGACCCCACAUCCCUGAGAAUGCCACCUUUU

ACCCAUUUUCAGCACAGAUGCUUUGGGCCUCAAGGCCUCCAGCCCAGAACCAAAGAUGGC

UACCCAAGUUAGGGCCAAUCUCGGUUGGCAGAGGGCAGACCCGCGGAGACUGCGUCCUGC

UCACAUCUGGAUGGCUGGAGGAAUUGUUUUCUGGUGGGGCAGGCAGGCCAUUUUAUUUCA

ACUACAGUGUGGUCUGUUUUCUCCCCUCCCAAGUCCCUUGGAAUGGUCCUGGGCAGAGCA

AGCGAGGGAGGAAAAUCAGACCCGCAGGGUCAUGCGGGGCCCUUGUCUCUGCCCUGGGGU

UCACCCUGGGCCAUGUUAAAGGGGGAAGUCCUAGGACAUUCCCUAGGAACUGAGGUUCAC

CCGGAUCUCUAUAGGUUCCACAAAACAAAAUCUCAGGGUCCCCAGGAACCACUGCCAACA

GGAACCGCGCCCAUGCGUGUGAGAAGUGGACGGCCUGACCCUGACCCUGUGAUGAGCAAA

GGCCUGGCCUGCGGGUUUCUGAGGGCACCAAAUCCCUGAGCUUCUUGCCCGACAGUCCCA

GCUUGGAAAACAGUGUUCAUGGUGUGGCUGGGUCUGCCCUGGAGGCAGGCAGCCAGGGCC

AUUGUGCCUGGAUAGCGGGGUCAGGGCAGACGGCUCCCCGCCCCGCUCCUGCUGGCUGUG

GGGACAGAGUCAUAGAAACACAUGUUUCUGCCUUCGCCGGGGGACCGCUGAGGUCAUGGG

GCAGCCCCAUUGAUAAGCUGCCUCCCAGAGUCUGUAUUUUGGGUCCAUGUGGCUGGCCCC

GGACUGGGGAGCCGGGGGAUCAGCUUGCAUCCUGUCCGUCCCCAGAACACUCAGGCUAGG

CCUGAGGCCCCGCAGGGCACUUGCAGCCUCCCGUCCUCUCCCCUGCAGCCCCGCCCAGGG

CCGUGUCCUCCCUCCCCGAGCUGGGCCUGGCGUACGUCCACCCCUUGCCCAGUGUCCAGC

ACACAGCCCCCGGGUGCAGGAGUCCUCGGCUGGGAGUCAGGACACCUGGUUUGUCCCGCC

CUGUGCGACUAGGCCCAGGCCCCCGCCCUUGGGGCCUGCUUUGCCCUCUCAGUGAACGAG

GCUGGACUCUGGCCAGCCUGCUGACCUCGUCCUGGCCGGCAGCCCGCCACCCCUCCCUCU

GCCGGCCACGACUGCGCAGGGCCGGGCGGACGCUGGAGGACGCGGACGAGAUGCGUCAGA

GUAGGCCCUGGCAGCCAGGGCCUCCCGGGAAGGGCUGGUGGGACGGGCGGGCCCCCCAGG

CGACCCCUGCUCCUGGCUGCCCGCCCAGUAUCAUCCCUCCUGCCUGGCAGCCCCAGUGGG

GGAGCUCUGUGUUCCUGGAUGGACGCAAGAUGACUCUCAUCUGGCCCCCCGCCCCGGUGA

CUUUGGGAGAGAGAAAAUCACAUUCUCCUUCGGCCCGCUGGCACUUUCUUCGAGGUGGCU

CUGCCUGGGUGUUUUCUUGGCAGACGACCAGAGAUAGUGGCUUCAAUUGCAAAACCCCGA

GCCUGCCUGCCUUCCCCUCUCUUACUCUUCAGAGAAGGUGCCUGGGUGCUUUUUUGGCAG

GGCCUGGCCUUGGGAGUCCCCUCCCCCAAGCCUCAGUUUUCUCAUCUGUAAGGUGGGCGU

GGAUGGCUUAUUGCAAGAAUAACUCAGUUAAUGACGGGCCACAGUGGGCGCUCAGAGAUU

AGCUGCCCUCCUUUGGAUCUGCUUUGGGACACCCCCUAGAGGUCACCCCAAGAGGGGGCU

GGAGUGCAGCUCCUUCAGCUGUCCCUCUGGCAUUUUGGAAGAUCAUAACAGCAAUUUCAC

CUUUGUUACUUUAUUCUGCAAGUGACUGAUGGCACCAGCAUGCGCCAGGCGCCGGGGGCG

CUGGAAACUGCACAGACAAGGCCCUGACUCCAGGCACUUACAUUCUAGUGAGGGCAUGGA

GAAGGAAACCAUUGUUUACGGGAUGAGUGGGUGAUGGGCAGAUGGGGCAUGGGGAGCAGG

GGAGAGUGGGCUGGUCAGGCAGGGAGGGCCUCUGGGGAGGUGACAUUGGAGGGCAAUGAG

AGCCUUAGUGUGAGGGAAAGCCAGACCCCCUGGGAACCACCAGUGCAAAGGCCCUGUGGC

CGCAUGAGCGUAUGUCCAGUGGACAGCAGAGAGGCCUAUAGCAGGGUGUAGAGUGGAGUG

AGCAGCCCAGGGCAGAGAGGGCGGAGAGGUCAGAGAGGUGGGCAGGCCCCACGGGUGGAA

GUGACGGGUCAGGGUUUUAUCUUGGGGCCUUGGAGAGGCAAGGGAUGCCUGUUCACAAAC

CAGCCCUCCCUCUCCCUCCUAGCCCUAGGAUGGUGGCAAGGAUUGUCCCAGCUUGAUGAC

CAUGGGGAAACUGAGGCAAGGGCUGGGUGGAGAGGGGAUGUACUGUUGCAGGCCCAGCAU

GUUCCGAUGAAUGUCCCCAGGCCCUUAUUCCCCCUCUCGUGCCCUGCCCCCCAACCGGCC

UUCAUGAGUGGGAAGGAAAAUGACAGUGAGGAGAAGGUGGCCCGACCCACUCGGCCCCAC

GUGCUGGUCACCAGCAUCCCCACGCCCACCCACGGACAGCCCUUGAAGCUUUCGUCUCCA

CUCUCCAGAUUGAGAAACCGAGGCACAGUUGGCUAAGUCACAUGGUCAGAGAGGGGAUCC

GAGCCUUUGCUCGCAACUGCAGGGCUGGGUAGGCAGGGUGGGGCCCCUCCCAGACACAUA

CUCUGUGCCAGGCCCUGAGAGUCCCCGCCCCACCCAGGCCGCCAGAAAGGAAAAUAGAAG

UGGCUGCCUUCCAAGAAAACAAAGGAGAGAGGCCGGUCGCGGUGGCUCAUGCCUGUAAUC

CCAGCACUUUGGGAAGCCAAGGUGGGUGGAUCAUUUGAGGUCAAGAGUUUGAGACCAGCC

UGACCAACAUAGUGAAAGCCUGUCUCCGCUAAAAUACCAAAAAAUUAGCCAGGUAUGGUG

GCGUGUGCCUGCAAUCCCAGCUACUAGGGAGACUGAGGCAGGAGGAUCACUUGAGCCCAG

GAAGCAGAGCUUGCAGUGAGCCAAGAUUGUGCCACUGCACUCCAGCCUGGGCAACAGAGC

AAGACUCUGUCUCCAAAAAAAUAAAUAAAUAAAUAAAAUAAAACAAAGAAGGGAGAGUGG

AGGGUGGGGAGAGCCAGCCAGGAGGGCUUCUGGAGGUGGCGCUCCUCCAGCAUGGCUACG

GGACAAGGGGCGGUCAGAUGGGCCCUGAGCUCUGAGCGUUUUCCACCUUCCUCCUCCACU

AUUGGUGGCCGCUCCCUCUGGGGGCUUAGGACUCUCGUUCCCCUCGAGAACUUGAACCCA

GCCCUUGUGCUGGCUGCUGCCUUCUUUACCCAUGAGAGAGCUCUGGCUGGGAUCAGAAGA

CCUCUUGUAAACUAUAAAGUGGUACCUCCAUGACCACAGUCCCCACUUGCUCUGGGGGCU

CAAGAAGGGUUUGGGCCUCUGUGUGUGCCCCCCAGAGGGUACCUGCUGAGAGCUUCUGGG

CAGCAGCCCCUGCCCCCGCUCACCCCCUGCCAAUCUGACUGCCCACACCCUACUAGGUGA

GGGGCCACAGGGAAACCAAGGCACAGGGAGCACUCAGGCCCCACAUAGAAAACGCGAGCU

CUCGGGCAGACUGCAUGGGACAGUGGCACCUCAUGUUUUGUGUGUGUUAUAAAGUCAUAU

UUUUAUCUCUAUAGAAUUAUCUUUACACAUAUACAAACGUUUGCAUUUCAACCUUAAAAU

UUAUAUUUUAUUUUUCUAUUUGUACACACAUAUAAAAAUAUUAUUUUUACUGUUCAUAGA

GAAAAUACAGCUGAUACGUCGAACCCUUUAUGGCUAGCAUAUAGUAUGGUCCUCAUUUCA

UUUUGUAACCGUUAUUAAGAUAUCAUUCAUAUACCAUACAUUUCACCAUUGAAAGCGUAU

CAUUUAGGCUAGGCACAGUGGCACACACCUGCAAUCCUAGCUCUUUGAGUGGCUAAAGCA

GGAGGAUAGCUUGAGCCCAGGAGUUCAAGACCAGCUUGGGCAACAUAGAGAAACCUUGGC

UCUACCACAAAAAAGAAAAAAAUGCAAAAAUCAGUCAGGUACGGUGAUGUGUGCCUUUAG

CUUCAGCAAUUCCGGAGGCUGAGGUGGGAGGAUUGCGUGAGCCCAGGGGGUCAAGGCUGC

AGUGAGAUUACAGCACUGCACUCCAGCCUGAGUGACAUAGCAAGACCCUGUCUCAAAAAA

AACGUGUAUCAUUCAGUUCUUCUUAGUAUAUUCAGUGUUAUACUAAGAAAGAAACCCUAU

GCCCAUCACUAGUCACCCCUAUCCUCCCUCCUCCCAGCCCUGGCACCCACUCAUCUCCUU

UCUGUCCCCGAUGGAUUGGCCCGUUCUGGACUUUUCACAGAAACGGAAUCUACACUAGAG

GCCUUCUGCGACUAGCUUGUUUCACGCGCAUCGUGUCUUCAGGGUCCACCCACGUCAUAG

CCCGUGUCAGAGCCUCAUGCCUUUUGAUGGCCAUGUGUUCCAUCGUGCGGACAGGGCACG

CUUUGUCUCCUCAUUCGUCUCUUGGUGGAUGUCGGGUUGUCUCUGCCUUCUGGCUGUUGU

GCGCUGGGCGGUGCUGCAGUGGUGGGCACGUGCAGGUUUCUGCAUAGACAUUUGCUCUCA

UUUGCCUGGUGUGUGCCUAGGAGUGGCAGUGCUGGGUUUAGGGCAAUGAGGGCUCACCGA

CUGCUUUCCAAAGUGGCUGCAGCAUUCUCUUUUAAAUUUCAGCUUGCAAGUGCGAGCUGG

UUGAAACGGAUGGUGGUAGGUAAACCUGUGGGGAGGGACAUAGGGGUGGCGGCAGUUGAA

GCCGCAGGUGGAGAAUGUUGGGACAGGUGAUACCAUCUGUGUUUGGACCUGGGUCAGGGU

ACCUUGUCUGUACCUUGAGGAUGGCGGGGAGAGCACAGCUGGGCCCAGGUCCAGGGAGUG

AGGCAUGGCAGUGGCCAAAGGAGAGGCUUUAGGUGGUCCUGGGAGAUCAAGCAGGGUCUG

GACAGGUGGAGGGAGGGGAGGGAGGAGAGUGGGCUGGCGGUGGGGGCGGUGGAGGUGCAU

GCAGAAGUCAGGGUGACCUUCCUCGUCUGGCCCCCGUCUGUGCGCCAUGGGCAGCCUAAG

CAGGCAUCACCGAGUCUGUGUCUGCUCUCCCCGCUCAGCUGAGCGUGUGGCAGAUGCAGA

CAGCAGGCUGGUACCUGCAGAACAGGCCGGUCACAUGCCGGUGGCCCCAUGGUCCUGCUA

UGGCAGAGAUUGAAGGAGGGGACUGCCGGCAGGGGCCUCAGCACCCUGGGCAUCGGCCCU

GCUCAUCUGCAGGAAGGAGAUGGGUGCCUCUGGGCGCCAGGCAUCCAGCACUGGCAGCUG

GGGACUGGGGUGGUGUGAGUUGUGUCCCUGUUGUCACAGCUCUCACCUCGAGGCCAGUAG

GCUGUGACUUAUAGUCUGCAUAGGACGUCGUCACUGUGGUCACAGCACAGAGUAGGGGCU

GUGCCGACAGGACCUGCCAGCUUCUGGCACCCAGAAGUUUUGCCCUCUGGCUGAAUUCCA

GGCCCGUGGCUGCUGCCCUCACGCCUGCCAUGUGGUCAUUUCACUAGCUUGGAAGACCUA

GGACAGAGACUGGGUUCUGCGUCGCCUCCAAAUACACCCAAAUGCAGGCCGGGCUGCUUC

CAUACCCCCAAACGUAGGCCGGGCCAUCUCCAUACCUGCAAACGCAGGCCAGGCCACCUC

CAUACCCCCAAACAUAGGCUGGGCCACCUCCAUACCCGCAGACGCAGGCCGGGCCACCUC

CAUACCCGCAAACACGGUCCAGGCUGCCUCCAAAUACCCCCAAACUCAGGCCGGGCUGCC

UCCAUACCCCCAAAUGCAGGCCGGGCUGCCUCCAUACAUCCAAAUGCAGGCUAGGCUGCU

UUGUGCCUAGCAAAUGCAGGUUGCAGGAGGGAGUCAAGAACUGGGCAGCCCCGGGCAGCC

ACACUGGGCAGGUGAAGGGUUCUCAGGCUGGAGCAGAUCCAGUUCCUGCCCUUGGAGACC

UCAUGCCAAGUGGGGAGCCAGAUGGGGAUUAUGGCAGGUGCUAUACCCGGUGCUGGGGAC

UUAGAAAUCCUGGAAGGCUUCCUGAAGGAAACGGCACCCACCGAACAGCUUGGGAGCCAC

ACCCCAAUGCACGCUCUGCCAGGCACCGUGUACCCUGUGUCGGCCUCUGGUCGCGCAUCU

CAGUAGCAGACAGAGGAAGGGACUGCGACUGUGGCUAAUGAGUAGACAGUGGGCAGGCCC

UACCUGCCCCUCCACACAAAGUCUGUGGCCUGGGCCCUUGUUAUCAUGUCCUCGUCCUUC

GUGGAUAGUGUGUGGGAGAGAGUCUAGGAAGUGGGUGCAGCUCCCCCACUCCUGCCUCUG

GUAACCGGAGCUCAUUCGGCUCCAUGCCCACUGUCUUGCCCUGGCACCCGAGCCUCUCAG

AUUCGAGUGAUGAGGAAUCUCUUGGUGAAUCUCUUCAUUUCUCUGUCCCUGAAUCGGGCU

GAUUCUUUGGCUUUCUGGCAGCUUCGGAAGGGCCACGUGUGGUGUGGGCCCCAGGUCUGG

GCUUCUCUGGGAUUUUCCUUGCCUGGAGAUGUCUUUGGGGAUGUUUGAUCCCAGCCUGGG

CCUCUAGCACUGCCCGGCUGGCCUCUAGGCUGGGGUGGGAGGCUGGCUUGGCCCCUGAUG

GGGCCUGCAAGAACCAGGGGAGUGAGUGAGGAGGCACUUGCCCGCCCACUCGUGCCUCCC

CUCCCCUGGCCCCAAAUCUUGGUCUGUUUUCCCAAGAGAAACCGUUGACAUUAUCUGGGU

GAGCUGCACAGUCGCCUCGUGUCUCAGGGCCUUAGCCUCGUCCCCAGGGCUUGAGACAGG

AGAGGCCAUCAAAACCGGGCAGCCCCCCCACCCUGGGAGAGUCCGGCUGUGGGCCCCCAG

CUGAUGGUGCUCGUGUGCCCGCAGGCUGCUGUCCCCAAGGCCCAGCCUCCUCACCCCGAC

UGGCGACCCCCGGGCCAAUGCCUCACCCCAGAAGCCACUGGACCUGAAGCAGCUGAAGCA

GCGAGCGGCUGCCAUCCCCCCCAUCGUGAGUGCCCACCCCCAGAGCCCCACAGAGUCCAC

CCUGACCUUGACUUUCCCAUUCAUUGAGACAGAGAAUCCCACGGGCGGGAGCAUGCACCU

GCAGCCUAGCUCAUCCGCUUCUGUUUCCUGGAGAAAUCCCCGUGGAGCUGAGGUGGGAAC

AGCUGGGGAAGCUGGCAGGCGUACCGGGUGGGGCUCCCAGUGAAGGCGGGAACUGGCGGC

CUAAGGCCUCCCCCCUUCCACCCCGCAGCACCCUUGAACCCCCAGAUUAGAUACUGGGUC

AUGGGGAGGCAGGAGCUUCCCCCACCCACUCAGCUAUAGCAACAGCUUGGCCAGGGAAGG

GUGGCCUACCAGUUCUGAUCCUGGGGCCCUCUGGGAACACCCUCCUGGAAGGUUCUCUCA

GCUGGGUGCGUGGGCCCUUGGAGGUGCCUUCUCCAGGGAGGUGAGUCUGGCCUUCCCAGU

UGCAACUGGGGGCUCCGGGCUAAAAGACCCUGAGCUGACCAGGCUGGUGGUCCCUUGGGA

CUUUGAGUGAUGGGCUCAGGGCGUGUCAGGCUACACGGCCCAGUCCCCGAGGCCCACAGA

AUCCUGUUGCUCAGUCCUGGCUUCCCUGAGACCCAGAGGAUUUGGGUCCACAGAAGGAGC

UGGAGGAGAGAGUCACAGCAGGCUCUAGCCUCCUGUCCACCACCAGGCUGCUAGGCCAGG

CUUUUUGGGAGGUGGGCUCCCCCUGUCCAUCACCAGGCAGCUGGGCUAGGCUUUCUGGGG

AGGUGGGUUCCCCCUGUUCACUACCAGGCAGCUGGACCAGGCUUUUUAGGAGGUGAACUC

CCCCUGUCCAUCACCAGGCAGCUGGGCCAGGUUCUGGGAGGUGGGUCCCCCUGUUCACCA

CCAGGCAGCUGGGCCAGACUUUUUAGGAGGUGAGCUCCCCCUGUGCACCACCAGGCAGCU

GGGCCAGGCUUUUUGGGAGGUGGGCUCCCCCUGUCCAUCACCAGGCAGCUGGGCCAGGUU

CUGGGAGGUGGGUCCCCCUGUUCACCACCAGGCAGCUGGGCCAGGCUUUUUGGGAGGUGG

GCUCCCCCUGUCCAUCACCAGGCAGCUGGGCUAGGCUGUCUGGGGAGGUGGGUUCCCCCU

GUUCACCACCGGGCAGCUGGGGCAGGCUUUCUGGGGAGGUGAGCUCCCCCUGUUCACCAC

CGGGCAGCUGGGCCAGGCUUUCUGGGAGGUGGGCUUCCCCAUGUGCUCUGUGCUAAGACC

UCCCUUUCUUGCCGCACCUGGGACCUUGAGCAUGUCGCUGGCUUUCCCGGGCCUUGGUUU

CCAGGCUGGUCGCGUGCAUGUCUCUCCCUGACCCUGUCACUUUCAGACACCCUCUGUGAA

UGCUCACUUCUGCCUGCUUCCCUCAGCAGGUCACCAAAGUCCAUGAGCCCCCCCGGGAGG

ACGCAGCUCCCACCAAGCCAGCUCCCCCAGCCCCACCGCCACCGCAAAACCUGCAGCCGG

AGAGCGACGCCCCUCAGCAGCCUGGCAGCAGCCCCCGGGGCAAGAGCAGGAGCCCGGCAC

CCCCCGCCGACAAGGAGGGUGAGUGCACACCAGUGGCUGAGUGGGGCUGGGGCAGCAGGG

GACUGACGGGCAAGGGAUGUUUGUGUGUCUAGAGCAGCUGCUGCAAACCCCAUGGCCACA

GUGGCAGGGAAAGCAAAGUAAACGAAUGGAACAGUUGAGCAGGGGGAGGCAGUUGGGAAU

GGUAGGGACUGCGGCAAAUUGGAGAACUCUUGUUUUCUCUAAAGUGGGCACGUAUUGGCC

UUGGCCAAGAGUCACCCCGUGGCAAAUCAGGCCUGGGGUGGUUCUUCCAGCAAAGCCAGA

AAUCCAUCUUAUGUGAAAUCCCCCUGGUCCCUGGUUUCUCAACACUGGCAACUUCUCAAA

AUUUUCAAAAGCAUUGUGAACACGCAAAAACAUGUCUACAGCCCAGCCUCUGAUUCGGUG

UGAAUCAGUCCACGCAGGAGCUUCUGGAAUCACAGAUGGGGCCCAGCACCCGGCUGGCAG

CAGCUCUGGGGCCUGGGCAGGAAGGGCUGCCCUCCCCAGCUGGCAUCCUGACCUUGCUCC

AGGCCCAGGUCUCUGGGUUCACCAGGAGCCAGGGUUGGGGGUGACUCCCGAGCCGCUGUG

UCACAGGGACUGCCCCGGGCACACCUCACUGUCAUCACGUGAAGCUCAAGGUCAGGAGAC

AGGCGGGCGUGAUUGCUUGUUCUCUGGAUCUUCAAUCUCUGCCCCACUUCCCACUAGGCC

CAAGAAAUUCUGAACCUGUGUCCCCAAAGAGGCAUCUUCUCCAGGUCAGCCUGGCACGCA

GCAGGUGCUGUUGUGCCUUGCAGGUGUUGGGGGCUGCUUCCCCAGAGGUUCCCCACCCUG

GGCUAGCCCCGGCGCUUGCUGGCAGGCUGCUCCAAAGGUCUGAGGGCAGCAGGACACCAG

GCCGACCUCCUGACCAGCACAGCCCAGGGACAAAUCCGGGUGACUGGAGCUACCAUCAGC

UGUCCCCUCCCCGCUGCCUUGGGUGUGACAUCCCCCUUCUCUUUUUUUUUUUUUUUUUUU

UGUUUGAGACGGAGUGUCACUCUCGCCAGGUUGGAGUGCAGUGGCACAAUCUCCGCUCAC

UGCAACCUCUGCCUCCGGGGUCCACCGAGGCUGCUGGGUCAGGACAUUUGUUGAAAACCC

AAGGCCCCAGGCACCAUGCUGGCAGAGCGGGGAUUAGGUAGGGAGAAGAGGAAGUCUCUG

CUCUGGGGAGCUGCUGUUGUUUUGAGUGAGGAGGAUGUUAAACCAGGGAACACGUAACGA

AGCAAGAUGGUUUUAGAGUCGUUCAUCCACCAUGAGAAACAAAACGGGUUGAGUGAAGAG

CGGCUCCGGGAAGGACCCUGUGGAGUGGGAAGCAGCCCCGCAGUGGGAGCUCUCUGGGUG

GGCAUUGCGGGCAGAGGGAACAGCACAGGGAAAGGCCCUGGUGUGGAAACACGCUCGGGG

UGUGUGAGGAGUCGCCGGGGGUGGUUGUGGCCAGGGAAGGGUGGGCGGGUGGACAGUGAG

GGAAGUGGGGACCGGAUUGUGUGGGGCCUCUCAGGCCACAGCGGGCAGUGGAUUUUGUCU

CAGGUGUGGCAGGGGCUGCUGGAGGGAUUUGAAGUGAGCGUGUGUUGCGGAAGGGGAAGG

AGGUGCUUGUUAUCUGAUUUGUAUCUUGGAAAGCUCUGUCUGGCCGUUGUUGAGUCAAGG

GCAGCAGCGGGAGGUCACAGCGCAGCUCUUGGCAGGCGACAGAGGCUGCCUGAACCGCAC

AGGUGGCCGUGGAGGUGGAAGGAAGGGCGCAUCGGGAGUUUGUUUUGGAGGCAAAGCCAG

UGGGGCGUUGAACCCGGUGUGGGGAUGACGCCAGGGAUGUCUCGCUGGAACUGGGAUGCC

CCAUGACCAGGGCUGGGCUACCUGGGGCCAGGGUACAGUGUGCGGCCCAGGGUGUGGCUC

UGGGAGACGUUGCAGGUUUGGGGCGCCAGCCCGGGAAGGAGCUGUGUAUCCAUGUCAGGG

GCCCCAGCAGCUUUGGCCAGCGUCAGAUCUGAUCAGGGCUGGAACUGCACGGUGCCAGCC

AGGUCCUGCUGAGCCAGGUCGCAGGCCCCUGUCCUGGGAGAAAGGGAGCCACCGGCCAGC

AGUUCCCGCGGCUGGCACUCAGCCCGUUGGUGUGGCCAGGGCCUGUGCCGUUUCCUGGCC

UCAUUCCCCGUGGGGACGGUGCACCUGGCGUUAGGCCCUGCAUGCCUUUCUUGCACAGCC

UGGCUUGCAGCUCAGCUCCCGCAGGGACACACAUGAACUUGACACCCAGCGCUACCUCUC

UGGUCUCUCAGGAAGCCAGCCUAUUCUGCCCCUGGCUCCCACACAGAGCGGCUCAGCCAA

GCAGGUCCCUCGGGGCCCCCUGGGCCCAACUGGAGGAACAGGUUGUCUCUCCAGGCUCUC

GGCCGCCCGCCCAGCCUGGCCUAGCCCCAGUUUGCCUUCCAGCAUCGAACAGCUCCCCUC

CUCUCUCUCCCAACUCUCUGGAACUGGGCUGGAGGAAGCACCAGAGGCUUACAGGGCUGG

GUAGCCAGAGGGGCCAGGUCCAGGGCGUUUGCAGGAUCUCCAGAUGUGAGGCAGGCCCUG

UACAGGUGCCUCCACUGGGGACUGGUUAAGAAUGAGAAUUCCCCUGCAUCUGUGCCCCCU

GCCUCCUGUGGGCUGCCCACCUCGCCAGGGCACAAGUCCAGGGGAAACAGUCUUCAAGGA

GAGAAUUGGGGGCAAUUUGGUACAUAGGCCUUCUUGCCCACCUGCUCUGCUCACAGCAGG

CAUCAUUAAUUGAUCUUAGCACUCUCCUCCGCUGUCACACCCUGAUGGGCCCUCAGAACC

UCCUUUGUUCAUCUGGAGGCGGCGUGAUAGAUGACACCUGCCUGCCAUUCCUGUCCCAGA

ACCCAGAGGGCUCCGUCAUUCCCAAGUUGUGUCUCUGCUCAACUUCUUGGCCGGUUAUCU

CUCCAUAGGAUUGGUCCUCAGGGAGAGGUUCAGAGCUGAGGACAGAUUUCAGCAAAGGGC

GAGAGGCCAGCAGGAAGAUGCCAUGAAGCCAGGCCUGAGCCCCGGGGCCCCGCUGAGUUU

ACAGGAUCUCCCCAGGCCUGAAAUCUAGGACCCAUCAUUGCUUCUCUGCCGCAGGCCAUG

UGGCAUAGCAGUCUGGAUUAAGGCCAUGGUCUGCAGCCCCAGUGCCUGGGUUCGAAGCCG

CUAUCUGCCACUUGCUAGCUGGGGCCUUUGACCAGGUGGCUUAACCUCUCGGCUUCCAUU

UUCCCGUCUGUGAAAUGGGGACAGUCAGUCAGUUCCUUGUAGGGUGCUGUGAGAAUAAAU

GAGUGAACACGCUUACAUUGCUGUCAGCCGUGGAGCCCUGAGAGAGCAUGACCUCUCGGU

AAUGCCCUUUGCACACUCCGCCUGGUAAGUAGCCCUGCCUGAGCUUGGCUUCUCAGCCAG

CCAGCGCACAACCGCUGCUUGCACUGGCUCAGUGGGCUUCUGAUCCUGACUCGGCUAAGU

AUUUGCUGGUGAACUCAGAUGAGCCCAUUGGUGUGUCUGUACCUCAGUUUCCCCUCUGUA

AAAUGUGUCUGGCAGCAGUCUCUGUGUGAGCUUAACCGGAGGCUCAUGGAGGCCGUGGGA

UGCUGAGCACAGUGCCUGUGCUGAGUACCACUCAGCAAACUGAGGCUGACGCUGUGGUUG

UUACUUGGAGGGUCUGGGCUUAUAACCCCCCACUGCCCGCCACAACACACACACACAGGC

GCACACACAUCCAUGCACUCGCACACACAUGCACGUGCACGUGCACACACAUCACAGGUU

ACCUCCUUCAACAUUGUGUACAGGCACACACACAUCCAUGCACUCACACACACGCACAUA

CACGUGCACGUGCACAUACACACAUCACAGGUUACGUCCUUCAACAUUGUGUACAGGCAC

AUACACACAUCCAUGCACUCACACACACAUGCACAUGCACGCGCACACACAUCACAGGUU

AUCUCCUUCAACAUUGUGUACAGGCACACACACACAUCCAUGCACACACACACAUCCAUG

CACUCACACGCACGCACGUGCGCGCGCGCACACACACAUCACAGGUUACCUCCUUCAACA

UUGUGUACAGGCACACACACAUCCAUGCACUCACACACACAUGCACGCACGUGUGCACAC

ACACACACCACAGGUUACCUCCUUCAACAUUGUGUACAGGUGCACACACACAUCCAUGCA

CUCACACACACAUGCACGCACGUGCGCACGCACACACACAUAUCACAGGUUACCUCCUUC

AACAUUGUGUACAGGCGCACACACACAUCCAUGCACUCACACACACAUGCACAUACACGU

GCACGCGCGUGCGCGCACACACACACACACAUCACAGGUUACCUCCUUCAACAUUGUGUA

GAGCACAGGCCAGCAAGCUUUUCCUGCAAAGAGCCGGAGAGUGAACAUUUUAGGCUUUGU

GGGCCAGACAAUCUCUGUUGCAAGGAUUCAGCUCUGCCAUAGACAAUACAUAAAUGCACA

GAUGUGGCCACGCUCCAGUAAAAGCGUAUUCACAAAGUCAGACGGCGGGGCCAGGCGUGG

GGGCUCACGCCUGUAAUUCCAGCACUCUGGAGGCCAAGGUGGGAGGAUCUCUUGAGCCCA

GUAGUUCAAGACCAGCCUGGGCACACAUAACAAGACCUCAUCUCUACAAAAACAAAAUAU

AAACUAGCCAAGCAUGGUGUGGUGACACGUGCUUGUAGUCCCAGCUACUUGGGAGCCUGA

AGUGGGAAGAUCCCUUGAACCCAAGAGUUCAAGACAAGCCUGGGGCAACAUAGCAAGACC

CCAUCUCUACAAAAGUAAAACAAAUUAGCCAGGCAUGGUGCGGUGGUGGGCGCCUGUAUU

CCCAGCUACUUGAGAGGCUGAGGUGGGAGAAUCACUUGAGCCCAGAGGUGAAGGCUGCUG

UAAGCCGUGACAGCACCACUGCACCCCAGCCUGAGUGACAGAAGGAGACCCUGUCUCAAA

AAAAUUAGGGGGCAGACAGACCCUCUGAGAUACCAUUUGCCACCUCCUGGCUUAGAGGCC

UGUCCAGGGACCUUGGCUUGCUGGGCUUGGUUGAGUGCUUGUGGGGUUGCAAAGCCAGAG

GGUCCCGUUCUCGUCCUCAGCGGCAGCUGAGAGCCCCCAGCUCAGACCUUGAGAUCCCCU

CCCCUGCUGAGCAGAAUACCAUGGGGUUACAGAGCAGCGCCCAGGCAGGCAGACUUGGGG

GGCUUCCCGGAGGAGCCCAGGCCGUCACAGGCCACCACUACUGUGGGUGCAGGAAGGCCA

UUUGUGUAGGGUCAGCCAGGGCUCCCGAGGUCUGACUGCCCCACCUCCUGCCCUCAGCAG

AGAAGCCUGUGUUCUUCCCAGCCUUCGCAGCCGAGGCCCAGAAGCUGCCUGGGGACCCCC

CUUGCUGGACUUCCGGCCUGCCCUUCCCCGUGCCCCCCCGUGAGGUGAUCAAGGCCUCCC

CGCAUGCCCCGGACCCCUCAGCCUUCUCCUACGCUCCACCUGGUAAGUAGCUCCGCCCGA

GCUUGGGCUUCUUCAGCCAGUGCACAAUCGCUGUUUGCACUGUUUGCACUGGCUCAGAGC

UGCACACAGAAGCAUCUUAAAGCCUUAUGGGACCUGGCUGGAGGAACAGCCUGGUCAGUG

GAGGGAAAGGGGGAGAUCUUUUAAGAAAGCUGCCCCAGGGUUGAGAUGGAAGCCACUGGC

AAGAGGGAGCCACAGCAGGUGCUUGAGCUGGUGAGGGGCAGGCUUAGAGUGGGAGCUAUG

UUUUCCGUUGACGGCUUAGAGCUAGAGAAUGGGCAGAUGGUAGAAGAGCCUGGGGAGGGA

GCAGAGGCGCUACCUUGGUUCACAGACUCUGUGAGGCUGCAUUUCGGCCCUGUUCUAGGU

CCUGCAGUCCUGCUGGGCCUCACCAGCUGUAAAGCAGCACAGCCGGGGGCUGGUACAGAC

UGCUGUUGGUAAAGGCUGGUCCCGAGGGAUGCGUGGGCCAAGGGAGCCAGAAGAACAGGG

AAAGUCAUGCCAGGCAGACGGAACAGCAAGUGCAAAAGCCCAGAGGCAGGAGAGAGCAGG

CAAAGCUUGAGAAACUGAAUCUGGCCACCACUGCCGAAGCUCGGUGCCCAGUGAGCUGGG

GAGACCCAGGGGCCAGGUCAGGCGAGCCUGAAGGCCAAGUCGGGGGCUAAGACUUUCUCC

UGAGGACAGUGGGAGCCAUCGAGGGCACACAGCACAGGGAGGGUGUGCACCAGUAGACUU

UUCCAAAAAGGUCCUUUUGGUUCCUAUAAGGGGAAGGGGCUACUGGGGGCUACAGUGGAA

AUGGGGAGACAGGGGAUCCAGAUGAGAGUUGAGGGCGCUUGGAUCAGGGCCUGGGCAGUG

GGUGAGGUGGGAGGCUUCCCCAGGAUAGUGGUGUCUGCACCUAGGACUCGGGUCACUAAU

GAGGACAGAGCCAGGCAGGGCAAUAGAGGAGUGCGCAUGGAUGGGCACUUCCUGGCCAGG

ACAGGGAAGAGGUGGAGUGCAGCCUGGAGUGUGUGUCCGGCAGGGACGUGACCGCUCCUG

GGCCCCACAAUGGACAGACAUGUGCUCCACAGGUCACCCACUGCCCCUGGGCCUCCAUGA

CACUGCCCGGCCCGUCCUGCCGCGCCCACCCACCAUCUCCAACCCGCCUCCCCUCAUCUC

CUCUGCCAAGCACCCCAGCGUCCUCGAGAGGCAAAUAGGUGCCAUCUCCCAAGUGAGUGG

UAGCCCUUCCUUUCUUGGGGGCUUAGUCUUCUUAUCUGUAAAGUGGGGGCAAUGGGACCA

AUGAAGUCUGAGUGGGGGCCAGGCUCUCGGGGGCCUGGGUCAUCUCCACUCAGGCAUCUG

GGGUCUGAGCUGGCUGGGUCCUGCCCCAGGCACUCAGGGCCUCGUCCCUGGGGGGUCACU

CUGUAUUGCAGUCAAGGGCAUGGCUCAGGAACCAGGCUGUGUGGCCUCAGGCAAGUUACU

UAACCUCCUGUUUCCCCCACUGCGAAAUGGAAGGGCAAAUGCAAGGGGCUCAGAGGGCAU

GGCCCCUUCACAGGGGAGGGGGCCGAGGCUUGGGAGUAGCAGUGACUUGUCUGCCGUCAC

ACAGCUGGACAGCAGCCCCAGCCUCUGACCCACACGUGGGCUUUUUGAACAGGAGCGUCA

GGCACUCUGGACUGUGGGGGAGGGACCAGGGUGCCCCUGUGACCCCACAACUCCGCCCCU

ACAGGGAAUGUCGGUCCAGCUCCACGUCCCGUACUCAGAGCAUGCCAAGGCCCCGGUGGG

CCCUGUCACCAUGGGGCUGCCCCUGCCCAUGGACCCCAAAAAGCUGGGUAAGGCUCCUGG

CCCAUCCGUCUGGGUGGCUCAGGCUGGGCUCUGGCUGUCCUGUGGGUGGGGCGGGGGAGG

AAGGGGUAUCUUGGCCCACGGUACUCCAGGUAGCAGCUGGGAGGUCCCCCCUCAGCAGCC

CUGGCUGGAACAUGGUGGGGAGGGGCAGCGCUUCCCAGCCCCUGGCUCAGGUUGGGUGGG

GACAAGCCUCCCGGGACCCUGCUCCGGCAGCACGUGACUCGCUCCUCUUACUGGUCCUCA

GCACCCUUCAGCGGAGUGAAGCAGGAGCAGCUGUCCCCACGGGGCCAGGCUGGGCCACCG

GAGAGCCUGGGGGUGCCCACAGCCCAGGAGGCGUCCGUGCUGAGAGGUGAGGGCCCUUCU

GCCCUGGGCCCCCAGCAUCUGCCCCUGUUCCUCGGGUGCCCAAAGGGCCCUUUAUUCAAA

ACCUGCUGGGAAGUGUCAUCUCCCAGAGGGUCUGGGGGCCCCUCUUCACAGCUGUCUCCC

UCACCAUUUAGGGGUAGGGGGACUCCCUCUGCGUUGAAACAACUAGCACAACUUGUGGGU

CUCUCAGUCAGGGUGGCUCUCAGGACUGGGGCCUGGGGAAGGAAGGGCCACAGACACGCC

CUCAGCCCCACAUCUUGUGUCUUCCAGGGACAGCUCUGGGCUCAGUUCCGGGCGGAAGCA

UCACCAAAGGCAUUCCCAGCACACGGGUGCCCUCGGACAGCGCCAUCACAUACCGCGGCU

CCAUCACCCACGUAGGUGUCCUGGGGUGCGGCAGGAAGGACGGUUGGGACCAGCACGGGG

CCAGCCCAUCUUAUAACCCCUCAUUGUCACCAAACCAUUAGCCUCAGCCGAUGGGGUGAU

GACUGGGGGACAGCAGUUCACUCCAUGAACUUUCAUCUAGUCUGCAUGGAAUCAGGCACU

GGGAUUGCCAAGCACAGCUCAAAACUCUGCCUUCAGGGAGCUGCCAUGUAGUGGAAGGAA

ACAGACCAGGGUAAAUAAGUAGGCUGGUGAAAACCGAGCAGGCAGGCAGAAUGUGAGCGU

ACCGCGGCUGAGAAUGCCUGGCCACGGACAUUGCUUAGACAGGUGAGGAAGGUGACAUGU

CAGCUGUGGCCAGAAGAAAUUGAGGGUGCAAGCCAUGCGGGAAUCUCAGGGAACAGCAUU

CUAGGCUCAGGGAACAGCAUGUGCAAAGACAGAGCAUGAGAGGGUUCCUGAAUCGUGCCA

GGACUGGUGCCCCAGCCUUGCAGCUGGUGAGCUGUGGACCCAAGGUUUUGUGCCACGUGU

GUCUGGCUCUAGAACACAGUGACAGAGCGUGGGUCCACACACUUGCACACGUGCAUGCAU

GUUCUAACAGUGGAGCUUCGGGCAACAAGAUGGUCCACAGGGAGGUGUUUUUCACUCAGG

CAGCGUCAAAUCCCACCACAGAGGAGCCAUUUACACUUUGCCCUUCUCUGAAAUCUGCAG

GUUUUAAAAAUCUGAGCUUUCAAAGGCAGCUGUGCUGAGUGGGAAGGGCAAGUGCUUUGG

ACAAAAGUCAGGCUGAGGGCCCUCGGGUGCCAGCUCCACCACCUACUCAGACAGGCAGCG

GCCUGCCCUGACUCCGUUUCCCCUUUGCUAACAGUAUUCCUGGGAGAGUCAUUCUCCUGA

AGUCGUAGGGACCUUUGAAGUUCCCAGGCAUCCACCUGCUCUGGGUUCCUGAGUGGCCAU

GACCCGCGCCCUUCCUCGAACCUCCCAGUUACACACAUCUCUUUGAUUGCUUUUGGCAGA

CAGCCAGCUGCCAAAACAUGUGAGAUAGGCAAGGGUCAAACUUCCGUGCAGUCUCACUAC

CAGAUAGUGAGGAAGUCAGCGUGUGGCCUGCACGCACAGAGCCGUUUAUUAACUGAGUAG

GUGCCAUAGGCAGGGACCACGUGGUUCCCAGCUGGCAAGGGAGGUCAAGGAAGUCCCAGC

UCCCUGAUCCUCCAGGGACUUCCUGCCCAUCUUGGGAGUAGAGUUCAUUGGGGCCAAGAG

CAAGAGGCUACAGGCUCAUCUCCUGGAAGCAGACGUUAUGCAAACAGAAGCUUCCCUGUU

AAUGGGAGUUUGUCCAGUGUAUUCUCCCAAGAGCAGGGGCCACCGGCUGGAUUUCUGUUC

UCUGUCCAUGUCCCUGAAGAUGGCACCUCUAAAAUACUAUAGCUCAAAAACAUCAAGCUG

GACACGGUGGCUCUUGCCUGUCAACCCAGCAUUUUGGGAGGCCAAGGUGGGAGGAUAGCU

UGAGCCCAGGAGUUUGAGACCAGCCUGGACAAAAAGAGGCCUGUCUCUACAGAAAAUUUA

AAAAUUGGCUGGGCAUGGUGGCACAUACCUGUAGUCUCAGCUACUUGGGACUGGGAGGCA

GGAAGAUCACUUGAGCCCAGGAGUUUGAGGCUGCAGUGAACUAUGACAGUGUUCUGCACU

CCAGCCUGGGCAAUACAGCAAGACCCCUUCUCAAAAAAAAAAAAUCAGAUGCCUCAGUUU

CACCAUGGAGAGCCCCCCUUUUUAAAUCAUUUAUUGUGGUGUUCAGUAAGCCACUGAUUU

CUGCAGUACAAACCCAGUUCUGACAUGGACCAUCUGAUGUUAGGGCUGGAUACAGAAAGG

GGUGCACUUGUGUUAGCUGCCUCUUGGGGGGCUUUUCCCGCCAGGAUGCUGCAGGAAUCC

UGUGCUUUCCUGAUGGGAGAGGUGGCUGCCCCGAGCUGUCACCUGACUCAGCACCUAUUG

UGCCACACUGUUAGUGUACAGGUGUCUUAAAGCACAGCAGGGAGAUGCUGCUCAGAGUAU

UUGCUUUGGGAAGUUUGGGGGGAGCUCAUCAGAAUUCAGGGCGUCUUGCUGUUGCCCUCC

GCAAAGACCAGGAUCUGCGGUGAACUCCCCGGGGUACCAGGUGCUGCCCUCUGCCAGGGG

AUGUCCCCAGCCAAGCAAGUCCAGCCAGAGACUCAGAGCUCACUGGUCCAGGUCUUGGGA

UAUAGUAGGACCUUUACCGUUGAAUCUGCUCCUGGAACCCUAGAAAGAGAAGAGGGAUAU

UGAGAUUUGGGGACCCGUCGUCUGUGCCAGAUGCCUUUGAGAAUCAUCAAUGCAGAAGGU

CUCCAUUUGUGGAUGUGCAAACUGAGGCUCAGAAAGGAGGUCCCACGGCAGCUUGGUAGU

AAAGUUGCUGUUUGACACCCAACAUUCUGCUUCCAAAGUCAUAUUCUAACUCUGAUGCUU

GUGUUCUUGAAAGUCACCCAAGGCAGGAUGCUGCCCCACGUGGCCAUCUCCUCUCUGCUU

GAACACAUCCUCCAACGGGAAGCUCAUUCCCUAUAUGGCAGUGGUUCUCAAUUGGGGGAA

AUUGUGCCUCCAACUCUCUAGGGACAUUUUCGAUUGUCCUAACUUGGUGGGGGACACACU

GGGGAGGCGUGUACUCCUGGCUUCUAGUGCUUUGAAGCCAGGGAUGCUGUUAACAGCCCA

CAGUGCACAGGACAGCCCCACAGCAAAGAAGGGUCCAGCUCCAGCUGUCAGGAGGGCCGA

GGUGGAAAACCUGGGUUAGAACUAAAAUUUCCGGUGUGCUGCCCUGACGUGAGUCCUUGU

CCUGGUUUUAGGAAACCAAAGUGCAUGACGUGGUCACGGGUACAGCACAGGAGCAGAAAC

CCCAGCGUCCCCGCCAGUUACCGUUUUCGGUAACUGAAUGUCAAGGCUCUGAGUAGACCC

CACGCAGUGGUGGGGACACAGACUCCAGGACCAGAAUGCCUGGGUUCAAGUCCCCGCCUG

CCCCUUAUUAGCCAGGUGACCCCGGGUAAAGUCACUGUGCCUCCCUGUGCCUCGGUUUCC

CCAUCUGAAACAGGCAUAAUCAAUAGGGUUGUCUUAGGGUUGUUUCGAGGAUUAAAUGAG

CAAAUCCAUAGAGAGCACCCAGAACAGCGUCCACUCAUGGGAAGCACUUGACAAGGGAUC

UUCAUUCUUCAGGUUCCUCAUAGGGUUUUGUUCCAUGCAAACUCUUACCUAUUUGAGACA

GUGUGUGUGUGGACACGCGUGUGCAUCGGUGGGCACAUGGGCUUUUAAGCACGUCUUUGC

CUGCAUUUGAGUUGAGAGGGGUCCUGGGCUGCAGCCUCCUGGGCGCUCACCCCUCUGCAC

CUGCAGGGCACGCCAGCUGACGUCCUGUACAAGGGCACCAUCACCAGGAUCAUCGGCGAG

GACAGCCCGAGUCGCUUGGACCGCGGCCGGGAGGACAGCCUGCCCAAGGGCCACGUCAUC

UACGAAGGCAAGAAGGGCCACGUCUUGUCCUAUGAGGGUGAGUCGCAGGAGGAGAGGAGG

CCCAGGACCAGGGGAGGAGUGUGCUUGGCCCACUGAGGUAGCUUCACAGGGAGGCAGGGC

UGGAUUGACAUCAGAAAGCACAAUCUGAUAGGUGGUGACCUCCUUAUCCCUGCAGGUAUG

CAAGCCAGCAGCAGGGAAGCGUUGGCCUUAGCUGCCUCCCACCUCUGCCCAGUUCUUUAC

AGUUUAGAAAACAAACUCAUGGCCAACCUUUUUAGAAGCAUAGGAGGGAAACUGAGGCCC

GGAACAGAAGCCCGAGCUCACGCCGCCAGGCCUCCAGCACCGUACUGACAAACCACGCAC

UCUCUCAUUGGCCAUGAAAGAGGCCAUGGCCAGAGUGCCCCUCGCCCCACUGUGUCCCAG

GCUCUUGCUGCGGAGCCCCCAUCCUCUCCCUCUCUAGGCUCUGGGUUCCAGAACGAGGAG

ACCCUGCCAGGAAGGAGUUAAGGGAAUCGAGUGCCGGGAAAGAGAAUUUCCUGGCAGCCU

AGGGCACCCAGGGGUGUGGAGAUGAAAGCUGCUAAUGGGCGCCUCUCUCAGCACUGCAGC

UGCGAGGCCCGGAAUUGCCUCUCCUCCAUCCACUUCCGCCUGUGCCCGCAGCCCCCUCCC

CAGGCCUGGGAGGUGGAGGUGGCACCGUGUGGCUUAGGAACAUAAUGCACUCCCUGCUGC

CACAGAGAUAGCCUUGGAGACAGGCCUGCAGCUGUGUCUUGGGUGCCAGCUCAUGCCCUG

GUGCCCCUGGACCGAGUGCCCUGGGGGUGGCGGGAAGCCUGGGAAGGGCUGGUGGUGGGG

UUAGUCAAGAGCUUGUCUUGAGAGGUCACUGGGUAGAGUCCCACCUUGGGACCCCAGACC

AGUGCCUGAGCCUUUAUAGGCCUUCAGCGUAUCGUCUUCAUCAUGGGUUUCAGUCGGGGC

CUUUAAACUCUCGUCUGCUCCCUGGGCCAGGUAGGCAGUGCAGGCAGCGGCAGGUGUGAG

ACUGUAGGGAGUGGGGAGGACUGUAGGGAAUGGGGAGGACUGUGGUGCCUGCUCACGCCG

UCCACUCCCCUGCGGCCACCGGUCAGCCAGGUUUCUCAUCUCCGUUUUUAUCUGAAAUCU

CCCGAUGUUUAAACAUCGGCGAUUAAUUUGGAACGUUUUCUGAACAGCAACCUAGUACCC

UCCUGUUGGCAACCCCUGGAGUAGCUCACGGGCCGUGGGCCACACGAAGCAAUGGUUGAA

AAGCCCGAGAGCCUGUCAGUUGCUCAUUCCCUCUGAGGGGUGGGGCGGGGGCUCCCGGGG

CUCAUUUCUGAUAGCUCUGGACUCGGCUGCCCUGGAAGGAGAGCCCUGGCUAGAUGGGCA

AAGCCCAGCCUUUACCUUCGGGGGCCACCUCCGUCUGUUCACCUCUCUGCCUGUCCGGGG

AGCAGUGAGCCGGGCCCAUGUAGGCUCCUUUGGCCUGGCGAGGCCACCCCUGCCACCCCU

CACCACUGCCUGCAACACACACCUCUCCGUGCACACGCAGACUUGUGGUCGGACACUCAC

AUGCACAUCGGCACAGGUUUCUGGGUGUGACACGUGUAUACAUACAAAGCCGUGUACUGC

CUGCACCCUGGUACAUGUGUGUACAUGGACCCACUUAGUUCUCAGCAGCCAGGCUCACGU

GCAUGUGCCCACAUCCACUCCUGCACACACAAGACCCGUGCCAGUGCACAUGCGUGCUCC

UGCCUGCCACACGUCCACACACUGCUGAUGUAUCGGUGCACACGCGUGCUCCUGCCUGCC

CGCACCUCCACUCUGCUGUAUCGGUGCACGCGUGCUGCUGCCUGCCACACAUCCACACUC

UGCUGCUGAUGUAUCGGUGCACGUGUGCUCCUGCCUGCCACACAUCCACACACUGCUGAU

GCGUCCUUGCUCACGUGCAUGUGUGUGCACUUGUUCACGCCCCAUGUCGGCACCCGUGGG

UGUGGACACAGACUCACGUGCUCAUGUGGUCACAGGCACACCCUUGCUUGCAGACAGAGC

ACCCUGGAGGGCUAGGGUACAGGGUGCAGGCAGCGUGCCCUGCAUCUCCCACCGUGCAAC

CCCCUGGAAAAGCUCCUGGGUCCUGCUGGCAGGCCCCCAGGGCCUGCAGGCUGCCAGCUC

CCUCUGGAGGCCUCGGCUGUGAGGCUUUGUGACGGGGCCAGCAUGGAAGCACUGCUGGCU

CCUGCCUACCGGCUCUGCCUGUCCUGCCUGCCCACCGUGGUCCUGGGCCCGUGCCCAGCU

CCUCACCGAGUGCUUUGUGUGGUUUCCAGGUGGCAUGUCUGUGACCCAGUGCUCCAAGGA

GGACGGCAGAAGCAGCUCAGGACCCCCCCAUGAGACGGCCGCCCCCAAGCGCACCUAUGA

CAUGAUGGAGGGCCGCGUGGGCAGAGCCAUCUCCUCAGCCAGCAUCGAAGGUGAUAGCAG

GGAGGAGACUUCAUCUCUCGGUGCCCCCUGGUGGGCGGUGGGGGGAUGGCUGACCCCGUU

UUACAGAUGGGGAAACCGAGGCUGGGCUUUCUGAGGCUCCAUCUGGAGGUAGCGCAGGGA

CCUUCCCGUGCUGGGUCCUACUCCACCAUCAUCGUGGGGAUGACCACUGGCGGCUGUAAA

CACUGACCCCUGUCACUGCCCAGUGUCGGCUCAGGGAGCCACGGAAUGAUGGCCUCACCC

UCUCCUACCCAGGUCUCAUGGGCCGUGCCAUCCCGCCGGAGCGACACAGCCCCCACCACC

UCAAAGAGCAGCACCACAUCCGCGGGUCCAUCACACAAGGUACUGCCCUGUUCCCUGCUC

CCUCGUUGCCCCCAACGGGUGUACAGUCACGCAGGGCGCGGGAGGGAGAGACACAGCCAG

AGUGUGGUGGGAACUCAGGACAAGUAUGCAGAAAGGCCUGCAGCACACAUGUACAUGAUC

AGUACGUGAGCUACGGAGCAAGGGUGUCUCUUACUUAUUUCAAAACAAAAACAAAAAGCA

AAAUACCACCGAUCACCCCUGUGCUUUUAGGUGUCACAUAGCAACUGUCCUGUGCUUGGC

ACUAACCCAGGUGCCACCUGCGUAUCGUUUUACAGAACAUCCUGGUGAGGCACAUGCGAU

UGGGAGAGGCUUGGAGAGCUCCGAGAACUCUUUCAGGUUCUCGCGGCUGGUCCAUGGCAC

AGCCAGCUACUGUGAACUUGGCAGCUUUGUGGGUUUUAUUUUUUAUUUUUUUAUUUUGUU

GUUGUUGUUGCUGUUUGAGACAGGGUCUCAUUCUGUUGCCCAGGCUGGAGUGCAGUGGCA

CGAUCUCAGCUCACUGCAGCCUCCGCCUUUCAGGCUUAAACAAUCCUCCCACUUCAGCCU

CCCAAGUAGCUGGGACCACAGAUGCACACCACCACAUCUAGCUAAUUUUUGUAUUUUUGU

AGAGGUGGGGUUUCACCAUGUUGCCCAGGCUGGUCUCGAACUCCUGAGCUCAAGCUGUCU

GCCUGCCGCAGCCCCCCAGAGUGUUGGGAUUACAGGCGUGAGCUACUGCACCCAGCCUGU

GGUUUUAGCUUCAUGAUUUCAUAGUGUUCCCGACUUGCUGAGGUGGUUCAGUUAAUAUUC

UUGUUUUAUGUGUGAAGAAGCUGAGGCCCAGAGAGGUCAGAUUUCCUGGUCAAGGUCACA

CAGCAAGUGGGGAUUUGAACUCAGGCAGACUAGCUCCAGAACCCACUGGUGUGGAGGCUC

UUGAUGGGUCUGGGUGGGGCGGGGCGUGAGGGUCAGUGCUGUCGGCCCGGCAGGGAUCCC

UCGGUCCUACGUGGAGGCACAGGAGGACUACCUGCGUCGGGAGGCCAAGCUCCUAAAGCG

GGAGGGCACGCCUCCGCCCCCACCGCCCUCACGGGACCUGACCGAGGCCUACAAGACGCA

GGCCCUGGGCCCCCUGAAGCUGAAGCCGGCCCAUGAGGGCCUGGUGGCCACGGUGAAGGA

GGCGGGCCGCUCCAUCCAUGAGAUCCCGCGCGAGGAGCUGCGGCACACGCCCGAGCUGCC

CCUGGCCCCGCGGCCGCUCAAGGAGGGCUCCAUCACGCAGGUAUGGCCCAGGGCCAGGCA

CACGGGCCCAGUUCUAGGAGGGGUGGCGGUGGCUGUGGGGCACUGCCCUGGGCCUCUCCA

CAUGGGGAAACCGAGGCUGAGAGCCCUCGCGUACCUUACAGUCACCCAGCUGCUCAUCAC

CGGGCCUCAGCUGUGCGUGUUCCAGGGCUGCGCAGGGGGCACCAGGCUCCUGACCUGAUU

CUACUGAACUCACAUUGUUCCCAUUCUUCAGGGAGGGAAACUGAGUCCCAGAGAGGCCAG

GCAGGCUUCCAAGGCCACAGGACUAAACAUAGUGACGAGUAACUGCCUCCGUUGAAUCUU

UGUGAGGGUCCAGGUGCGGCCUGAGGAUGUUGCAUGCGUUCAUUGUUUCACCCCCUAGCA

AUGCUCUGAGGUCGUUUUCUUAAUGACCUUAUUUUAUUGCUGAGUAAAUUGAGGUUCAGA

GAGGUUCAACGACUCACCCAGAGUCACGCAGCAAAUGCAGUUGUGAAACCCAAAUUCAGA

UGUUCCUACAGCCGCAGCAUCCACUGCACCCACCAGCAGGUUGCACCACAAGAGGCCCCA

GUCCCCCCAGGCGGCCCCAGCUCAGUAGGGGAAGUUCCGUGCCGAUGGUACGAGGACGAG

GAGCUGUUCGGUGGAAAGCCCCUGAAGGCCACUGUCCUUCCACAUGGGCAGAGGUGGCCU

CUUGUGAAGGGGAAGGAGAAUGGGAGCCACCACGGGGCUGUGGGGCUGUGAGGCGGAAGG

ACUGGGGUGGGUGUCCCGGGAGGGGUUCCAGCUUGUAGGAAGGUUUUGAAGCCAGGGAGA

AGGCAGAAGCAGUAAGAUCCCUGAUUGCCAGGGGAAGGGUUUGGCUCUCAGCCCCUAGGC

AAUUAUGGAGUCCUUGGAAGCAUCCACCGCAUGACCAAGACAGGGUCCAGAUUCUAGAAU

AUUCUUUUGAAAAACAAGGGCAGUUCCCCUUCUUACGACAGUAAUGAAGACAUCCCUAAA

UAGAGUUUUGUUGCUUGCAAAGCCCUAAAGUCCCACCAUAUCAAGUGUCUCCGAAGCCUG

CUGAAAAGAGGCAGGGGACCUGGUGGCCUGGCUACGAAGGUCCCAGUCUGGACUGUGACC

CCCCCAUUUCCUCACCAUCCUUUCUGUCUGGAGGGCAAACACCUCAGCCCUGACCUCAGU

GUCCCUGGGGCUGAAAGCCUCAGGGCGGGUAGUAUUGGGUCUGGGUGCUGACUUUUUCUG

CUUGGCAUUGGGUGGGCCAUGGAGGGUCCCAGGCUGAACAGAGGAAUGUUUUUUACCCAC

AUGAGGGUGUUGGGCUUCUUUCUCGCAAACUCCAGGGACCAUCAGAGAGCCCACCACUCG

CGGCAGGGAGAGUUGACUGUUGAACUUUUUACCCCUUUCUGCAGUCCCCCCAGGGAGCGU

GGGGACCAGGGUCAGGCCCAGGGUGCGCAGGGCAGUAAGUAACAAGUGUGCCAUCUCAGG

GUUAGCAAAGCCCUCUGUCUCCCUGCCUCUGGAGGCAUCAGAUGUCACUUCCAUCUUAUA

GAUGACAAACUUUUUGAGGCUCAGAAGGGGGAUGCAGCUGGUCUGGGCUAUGGCUGUGGC

CAGGGCUAGAGCUUACAUCCCCUCUGCCCCAGGGCACCCCGCUCAAGUACGACACCGGCG

CGUCCACCACUGGCUCCAAAAAGCACGACGUACGCUCCCUCAUCGGCAGCCCCGGCCGGA

CGUUCCCACCCGUGCACCCGCUGGAUGUGAUGGCCGACGCCCGGGCACUGGAACGUGCCU

GCUACGAGGAGAGCCUGAAGAGCCGGCCAGGGACCGCCAGCAGCUCGGGGGGCUCCAUUG

CGCGCGGCGCCCCGGUCAUUGUGCCUGAGCUGGGUAAGCCGCGGCAGAGCCCCCUGACCU

AUGAGGACCACGGGGCACCCUUUGCCGGCCACCUCCCACGAGGUUCGCCCGUGACCACGC

GGGAGCCCACGCCGCGCCUGCAGGAGGGUGAGUGGGGUGUGCAUGGGCGUGAGUGGGGUG

GGCGCCUGUCUGGAGAAGCUGUGCCUCCCCAUCCACCAUUAGCUUAGUUUGCACCUGGGA

UAUCCUCGCCACCCGCUUUCCACCACAUCCAAACCACCUGCAGGCCCGUGGGCUCUGCCU

CCGAUUCCAAACCCUGUCCAACUCCUUGCCACCUCCCAGACCACCGUGGUGUCUCACCUA

GCUUCCCCCACGCCCCUCCCUCUUCCUGCUGUAAUCCACUCUGCAAACAGCUACCCGGAU

ACUUUCUAAAAAUGCAAAUCAUAUUAUUCCACUUCCCUGCUUCCAUCCUUCUAGCAACUU

CACACAUUUUGCUAUGGCCUUGGGGCGCCUGCCUGUUGGGGCCCUGCCUGCCUCUCAUUC

AGCCGGAUUCCUUCGUCCUCCCCAGCCCCAGCCCCUGGGCCCUCUUUCUCUUUGUUCCCU

GGCCAUGCUUAGCUCGGUCAAUUCAGUAUUUGCUGGGGGCCUUUGCGUGGCUCCUCCUCU

CUGCCUGCCAUGUCCCCGCCUUCCAGAUCUUUACUUAGUGGGUUUCUUUCCAUCCCUCAG

GUCUUUGUUUACAUAUUACAUCCUUGGGGAGGCUUCUAACCAGACCCCCUAUCUCCAGUU

CAUAUCACAUGCUGUGACAUUUUAAAAUUGUCUUCCGGCCAGGCAUGGUGGCUCACACCU

GUAAUCCCAGCAGUUUGGGAGGUCAAGGCAGGCAGAUCACCUGAGGUCAGGAGUUCAAGA

CCAGCCUGGUCAACAUGGUGAAACCCUGUCUCUACUAAAAAUACAAAAAAAUAACCGGGU

GUGGUGGUACGCACCUGUAUUCCCAGCUACUCGGGAGGCUGAGGCAGGAGAAUCACUUGA

GCCUGGGAGGCAGAGGUUACAGUGAACGGAGAUCGUGCCAUUGCACUCCAGCCUGGGCAA

CAAGAGUGAAACUCUUAUCUCAAAAAAAAAAAAAAAUGAAAGAAAAUUUUCUUCUGAGCG

UGUUUCACUCUGUAAUUCUCAUUUGUUUGCUAGUUUAUCACCUGUCUCUCGCAUUGAAUG

UCAGCUUGUGAGGGCUGGGAUUUCUGUUUCGUUCACUGGGGUGACCCCAGUUCUCACAAC

AAUGCUUGCCACGUAGUAGAGGCUGCAUCAAUAUUUUUUAAUUGAUUGAGUGAGUGAAUG

GAUGAAAGAAUGAAUUUUUUAAAAACUAUAACACAAAAGCAAAUGAGUCAGUGAGCAAAA

AGUGAACUAAGGCAAUGAAGAAAUGAAGGAGUGAAUGAAGAGACCUGGUCCUUGGGAUCC

CGAGGUCCCUAUCCUCAAACAACUCCCCGUAAAUGCCAGCCCCAGAGGCCCGAUGCAUCC

ACCUUGCCCGUCCACAGGCAGCCUUUCGUCCAGCAAGGCAUCCCAGGACCGAAAGCUGAC

GUCGACGCCUCGUGAGAUCGCCAAGUCCCCGCACAGCACCGUGCCCGAGCACCACCCACA

CCCCAUCUCGCCCUAUGAGCACCUGCUUCGGGGCGUGAGUGGCGUGGACCUGUAUCGCAG

CCACAUCCCCCUGGCCUUCGACCCCACCUCCAUACCCCGCGGCAUCCCUCUGGACGCAGG

UGAUUGCCCUGGGGCUCCCAGAACCCUGCAGUGGUGCUGAACAGGGCCACGGACCUCAUC

AGUGUUCGCUCAGGGACUCCUUAGGCAUCAACUGUCAGGUUCCCCUGGAUGGCGAAACUG

AGGCCUCGGGAUUGGAAGACCCAACAGUGUAAUCAUGAGCUUAGGUUGGAGCAGAAUUUC

UCUUAGUAGUUUGCAGGACAUGUGGGGUUAAACAUUUCAGUGGUUUUCUUUUCCGGCAGG

ACUUAUCAGUGCCUUUAGCAAUGCAAAGGUAUAGAAUGAGGACUUGAGUAUAUGCAUUUU

UCAAAUAGACAUGAUCUGAAAGUCUUUUUUAAAAGUUGCCGGGCACGGUGGCUCACACCU

GUAAUCCCAGCACUUUGGGAGGCCGAGGCAGGCGGAUCACAAGGUCAGGAGAUAGAGACC

AUCCUGGCUAACACGGUGAAACCCCGUCCCUACUAAAAAUACAAAAACUAGCCGGGUGUG

GUGGCGGGCGCCUGUAGUCCCAGCUACUCGGGAGGCUGAGGCAGGAGAAUGGCGUGAACC

CGGGAGGCGGAGCUUGCAGUGAGCCAAGAUCGCGCCACUGCACUCCAGCCUGGGCGACAG

AGCGAGACUCCUUCUCUAAAAAUAAAAGAAAUUAAAAAAAAAGAAAUAAAAAAAGUUGCA

UCCCUUUGGAGUGUUAAUCUGCAUUGGGAUGUCCUAUGUUUGGGACAACUUUGAUGCAAA

AAGCAUCCUUCGUAGAAGUCACCCUCUUGUGUCCUGGCGUGAUGUUUUCCUGCUGUCCGA

CGCUCAGUUCUGGUUUGUGCUUUGGGCAGCCACACAUGUAGGUGGGAGAAGCUGUCCGGG

UGCAGAAGUAGGGGGCAUCCAGACAGGUGGAGCGACACCAUCAGGCCUAGGUAUGGCUGG

CCUCACAUGAGCUCCCCUCUGCCCCGCAGCCGCUGCCUACUACCUGCCCCGACACCUGGC

CCCCAACCCCACCUACCCGCACCUGUACCCACCCUACCUCAUCCGCGGCUACCCCGACAC

GGCGGCGCUGGAGAACCGGCAGACCAUCAUCAAUGACUACAUCACCUCGCAGCAGAUGCA

CCACAACGCGGCCACCGCCAUGGCCCAGCGAGCUGAUAUGCUGAGGGGCCUCUCGCCCCG

CGAGUCCUCGCUGGCACUCAACUACGCUGCGGGUCCCCGAGGUGAGUGGGUGGGCAGACC

ACCUCCGCUGGGUUUGGCCUUAUUCCCAAAGGACAUGGGCGUGCCCCUGUGGCCUCGCGG

AGGCAGCUAGACCUGGUCACCUUGUGGGUCACCUUGUGUGAACGGACCUGAGUGGGUGGC

CUGGGGUUGUGCGUGCUGUGGGUGCUGGUUGGCAUCUGGUAGGUGAGUGCACAGCGUGUG

GCUCCUGGCUGCAUCCUCAGUGGGUGUGCGUGCAUCUGUGUAUACUCUUAGGAUACAGGG

GCCUCAGGAGUUUAAAGAUCAAAAUGUGGCCGGGCACAGUGGCUCAUGCCUGUAAUCCCA

GCACUUGGGGAGGGCGAGGCAGGUGGAUAACAAGGUCAGAAGUUCGAGACCAGUCUGACC

AACAUGGUGAAACCCGUCUCUCCUAAAAAUACAAAAAUUAGCCAGGCAUGGUGAUGCGCA

CCUGUAGUCCCAGCUACUCACUAGGCUGAGGCAAGAGAAUCACUUGAACCCAGGAGGUGG

AGGUUACAGUGAGUGGAGAUUUUACCAUUGCACUCCAGCCUGGGCAACAGGGCAAGACUG

UGUCUCGAAAAAAAAAAAAAGAUGAAAAUGUGAGGCUGUUUGGAGUUUGUUCCUUUGCCU

UGUAAACAGCCCACAGCUGCUUUGCGUGCACACGUUCCAGGGCCAUCCUCAGAAAUGCUU

CUGGAAUAACCAAGUUCUAGCUGGGGCUCAGCUGGAAAAGCUGAAGUCACACUUAAGUAU

UUUGAACAGUGAGGAUUGAAUACAGGGAAUGGAUUGUGUAGGCGUCAGAGGCUGAAGGGG

CACAGAGGGCCUGAGAUGGGAACCAGUGAGGGCAGCUGCAGGAGAUGCCCCGGCUCGGGC

UUGGGAGCAGAAGAGGAGGUGGUACCGAGAGAACCUGAGCAUUCAGAAAAGGGUUCCAUG

GCUGGUGCUGGGAGCCGAGGAGGGAGUGCCUGCCACCAGCUCUGCUGGCUCCAGGAGUGU

GUGCCGUGCUCUCCAGGAGGGUGAUCUGGCCGGUGGGCAGCCUGGCCUCUCUCCUCCCUG

UGGCUACAGCCCUGGCCCAACACCUCCCGCAGGCAUCAUCGACCUGUCCCAAGUGCCACA

CCUGCCUGUGCUCGUGCCCCCGACACCAGGCACCCCAGCCACCGCCAUGGACCGCCUUGC

CUACCUCCCCACCGCGCCCCAGCCCUUCAGCAGCCGCCACAGCAGCUCCCCACUCUCCCC

AGGUAGCGCCACUGCCCAGUCUGGGGUGGGGACCCCGGCAUCCAUGGGAGGCGGCUGGGG

GAUGGGCGGGCAGAAGCCCUGCUCUCUUUCCCACCCCAGAAGACAAAGCCAGGCUCUUCU

UCGGCCCUGGGGCUGAGUCUCUGGCCUUUGGGUUUCCUAGGAGGUCCAACACACUUGACA

AAACCAACCACCACGUCCUCGUCCGAGCGGGAGCGAGACCGGGAUCGAGAGCGGGACCGG

GAUCGGGAGCGGGAAAAGUCCAUCCUCACGUCCACCACGACGGUGGAGCACGCACCCAUC

UGGAGACCUGGUAGGGCAUCAGAGCCCCCACCCCCCGCUCCGGGACUCCUUGUGGGCCGC

AAGAGGCCUCCCCCUGCUGAUGCCACUGACUGUCACCAGGUACAGAGCAGAGCAGCGGCA

GCAGCGGCGGGGGUGGGGGCAGCAGCAGCCGCCCCGCCUCCCACUCCCAUGCCCACCAGC

ACUCGCCCAUCUCCCCUCGGACCCAGGAUGCCCUCCAGCAGAGACCCAGUGUGCUUCACA

ACACAGGCAUGAAGGGUAUCAUCACCGCUGUGGAGCCCAGCACGCCCACGGUCCUGAGGU

GGGCCAGGUUGGCAUGGGGGAGGGGGCGGGCAGGUGGAUGGGUGGUCAGUAGGAGGAUGA

GCAGAUAAGAGGAUGCUUGGUGGGAGGUAUAUGGGAGGUGGGUGGGUGGGCAGAUGUGUG

GGGGUAGAAGAAUAGACUAUGUGUGAGUAGUGGAUGGGUGGGUGGUCGGGUUAGGUGGGU

GUGUGGGUCAGUGGGAAGUUAGGUUAGCUGCGUGGGUGGAUGGAUGGAUGGAUGGGGGGG

UGGUCGGGUUAGGUGGGUGGGUGGGUCAGUGGAAAGAUAGAUGGCUGGGUUAGCUGGUUG

GAUGGGUGGAUGAUCAGGUUUGGUAGGUAGGUGGGUGGGUGGAAGGAUAGAUGGCCGGGU

UAGCCAGAUAGGUGGUUGGGUUAGGUGGGUAGGUGGGUCAGUGGGAGGAUAGAUGGUUAG

GUUAGCUGGGUGGGUGGAUGGAUGGAUGGGUGGGUGAUAGGGUUAGGUAGGUAGGUGGGU

GGGUGAGAGGAUAGAUAGCUGGGUUAGCUGGGUGGAUGGAUGGGUGGGUGGUCACGUUAG

GUAGGUAAGUAGAUGGGCAAAAGGAUAGAUGGCCAAGUUAGCUGUGUGGGUGGGUGGGUG

GUAGGUAGGUAGGUAGGUAGGUAGGUAGGUAGGUAGGUGGCUAAGAGGAUAAAUGACUGA

GUUAGCCAGAUGGAUGGAUGGAUGGAUGGAUGGAUGGAUGGAUGGGCAGGUGGUUGGGUU

AGGUAGGUAGGUGGGUGGGUGAGAGGAUAGAUGACUGGCUUAGCUGGGUGGGUGGGUGGG

UGGGUGGUAGGUAGAUCGGGUUAGGUAGGUAGAUGGGUGGGUAAGAGGAUAGAUGGCUGG

GUUAACUGGGUGGAUGGAUGGAUGGGUGGGUGGGUGGUUGGGUUAAGUAGGUAGGUAGGU

GGGAGGAAGGAUAGAUGGCCAGAUUAGCCAUGUGGAUGGAUGGAUGGGUGGGUGGCUGAG

UUAGGUAGGUAGGUGGGUGGGUGAGAGUAUAGACGCUGGCUUAGCUGGGUGGGUGGAUGG

GUGGGUGGUAGGGUUAGGGAGGUAGGUGGGUGGGUGAGAGUAUAGAUGGCUGGGUUAGCU

GGGUGGGUGGGUGGAUGGGUGGGUGGUAGGGUUAGGUGGAUGAUGGAAUGGAUGGUUGGA

UGAGUAAUUGUGGGGAUGAGUGGAUGGAGGCCCCAGUGGAUGGACGAUGAGUUGGGCGGU

GGGAUGAGUAGAUGGGGAGGUUGUUUGGUUUGAGAAUAGAAUCUGUGGAGAGGGAGAGAC

UGAAUGGGGACUGUGAGGAAAGACUUUCCCGGUCCCCCACAUUUAGCAAGGCCAGCAAGG

AAAAGAGGUGUCCCUCUUGCCUGGACAAAGUCCCAAGUUUCUCUGAGAUGGGAGAGGCCC

CUGAGUGCCCUCUGGUGACACACACUCCAGAGACUGUGGGCAGAGCUGCUUCACCAGGGG

GGUGGGGUCACACCUCAACACCCUUCCCUGCCCGGAACCUUUUUUUUUUUUUUUUUUUGA

UGGAGUUGUACUCUGUCGCCCAGGCUGGAGUGCAGUGGUACGAUCUCUGCUUACUGCAAC

CUCCACCUCCUGGGUUCAAGUGAUUCUCCUGCCUCAGCCUCCCAAGUAGCUGCAGUUACA

GGCACGUGCAACCACACCCGGCUAAUUUUUAUAUUUUUAGUAGAAAUGCGGUUUCACCAC

GUUGGCCAGGCUGGUCUCAAACUUCUGACCUCAAGUGAUCUGCCUGCCUCAGCCUCCCAA

AGUGCCGGGAUUACAGGCGUGCGCCACCGCACUCAGCCUCCAGGACCUAUUUAGAGGCAA

CUUACUCCUCCAAAAUGAAUUUUCAUUCAAAAAAGUACUUGUGCCCCAACUUGCCGCUUG

GAUGCCUGAAGUCAUACCUCCCCUAAUCAUUAUUUCAAUUAUGAAAAAUGGCAUCGUUUC

CCAAAAGCAGUGGCGACUUUGUCACCCCUGACUCAGACCCAGCAGGUGCAUGAAGCACUA

AUCCCUGGCUGUGGGGAGCAGGGUUCCGCACACAGGCUCUGUGGGAUAUGCCCGUGACAG

CCGAUUCAAAAAUAUAAUGGAGAAAACGAGUGCCUGCCCACUGCCCGCUCAUGAAUGAGG

CUCUGGGAGGUAGAGUUAGCGAGAGAUGGGGGUGCCCCCAUGGAAACGCAGGGCUCCGGG

AAGGCCACGUGGGUGGAGCAGCCGGCUCCUGUUCCAGCCCCAGCCCUGUGCAGGGGCAGC

UGUGCCCCAGCUUCUCACUCACAGAGCUGGGAACAGCAGCUUUCUCUGUUCUCAACGCCC

ACCCUGGCCGCCCCCUGGCAUCCUUCCCCUUGCUCUCUCACUCCUCACGUGGCUUUUUUA

GCUCUUCGGGUAUCCUGAGCAGGCCCAGCUCCCUCAUGUGCCCCCUCCCUGAAGACCUCC

UAGCCCUGCCUCAGCUUCCUUGCCGUUCUCUCCUGGUACCUCAUUGACUACCUCCACACA

GUUACCAGGCUCUGUAGAGCUUGGGAUAUCUGUUAAUGUGAUCGUAGUCUGUUUUCUGAA

UUAGAUGGUAAGAUGCUUGCAUCCGUUUUAUGACCGCUGUAUUCACAGUGUCUAGAACAG

UGCCUGGCACCUAGUAGCUGCUUAAUCAGAAGUUUUGGAAUGAUGCAAGGAAUGAAUGAA

CGAAUGAGUGGGAUGGGUCAAACCAUGACGCACAGAGUCUGGCAGGUUACAGUCAGGAGG

GCAGUUUCACUCUGGGAGCAGCAGGGGAUGUGGAUUUAUCCCAGGGCAAUGGGGACUCAU

CGAGGGUGGUGGAGGAAGAGGGCAGCUCCCAUGACUGCCUGACCGCCUUCUCUCCUCCCC

CAGGUCCACCUCCACCUCCUCACCCGUUCGCCCGGCUGCCACAUUCCCACCUGCCACCCA

CUGCCCACUGGGCGGCACCCUCGAUGGGGUCUACCCUACCCUCAUGGAGCCCGUCUUGCU

GCCCAAGGAGGCCCCCCGGGUCGCCCGGCCAGAGCGGCCCCGAGCAGACACCGGCCAUGC

CUUCCUCGCCAAGCCCCCAGCCCGCUCCGGGCUGGAGCCCGCCUCCUCCCCCAGCAAGGG

CUCGGAGCCCCGGCCCCUAGUGCCUCCUGUCUCUGGCCACGCCACCAUCGCCCGCACCCC

UGCGAAGAACCUCGCACCUCACCACGCCAGCCCGGACCCGCCGGCGCCACCUGCCUCGGC

CUCGGACCCGCACCGGGAAAAGACUCAAAGUAAACCCUUUUCCAUCCAGGAACUGGAACU

CCGUUCUCUGGGUAAGACCACCCUGACAGCGGCCACCUUCAUAGACGCGAUUAUCAUGCG

UCAAAUUGCUCACGAUAAAGGGGCGCGAGAAGGAGGUGCGCUGGCCAACGGCUCCCCUCG

CGAUGGUAAGACUUCCGGCCCGCACCCACCCCGUCUCGUGGUCCAAAGAUAUUUUCAGAU

CUCUGCUUUUUACUUUGGCCCCCGUUUUUUUUGUUGUUGGUUUUGGUAUUUUGUUUUGAA

GCCCAUCCGUCCUCGCCGGUUUGCACGCGCUGACGACUACUCCGGCCGCGCCUGCCCCUC

UGGUUUGGGGGCGCUCAUCAUUUGCACAUCAUUUUACCAUGGUUUUUUUUUUUUGGAUUU

UUGCUUUUUUUUCUUUAAUGAAUGGAUCUGUGAUUCUGACUUCGACUGCGCCCCCAUCUC

CCUCUUUGCGCCUGUGUCCAGGGAGCAGGGAUGGGGCUGCGGGAGGGCUCGGGCCUACGC

CCCCACCUGCCGGCUGCCUGGAUGCUGUCGGACUGGGGGAAGUGGAGGCAGGCGGUGCAA

GGAGAAGCUGAGGCGGGGCAGGGACCUGCGCUGUCGAGGAGGAGCUGGGUCUGGCUCUUG

CAUCUUGCCCUGUCCCCAGCCCCUGUACCCCAGAAAAAGGGGAGCCCUCUGCCUCUGGAC

CCCUGCCUUGGCCCUAGUUCAUGGCUCCUCUCUGUUGGACUGGGAUGGCCGAGGCUAUAG

CCCAGGCGGGGCCCCGGGGACCCAGGGUCACUCCCAGCCACACCCCCACUUCUCACUCCG

CCCCACACACUCCUUCCCCAGAGACCCAUGCUGCCCCCAUCUCACGCUGGCCUCGCCCGG

CCUCCACCACCUGAACCCAUCUCUGUCCCUUCUUGCCUAAUCUCUCUCUGUGUCUCCCUC

UCUGUCUGUCUCUGUCCCGGGCUCUGCAUCUCUCUCCACCUCUCCCUUGGCCUCCCUGUC

UCUCCCCAACACCCCUCUCUGCCUUACUGUCUUUGGGAGCCCAAACCCUACCCCUAGCUU

GGGUUCCCCUUGACCCCCCCGGGGUCCCAGCCAGCUGGGAGGGCAGCCCUGCCCCUCGGG

CUCCGAAACCCUGGGCCCGGUGCCUGACUCUGCACCCCCCGCCUGCCCUAGGUUACCACG

GCAGCAGCUACAGCCCCGAAGGGGUGGAGCCCGUCAGCCCUGUGAGCUCACCCAGUCUGA

CCCACGACAAGGGGCUCCCCAAGCACCUGGAAGAGCUCGACAAGAGCCACCUGGAGGGGG

AGCUGCGGCCCAAGCAGCCAGGUACGCCCCACCCAGUACCCAGGCCCCCGAAGCCCUGCA

CAGUGAGGACCCUCAAGGCCCCAUCAUGCAGAUAGGAAAACAGAGGUGCCUAAAGGCCAA

GGAAUUGGCUGGAUCAUGAGGCUCAAAGGCAUGGGGCUGGGAUUUGGGCCCAGCAGCCCU

GGGGCCAGCAGACACCCCCUGACCAGCUCUGCUCUGCCUGCAGGCCCCGUGAAGCUUGGC

GGGGAGGCCGCCCACCUCCCACACCUGCGGCCGCUGCCUGAGAGCCAGCCCUCGUCCAGC

CCGCUGCUCCAGACCGCCCCAGGGGUCAAAGGUCACCAGCGGGUGGUCACCCUGGCCCAG

CACAUCAGUGUAACUACGCGUUCUCUGCUGCUGCUUGUCACCUUUGCACCUGGGGGCACC

AGGCCUGGAGAGGGGAUGGGGAACCCCACAGCCCUUCUGUCCUGGCGGGGUGGCUGGGGG

AUCCAGGGCAUGGCGCUGGGGGGAUCCAGGGCGUGGGUGAGGGUGAGAUCCCAAAGCCCC

GAGCACCGGCACCAUCACCGCCCCCUAAUCCAUGGGAGGAGCCUGUGAUGCGAGCCGAUG

GCAUCUUCACGGGCAAUGAGGCCUUCCUGGUGGCCCAGGUUUCUCAGUGUCAUGGGCUGG

UCUCAUCAGCCAUCUGCCAACUACCAGCUUGGGACCGCUGACCACAGCCCCACUCCCAUG

CACACUGGGACACGGAGGCCCAGAGGGUGGCGGGCAGGUCCACAGUCACCCAGGAAGCUG

GCCCCACCCAGGAUUCUGCCCCGAGCUCCGUCUAGCCCCUCCCCACCCCCAGAAGGUUCU

GUCAGGAGAGUGCUGCCUGACUCUGGGCCCCCCCACUUGCCUGCAGGAGGUCAUCACACA

GGACUACACCCGGCACCACCCACAGCAGCUCAGCGCACCCCUGCCCGCCCCCCUCUACUC

CUUCCCUGGGGCCAGCUGCCCCGUCCUGGACCUCCGCCGCCCACCCAGUGACCUCUACCU

CCCGCCCCCGGACCAUGGUGCCCCGGCCCGUGGCUCCCCCCACAGCGAAGGGGGCAAGAG

GUGAGCGAGGGAUGGGGGUGCCUCUUGCUGGUCAGCCGGCGGGAGGCCUUCGUCAGCUGC

CUGCCUCAGAGCUGGGUCUGAGCUCAGCUGCAUAUGAGGCCCGCCUGGUGCCAUUGCACA

GGCAAGAAAUGGAGGCUCCAGGAGAUGGGGGAGUCACAGAGCGGAUACAUAACGAAUAAU

AGCUGAUGAAAGUACAUGCUGUGCUCAGUGGGUUCCAGGCACUCACGUGUACCCUUUACA

CACAGCCCCCCAGGAGACAUAGGCAGCAUUCUCCCCAUUUCACAGAUGAAGAAACUAAGG

CCUGGAGAAGUGAAUUCACUUGCCCACAGGGGCACAGUCAGUAGGUGACAGAGGGGGAUU

UGUUCCAGGGCUGUAUGAUUAGAGAGCAGCCUUUCCACACAUGCACACCCACACACAUGC

GCACACACACACAUGCACACCCGCACACAUGCGCACCCGCACACACACACGUGCACACCC

ACACACAUGCGCGCGCACACACAUGCACACCCGCACACAUGCGCGCGCACACACACAUGC

ACACCCGCACACAUGCGCACACACACAUGCACACCCGCACACAGGCGCACCCGCACACCC

CUGUAGACACAGGCACAUGCACAUGCUCACGCACACUCCCACUCCCACCCCCGCCCCCAC

CCCCACCCUGGUGAACCGUGGGGCCUCUGGGGGUCAAAAGAGAAAGAGAGGGGAGGGCCC

UGAGCUCCAGGGUGAAGGAGGCGUUUUGGUGGGGGCGGGGGGGGUAAUGUGUGUGCGAGG

ACAGACAUGGCAAGACAGCAGGACAUCUUUGGGGGGCAGUGGUGUAGCUGGCACUGGGGU

ACAACCAGAAUUCAGAGCAGGGGUCAAUAAACUGUGGCCCAUGGGCCAGAUCUAGCCCGG

GCCCUCUGUUUGUACAAUUCAUGAACUAAAAAAAUGAUUUUACAUUUUUAAAGGGUUGUU

UAAAAAAAAAAUAAAAAUGAUAACGAUACAUGCCAGAGAUUACUUGUGGUCGAAAAUGCC

UAAAACGUUUAUGAUUUGGCCACGUACAGGAAAAGCGUGUGGGGCCCUGGUUUAGAGUGG

AGGGAGGGUGCGCCCCCUCAGUAGGGAGACCUCUGACCACAUCUGGGGCCCUUUCUCCAU

CCAGGUCUCCAGAGCCAAACAAGACGUCGGUCUUGGGUGGUGGUGAGGACGGUAUUGAAC

CUGUGUCCCCACCGGAGGGCAUGACGGAGCCAGGGCACUCCCGGAGUGCUGUGUACCCGC

UGCUGUACCGGGAUGGGGAACAGACGGAGCCCAGGUACUUCUGUGGGCACAUGCGCUGCC

CCCGGGAUGCUCUGGUAUCCCUUGCCCAUCCUUGGCCCCAGUCCACCGUGGUGCCAUGUG

GAGAGUGACAAGGGCACAGGGCUCAGCUGGGUGACCUCAAGCCUGCCAAGCAGGUUUCAC

CAACUUGGGGGUGUGAUACAUGCCCACCCUCCCUGGGGAGACCUCAGUGGUAGCUUUCCA

UGCUUUGGGCUGGGAUCUCAGCUGGACCAGGCCCCCGUUGACAGCCCCUGUUGAACCUCU

AAGAAAUAAUGAGCUAGGUGUGCUGGGCCAGACCGGGGGCGAUGGGUGAGGUGGGACCUG

AGAAGGAAGCUGGGCCCGCUGCCCCUGGGGAAGGGCAUGAUCGGAACCCAAUUUCAGUCC

UUGGGGCUCUCUUGAGAGGGUCAGGCUGGAGCAAGUGGUCAGAGGCAGCCCACCACGCAG

CCAGGCGUCUCCCAAGACACCCCUAGCCCCAGGACGGGUGAACCGCAGAGGAGAUUUCAG

GAGCCGUGAUCUUCUACCAGGCAGGGGAUGCAGGCGUGGGGGCGGGUGAAGCUUGCUUCC

AAAUGUCUAAGGCAUCUCAGGUGGUGAGUUCCCCAUCAUCAAAGGCAUGCAAGCUCGGCA

CCAAGUGAGCUGAUGUGAGGUGUUUGAUCCUCACAGCAGGAUGGGCUCCAAGUCUCCAGG

CAACACCAGCCAGCCGCCAGCCUUCUUCAGCAAGCUGACCGAGAGCAACUCCGCCAUGGU

CAAGUCCAAGAAGCAAGAGAUCAACAAGAAGCUGAACACCCACAACCGGAAUGAGCCUGA

AUACAGUAAGGGGCCUGCAGGCUCCCGGGGAAGCAUGGGGCCACAGGUGGGCGGGUGGCC

UGCCUGGGCAGCUGGAGCCGCCCAGUGGCAGAAACCCACGGUGCACCUUCGAAAGCUAAG

UGGCCCUGCUGACCACCUCCCCCCAGGCCCUUUGCCUCACAUUUGGGGAGCCCCAGGGCA

GUUUCUUGAUUUGCUGGGCUUUCCAUAGGAGCUUACUGGCACAGAAGAAUAGCACCCAGC

ACAUAGUAGGUGCCCAGUGAAUACCUGCAUGAAUACUGGGACCAGGGGUUGGAUCCCUCC

CACACAAGGGCCGGGCGCCUCCCACACUCAGCACCUGUGUGGCUUUGCACCCAUUGACGU

GGUUGCUGGGUAUGAACGCCCCACUCUGCUUCCCAGUCCCUAGCACAGCGCCUGGCAGUU

AGCAGAUCCACCAGGGAAUACGUGAGUGGGUGGGCAAAUAAAGAAUCUGUCACAGUCCCC

GACCCCAAGAAGCCUCAUCUGCCAGGGAAGUUUGGACAAAUCACAGAUGCUUUUCCCUUC

CUGGGGCUGGAGUAGAAACCUUGCAGAUAGUCACUGGCUUGCCGGGCACGGUGGCUCAUG

CCUAUAGGCCCAGCACUUUGGGAGGAUGAGGCAGGAGGAUUGCUUGAAGCCAGGAGUUCG

AGACCAGUCUGUGCAACAUAGCAAGACCCCAUCUCUACAAAAAACUUUAAAAACAGGCAC

ACACCUAUAGUCCAAGCUACUGGGGAGGCUGAGAUAGGAGGAUUUCUUGAGCCUCGGAGG

UCAAGGCUGCAGUGAGCUAUGAUCACACCACUGCACUCCAGCCUGGACAACAGAGCAAGA

CACUGUCUUAAAAAAAAAAAAAAUCUCUGACCCAGGCUGGUAACUCCAGGGCCCUGUAAG

UGCAGUCCAGGGAACCGUAGCAUCAGCAUCCCCAGGGUACUGGUUAGAAAUGCAGGCCCU

UGGCCAGGCGCGGUGGCUUACGCCUGUAAUCCCAGCACUUUGGGAGGUCAAGGCGGGUGG

AUCAUAUGAGGUCAGGAGUUUGAGACCAGCCUGACCAACAUGGUGAAACCCCGUCUCUAC

UAAAAAUACAAAAAUUAGCCAGGCGUGGUGGCGGAUGUCUGUAACCCCAGCUACUCGGGA

GGCUGAGGCAGGAGAAUCACUUGAACCUGGGAGGCGGAGGUUGCAGGGAGCCGAGAUUGC

ACAACUGCACUCCAGCCUGGGCAACAGAGCGAGACUCUAUCUCAAAAAAAAAAAAAAAAA

GAAAUGCAGACGCUUGGCCCUGUCCCAGGCCUGCUGCAUGAGAACCUGCAAUGCACAAGU

UUCCCCAGGUGAUGCCAGCACACCUGGCCUGGACCACACGGGACUGGUAGGGCAGGUAAU

UCCCAGAGACCUGGGGGCCUCACCCACUCUGUCACCCGCUUCCAGAUAUCAGCCAGCCUG

GGACGGAGAUCUUCAAUAUGCCCGCCAUCACCGGAACAGGUAACCCAUCCAGCCCUUGCU

AUAUGGCUGCCCUGGUCCCCUCCGCUCCCUCCCCACCCCUGCUCCAGCUGUCAUGAAGGG

ACGAGGAGCCUUCGCUAGUCUGGGUGUACCCCCUCAUUCUGGGAUGAACUAACCGCACAG

UAGGAUUCAGAGUCACACAACAGGCAGGCGAGGCUUGUUCCCUGUGUAGACAGGAUCCUC

GCUGUGCAGGGAAUCUCUGGAGUUAAGAUCCCCUCGGGUGGUUAGUAAGUAUCAGAUGCA

CCCUCACCAGCUGGAAACUCACCUUGCUUCUUCGCCAGCCUCAGCUGGAGAUGCACAUGU

CUGGACGAGGGGUGGGCCUGAGCUCAGAGCACAAGCCUCCGAGUUCACUCGGGCGUUUGU

UAUAGCUAGAGCUUCAUUCCUUAAAUCCAGCCAGGGAACUGGGAAGCCUUACUUUUUCUU

UCAAGAUCAAAUACAGGUGUGUGGCAGAGAUAGGUGUAAAAUUGACACGCACUUUUAAGC

UGAAACUUAAGACUUCUAUGAUCUUUUGGACUUAGGGGUCCCUUGAGGUUGGAGCCCCAU

CCUCUAGGAGGGCCCCAUUGUGUAUUUCCUUGGUGAGUCUGGGGUGUGGCCUCUGGGGGU

CACUCUGCAUGGGCAGGCCUGGCCCAGUGGGGCUGAGGCAGUUUUGGGGUCGGCUGCCUC

UGUGUGGGUGCCUGGUUAUCCUCUGGUCCUUUGGUGGAAGCUGAGGCCAGGAUGGAGGUC

GGAGAGGUUUGCUGAUCUUCCCUGGGGAACAUUCCUGGGCCUUGAGCCCUGGGAAUGGUG

AGCGAAGGAUAGUCGUUCAGAUAUUAUCAGGGGGUCAGCGAGGCCUCCAAAUGGGAGUCC

CAACUAGGACACCUCCACCUGCCCAGUGACUAGACACCGGGGGCAUUGCCAAGCCUCAGG

CAACGGGAAGAAGACAAUUUGGUCGAAACAACAAGAGGGUUAGAAAUGAAUGUUCCUGGG

AUGUUCUAAAUCUUGAAGUAGGUGCUAGACACACAGGUGUAUCUGUGUGUAAAAACUCAU

GGCAUGGUACAGUGAAGAUUUAUGCUAACACUUAUAUAUAAAUAUUUUGUAUUUAUCUUU

UUAAGACACGGUCUUUCUGUCACCUAGGCCAGAGUGCAGUGGUGCAAUCACAGCUCACUG

CAGCCUCCGCCUCCCAGGCUUAGAUGAUCCUCCUACCUCUCAGCCUCCUGAGCAGCUGGG

ACUACAGGCAUGCGCCACUGCACCCGAGUAAUUUUUUAUCUUUUUUGUAGAGACAGGCUC

UCAAUAUGUUACCCAGGCUAGUCUUGAACUCCUGUCCUCAAGCUGAGGAUCCUCCCACCU

CGACUGAUUUUUGUUUUUUGUGUUUGUUUGUUUGUCUGUUUGUUUGACAGAGUCUUGCUC

UGUCGCCCAGGCUGGAGUGCAGUGGUGCAAUCUUGGCUCACUGCAGCCUCUGCCUCCCGG

GUUUCAGCAGUUCUUCCACCUCAGCCUCCCAAGUAGCGGGAUUACAGACACCCGCCACUA

CACCUGGCUAAUUUUUGUAUUUUUAGUAGAGACGGGGUUUCAUCAUAUUGGCCAGGCUGA

UCUCGAACUCCUGACGUCAGAUGAUCCACCCACAUUGGGCCUCCCAAAGUGCUGGGAUUA

CAGGCGUGAGCAGUCACGCCCAGCCUGAUUUUUUCUUUUUAUGCAGUUUUAGUCCACAAG

AAGAAAUUUUCCAGGCUCCAGUCUUUGCCACUCAACCUGUGUCAGACUUCACCUUUAUAA

AUGGAGAUCGUUAAGCCUGGAGCCAGCUCCAUCAGGUCACGGACUCGUGGCCUUGGGUAG

AAGGCCCAGUCGCGCUGCUGUGAUUUCAGCACUCACCCUUGUUGGAAAUGUUGCUUUUUC

UUCAUGCACAUGGCUGUUUUUUCAAAAGUGCUGCAGAAAUGUGGCCAAGACAGCAGAAAA

GGUGUGAGCAUCCCUGGCUACAAACGUAUUUGAAACCAAAAGGGAAAAGAAAACGCAGGC

AGCCAGCAGUUGCUACAGAAACGUGAUUUUCAAAGCAUCUGCAUCACCCGCAACCUCAUG

GAAGCCUCGGUUUGGUUUACAAAGGAGCGAGUGGUCGGAAAGACGAUUUGACCACUUUGU

AUUCGUAUGAUUCAUUCGGAGCAGAAGUUUAAAACUCCAUGAAACACUUGCCCCGUGGUG

GGCACGGUGGAAUGGGGCUUCACUGGGGGCUUUUGAAAGCAAAGGCUGGUUUAACAAAAG

UUCUGAAAAGACAGCACCUCUGGGGGGUGUGCAAAGGCCCCCACCUCCACGUGGUUCACA

AGAAAGAAAAAAGGGAAAGGAAAUGUGGUUAACAGAAAAGGAGGUUUCCCCGAUUGUCAG

GAGGUGUUCUGGAAGCAUCCUCUUGGGACCGGUUUGUUGAUUCGACCACUUUGUCCUUGG

GACUGGGCAGCUGGGCCAGCUGGGGCCGGACCAGGGCUGGUCCCGUGAUUGUGUCUACUU

CUGCCCUGUCCCCUGCACGCACCGUAACUGCAUGGCUAUGACCCCGCCCUCAGCGUCCGU

CUCUGCACCUCUUUCUCCUCAUGGCUCCUGGGUGGGGAGAGGCAGAGGGAGGAGAGCAGG

CCCAGCUUGGUGGGGAGGUACGGGGCUGCACGUCCCAUCACACAGGUGGAGGUGGGGCAG

CGGGAGGACCAUCUGGUCACCUUCUCUCUCUCAGUCCCUUCCCCAGCCCCCAGCCAGCCC

CCAGCUCUGGCUUGAGCCAAUUUUCUAGCAGCCUGUUCCCCAAACAGGGUAGCCCUCCCA

UCUCCCACCCUCUCCAUUAAGGCCACUUGAGAUUUAAAAAAAAAACAAACAAAACAGCCC

CAGCUAGGAUUGGAGGUGCAGACGGGGCUUGUGAUUUCCCAGAGGACAGAAUAGGAAUGA

GAAUAGGGGCUGGUGGGGGCAUCUGACCUCCCCUACCCCACCUCCCUGGCAGUGCCCAAG

AGCUUCCGGGGCCCCAGGUAGAAGGAACCAGCCUCUCCCCUUUUAUCACCACCCAGCAGG

AAAAAAAGGGUGGGGAGGGAUAGGGAAAUAAAUAUGUUGCUUUGCCGAAAUGUGCUCACU

GUGUAUUUCUCUCUCCUCCUCCUCCUCCUCCUCCUUCCCUCUCUCUCUCCCUUCUCUCUC

UCUGUCGCCCCUCUGGCUCCCCCUCCCCGGCCCCCAUGUGUCUGUCUGUCUGUCUGUCUC

UCUCUCCCAGGCCUUAUGACCUAUAGAAGCCAGGCGGUGCAGGAACAUGCCAGCACCAAC

AUGGGGCUGGAGGCCAUAAUUAGAAAGGCACUCAUGGGUAAAUAUGACCAGUGGGAAGAG

UCCCCGCCGCUCAGCGCCAAUGCUUUUAACCCUCUGAAUGCCAGUGCCAGCCUGCCCGCU

GCUAUGCCCAUAACCGCUGCUGACGGACGGAGUGACCACACACUCACCUCGCCAGGUCUG

CAGGCCACCCCCGCCCCGCCCCCGUCUGUCCCCACCCCCGGUGUGAUUAAUCCUCGCUCC

UCCGCGCUCCUCUGACAACCCCCUCCUCGAGCUUUGGAGCUUGUGACUUUAUUUUUGUGC

GUGUUUGACCUCGUUCUGGAGUUUGCUAAUCUGAAGCUGGGCUGACACCCCCCAAGUGUC

UGUACCCUCUGCCCCCCAGCCCCGGCCCUCCUGCCCACUAGGCCCGAAGCGCUGCCGCCU

CCCUCGGACACUCACACUGCUGUCCGCCCCCCAGUCCUCCCGCCUUCCUCCCUGCGGGGA

CCCGGCUUCUUGGCCCAUCUGUCUCCUUGGGGGAGAGCAGGCUGGAGUGAAGCCCCACCC

ACACUGUGUGGACAGGGGAAUGGCAGCCAGGCCUGUGCUCAGCAUCUGCCAGGCCCACUA

UGUGUCCUCAGCUGCCCCUUAGCCUGGUGGGGAGGAGCCCAGGGUCUGUCUCAGCCCUGG

GAGUCAGGGAGCCUUAGGGGUCAGCCUGGUUCCCCAUCAAUACCCUCUGUAUCGGGGCAG

GGAGCUGAGGAGAGAGUUGCAGUUGUCCAACCUGGACACUGAGGCCCCAAGAAGCUUCUG

GAGCCUCUGUGGGGGUCAGGCCUGGCCUCAGGGGUCCUGACUUCUCUGCACGGGGCCUGG

AUCCUGCCUAGCCUUAGCAUGGUCCUGGGGCCACGCUCAAACUGGAAGCCCAGCUUCAUG

CUUAGGGUUCCAGCCCCUGGGGCUGGGGUCGCUGCAGGACAGCCCAGGGGGCUAUUGCAA

ACAGCAGACAGUUUAGCCACCUCCCCCUGCCCAGCAGAAACUAUCUCCACCCUCCAGGCU

CAUGGCUGGCCAUCUGGUCAACCCUGGCUUCCCAGCUGGGGCGGGCAGCAGGGAGGAGGG

GUUCACGUUACUGCUCUCCCUCCCUCUUUGGAGGGCCCAUGGCAGGACCUCACCCUGCCC

CUGUGGCCCCACCGUAGCGUCGGUGCUGUCUUCACUGCCCAACGCAGCCCCUUCCCAUCU

UGUCCCCCUGCAGGUGGCGGCGGGAAGGCCAAGGUCUCUGGCAGACCCAGCAGCCGAAAA

GCCAAGUCCCCGGCCCCGGGCCUGGCAUCUGGGGACCGGCCACCCUCUGUCUCCUCAGUG

CACUCGGAGGGAGACUGCAACCGCCGGACGCCGCUCACCAACCGCGUGUGGGAGGACAGG

CCCUCGUCCGCAGGUGGGCACCAGGUGGGGACAGGGCUGGGCUCGCUGAGCCCCCAACAC

UGGGCUGUGAAUGCUGCCGGGGCACUGAAUGCUGAGCGCCUGCUGCAUGCAGAGCUCAGA

CUCGGGCCUGUUCUGUGGAAACGCUGUCCAGGGGCCUGGGCAGCUGAGCCUGGCUCACGA

ACCAUCAGGAUGCUUCUGCCAGGGGCACAGGCAGAGGAAAAGGGUGUGGUUGGGGCCGGC

CAGAUCACAUGGGCCUGGUGGACCAUCCAGGGGAGCCUGGACUUCCUUUGAUGGGUAGUG

AGGAGGCGUGGAGGGCUUUCGGAAGGGAAAUGCUCAUCUAACAUAGGGUCAGAAGGCCCC

UGGGAGAAUAGACCAGAGGGACGGUGAAUAGGCUACUGCUAUAGUCCAGGCAAAAGACAG

CGGCAGGGUGUGAACAGGGACGGAAGUAGAACAGGUGAAGAGAGGCAGACGGUUCUGAGA

CCUGUUCGCAAUUAGAAAGGUCUGGGAGGACUGUUGGACAGAAACGCUGAGGCCUCUGUU

CCCGGUGUGGUGGGCAGGCGGCCAGCAGGGGCUGCCGGGCUCACAGAGGCCUCCUGGGCA

UCUUUCGCUUCUUCCCGCAGGUUCCACGCCAUUCCCCUACAACCCCCUGAUCAUGCGGCU

GCAGGCGGGUGUCAUGGCUUCCCCACCCCCACCGGGCCUCCCCGCGGGCAGCGGGCCCCU

CGCUGGCCCCCACCACGCCUGGGACGAGGAGCCCAAGCCACUGCUCUGCUCGCAGUACGA

GACACUCUCCGACAGCGAGUGACUCAGAACAGGGCGGGGGGGGGGGCGGUGUCAGGUCCC

AGCGAGCCACAGGAACGGCCCUGCAGGAGCAGGGCGGCUGCCGACUCCCCCAACCAAGGA

AGGAGCCCCUGAGUCCGCCUGCGCCUCCAUCCAUCUGUCCGUCCAGAGCCGGCAUCCUUG

CCUGUCUAAAGCCUUAACUAAGACUCCCGCCCCGGGCUGGCCCUGUGCAGACCUUACUCA

GGGGAUGUUUACCUGGUGCUCGGGAAGGGAGGGGAAGGGGCCGGGGAGGGGGCACGGCAG

GCGUGUGGCAGCCACACGCAGGCGGCCAGGGCGGCCAGGGACCCAAAGCAGGAUGACCAC

GCACCUCCACGCCACUGCCUCCCCCGAAUGCAUUUGGAACCAAAGUCUAAACUGAGCUCG

CAGCCCCCGCGCCCUCCCUCCGCCUCCCAUCCCGCUUAGCGCUCUGGACAGAUGGACGCA

GGCCCUGUCCAGCCCCCAGUGCGCUCGUUCCGGUCCCCACAGACUGCCCCAGCCAACGAG

AUUGCUGGAAACCAAGUCAGGCCAGGUGGGCGGACAAAAGGGCCAGGUGCGGCCUGGGGG

GAACGGAUGCUCCGAGGACUGGACUGUUUUUUUCACACAUCGUUGCCGCAGCGGUGGGAA

GGAAAGGCAGAUGUAAAUGAUGUGUUGGUUUACAGGGUAUAUUUUUGAUACCUUCAAUGA

AUUAAUUCAGAUGUUUUACGCAAGGAAGGACUUACCCAGUAUUACUGCUGCUGUGCUUUU

GAUCUCUGCUUACCGUUCAAGAGGCGUGUGCAGGCCGACAGUCGGUGACCCCAUCACUCG

CAGGACCAAGGGGGCGGGGACUGCUGGCUCACGCCCCGCUGUGUCCUCCCUCCCUCCCUU

CCUUGGGCAGAAUGAAUUCGAUGCGUAUUCUGUGGCCGCCAUCUGCGCAGGGUGGUGGUA

UUCUGUCAUUUACACACGUCGUUCUAAUUAAAAAGCGAAUUAUACUCC
