# Supplementary material for: The pan-cancer lncRNA PLANE regulates an alternative splicing program to promote cancer pathogenesis
Source: Nat Commun. 2021 Jun 18;12:3734. doi: 10.1038/s41467-021-24099-4 (PMC8213729; doi:10.1038/s41467-021-24099-4)
Supplement: Supplementary file 7 — Reporting Summary [file 41467_2021_24099_MOESM7_ESM.pdf]

nature research

Corresponding author(s): Xu Dong Zhang

Last updated by author(s): May 15, 2021

Reporting Summary

Nature Research wishes to improve the reproducibility of the work that we publish. This form provides structure for consistency and transparency in reporting. For further information on Nature Research policies, see our Editorial Policies and the Editorial Policy Checklist.

Statistics

For all statistical analyses, confirm that the following items are present in the figure legend, table legend, main text, or Methods section.

n/a	Confirmed	
☐	☒	The exact sample size (*n*) for each experimental group/condition, given as a discrete number and unit of measurement
☐	☒	A statement on whether measurements were taken from distinct samples or whether the same sample was measured repeatedly
☐	☒	The statistical test(s) used AND whether they are one- or two-sided<br>*Only common tests should be described solely by name; describe more complex techniques in the Methods section.*
☒	☐	A description of all covariates tested
☐	☒	A description of any assumptions or corrections, such as tests of normality and adjustment for multiple comparisons
☐	☒	A full description of the statistical parameters including central tendency (e.g. means) or other basic estimates (e.g. regression coefficient) AND variation (e.g. standard deviation) or associated estimates of uncertainty (e.g. confidence intervals)
☐	☒	For null hypothesis testing, the test statistic (e.g. *F*, *t*, *r*) with confidence intervals, effect sizes, degrees of freedom and *P* value noted<br>*Give P values as exact values whenever suitable.*
☒	☐	For Bayesian analysis, information on the choice of priors and Markov chain Monte Carlo settings
☒	☐	For hierarchical and complex designs, identification of the appropriate level for tests and full reporting of outcomes
☐	☒	Estimates of effect sizes (e.g. Cohen's *d*, Pearson's *r*), indicating how they were calculated

Our web collection on statistics for biologists contains articles on many of the points above.

Software and code

Policy information about availability of computer code

Data collection	Step One V2.2.3 software; Image Studio V5.2 software; Image Lab V6.0 software, BD FACS Diva v8.0.2 software; Olympus Fluoview 3000 FV31S-SW V2.4.1.198 software.
Data analysis	Microsoft Excel 2016 software; GraphPad Prism V8.4.2 software; adobe photoshop elements V12.0 software; ImageJ V1.50e software; FlowJo v7.6 software; Gene Set Enrichment Analysis (GSEA) V4.1.0 software; MAJIQ V2.1 software; IGV_2.8.13 software; GeneMarker V1.91 software; HiSeq Control (v3.4.0) Software; NovaSeq Control (v1.6) Software.

For manuscripts utilizing custom algorithms or software that are central to the research but not yet described in published literature, software must be made available to editors and reviewers. We strongly encourage code deposition in a community repository (e.g. GitHub). See the Nature Research guidelines for submitting code & software for further information.

Data

Policy information about availability of data

All manuscripts must include a data availability statement. This statement should provide the following information, where applicable:

- Accession codes, unique identifiers, or web links for publicly available datasets
- A list of figures that have associated raw data
- A description of any restrictions on data availability

The RNA sequencing data have been deposited in the NCBI Gene Expression Omnibus database under the accession code GSE162215 (https://www.ncbi.nlm.nih.gov/geo/query/acc.cgi?acc=GSE162215) and GSE172124 (https://www.ncbi.nlm.nih.gov/geo/query/acc.cgi?acc=GSE172124). The mass spectrometry proteomics data have been deposited to the ProteomeXchange Consortium (http://proteomecentral.proteomexchange.org) via the iProX partner repository with the dataset identifier PXD022747. The long noncoding RNA expression data and E2F1 mRNA expression data referenced during the study are available in a public repository from the Cancer RNA-seq Nexus dataset (http://syslab4.nchu.edu.tw/). The PLANE expression and relevant cancer patient survival

data referenced during the study are available in a public repository from the GEPIA website (http://gepia.cancer-pku.cn/) under the accession codes TCGA-LUSC, TCGA-COAD, TCGA-KIRC and TCGA-UCEC. The MELTF expression and relevant cancer patient survival data were obtained from the human protein atlas website (https://www.proteinatlas.org/). The gene amplification frequency data referenced during the study are available in a public repository from the cBioPortal website (https://www.cbioportal.org/) under the accession code TCGA PanCancer Atlas Studies. The source data underlying Figs. 1a, c-h, 2a-g, i, 3a-d, f-k, 4b-k, 5a-f, h, 6a-h and Supplementary Figs. 1a, b, d, f, 2a-c, e, 3b-f, 4c, d, 5a, b, d-f, 7a-c, 8b, c, e, h, 9a-d, 10a, b, 11b, 12g-i, 13a-h are provided as a Source Data file.

Field-specific reporting

Please select the one below that is the best fit for your research. If you are not sure, read the appropriate sections before making your selection.

☒ Life sciences ☐ Behavioural & social sciences ☐ Ecological, evolutionary & environmental sciences

For a reference copy of the document with all sections, see nature.com/documents/nr-reporting-summary-flat.pdf

Life sciences study design

All studies must disclose on these points even when the disclosure is negative.

Sample size	No study size calculation was performed and the sample size was based on our prior studies using the same types of assays and published literature to ensure statistically significant results (Luo et al. Nat Commun. 2019 PMID: 31189930; Wang et al. Cancer Cell. 2018 PMID: 29622465; Lu et al. Cancer Res. 2018 PMID: 29180471). All key experiments were repeated independently using different cell lines or different techniques. For in vitro studies n=3 was used as a standard sample size. For in vivo mouse model, n=6 was chosen to detect the difference of tumor size and weight among different groups. For human tissue samples, sample size was determined by the availability of samples. No statistical method was used to predetermine sample size as sample size selection with the above published methods is sufficient to detect meaningful biological differences with good reproducibility
Data exclusions	No data were excluded for this study.
Replication	n=3 biological replicates for in vitro cellular experiments unless otherwise specified. The in vivo animal experiments were performed using 6 mice per group. All attempts at replication were successful.
Randomization	The cells and animals were randomly grouped for the experiments. Randomization was not relevant for human tissue samples as the samples were grouped according to their pathology (normal, carcinoma and adenoma).
Blinding	For experiments using cell lines the investigators were not blinded during data acquisition and analysis. The application of treatments and processing procedures negated the possibility of blinding but there was no human bias given all data was collected independently using instrumentation. Similarly, in the animal experiments the investigator was not blinded to the group allocation as the same investigator both planned and performed the experiment. Two observers measured volumes/weights to alleviate human bias in these data. The scoring and quantification of human tissue samples were performed in a blinded manner.

Reporting for specific materials, systems and methods

We require information from authors about some types of materials, experimental systems and methods used in many studies. Here, indicate whether each material, system or method listed is relevant to your study. If you are not sure if a list item applies to your research, read the appropriate section before selecting a response.

Materials & experimental systems

n/a	Involved in the study	
☐	☒	Antibodies
☐	☒	Eukaryotic cell lines
☒	☐	Palaeontology and archaeology
☐	☒	Animals and other organisms
☐	☒	Human research participants
☒	☐	Clinical data
☒	☐	Dual use research of concern

Methods

n/a	Involved in the study	
☒	☐	ChIP-seq
☐	☒	Flow cytometry
☒	☐	MRI-based neuroimaging

Antibodies

Antibodies used	All antibodies and relative information about their species, catalog numbers, companies and dilution have been mentioned in the method section. E2F1 (ab179445), Rabbit monoclonal IgG; H3K4me3 (ab1012), Mouse monoclonal IgG; H3K27me3 (ab192985), Rabbit monoclonal IgG; SC35 (ab11826), Mouse monoclonal IgG; U1-70K (ab51266), Rabbit polyclona IgG; hnRNPK (ab52600), Rabbit monoclonal IgG and Normal rabbit IgG (ab172730) antibodies were purchased from Abcam; Normal mouse IgG (sc-2025) antibody was from Santa Cruz Biotechnology; HRP Conjugated AffiniPure Goat Anti-mouse IgG (BA1050), HRP Conjugated AffiniPure Goat Anti-rabbit IgG (BA1054) and CY3 Conjugated AffiniPure Goat Anti-rabbit IgG (BA1032) antibodies were from BOSTER Biological Technology; hnRNPM (26897-1-AP) antibody was from Proteintech Group; Chicken anti-Mouse IgG (H+L) Cross-Adsorbed Secondary Antibody (A21200) was from ThermoFisher Scientific. NCOR2 (62370), Rabbit monoclonal IgG, was from Cell Signaling Technology.

Validation	E2F1: Abcam, Cat No. ab179445, Species: mouse, human. Abcam website antibody validation: 1) western blot analysis of E2F1 expression in whole cell lysates from HepG2, Hela, NIH 3T3, T47D and MDA-MB-231 cells and lysates from human lung, spleen and fetal muscle tissues shows a single band of E2F1 at 70 kDa; 2) Western blot analysis on immunoprecipitation assay using cell lysate from NIH 3T3 cells shows a single band of E2F1 at 70 kDa. 3) ChIP analysis shows interactions between E2F1 and promoter regions of tapasin, TAP1, TAP2 and LMP2 genes using lysates from NIH/3T3 cells.<br>H3K4me3: Abcam, Cat No. ab1012, Species: Rat, Cow, Human and Rice. Abcam website antibody validation: 1) western blot analysis of H3K4me3 expression in Calf Thymus Histone Preparation Nuclear Lysate shows a single band of H3K4me3 at the expected MW of 15 kDa; 2) ChIP assay using chromatin from U2OS cells shows the transcribed region of GAPDH, RPL30 and ALDOA genes are co-immunoprecipitated with H3K4me3; 3) immunocytochemistry in SK-N-SK cells shows H3K4me3 is localized in the nucleus.<br>H3K27me3: Abcam, Cat No. ab192985, Species: Mouse, Rat, Human. Abcam website antibody validation: 1) western blot analysis of H3K27me3 expression in the whole cell lysates from Hela and NIH 3T3 cells shows a single band of H3K27me3 at the expected MW of 15 kDa; 2) western blot analysis of H3K27me3 expression in the cell lysate from the EED-/- mouse lacking H3K27me3 shows the absence of signal at the expected MW; 3) ChIP assay using chromatin from Hela cells shows the transcribed region of GAPDH, RPL30, MYO-D, SERPINA, STA2 and STAa genes are co-immunoprecipitated with H3K27me3. 4) Immunofluorescent analysis of 4 paraformaldehyde-fixed, 0.1% Triton X-100 permeabilized HeLa (Human epithelial cells from cervix adenocarcinoma) cells shows nuclear staining.<br>SC35: Abcam, Cat No. ab11826, Species: Mouse, Rat, Human. Abcam website antibody validation: 1) immunofluorescence analysis of SC35 localization in Rin-5F, NIH 3T3, MCF-7, HEK-293, U2OS, HT-29 and Hela cells shows positive signals in the nucleus; 2) immunofluorescence analysis of SC35 in HEK-293T and RKO cells transiently transfected with CDX2/AS-His shows colocalization of CDX2/AS and SC35 in the nucleus.<br>U1-70K: Abcam, Cat No. ab51266, Species: Human. Abcam website antibody validation: 1) western blot analysis of U1-70K expression in the cell lysate from HepG2 cells shows a single band at the expected MW of 52 kDa; 2) immunohistochemistry (formalin/PFA-fixed paraffin-embedded sections) analysis of human pineal tissue shows positive staining in the nuclei of pinealocytes.<br>hnRNPK: Abcam, Cat No. ab52600, Species: Mouse, Rat, Human. Abcam website antibody validation: 1) western blot analysis of hnRNPK expression in whole cell lysates from Jurkat, Hela, NIH 3T3, MEF and PC-12 cells shows a single band at the expected MW of 51 kDa; 2) western blot analysis on the immunoprecipitation assay using the whole cell lysate from Jurkat cells shows a single band at the expected MW of 51 kDa in the group using hnRNPK antibody but not in the group using rabbit IgG antibody.<br>NCOR2: Cell Signaling Technology, Cat No. 62370, Species: Human, Monkey. Cell Signaling Technology website antibody validation: western blot analysis of NCOR2 expression in the whole cell lysates from MOLT-4, SW620, Hela and 293T cells shows bands at the expected MW of 270 kDa. Certificate of analysis from CST website: https://media.cellsignal.com/coa/62370/1/62370-lot-1-coa.pdf.<br>hnRNPM: Proteintech Group, Cat No. 26897-1-AP, Species: Human, Mouse. Proteintech Group website antibody validation: 1) western blot analysis of hnRNPM expression in whole cell lysates from colo 320, MCF-7, Caco-2 and MDA-MB-453s cells shows bands at expected MW of 73-77 kDa; 2) western blot analysis of hnRNPM expression in MCF-7 cells transfected with control shRNA or hnRNPM shRNA shows decreased signal in the hnRNPM shRNA transfected MCF-7 cells at the expected MW of 73-77 kDa, confirming the specificity of the antibody for hnRNPM.

Eukaryotic cell lines

Policy information about cell lines

Cell line source(s)	A549, MCF-7, HCT116 and Eca109 cell lines were purchased from ATCC. H1299, CCC-HSF-1 and CCC-HIE-2 cell lines were purchased from National Science and Technology Infrastructure (NSTI, Shanghai, China). NCI-H1975 and NCI-H226 cell lines were kind gifts from Prof Xiaoju Zhang (Respiration Department, Henan Provincial People's Hospital, Zhengzhou, China), originally purchased from ATCC.
Authentication	Individual cell line authentication was confirmed using the AmpFISTR Identifiler PCR Amplification Kit (ThermoFisher Scientific, #4427368) from Applied Biosystems and GeneMarker V1.91 software (SoftGenetics LLC).
Mycoplasma contamination	All cells used in this study were tested negative for mycoplasma contamination.
Commonly misidentified lines (See ICLAC register)	No commonly misidentified cell lines were used.

Animals and other organisms

Policy information about studies involving animals; ARRIVE guidelines recommended for reporting animal research

Laboratory animals	nu/nu nude mice, female, 4-week old were purchased from Shanghai SLAC Laboratory Animal Co.,Ltd. All the mice were housed in a temperature-controlled room (21-23°C) with 40–60% humidity and a light/dark cycle of 12h/12h.
Wild animals	This study did not involve wild animals.
Field-collected samples	This study did not involve samples collected from the field.
Ethics oversight	Studies on animals were conducted in accordance with relevant guidelines and regulations and were approved by the Animal Research Ethics Committee of the first affiliated hospital, Shanxi Medical University and Shanxi Cancer Hospital and Institute (China).

Note that full information on the approval of the study protocol must also be provided in the manuscript.

Human research participants

Policy information about studies involving human research participants

Population characteristics	The tissue microarray of a cohort of 75 lung squamous cell carcinoma (LUSC) with paired normal tissues were purchased from Shanghai Outdo Biotech Co., Ltd. All 75 patients have the information of clinicopathological characteristics. This cohort were composed of 69 males and 6 females with median age of 62 years old.<br>The tissue microarray of a cohort of 92 lung adenocarcinoma (LUAD) with paired normal tissues were purchased from Shanghai Outdo Biotech Co., Ltd. All 92 patients have the information of clinicopathological characteristics. This cohort were composed of 51 males and 41 females with median age of 63 years old.<br>The tissue microarray of a cohort of 89 colon cancer with paired normal tissues were purchased from Shanghai Outdo Biotech Co., Ltd. All 89 patients have the information of clinicopathological characteristics. This cohort were composed of 51 males and 38 females with median age of 68 years old.<br>A cohort of 12 colon tissues, 12 colon adenoma tissues and 12 normal colon tissues were collected at the Shanxi Cancer Hospital, and the covariate-relevant characteristics were not recorded as it is irrelavent to this study.<br>A cohort of 22 LUSC tissues, 24 LUAD tissues and 3 adjacent normal tissues were collected by the Henan Provincial People's Hospital (Zhengzhou, China), and the covariate-relevant characteristics were not recorded as it is irrelavent to this study.<br>A cohort of 25 freshly isolated ESCC tissues and adjacent normal tissues were collected by the Department of Thoracic Surgery, the First Affiliated Hospital of Anhui Medical University, and the covariate-relevant characteristics were not recorded as it is irrelavent to this study.<br>A cohort of 25 freshly isolated BRCA tissues and adjacent normal tissues were collected by the Department of Breast Surgery, Shanxi Bethune Hospital, and the covariate-relevant characteristics were not recorded as it is irrelavent to this study.
Recruitment	Sample recruitment in this study was from cancer patients across multiple institutes following the guidelines approved by the Human Research Ethics Committees of the Henan Provincial People's Hospital. the Human Research Ethics Committees of Shanxi Bethune Hospital and the institutional review board of Anhui Medical University. No self-selection bias or other biases are present.
Ethics oversight	All human studies were conducted in accordance with relevant guidelines and regulations. Studies using formalin-fixed paraffin-embedded (FFPE) normal colon mucosa, colon adenoma, and COAD tissues retrieved from archives of the Department of Pathology at Shanxi Cancer Hospital (Taiyuan, China) were approved by the Human Research Ethics Committees of the Shanxi Cancer Hospital. Studies using freshly isolated LUAD, LUSC and adjacent normal tissues collected by the Henan Provincial People's Hospital (Zhengzhou, China) were approved by the Human Research Ethics Committees of the Henan Provincial People's Hospital. Studies using freshly isolated BRCA and adjacent normal tissues collected by the Department of Breast Surgery, Shanxi Bethune Hospital (Taiyuan, China) were approved by the Human Research Ethics Committees of Shanxi Bethune Hospital. Studies using freshly isolated ESCC and adjacent normal tissues collected by the Department of Thoracic Surgery, the First Affiliated Hospital of Anhui Medical University (Hefei, China) were approved by the institutional review board of Anhui Medical University.

Note that full information on the approval of the study protocol must also be provided in the manuscript.

Flow Cytometry

Plots

Confirm that:

☒ The axis labels state the marker and fluorochrome used (e.g. CD4-FITC).

☒ The axis scales are clearly visible. Include numbers along axes only for bottom left plot of group (a 'group' is an analysis of identical markers).

☒ All plots are contour plots with outliers or pseudocolor plots.

☒ A numerical value for number of cells or percentage (with statistics) is provided.

Methodology

Sample preparation	Cell cycle analysis was performed using the Cell Cycle and Apoptosis Analysis Kit (Meilunbio, #MA0334; Dalian, China) according to the manufacturer's instructions followed by flow cytometry. Briefly, cells were harvested and fixed in 75% ethanol at 4 °C overnight. After being centrifuged, cells were incubated in the staining solution at 37 °C in the dark for 30 min. Then cells were subjected to analysis using a flow cytometer (FACSAria, BD Biosciences).
Instrument	BD FACSCanto flow cytometer
Software	BD FACS Diva v8.0.2 software
Cell population abundance	No sorting was employed.
Gating strategy	Cells were gated based on forward and side scatter plots, only avoiding debris and aggregates and no extensive gating strategy was used.

☒ Tick this box to confirm that a figure exemplifying the gating strategy is provided in the Supplementary Information.
