# Supplementary material for: Author Correction: The pan-cancer lncRNA PLANE regulates an alternative splicing program to promote cancer pathogenesis
Source: Nat Commun. 2025 May 16;16:4556. doi: 10.1038/s41467-025-59086-6 (PMC12084411; doi:10.1038/s41467-025-59086-6)

**Original data for colony formation assays in A549 cells with PLANE knocked down by siRNA as shown in Figure 2c**

The published version:

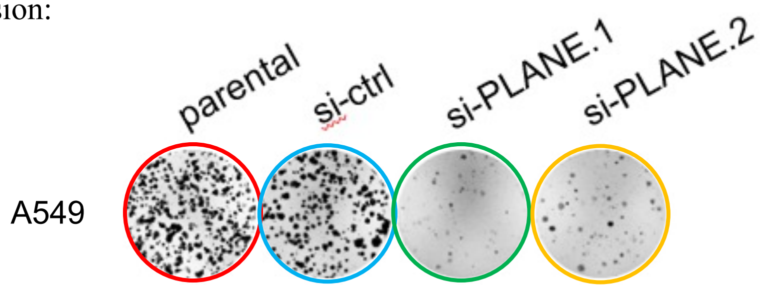


The original data used for the presentation as shown above:

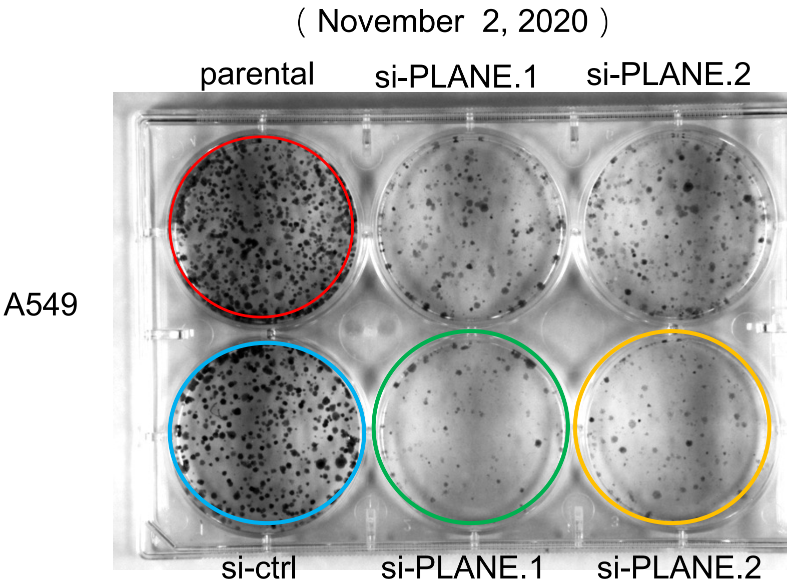


The additional repeats :

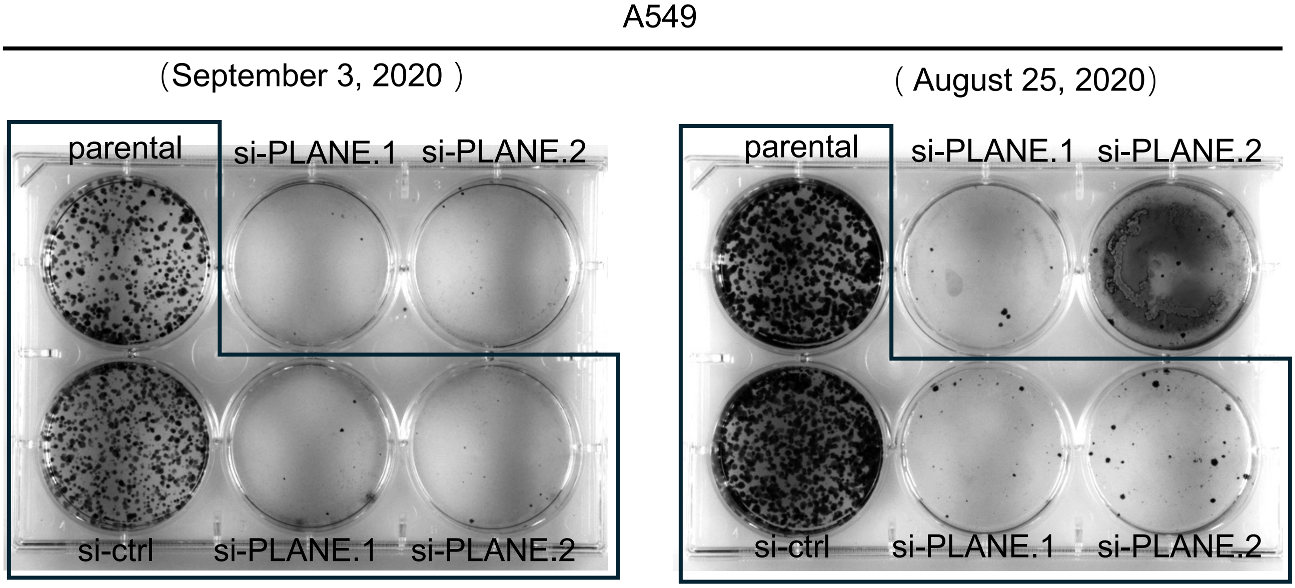

**Original data for colony formation assays in H1299 cells with PLANE knocked down by siRNA as shown in Figure 2c:**

The published version:

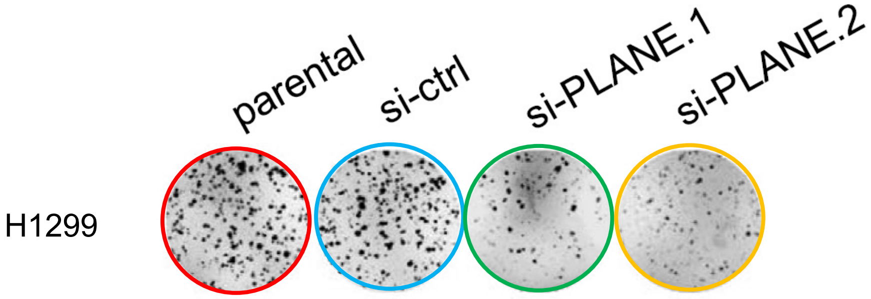


The original data used for the presentation as shown above:

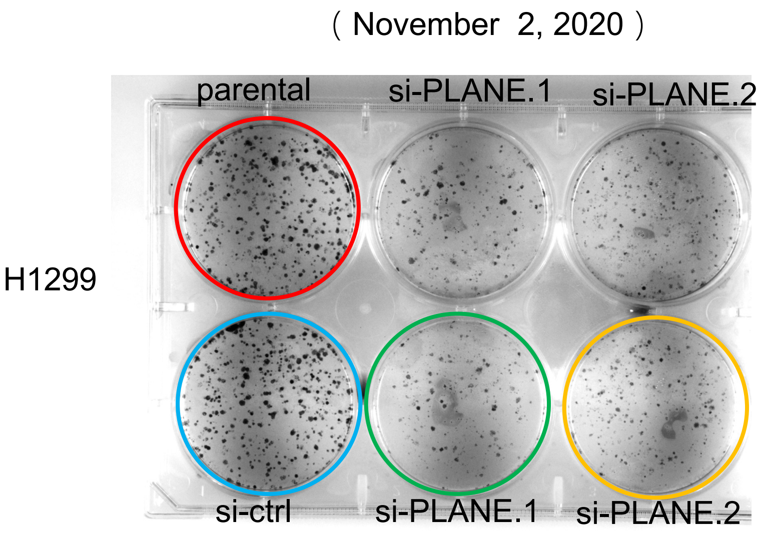


The additional repeats :

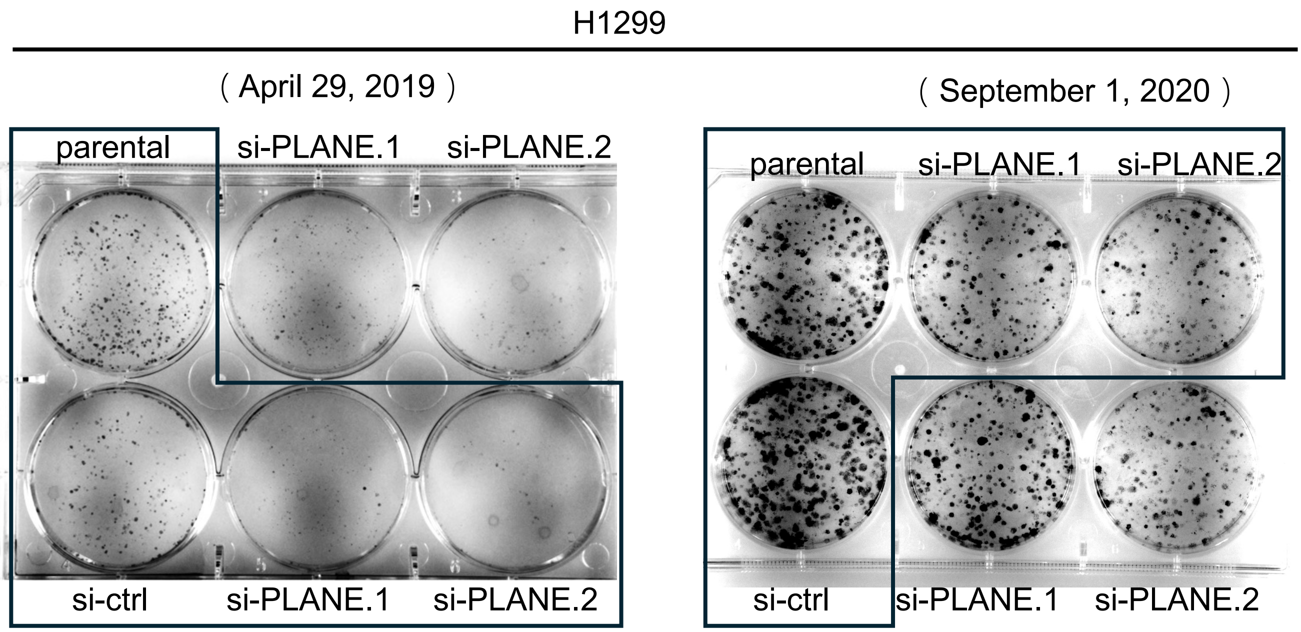

**Original data for colony formation assays in NCI-H226 cells with PLANE knocked down by siRNA as shown in Figure 2c**

The published version:

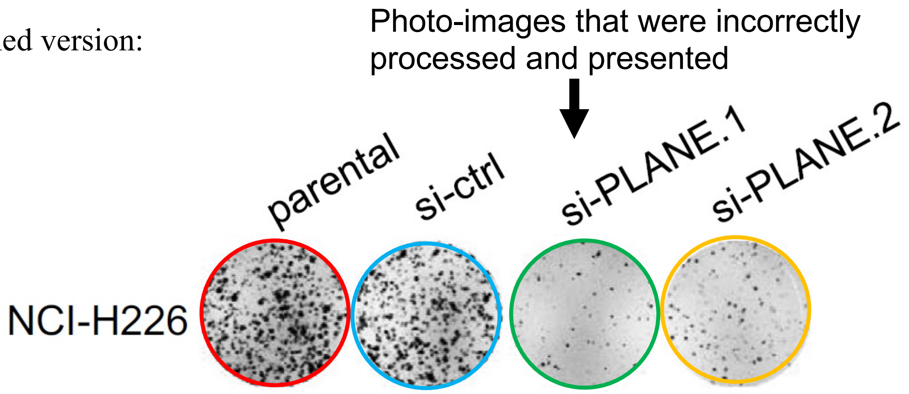


The original data used for the correct and incorrect photographs:

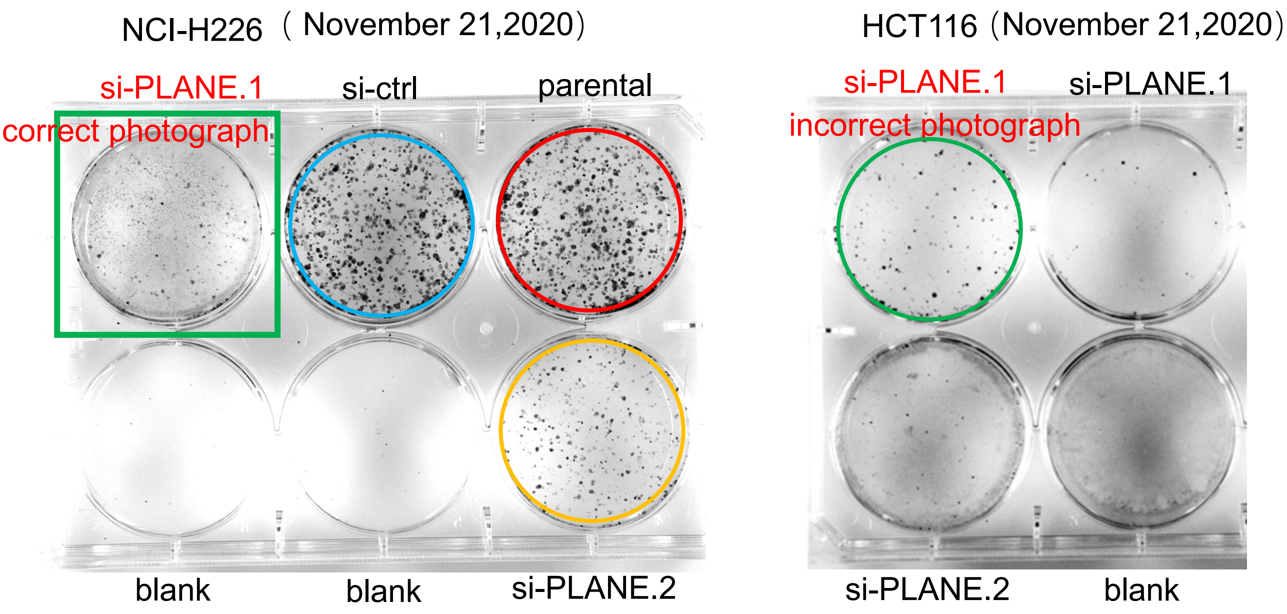


The additional repeats :

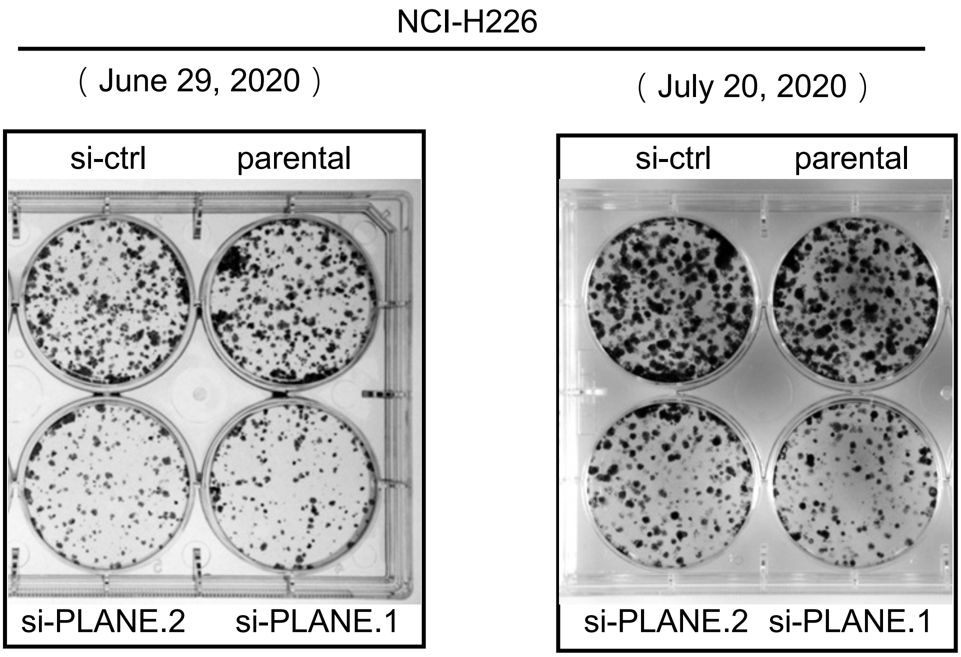

**Original data for colony formation assays in HCT-116 cells with PLANE knocked down by siRNA as shown in Figure 2c**

The published version:

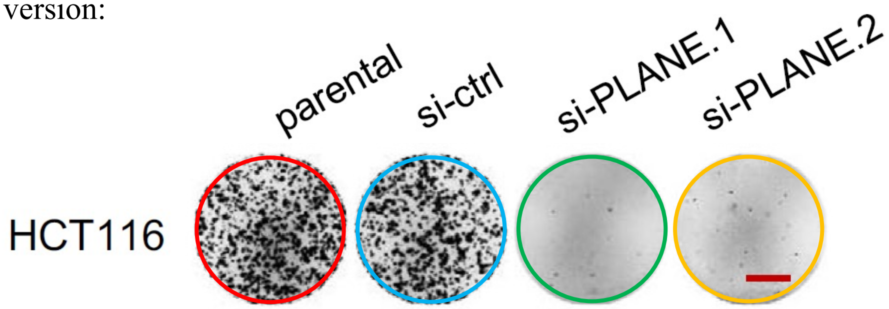


The original data used for the presentation as shown above:

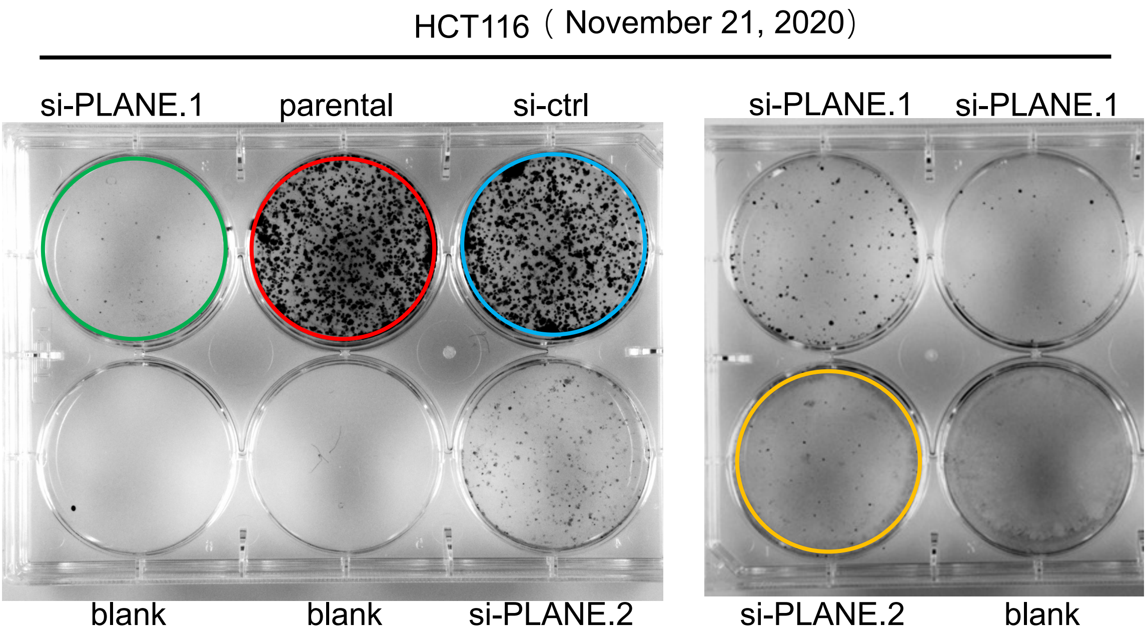


The additional repeats :

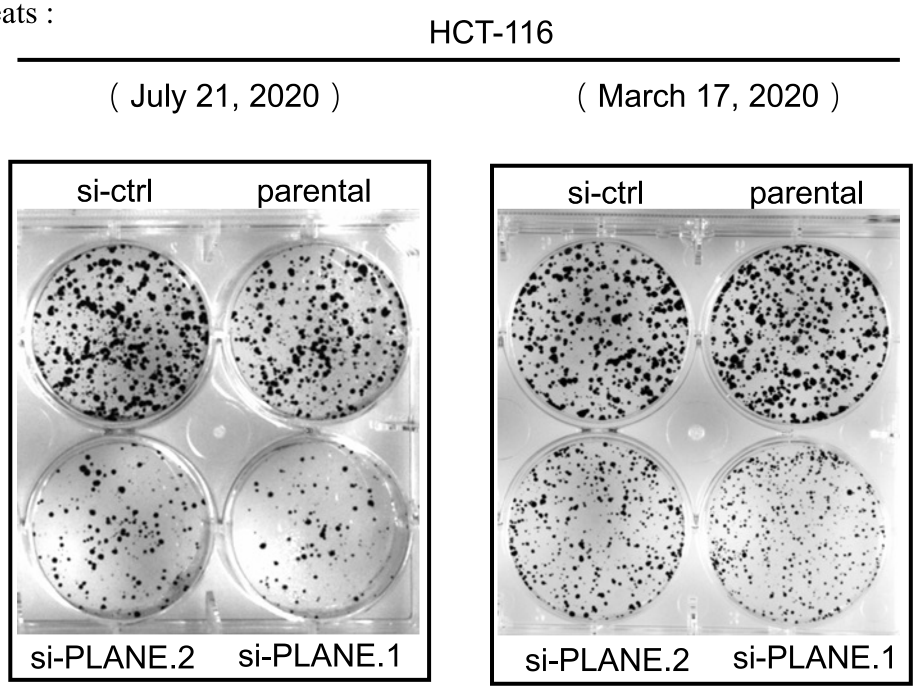

**Original data for colony formation assays in A549.shPLANE.1 cells with PLANE knocked down by shRNA as shown in Figure 2f**

The published version:

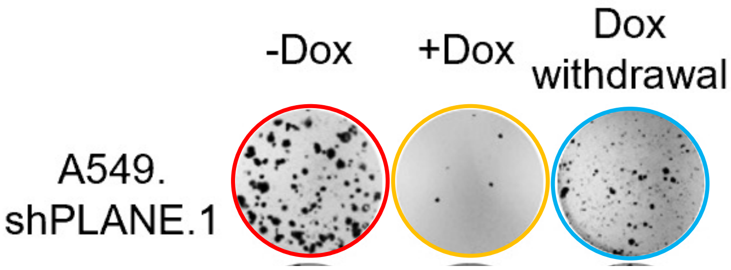


The original data used for the presentation as shown above:

A549.shPLANE.1 （April 29, 2019）

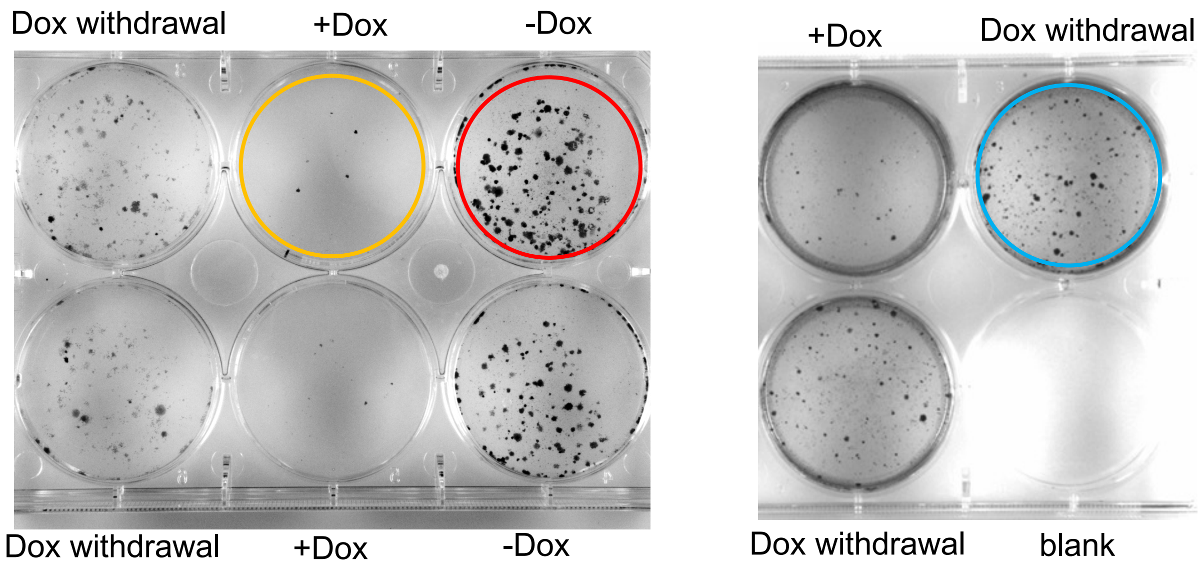


The additional repeats :

（May 16, 2019） （April 30, 2020）

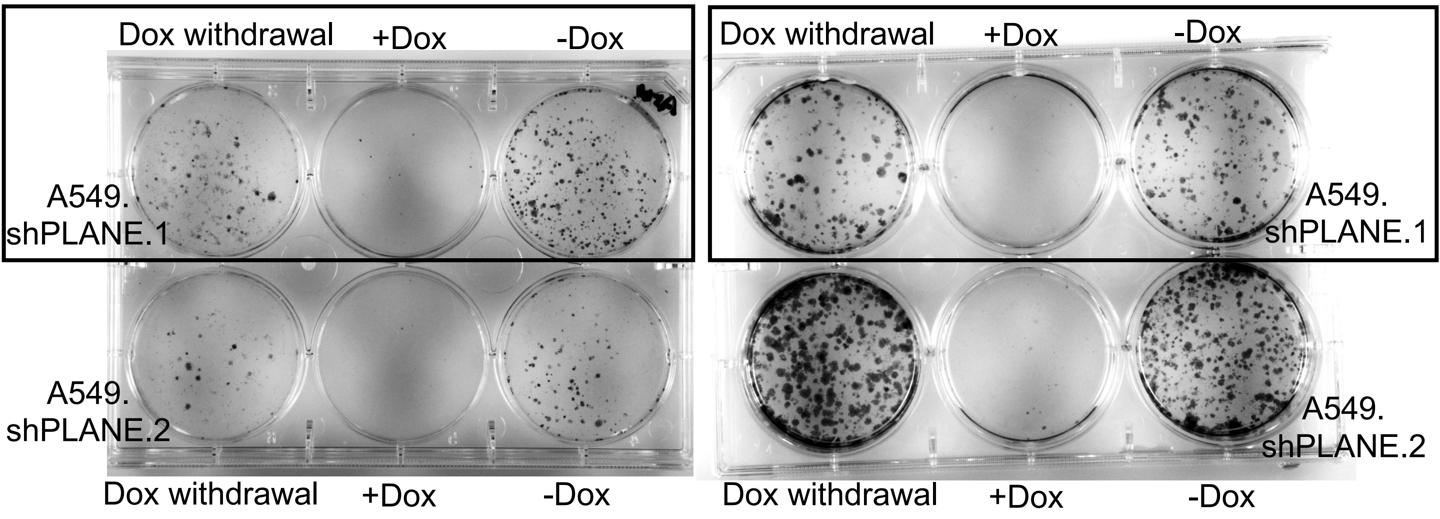

**Original data for colony formation assays in A549.shPLANE.2 cells with PLANE knocked down by shRNA as shown in Figure 2f**

The correct version:

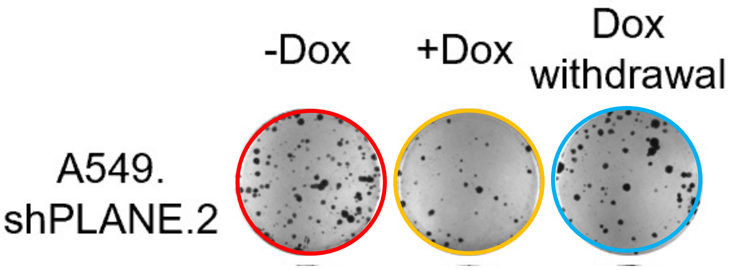


The original data used for the presentation as shown above:

(September 25, 2020)

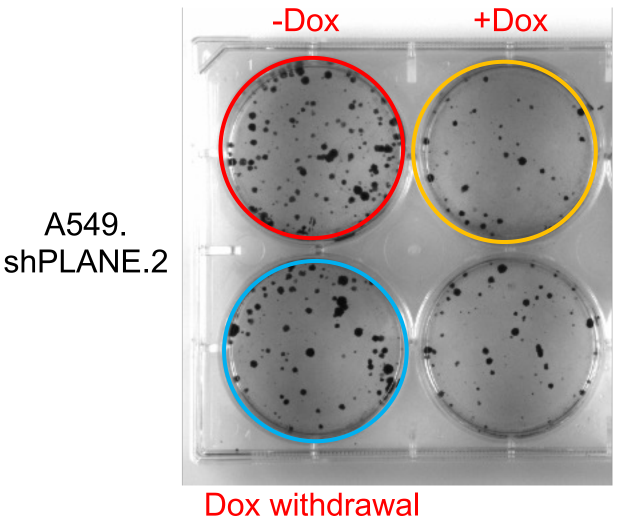


The additional repeats :

( May 16, 2019 )

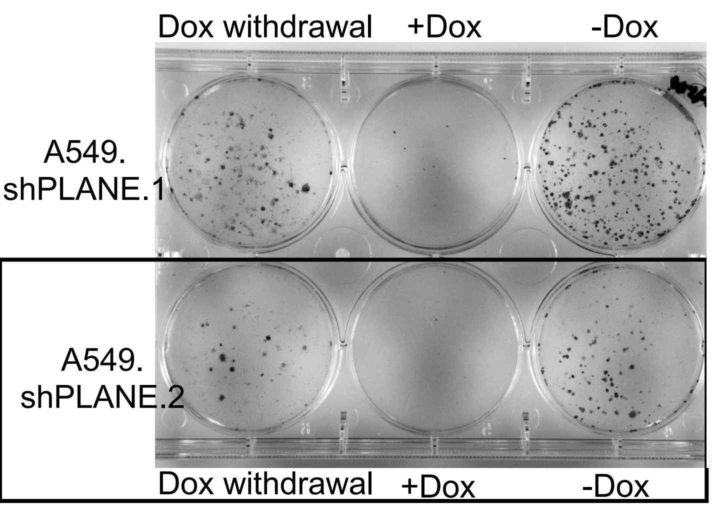


( April 30, 2020 )

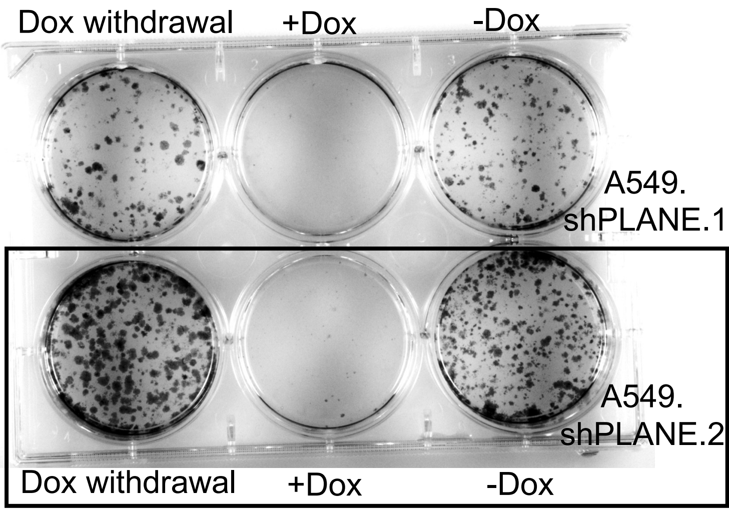

**Original data for colony formation assays in H1299.shPLANE.1 cells with PLANE knocked down by shRNA as shown in Figure 2f**

The correct version:

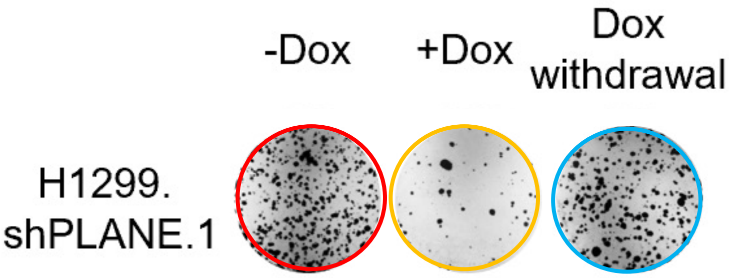


The original data used for the presentation as shown above:

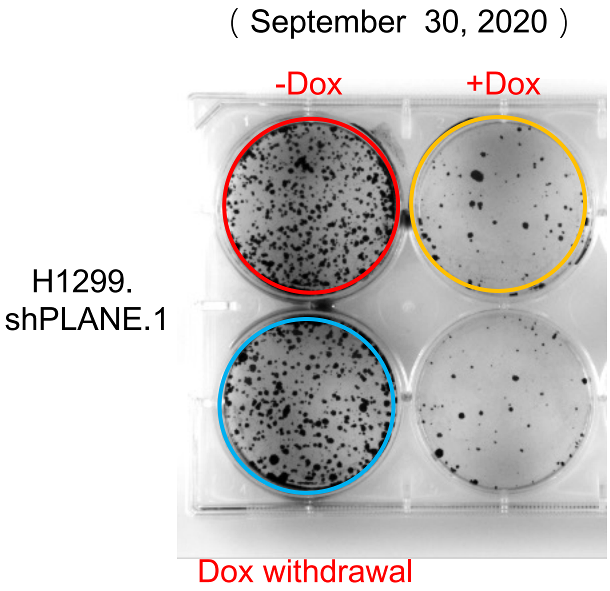


The additional repeats :

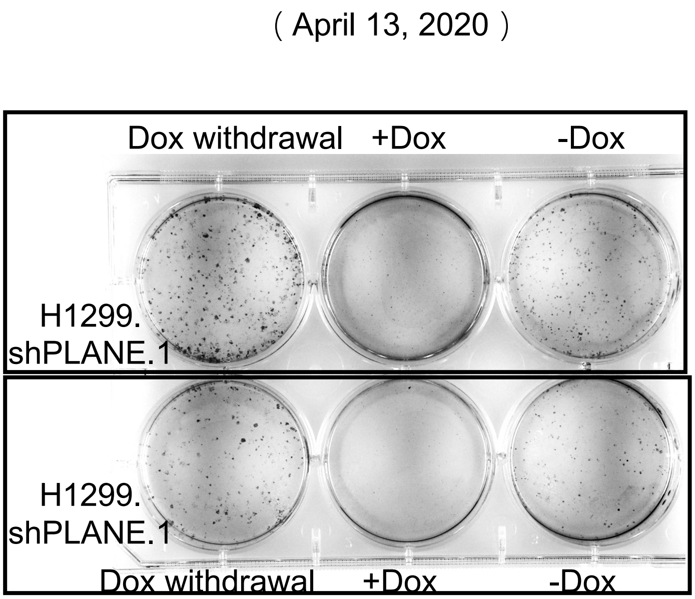


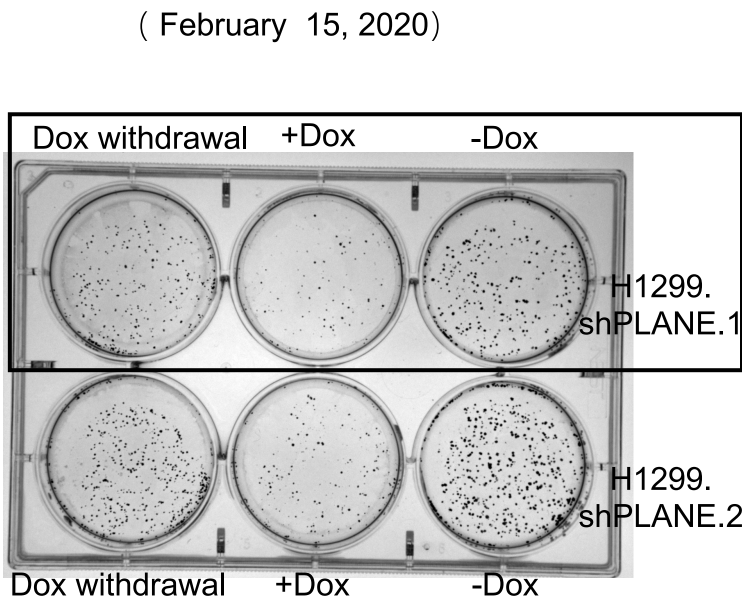

**Original data for colony formation assays in H1299.shPLANE.2 cells with PLANE knocked down by siRNA as shown in Figure 2f**

The published version:

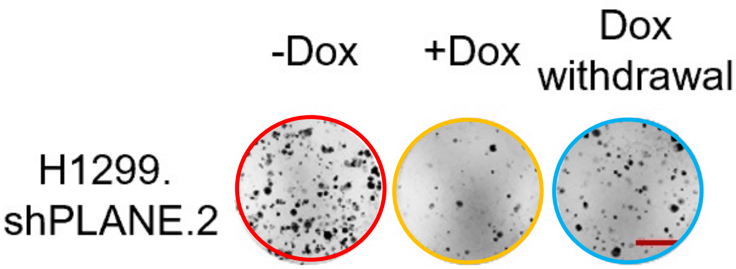


The original data used for the presentation as shown above:

( November 11, 2020 )

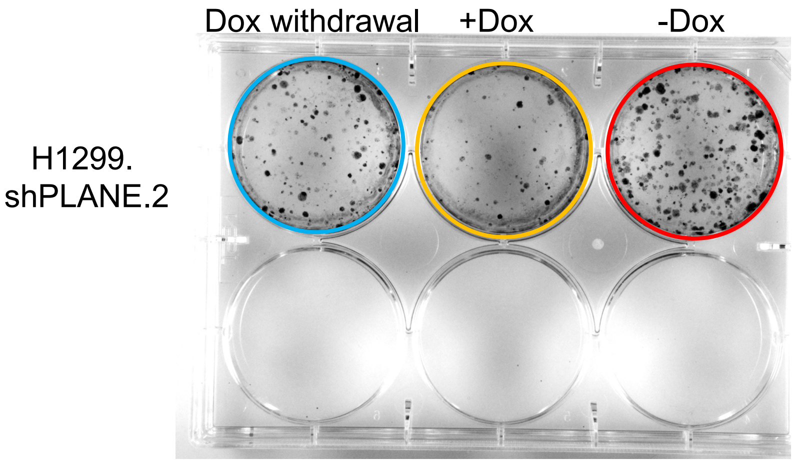


The additional repeats :

( April 13, 2020 )

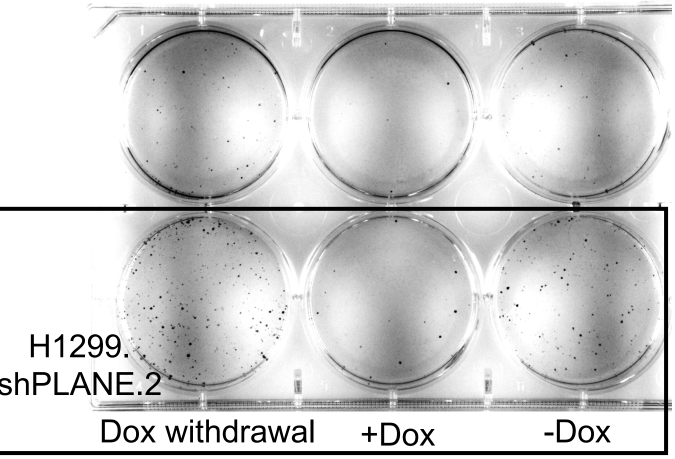


( February 15, 2020 )

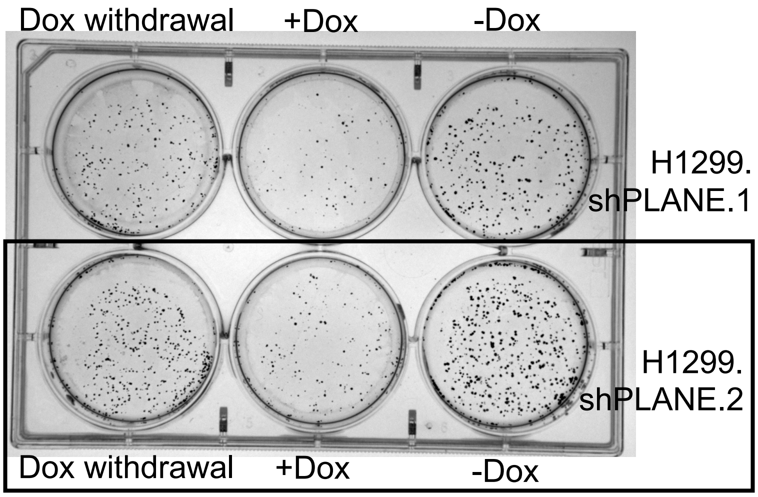

Supplement: Supplementary file 1 — Raw data [file 41467_2025_59086_MOESM1_ESM.pdf]
